# OCCAM: prediction of small ORFs in bacterial genomes by means of a target-decoy database approach and machine learning techniques

**DOI:** 10.1093/database/baaa067

**Published:** 2020-11-18

**Authors:** Fabio R. Cerqueira, Ana Tereza Ribeiro Vasconcelos

**Affiliations:** Department of Production Engineering, Universidade Federal Fluminense, Rua Domingos Silvério s/n, Petrópolis, 25 650-050, Rio de Janeiro, Brazil; Graduate Program in Computer Science, Universidade Federal de Viçosa, 36570-900, Minas Gerais, Brazil; Laboratório Nacional de Computação Científica, Rua Getúlio Vargas 333, Petropólis, 25 651-071, Brazil

## Abstract

Small open reading frames (ORFs) have been systematically disregarded by automatic genome annotation. The difficulty in finding patterns in tiny sequences is the main reason that makes small ORFs to be overlooked by computational procedures. However, advances in experimental methods show that small proteins can play vital roles in cellular activities. Hence, it is urgent to make progress in the development of computational approaches to speed up the identification of potential small ORFs. In this work, our focus is on bacterial genomes. We improve a previous approach to identify small ORFs in bacteria. Our method uses machine learning techniques and decoy subject sequences to filter out spurious ORF alignments. We show that an advanced multivariate analysis can be more effective in terms of sensitivity than applying the simplistic and widely used *e*-value cutoff. This is particularly important in the case of small ORFs for which alignments present higher *e*-values than usual. Experiments with control datasets show that the machine learning algorithms used in our method to curate significant alignments can achieve average sensitivity and specificity of 97.06% and 99.61%, respectively. Therefore, an important step is provided here toward the construction of more accurate computational tools for the identification of small ORFs in bacteria.

## Introduction

It is well known that small proteins (<100 amino acid residues) play a vital role in several important cellular activities in all sorts of organisms, including prokaryotes (1, 2). Small proteins in prokaryotes may act, for instance, as intercellular signals, intracellular toxins and kinase inhibitors (3–5). Hobbs et al. cite additional key functions such as: acting as antibiotics, structural role, alteration in membrane fluidity, acting as metal chaperones and regulation in larger proteins (6). In *Salmonella*, the protein MgtR composed of only 30 amino acids binds to the virulence factor MgtC, causing its degradation (7). In *Bacillus subtilis*, the protein Sda of only 46 amino acids represses sporulation by inhibiting kinase KinA (8, 9). In *Staphylococcus aureus*, small proteins composed of 20 to 22 amino acids are excreted during infection causing membrane disruption of neutrophils, leading to cell lysis (10).

It is also worth mentioning the small proteins that are encoded by bacterial dual-function small RNAs (sRNAs) that are RNA molecules composed of 50 to 350 nt and regulate gene expression as well as proteins (11–16). Even though the role of proteins encoded by sRNAs is still awaiting elucidation, it is likely that such proteins act in many cases in conjunction with the riboregulation in the same physiological pathway, both in a complementary manner and independently (16, 17).

An important type of short peptide, termed signal peptide, composed of 20–30 amino acids, has also been long reported in the literature (18). Signal peptides are present at the N-terminus of a vast number of nascent proteins that are aimed for the secretory pathway. In bacteria, the signal peptides mark proteins to direct them to the SecYEG protein-conducting channel. In addition, signal peptides have been shown to play a key role in regulation of protein biogenesis (18).

Although the importance of small proteins is widely recognized, the genome annotation processes have not yet achieved satisfying results concerning the detection of small open reading frames (ORFs) (2, 19–21). In particular in this work, we address the challenge of *in silico* detection of small ORFs in bacteria, for which the current obstacles result in a very reduced number of annotated small ORFs in the main public repositories. The difficulties can be explained by the fact that small ORFs are hidden in the huge number of random ORFs that a genome might contain. Additionally, small ORFs present peculiar characteristics that are not yet known. As a result, both *in silico* approaches, namely intrinsic and extrinsic, are affected (1–3, 5, 19–23).

In the intrinsic approach, the sequence under analysis is scrutinized regarding its coding potential as well as the presence of distinct features such as the ribosome binding site (RBS). However, the reduced size of sORFs means also reduced amount of information. Furthermore, as described by Warren et al. (22), a small ORF might present a composition that is completely anomalous when compared to large ORFs. The authors show that the guanine-cytosine (GC) content in several prokaryotes might be highly variable, meaning that no pattern can be inferred. Hemm et al. also demonstrate the small ORF peculiarity in a previous work, where they confirm the existence of short-protein coding genes without any discernible RBS (24).

The extrinsic approach, in turn, is based on similarity between conserved sequences among different species, i.e. the procedure is essentially searching for homologous sequences. However, the search is carried out in databases (DBs) of proteins that are already annotated, which is not an interesting solution for small ORF detection because the number of annotated small ORFs up until now is extremely low (3, 22).

For eukaryotes, the studies to detect small ORFs have been intensive (25–28). It is also worth mentioning the availability of some important tools for small ORF detection in eukaryotes: sORF finder (29), PhyloCSF (30), HAltORF (31) and uPEPperoni (32).

The number of computational methods for identification of small ORFs focused on prokaryotes, however, is scarce. Warren et al. described a methodology for a large-scale analysis of missing genes in all annotated prokaryotic genomes (22). In the course of this paper, we refer to the Warren and colleagues’ work as MGP (missing genes in prokaryotes). In MGP, the authors use the extrinsic approach trying to circumvent its limitations through the comparison also of unannotated parts of the genomes. Suppose gene *a* in genome *A* and gene *b* in genome *B* are yet unknown. If *a* is similar to *b*, then both might be identified when we compare *A* to *B*. However, if the goal is a broad analysis using all annotated prokaryotic genomes, the computational costs involved might turn the whole study into an infeasible process. To overcome this issue, the authors developed a methodology—explained in the next section—combined with a high-performance computing platform to identify overlooked genes in the annotation of all prokaryotic genomes in GenBank. The results showed that, indeed, there is strong evidence that the number of small genes systematically ignored in prokaryote genome annotation is very high. Still, the authors demonstrate that this fact occurs mainly due to the incapacity of current computational tools to detect small ORFs. They list 1153 missing gene candidates, where the vast majority are small (≤300 nt). In addition to a rather variable GC content, Warren and colleagues point out that only 127 candidates could have an RBS assigned. Furthermore, many such identified genes could not be detected by traditional computational tools for gene prediction such as Glimmer (33), EasyGene (34) and GeneMark (35), possibly because of the yet-unknown divergent characteristics of small ORFs.

Up until 2012, the work of Warren et al. was the only one to tackle such a large-scale analysis for prokaryotes. A similar effort that led to a computational tool termed COMBREX was then published in 2012 by Wood and coworkers (23). In COMBREX, the program Glimmer is used to select missing gene candidates that are then submitted to a search for homologous sequences using BLAST (36). The authors also demonstrated that most of their uncovered genes are small.

Aiming to identify small genes, Goli and Nair extracted 117 coding attributes from six prokaryotic organisms: physicochemical and conformational properties (65 attributes), *k*-mer frequencies (84 attributes), GC content and its fraction at different codon positions (4 attributes), codon usage bias (6 attributes), amino acid properties (2 attributes) and rho statistic (16 attributes) (37). By using Fast Correlation Based Feature Selection, the authors could reduce the initial set to 22 attributes with high discriminant power. ML algorithms were then used on the final dataset to build a predictive model that was evaluated by cross validations and presented high accuracy. However, the authors did not perform genome-wide experiments. Their results were limited to validations in the training set composed of pre-selected coding and non-coding sequences of DNA fragments ( i.e. not entire ORFs) of the six chosen organisms.

ÓhÉigeartaigh et al. developed SearchDOGs, a software that uses BLAST searches combined with a synteny analysis approach to automatically detect missing genes in bacterial genomes (38). The authors identified 155 gene candidates in the *Shigella boydii* sb227 genome, including 56 candidates of length < 60 codons. As stated by the authors, the analysis proposed in the method is limited to species that are phylogenetically close. Their approach identifies unannotated genes for which an ortholog exists and is annotated in another species.

A recent preprint was released by Li and Chao describing a *de novo* approach based on ML, namely, support vector machines, to identify small ORFs in bacterial genomes (39). First, the authors identified discriminant attributes by studying known small proteins in *Escherichia coli*. Then, they built an ML model that could achieve 92.8% of accuracy in a 10-fold cross validation. Further experiments with 549 bacterial genomes were performed using their model, leading to the identification of >100,000 novel small ORF candidates. Unfortunately, the authors do not show important statistical measures such as sensitivity and specificity. Consequently, when it is described that a discriminant probability of 0.9 was used in further experiments, for example, it is possible that their results presented good specificity but poor sensitivity. Additionally, we found no mention regarding the availability of their computational tool.

In spite of the fact that the work of Warren et al. has provided a great contribution demonstrating that small genes have been systematically ignored in the annotation of prokaryote genomes, the MGP methodology has room for many improvements, especially concerning sensitivity (40). Furthermore, the authors present the method, but they did not make a computational tool available. In this work, we propose an improvement of MGP with a focus on small ORFs in bacteria, as the identification of long ORFs—i.e. those that encode proteins with 100 or more residues—has already well-established solutions. We describe a method built upon ML techniques together with the use of decoy subject sequences to improve elimination of false BLAST alignments. We show that this is more effective than using traditional *e*-value thresholds. Our results also demonstrate that the whole procedure, named here OCCAM, could maintain specificity and sensitivity at high levels, and obtain similar specificity yet superior sensitivity when compared with MGP.

## Methods

### Extraction of queries and construction of the subject DB

The first phase of our approach is to build queries and the subject DB, similarly to MGP. The idea is to provide the possibility of comparing intergenic ORFs of sequenced genomes to all possible ORFs of the same genomes. Only ORFs with length in a certain range are considered. The sequences from which the ORFs are extracted are replicons (chromosomes and plasmids) of all bacterial genomes available in the RefSeq repository of the National Center for Biotechnology Information (41). Data were downloaded in February of 2014, totaling 2281 genomes. Considering only genomes whose identifier starts with ‘NC’, i.e. finished genomes, and ignoring sequences for which the annotation has inconsistencies, we could select 4239 replicons to proceed with our work.

**Figure 1.**
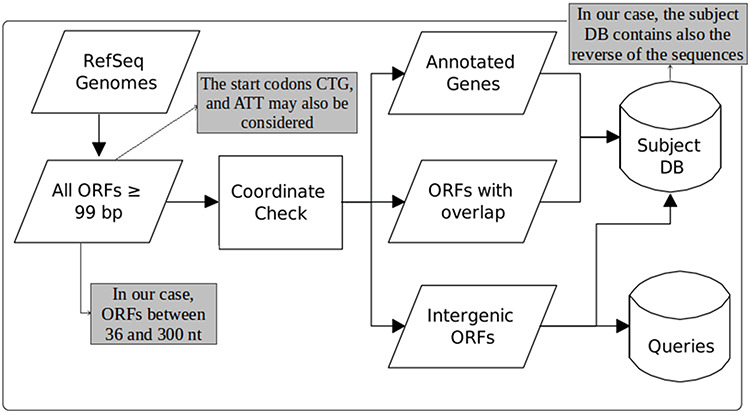
Construction of queries and the subject database (adapted from the work of Warren et al. (22)). The original figure can be identified by white-background shapes. Illustrated in gray, the differences in our case are: only ORFs of length between 36 and 300 are considered; the start codons CTG and ATT may also be taken into account in addition to ATG, GTG and TTG; and the sequences that compose the subject DB are the ones coming from the three groups plus their reverse.

Figure 1, taken from the work of Warren and co-workers (22), illustrates the steps for each DB construction. ORF extraction is performed like in MGP with the difference that our focus is on small ORFs, including very small ORFs, i.e. we consider ORFs of length between 36 and 300 nts. Note that in MGP long ORFs are allowed and very small ones are ignored (they consider only ORFs ≥99 nt). Another difference is that in MGP only the start codons ATG, GTG and TTG are taken into account. Our computational tool uses these same start codons as default, but allows also the use of CTG and ATT in its parameterization. Furthermore, in our case, although all ORFs of the available bacterial genomes are extracted, the user can consider as query only ORFs of organisms of interest instead of ORFs of all organisms in RefSeq as done in MGP.

To build the queries and the subject DB, as can be seen in Figure 1, the coordinates of the extracted ORFs are compared to the coordinates of the current annotation. Then, the ORFs are split into three categories: (1) *annotated*—those corresponding to already-annotated ORFs; (2) *entity-overlapping*—those overlapping with some annotated entity (coding genes, RNA genes, pseudogenes, etc.); and (3) *intergenic*—those isolated from any other already-annotated genomic entity. After this separation, all ORFs are translated to amino acid sequences. The query datasets are then constructed with ORFs of group 3, whereas the subject DB is conceived with all groups. However, it is important to highlight here another important difference regarding MGP. In our case, the subject DB is filled with the sequences of all groups in addition to the reverse of these sequences. The use of reverse—or decoy—sequences is a fundamental feature to permit the identification of random alignments, as described in the following sections.

Another important thing to point out is that we further split the resulting ORFs into two extra categories: very small ORFs (vsORFs), which are those composed of 36 (11 residues) to 90 (29 residues) nucleotides; and small ORFs (sORFs), which are those composed of 93 (30 residues) to 300 (99 residues) nucleotides. This separation is important because the BLASTP parameters are optimized in the case of query sequences containing fewer than 30 amino acids. Also, such a stratification makes possible to use more restrictive filters on alignments of vsORFs that are even harder to identify. Note that we are aware that ‘small’ and ‘very small’ are arbitrary and ambiguous terms. Hence, our intention is by no means to establish a definition concerning ORF size ranges. On the other hand, this distinction is necessary here for practical reasons, mainly the reasons described above, i.e. an optimized BLASTP execution and a different filtering process of the resulting alignments, since very small ORFs are more easily confounded with random sequences. Note that separate runs are performed for both categories (distinct datasets and distinct parameterization), which means that the category split also facilitates the reporting of results. It is also important to clarify that the minimum size of 11 residues was defined according to the shortest proteins that we could find in the literature at the time that we started our research. Recent works, however, have been described proteins < 11 residues long (42). Even though such proteins are out of the scope of our work, the size ranges are parameters of our programs, which means that the extraction of queries and the construction of the subject DB, as illustrate in Figure 1, can be performed with alternative ORF lengths according to the user needs.

**Figure 2.**
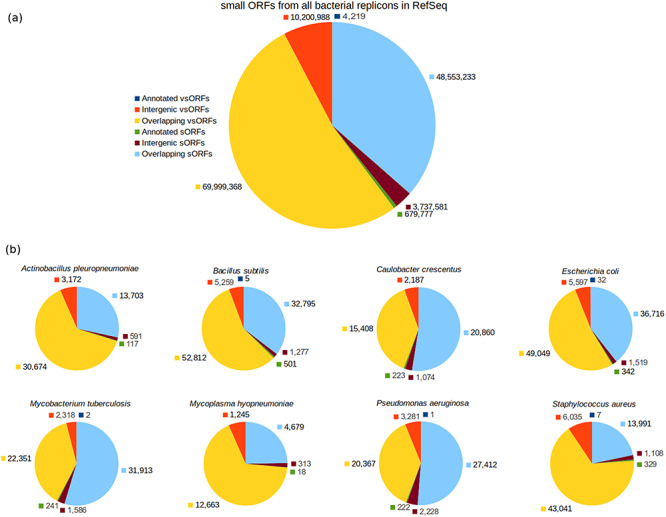
Category breakdown of small ORFs. (a) The whole set of small ORFs obtained for all bacteria in RefSeq. (b) Small ORFs of the eight bacteria that we used in our experiments.

The ORF category breakdown is shown in Figure 2. As can be seen in the chart of all small ORFs (Figure 2a), the number of annotated small ORFs is very low, specially considering that a bacterium has typically thousands of protein-coding genes composed of 100 or more amino acid residues. The number of annotated vsORFs is particularly small, i.e. the amount of 4219 means an average value smaller than 2 annotated vsORFs per bacterial genome. Another interesting thing is the room for finding new small ORFs, as can be noted by inspecting the intergenic portions. The number of intergenic small ORFs is three orders of magnitude greater than the number of annotated small ORFs (for both sORFs and vsORFs). The overlapping regions also express a significant potential for new small ORFs. However, similarly to Warren and co-workers, we preferred to explore only the intergenic regions.

### Datasets used in the experiments

For our experiments, we selected the genomes of eight bacteria: *E. coli* str. K12 substr. MG1655, *Bacillus subtilis* 168, *Pseudomonas aeruginosa* PAO1, *Staphylococcus aureus* MRSA252, *Actinobacillus pleuropneumoniae* serovar 5b L20, *Caulobacter crescentus* CB15, *Mycobacterium tuberculosis* H37Rv uid57 777 and *Mycoplasma hyopneumoniae* 7448, for which the resulting ORF datasets were named with the prefixes: Ec, Bs, Pa, Sa, Ap, Cc, Mt and Mh, respectively, alluding the bacterium names. Additionally, the suffixes vso and so were also used to compose the dataset names to indicate whether a given dataset contains vsORFs or sORFs, respectively. Therefore, Bs_vso and Bs_so, for instance, are the names of the datasets containing vsORFs and sORFs, respectively, of *B. subtilis*. Those bacteria were chosen because of their broad use in biological studies and/or their importance in terms of economy as well as public health (43–47). The accession numbers of each genome can be found in supporting File S1. The number of intergenic small ORFs that served as query sequences in the BLAST searches—for each of the 16 resultant datasets—can be visualized in Figure 2b (red for vsORFs and brown for sORFs). Also, the start codon distribution in each dataset is depicted in supporting File S2.

Control query datasets were additionally built using small ORFs of *E. coli*, *B. subtilis*, *P. aeruginosa* and *S. aureus* to measure how accurate OCCAM is to identify significant alignments among the plethora of random alignments that occur in the case of short sequences. Each control dataset, regarding a bacterium, was built using the sequences of annotated small ORFs, which are thus positive cases, plus shuffled sequences of intergenic small ORFs, which are negative examples, similarly to the work of Ladoukakis et al. (26). In this way, it becomes easier to identify true/false positives as well as true/false negatives when evaluating data mining algorithms. If an annotated small ORF is detected by the computational tool as a missing gene, then it is obviously a true positive. If a shuffled sequence of an intergenic ORF is reported as a missing gene, then it is certainly a false positive. It is important to notice that the number of shuffled sequences in a control dataset is far greater than the number of annotated sequences. In the case of *B. subtilis*, for instance, the resultant vsORF control dataset contains five annotated vsORFs used as positive examples, and 5259 shuffled intergenic vsORFs as negative examples, as can be seen in Figure 2b. Therefore, the probability of our programs to identify an annotated (i.e. a positive) sequence as a missing gene by chance is close to zero.

The letter ‘a’—alluding ‘annotated’—was used to compose the names of the control datasets. Hence, Ec_avso and Ec_aso, for example, are the names of the control datasets containing annotated/shuffled vsORF sequences and annotated/shuffled sORF sequences, respectively, of *E. coli*. Therefore, as we used four bacteria, we ended up with eight control datasets, i.e. each bacterium gave rise to two datasets: one for vsORFs and another for sORFs.

The control datasets are also useful to show that decoy sequences can in fact be applied in an efficient approach to detect spurious alignments, as can be seen in the result section.

### BLASTP execution and alignment processing

Once the DBs are built, the next step is to execute BLASTP to compare query sequences to subject sequences for identifying homologs. As in MGP, we use mpiBLAST that promotes a huge gain in performance through parallelization of BLAST programs (48). After the search, we analyze the resulting alignments looking for evidence of new genes not reported in the current genome annotation.

In MGP, the resulting alignments of BLASTP are filtered using *e*-value ≤10^−5^. ORFs are then labeled in two stages. First, for each query, its best alignment—regarding *e*-value and identity—to a sequence of a distinct replicon is inspect. Thereafter, the query is labeled using the following classification:

*Absent annotation* (AA)—if the query aligns to an annotated ORF and the query coverage as well as the subject coverage are at least 80%;
*Genomic artifact* (GA)—if the query aligns to an entity-overlapping ORF and the query coverage as well as the subject coverage are at least 80%; and
*Potentially missing gene*—if the query aligns to an intergenic ORF pertaining to a different taxonomic family, the query coverage as well as the subject coverage are at least 80%, and the average coverage of any other alignment suggesting a different label is at least 20% less than the average coverage of the best alignment. This 20% criterion can be thought as a reinforcement of the evidence that the ORF is potentially a missing gene.

Those queries for which no alignment could be found according to the criteria above are labeled *unclassified*.

The second stage of MGP is to further analyze potentially missing genes to find evidence that they are really missing. In this step, ORFs labeled potentially missing genes are clustered. A pair of such ORFs are joined if they align to each other. The members of groups that contain at least two ORFs of distinct taxonomic family are labeled *missing gene* (MG). The restriction concerning different taxonomic families—used in the first and second stage for the missing gene case—is to guarantee enough phylogenetic distance so that the evidence provided by the alignment is strong, i.e. the alignment did not happen simply because the genomes have very similar sequences due to phylogenetic proximity.

Once more, some significant differences between OCCAM and MGP have to be described concerning the way ORFs are labeled according to the alignments.

The first distinction concerns the way random alignments are identified. Instead of using an *e*-value cutoff, our method applies a target-decoy database (TDDB) approach, which is largely used in mass spectrometry data analysis for protein identification (49–51), associated with ML algorithms, as proposed previously also for proteomics (52). Our goal is to filter out random alignments without compromising sensitivity. Especially in the case of very small ORFs, where *e*-values tend to be higher, the threshold value 10^−5^ might be very stringent. The gain in sensitivity provided by our method to separate random alignments is a major contribution provided in this work. We also pick the best query alignment, but using the probabilities generated by the resulting ML model (see details later on in the text), instead of considering isolated parameters such as *e*-value and identity.

Second, regarding the criteria to identify evidence of homology, OCCAM considers a minimum query and subject coverage of 80% for sORFs, as in MGP. However, we realized that the occurrence of this percentage is not rare in random hits of vsORFs—which are not covered in MGP. In the dataset Ec_avso, for instance, a wrong hit was reported with query coverage = 92.31%, subject coverage = 100% and an identity = 84.62%. Therefore, it is necessary to consider a higher minimum coverage. Additionally, a minimum percentage of identity is also necessary, as can be seen in the same example. We also found examples in our datasets that have shown not to be random, where the coverage percentage was high for both query and subject, the *e*-value was very low, but the percentage of identity was around 40%. A hit with such a low identity is difficult to be trusted as a sign of homology (the percentage of conservation was even lower). It reinforces the need of considering also a minimum value for identity. Analyzing the control datasets, we could notice that a minimum of 80% for the three variables: minimum query coverage, minimum subject coverage and minimum identity is a suitable value for sORFs, while for vsORFs—which are more prone to random alignments—a minimum of 90% for the same variables is more adequate.

The third difference between OCCAM and MGP is related to the stage to provide more evidence that the MG classification is correct. In the work of Warren et al., the second phase of the alignment analysis is a clustering procedure. It aims at placing similar ORFs that were considered potentially missing genes in the same group to provide an evidence that they are indeed missing genes. It is important to note, however, that the MGP clustering is possible because the BLASTP search takes as queries all ORFs identified in all sequenced prokaryote genomes. However, the computational resources to perform such a high-cost search are rare. Warren et al. used a high-performance distributed platform to accomplish their work. They do not mention the time needed to perform such a huge BLASTP search in their work. However, especially in our case where a much bulkier query and subject DBs are obtained due to considering very small ORFs, such a BLASTP search would probably take months to be accomplished, even in a high-performance platform. As can be seen in our experiments, the TDDB+ML step for separating significant hits plus the above-mentioned homology criteria (minimum coverage/identity) were enough to guarantee a good specificity, which is a challenging aspect of computational solutions (40). As a result, we could eliminate the clustering procedure without compromising the quality of the results. With the exclusion of the clustering phase, it is possible to use as queries only ORFs of the target organisms, making running time feasible even in simpler computational platforms. Note that in our approach the subject DB still includes the small ORFs of all sequenced bacterial genomes in RefSeq. Additionally, the *α* score—proposed by Warren et al. —and the *β* score—conceived in this work—are provided as options to the user to further assess the quality of the classification. See the details of those scores in subsequent sections.

Finally, an important difference between our method and MGP concerns ORFs classified as GA. In MGP, this type of ORF is ignored. However, overlapping genes have been widely reported in the literature (53). For this reason, although we keep the classifications GA and MG distinct according to the alignment—as describe earlier in the text—and do not include entity-overlapping ORFs in the query DB, GAs might be also considered as potentially missing genes in our work. To consider a GA query as potentially missing gene, the alignment must be to a replicon of a different taxonomic family—just as in the case of a query that aligns to an intergenic ORF—so as to minimize the chance that the alignment is meaningless.

### The TDDB approach

The most successful procedure hitherto to identify and quantify proteins in a complex mixture is the so-called shotgun proteomics for which the strategy liquid chromatography coupled with tandem mass spectrometry (LC-MS/MS) is a fundamental part (54, 55). In this procedure, thousands of spectra—each corresponding to a peptide—are generated. For each spectrum, the goal is to reveal the sequence of the peptide represented by the observed set of peaks. Typically, sequences are assigned by performing a search in a protein sequence DB from which computationally produced peptide sequences are compared to the pattern of peaks of each spectrum. The best spectrum hit, i.e. the sequence assignment presenting the best scores, is reported as the most probable sequence of the spectrum (56). However, the error rate in this spectrum interpretation process is very high, which led to studies for automatizing curation of sequence assignments (52, 57, 58).

**Figure 3.**
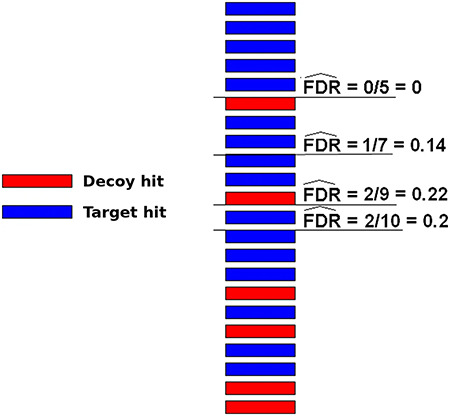
Illustration of how FDRs can be estimated using a composite target-decoy DB (from the work describing the method MUMAL by Cerqueira et al. (52)). In this example, only one score (univariate analysis) is used. The hits are presented in descending order, i.e. the highest score is on top. For a given threshold value, the number of decoy hits with score greater or equal to this value is used to estimate the number of wrong matches among target hits obtained using the same threshold.

A very popular method to evaluate spectrum-sequence matches is the target-decoy search strategy. In this method, besides using the target protein sequences, a DB composed of decoy sequences are also included in the assignment procedure. There are roughly two ways to use such sequences. In one approach, target and decoy DBs are used independently, which means that two separated searches are performed using one subject DB at a time. In another strategy, a unique composite TDDB is created and only one search is necessary. Typically in both schemes, decoy DBs may be conceived by reversing or shuffling the target DB sequences (59). For any of these methods, the resulting false sequences have to be produced in a way that it is reasonable to assume that a wrong hit has an equal probability to come from either DB (target or decoy). In this case, the number of decoy hits is an excellent estimate to the number of wrong hits among target ones. Therefore, one can try different threshold values for the hit scores and measure the false discovery rate (FDR) counting the number of decoy hits (49–51). The threshold values that indicate an acceptable quantity of random target hits ( e.g. 1% FDR) are then taken to filter out wrong sequence assignments. Figure 3 illustrates how FDR can be estimated using decoy sequences. Note that the decoy hits are used exclusively to predict the number of wrong matches among target hits, which are, naturally, the only results of interest, i.e. decoy hits are subsequently disregarded.

**Figure 4.**
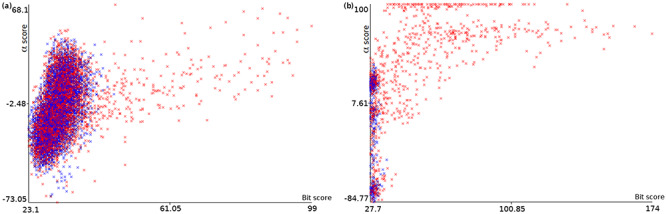
Plots of *α* score vs. bit score to *E. coli* datasets: Ec_vso (a) and Ec_so (b). Blue data points are decoy hits, while red data points represent target hits.

Here, the TDDB technique is used for BLASTP searches. To our knowledge, this is the first work that applies TDDB to this purpose. We have chosen to construct a composite subject DB, i.e. target and decoy sequences are maintained in only one DB, meaning that just one search is needed. Additionally, decoys are produced by reversing target sequences keeping the amino acid distribution.

Let *s*[*i*..*j*] represent a subsequence of a sequence *s* with size *n*, where *i* and *j* ∈ 1, 2, ..., *n* and *i* ≤ *j*. A decoy sequence is obtained from an amino acid sequence *s* by concatenating the letter ‘M’ (methionine) to the reverse of *s*[2..*n*].

The objective to be achieved with TDDB is to replace the use of a fixed *e*-value threshold as seen in the MGP method (*e* ≤ 10^−5^) because it might mean a poor sensitivity. Especially in the case of vsORFs, greater *e*-values are common in good hits. Therefore, instead of defining a fixed threshold, a TDDB approach with multiple scores is used to identify random alignments, increasing sensitivity without worsening FDR.

Figure 4 illustrates how a TDDB approach might help to identify random BLASTP alignments. This is similar to the illustration presented in Figure 3, but this time real data are shown in a two-dimensional space. The figure shows two plots of *α* score vs. bit score where data points represent alignments. Red data points indicate alignments of intergenic ORFs to target sequences, whereas blue data points indicate alignments of intergenic ORFs to decoy sequences. The plots concern the *E. coli* datasets: Ec_vso and Ec_so, respectively. In Figure 4a and b, it can be seen a well-defined cloud of random alignments (mixed blue and red data points), where approximately one half is composed of alignments to target sequences, while the other half is composed of alignments to decoy sequences. Significant hits can be identified as the red data points outside the dense cloud, because regions with only target hits mean regions with $\widehat{FDR}=0$. Therefore, decoy hits can be used to pin down target hits that arose by chance. Similar plots can be viewed in supporting File S3 for the other datasets. We use ML techniques to perform a multivariate analysis of decoy hits in order to select target hits of interest (regions of low FDR), as described below.

### Classification attributes

Before providing the details concerning the ML techniques used in this work to identify the cloud of random hits, it is important to describe the attributes that the learning algorithms take into account to evaluate alignments of intergenic ORFs to sequences in the subject DB.

Six attributes are used. Four of them are standard BLAST alignment quality parameters: bit score, percent query coverage, percent subject coverage and percent identity. The other two attributes are the scores *α* and *β*.

The *α* score—proposed in MGP—is also called uniqueness score and is a measure of the robustness of a classification. Let *I* be the average percent identity of an alignment given by averaging the percent identity values with respect to the query and subject length. For instance, the percent identity regarding the query is the number of identities in the alignment divided by the length of the query times one hundred. The uniqueness score is then given by: *α* = *I*_1_ − *I*_2_, where *I*_1_ is the highest *I* among the alignments that support the classification, while *I*_2_ is the highest *I* among the alignments that indicate an alternative classification.

The *β* score—proposed here—is a count of alignments that corroborate the classification given by the best alignment. Thus, it resembles, to a less extent, the clustering process in MGP. A high *β* value provides more confidence to the ORF categorization because it means that besides the best alignment indicating a certain ORF category, the query ORF also aligns to other subject ORFs that put this query in the same category. Therefore, a high *β* score for an MG hit, for instance, means that the query ORF aligns to many intergenic ORFs of distinct organisms, which is a strong indication that the assignment of the MG category to the query ORF is correct.

### Analyzing target and decoy hits by ML techniques to select significant hits

Although the TDDB technique provides an interesting solution to identify groups of significant hits, it is often not explored to its full potential because only one or two scores are typically used, even when there are other important scores related to the hit quality. Additionally, in a standard TDDB analysis, thresholds are pursued for each score individually, i.e. scores are not investigated in conjunction, which could define more interesting decision boundaries to separate random and meaningful hits.

To overcome the above-mentioned drawbacks, Cerqueira and coworkers proposed a method in shotgun proteomics termed MUMAL, where artificial neural networks (ANN) are used to perform a multivariate TDDB analysis. In their work, six scores are used as input attributes of an ANN to compose hyperplanes for defining decision boundaries that perform well even in non-linearly separable data (52).

In MUMAL, hits are formatted as an ARFF file to be used in Weka (60, 61), a suite of ML algorithms implemented in Java. Hits to decoy sequences are considered class 0, while hits to target sequences are labeled class 1. As already mentioned, a significant part of instances of class 1 are wrong hits (similarly to what is illustrated by red data points in the dense clouds of Figure 4a and b), i.e. they are very similar to hits of class 0 that are obviously incorrect. Therefore, as described by Cerqueira et al., datasets constructed this way can be considered as very noisy. After some experiments with ANN and support vector machine (62), the authors reported that ANNs were capable to cope better with their noisy data. They describe, in addition, that even not obtaining a very good ML model, due to the characteristics of the datasets, they compensate this issue with the use of decoys for identifying group of hits presenting low FDR. This is accomplished by trying different discriminant probabilities. Note that in MUMAL, the chosen ANN approach applies the sigmoid as activation function in order to produce output values in the range [0, 1] that can be interpreted as probabilities. The value 0.5 is the default discriminant probability to separate instances of class 0 and class 1. However, other probability thresholds are explored to estimate FDR by the equation:

(1) \begin{equation*} \widehat{FDR} = \frac{D_T}{N_T\,\textrm{-}\,D_T}, \end{equation*}

where *D*_*T*_ is the number of decoy hits with probability greater or equal to threshold *T*, and *N*_*T*_ is the total number of hits (decoys and targets) obtained using the same threshold value. All discriminant probabilities that lead to FDRs varying from 0% to 100% are reported such that the user has the chance to choose the set of hits presenting the most appropriate FDR. Typically, an FDR of 1% is chosen because Elias et al. and Balgley et al. have shown that this value is the best trade-off between sensitivity and precision (49, 54).

Once more, we bring those ideas applied to shotgun proteomics to our problem. As in MUMAL, we also format the data as an ARFF file to use an ANN approach in the Weka suite, as recommended by the authors. Given an intergenic ORF, if its best alignment is to a decoy sequence (reverse sequence in the subject DB), then it is labeled 0 (blue data points in Figure 4a and b). Otherwise, i.e. the ORF best alignment is to a target sequence, then the label 1 (red data points in Figure 4a and b) is assigned.

An important contribution in our case, however, is that we use an ANN approach associated to an algorithm implemented in Weka called threshold selector. This algorithm changes the mid-point probability threshold of a classifier in order to optimize some performance measure. In this work, precision concerning class 1 was chosen as the measure to be optimized. Hence, the ANN output probabilities are rearranged so that the group of instances with *P* ≥ 0.5 are exclusively of class 1 (maximum precision), i.e. with $\widehat{FDR} = 0$. Furthermore, we use the option of the threshold selector algorithm (TSA) to expand the ANN output probabilities to the interval [0, 1], i.e. probabilities are normalized so that the lowest value is mapped to 0, while the greatest value is mapped to 1. As a result, the new probabilities provide a more consistent notion of the hit quality. In MUMAL, due to the noisy aspect of the datasets, the raw probabilities given by the ANN are useful for FDR estimation, as stated above, but useless to assess the hit quality individually. In our case, with the normalization provided by TSA, probability values become useful to assess the alignment.

It is also important to highlight that the division in two groups performed by TSA, i.e. the separation of instances according to the 0.5 mid-point, provides the identification of the cloud of random hits. Instances with *P* < 0.5 are hits in the group corresponding to the cloud, while instances with *P* ≥ 0.5 (the group with no decoys, i.e. $\widehat{FDR} = 0$) represent hits out of the cloud, i.e. hits regarded as significant alignments. In a sense, decoy hits are thus used to make the ML algorithms learn what is a random hit. The obtained model is then able to relocate those label-1 instances that are detected as random to the label-0 group.

At this point, it is important to call attention to the peculiar usage of classification algorithms in OCCAM. Normally, supervised algorithms use some training set to build a predictive model to be used in future examples. Hence, the goal is typically to maximize model generalization to assure accuracy in coping with unknown instances. For this reason, the obtained models are usually validated on test sets that must be distinct from the training set in order to assure generality. In our case, however, the aim is to recognize and eliminate spurious alignments in a given dataset, i.e. it is not the case of applying the obtained model on a distinct data. Each alignment dataset will thus has its own overfitted ML model. For each dataset, the idea is to apply the ANN+TSA strategy to build an ML model that uses decoy hits to learn what is a wrong target hit in the given data. Remember that in the obtained model the mid-point probability threshold is changed by TSA in a way that examples with *P* ≥ 0.5 are all target hits, i.e. $\widehat{FDR} = 0$. In practice, the resulting model relocates spurious target hits—that were originally in class 1—to class 0. Therefore, instead of using one or two scores in a naive way—as illustrated in Figure 3—we propose the use of ML to provide a multivariate analysis of decoy-target hits, leading to more elaborated decision boundaries to define regions of low FDR values. Note that the most typical use of a target-decoy approach is exploring scores individually (as seen in Figure 3), which results in poor linear decision boundaries.

After this process of relocating low-quality target alignments, the resulting labels can be thought as useless alignments (label 0) and potentially useful alignments (label 1). What to do with label-1 hits is a matter of a particular objective, as explained in the next section. This relabeling procedure is illustrated in the result section for the case presented in Figure 4.

### Selecting hits of interest

After identifying non-random target hits (set of target alignments with $\widehat{FDR}=0\%$), by using a TDDB approach for the search and ML algorithms for assessing the resultant hits, the next phase is to deal with them according to the objective of a particular study. Note that not being random does not mean that the alignment is valuable. Often, it can be observed a certain similarity between genomes of phylogenetically distant organisms because they were originated from a common ancestor. Therefore, even in the case of MGs for which the alignments are considered only to ORFs of organisms of a different taxonomic family, it is common the situation where many alignments occur due to a remaining similarity, especially for small sequences. In this case, many good hits are expected, i.e. they are out of the cloud of random hits, but they do not mean function conservation.

As a consequence, some further filtering has to be done. Here, our objective is the same as described in the work of Warren et al., i.e. use homology to identify absent annotations and missing genes. For this reason, as already described, we use the value of 80% for query coverage, subject coverage and identity in the case of sORFs, and 90% for the same variables in the case of vsORFs. However, it is important to note that in OCCAM the user is free to apply any other filtering according to a specific aim. For instance, we have observed several significant alignments with query coverage = 100% and identity = 100%, but a low subject coverage (e.g. 30%). In many of these cases, the alignment is to a suffix of an already-annotated ORF. Such alignments might mean cases of inconsistent gene start sites, which is reported as a very frequent problem in the current annotation of prokaryotic genomes (63). These alignments were not further analyzed because they are out of the scope of the study reported here. However, it is important to highlight that such a study is an interesting possibility and demonstrates the importance of the flexibility provided by OCCAM, letting the user apply different additional filters according to convenience. Another example is the possible use of the scores *α* and *β*. In OCCAM, the user can apply different threshold values of these scores regarding a particular aim, for instance, to ensure strong specificity so as to minimize bench work. It is important to notice that our experiments show that the TDDB+ML approach alone can deliver very good sensitivity and specificity. But letting the user free to use whatever suits him/her is an important feature of a computational tool. The fact that our effort resulted in a free software is one of the important contributions of this work, especially because the resulting programs are highly parameterized.

**Figure 5.**
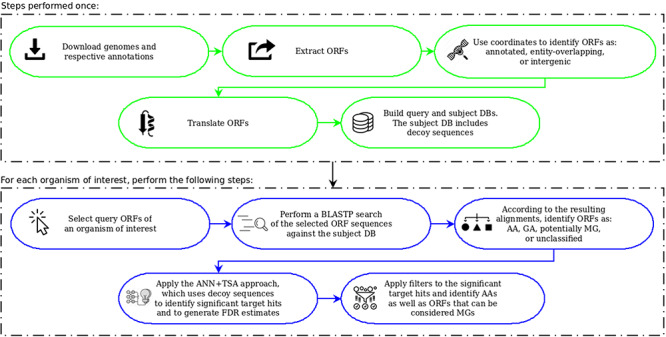
OCCAM pipeline. In green are the steps to be performed only once for a given version of genomes and respective annotations. In blue are the steps to be executed for each organism of interest.

### Summarizing the OCCAM method

Figure 5 summarizes the OCCAM pipeline. In green, it is illustrated the steps to build queries and the subject DB. Unless the genomes and annotations in use require an update, those steps have to be performed only once. Depicted in blue are the steps to be executed for each organism under study. The possibility to execute these steps only for the organism of interest is an important contribution of this work. It allows the use of affordable computational platforms, i.e. no complex and expensive high performance computing platform is needed to perform a huge BLAST search for queries extracted from all bacterial genomes. Note that this is particularly critical when considering vsORFs, because the number of such small ORF candidates in a single genome is far higher compared to the case where only longer ORFs are taken into account.

It is also opportune to highlight the data mining approach of OCCAM. Table 1 summarizes the data mining procedures of MGP and OCCAM to the case of missing genes. As the details were already described, we emphasize here just the main differences:

OCCAM uses decoy sequences associated with ML algorithms to filter out random BLASTP alignments. This is a major contribution as a univariate analysis, i.e. using only *e*-value to assess hit quality, is certainly less comprehensive than a multivariate ANN model. The use of *e*-value may be particularly problematic in the case of small ORFs for which greater *e*-values are common;
We realized that besides demanding high query/ subject coverage, filtering by a meaningful identity is also an important criterion to denote evidence of homology. We use an 80% minimum coverage/identity for sORFs and an 90% minimum coverage/identity for vsORFs. MGP uses an 80% coverage (and identity is not considered), but vsORFs are not included in their work;
Even though our results show that the steps described in the two items above are enough to obtain good sensitivity and specificity, *α* and *β* scores can be used to apply more stringent filters, for instance, to minimize *in vitro* experiments by focusing on small ORFs that present very strong evidence of being a missing gene. The *β* score, in particular, resembles in some extent the clustering procedure in MGP, and can be thus used in a similar fashion to a more restrictive filtering.

Finally, in addition to the characteristics described in Table 1, it is also important to remember that GA ORFs that fit the same criteria applied to potentially missing genes (including the minimum coverage/identity principle) are also considered missing genes in OCCAM.

**Table 1.** General differences in the data mining procedures of MGP and OCCAM to analyze an intergenic query ORF alignment for the case of a missing genes.

	Identifying significant BLASTP hits	Finding evidence of homology	Obtaining more evidence for potential MGs
**MGP**	*e*-value ≤10^−5^.	Significant query and subject coverage as well as the 20% criterion.	Clustering procedure.
**OCCAM**	A decoy DB approach plus ML techniques.	Significant query and subject coverage as well as identity.	Threshold values for the scores *α* and *β* may be used.

### Main metrics

For some of the results shown in the next section, the main metrics used to evaluate our approach are accuracy (Ac), sensitivity (Sn), specificity (Sp) and precision (Pr) described in Equations 2, 3, 4 and 5, respectively.

(2) \begin{equation*} {\rm Ac} = \frac{{\rm TP}+{\rm TN}}{{\rm TP}+{\rm TN}+{\rm FP}+{\rm FN}} \cdot 100, \end{equation*}

 

(3) \begin{equation*} {\rm Sn} = \frac{{\rm TP}}{{\rm TP}+{\rm FN}} \cdot 100, \end{equation*}

 

(4) \begin{equation*} {\rm Sp} = \frac{{\rm TN}}{{\rm FP}+{\rm TN}} \cdot 100,\vspace*{2pt} \end{equation*}

As already described, precision is also applied as the optimization parameter that TSA uses to relocate the mid-point probability threshold of the ANN classifier so that hits with *P* ≥ 0.5 are those out of the cloud of random hits ($\widehat{FDR}=0$).

(5) \begin{equation*} {\rm Pr} = \frac{{\rm TP}}{{\rm TP}+{\rm FP}} \cdot 100,\vspace*{2pt} \end{equation*}

In the presented equations, TP, TN, FP and FN are the number of true positives, true negatives, false positives and false negatives, respectively.

## Results and discussion

All experiments were run in a computer of 96 processors Intel® Xeon® CPU X7550, 2.00 GHz, 8 CPU cores and 396 GB of shared memory.

**Figure 6.**
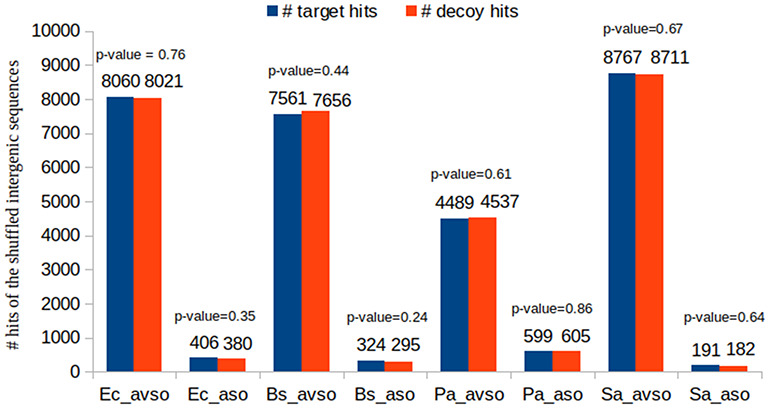
Showing that a wrong hit has the same probability of being decoy or target.

To the ORF searches, we used the command line BLASTP version 2.2, setting -evalue 100 so that no eventually correct alignment presenting a high *e*-value is missed. For vsORFs, the task parameter was set to blastp-short, whereas for sORFs this parameter was set to blastp. Note that while the blastp option means the execution of a standard BLASTP search, the blastp-short option means that BLASTP automatically optimizes the search parameters, such as the substitution matrix and the word size, for queries shorter than 30 residues. Except for the parameters evalue and task, all the other parameters of BLASTP were kept with their default values.

To deploy the described ML methods in our computational tool, we used the API of the Weka ML toolkit, version 3.7, that implements all algorithms and techniques already mentioned. For TSA, the parameter setting was: classifier = MultilayerPerceptron, evaluationMode = Entire training set, measure = PRECISION, and rangeCorrection = Correct based on min/max observed. For ANN, the parameter setting was: trainingTime = 1000. All other parameters for both algorithms were kept with their default values. Note that the standard ANN in Weka—which was used here—is an implementation of the Multilayer Perceptron architecture (62) with one hidden layer containing $\lceil {\frac {m+c} {2} } \rceil$ neurons, where *m* is the number of attributes and *c* is the number of classes. As we use six attributes and one class, the number or neurons in the hidden layer is 4. The activation functions of the neurons are the sigmoid function. The input layer is composed of six nodes each representing an attribute.

Using all processors, the construction of the DBs—as described in Figure 1—took nearly 5 hours. It is important to remember that this step is performed only once for a given version of the genomes and their annotations. An mpiBLAST run for one bacterium using all processors took on average 3.5 hours to search the small ORFs of the organism against the subject DB. The other steps do not need parallel computing. The ML model construction and its application for one organism took 1 minute on average, and the filtering procedures took negligible execution times. Therefore, after constructing the DBs, any new search for small ORFs of a target bacterium takes approximately 3.5 hours.

### Showing the suitability of decoy sequences to support the identification of random target hits

As previously described, we have chosen to construct decoy sequences by reversing real ORF amino acid sequences. This is a widely used approach in shotgun proteomics. Most importantly, if the composite target-decoy subject DB is constructed in such a way that in a future search a wrong hit has an equal probability of being target or decoy, then the number of decoy hits can be used as an excellent estimate for the number of wrong target hits, which also results in a very efficient FDR estimate, as already described (49–51, 59). Figure 6 shows that this is the case in our work. This experiment used the BLASTP results of the control datasets. We counted the number of decoy and target hits regarding the queries obtained by shuffling the intergenic ORFs of the respective organisms (see how the control datasets were built in Section ‘Datasets used in the experiments’). As the shuffled sequences are not real ORF sequences, their reported alignments are obviously wrong. Therefore, if the reported hits are approximately half decoys and half targets, it is reasonable to assume that the target-decoy approach to FDR estimation is appropriate. For each control dataset, we performed the following proportion hypothesis test: *H*_0_: *p*_*d*_ = 0.5; *H*_1_: *p*_*d*_≠0.5, where *p*_*d*_ is the proportion of decoy hits. Figure 6 shows the resulting *P*-values in each case. It can be seen that none of them supported the rejection of *H*_0_ for any of the customary significance levels: 0.01, 0.05 and 0.10.

### Illustrating the relabeling process

**Figure 7.**
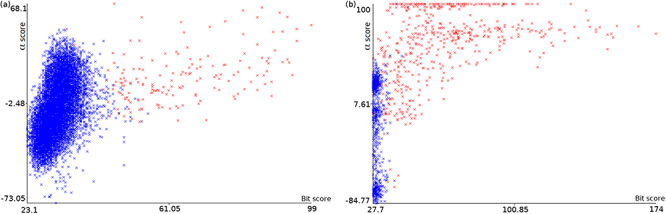
Plots of *α* score vs. bit score to *E. coli* datasets: Ec_vso and Ec_so. Clouds of random alignments are identified by relabeling instances according to the obtained ML model. These plots are the same plots presented in Figure 4a and b, but here all label-1 hits considered random alignments were relabeled to 0. Therefore, the blue data points are those regarded as random, while red data points are considered significant alignments.

We performed experiments to illustrate the process of relabeling random target hits guided by decoy hits. Figure 7 shows the automatic identification of the clouds of random alignments presented previously in Figure 4a and b. After obtaining the ANN+TSA models using the datasets Ec_vso and Ec_so, and applying the respective models to the same datasets to perform the FDR analysis, the instances are relabeled according to their probabilities. Figure 7a and b shows plots for the datasets Ec_vso and Ec_so, respectively, where instances with *P* < 0.5 are labeled 0 (blue) and instances with *P* ≥ 0.5 are labeled 1 (red). Comparing Figure 4a with Figure 7a as well as Figure 4b with Figure 7b, it can be seen in general that red points inside and close to the cloud were relabeled to 0 (blue), while red points clearly out of the cloud were kept with label 1 (still red).

The identification of random alignments depicted in Figure 7 can be expressed also in numerical terms by the resulting confusion matrix. Tables 2a and 2b show the confusion matrices for Ec_vso and Ec_so, respectively, after applying the ANN models on the same data to perform the FDR analysis as described previously.

**Table 2.** Confusion matrices for *E. coli* datasets. (a) Ec_vso, (b) Ec_so.

(a)	Predicted class			(b)	Predicted class		
		0	1			0	1
Actual	0	7396	0	Actual	0	359	0
class	1	8182	177	class	1	440	530

As can be seen, the relabeling process was as expected, i.e. the decoy hits (class 0) were all correctly classified as 0, while a meaningful number of class-1 instances were relocated to class 0, i.e. they were originally class 1 (alignment to a target sequence), but were classified—thus relabeled—as class 0. Those relabeled instances are the ones pertaining to the cloud of random alignments, while the class-1 instances kept as such are those outside the cloud.

Therefore, the original labels in the datasets are interpreted as 0: alignment to a decoy sequence, and 1: alignment to a target sequence, whereas the labels after the ML model application are interpreted as 0: random alignment, and 1: significant alignment.

It is also interesting to note the proportion of random hits among class-1 instances comparing Ec_vso with Ec_so. For Ec_vso, only 2% (177) of class-1 hits could be part of the 0% FDR group. For Ec_so, in turn, the 0% FDR group is composed of 55% (530) of target instances. This difference is also expected because shorter sequences are more prone to random alignments. The same experiment performed to the other datasets led to the same patterns observed in Figure 7. The plots for the other datasets can be viewed in supporting File S3.

### Testing the ANN+TSA approach for hit curation

The goal of the previous experiment was to illustrate the coherence of our ML models. Figure 7 and Table 2 show that the models are presenting the behavior for which they were built, and the use of decoy hits appears to be an appropriate means to show the learning algorithms what is a wrong hit. Nevertheless, to provide a convincing validation of our ANN+TSA approach as a powerful filter to eliminate hits occurred by chance, we applied the resulting models built from hits of the datasets Bs_vso, Bs_so, Ec_vso, Ec_so, Pa_vso, Pa_so, Sa_vso and Sa_so on hits of the control datasets Bs_avso, Bs_aso, Ec_avso, Ec_aso, Pa_avso, Pa_aso, Sa_avso and Sa_aso, respectively. Hence, for each validation test, we used the ANN+TSA algorithms on one dataset and applied the resulting model on another (the control). Testing this way was the solution that we found to computationally evaluate the models generated for each original dataset. Consider dataset Ec_vso, for instance. After applying our described ML strategy on the alignments found by the BLASTP search, it would result in a set of target hits with $\widehat{FDR}=0$. However, as in this case the experiment is being performed on the computational level, it is not possible to affirm whether those hits are in fact positives. Similarly, we cannot confirm whether the ignored target hits are really negatives. On the other hand, if the resulting model built with Ec_vso alignments is applied on alignments found for Ec_avso—for which we know the positive and negative ORFs (annotated and shuffled intergenic ORFs, respectively)—it is possible to recognize true and false predictions. Therefore, for each control dataset, we selected the top-ranked target hits with probability greater than or equal to the discriminant probability found by TSA in the training dataset (threshold for which precision is maximum, i.e. $\widehat{FDR}=0$) as the positive instances (significant hits), and the top-ranked target hits with probability less than the discriminant probability as the negative instances (hits to be disregarded). Next, we identified the annotated small ORFs and the false (shuffled) small ORFs among the queries of all top-ranked target hits. As a result, the positive hits whose queries were found as annotated were labeled true positives, while the positive hits whose queries were found as false ORFs were labeled false positives. Negative hits, in turn, were either labeled as true negatives when their queries were identified as false small ORFs or labeled as false negatives when their queries were identified as annotated small ORFs.

**Table 3.** Testing the ANN+TSA approach. The models were built using hits of four bacteria, and then were applied on the hits of the control datasets of the same organisms. Note that no filtering—such as minimum coverage and identity—is applied here, i.e. this experiment regards only the use of decoy hits and ML algorithms for alignment curation.

Dataset	# top-ranked hits	TP	FP	TN	FN	Ac	Sn	Sp	Pr
Ec_avso	2848	25	0	2819	4	99.86	86.21	100	100
Ec_aso	668	332	4	325	7	98.35	97.94	98.78	98.81
Bs_avso	2609	5	0	2604	0	100	100	100	100
Bs_aso	745	484	0	255	6	99.19	98.78	100	100
Pa_avso	1664	1	0	1663	0	100	100	100	100
Pa_aso	729	205	0	512	12	98.35	94.47	100	100
Sa_avso	3001	7	3	2991	0	99.90	100	99.90	70
Sa_aso	490	320	3	164	3	98.78	99.07	98.20	99.07
Mean						99.30	97.06	99.61	95.99

Table 3 shows the result of this experiment. Note that ANN+TSA alone was enough to result in a very effective curation procedure. The average values for accuracy, sensitivity, specificity and precision were, respectively, 99.30, 97.06, 99.61 and 95.99.

### Comparing OCCAM with MGP using the control datasets

To the best of our knowledge, there are only five previous works that aimed specifically for small ORFs in bacteria, as described in the introduction. The methods COMBREX and SearchDOGs can find only what is described here as absent annotation, i.e. unidentified genes of a species that have annotated homologs in other species. Furthermore, COMBREX uses Glimmer to preselect candidates. However, small genes in prokaryotes are missing precisely because programs such as Glimmer cannot detect them. Also, SearchDOGs analyzes only the set of genomes given as input. It is up to the user to decide which genomes to include. In fact, the authors describe in their manuscript that SearchDOGs is not suitable for large-scale analyses. Therefore, those two methods are clearly less sensitive than our approach. Note that OCCAM seeks for homologs in all bacterial genomes and considers annotated and unannotated homologs. Finally, the work of Goli and Nair as well as the work of Li and Chao do not provide a computational tool. As a result, we made comparisons only with MGP. Note that the comparisons were partial, as shown next, because the MGP authors did not provide a computational tool either.

To perform a first comparison between OCCAM and MGP, we used the same control datasets mentioned in the previous experiment. Even though the MGP computational tool is not available, filtering out hits by *e* > 10^−5^ is trivial. Furthermore, the step of requiring a significant coverage for the query and the subject sequences of a hit is identical in both methods (see Table 1), with the observation that a minimum query/subject coverage of 90% for vsORFs was used also for MGP, which does not consider such category of ORFs. Therefore, we could compare both methods until the point of coverage filtering, i.e. nor the 20% criterion/the clustering procedure were performed for MGP neither the identity (or any other attribute) filtering was performed for OCCAM.

Table 4 shows this first comparison. It can be noted that the methods presented the same results for vsORFs, which are present in a small quantity in the datasets. For sORFs, on the other hand, OCCAM could reach a higher sensitivity, i.e. it could select 44 more hits than MGP (1226 vs 1182).

It can be also noted in Table 4 that both methods could reach maximum specificity and precision. When choosing a very restrictive *e*-value cutoff, such as 10^−5^ (specially in the case of small ORFs), it is not difficult to achieve good specificity. However, sensitivity might be harmed. The challenge is exactly applying some filtering approach that keeps specificity high, i.e. which leads to a result that will not make biologists spend time and money with too many false positives, while not harming sensitivity, i.e. not missing important small ORFs that might be responsible for essential functions in the organism. The OCCAM results shown in Table 4 demonstrate that it is possible to find a solution with better sensitivity without compromising specificity.

In this vein, as ANN+TSA alone (Table 3) already leads to very good sensitivity and specificity (mean of 97.06 and 99.61, respectively), the user has the option of applying this approach and then inspecting the selected alignments carefully instead of using automatic filters. This flexibility that the user has to perform an attentive analysis to maximize sensitivity—or alternatively to apply rigorous filters to maximize specificity—is a key characteristic of the OCCAM computational tool.

**Table 4.** Comparing OCCAM with MGP. The comparison is with the part of the MGP method that we could reproduce here. To filter out random hits, OCCAM uses ANN+TSA, while MGP uses the *e*-value cutoff 10^−5^. As a general evidence of homology, both OCCAM and MGP use minimum query and subject coverage for further filtering. In each line, the OCCAM results are shown first (top) and the MGP results are shown next (bottom).

Dataset	# top-ranked hits	TP	FP	TN	FN	Ac	Sn	Sp	Pr
Ec_avso	2848	23	0	2819	6	99.79	79.31	100	100
		23	0	2819	6	99.79	79.31	100	100
Ec_aso	668	285	0	329	54	91.92	84.07	100	100
		276	0	329	63	90.57	81.42	100	100
Bs_avso	2609	3	0	2604	2	99.92	60	100	100
		3	0	2604	2	99.92	60	100	100
Bs_aso	745	439	0	255	51	93.15	89.59	100	100
		425	0	255	65	91.28	86.73	100	100
Pa_avso	1664	1	0	1663	0	100	100	100	100
		1	0	1663	0	100	100	100	100
Pa_aso	729	189	0	513	27	96.30	87.50	100	100
		184	0	513	32	95.61	85.19	100	100
Sa_avso	3001	5	0	2994	2	99.93	71.43	100	100
		5	0	2994	2	99.93	71.43	100	100
Sa_aso	490	281	0	168	41	91.63	87.27	100	100
		265	0	168	57	88.37	82.30	100	100
Sum/Mean		1226	0	11 345	183	96.58	82.40	100	100
		1182	0	11 345	227	95.68	80.80	100	100

### Comparing OCCAM with MGP in the identification of absent annotations

**Figure 8.**
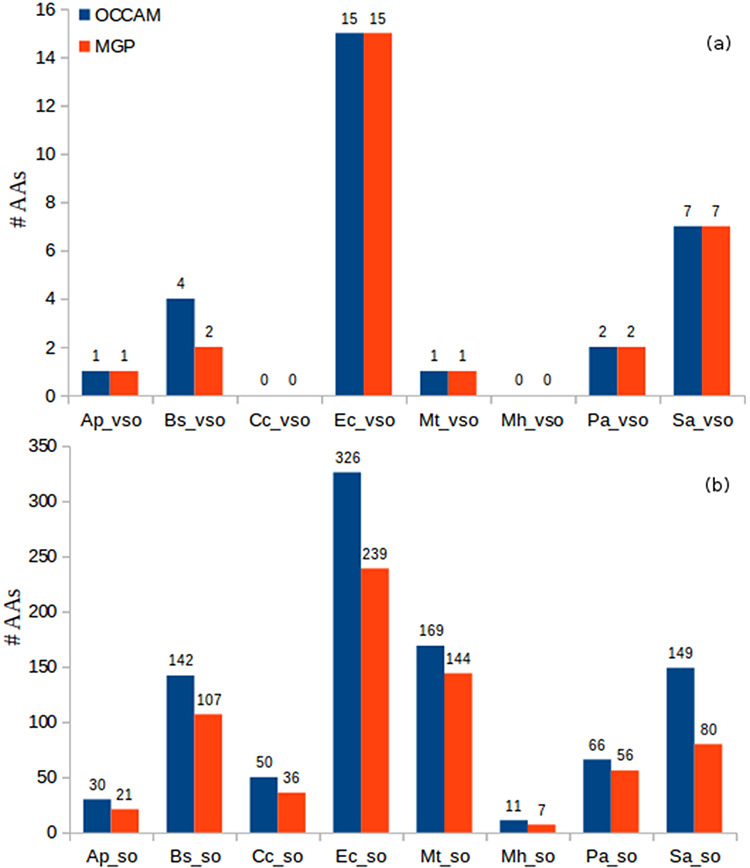
Identification of AAs in OCCAM and MGP for all datasets. (a) vsORF chart. (b) sORF chart. Total number of AAs for OCCAM: 973. Total number of AAs for MGP: 718. The difference of 255 is exactly the number of hits with *e*-value > 10^−5^.

Another experiment to compare OCCAM with MGP was regarding the small ORFs classified as absent annotation. In this case, it is also possible to compare both methods, because to consider a small ORF as AA, it suffices to identify those significant query alignments to already annotated ORFs with high query/subject coverage (80% and 90% for sORFs and vsORFs, respectively). Figure 8 shows the identification of AAs in both methods for all datasets of the eight organisms used in this work. In this case, the OCCAM method was used as designed, i.e. for each dataset containing the resulting query BLASTP alignments of an organism—including alignments to decoy sequences—an ML model was built to relocate random class-1 hits to class 0. The hit details of all small ORFs found as AA by OCCAM for the eight tested bacteria can be found in supporting File S4.

It can be seen in Figure 8 the higher sensitivity that OCCAM could again achieve. OCCAM identified 255 more hits when compared with MGP, meaning a 35.52% increase in sensitivity. It is important to notice that OCCAM could detect the same 718 AAs found by MGP plus another 255 hits that were lost by the latter due to presenting *e*-values greater than 10^−5^. Once again, the differences are particularly notable in the case of sORFs. For vsORFs, the results were nearly the same. The only difference was for *B. subtilis* for which OCCAM could find two extra AAs. The much higher number of sORFs compared with vsORFs also stands out. It is expected as the number of annotated vsORFs is even lower compared to the number of annotated sORFs.

Note that this experiment is a robust validation of our proposed ML method for alignment curation, because the results shown in Figure 8 can be considered true positives. Remember that a query ORF is considered an absent annotation if it aligns to a validated/annotated ORF of a distinct organism.

It is worth noting that even for well-studied bacteria, such as *E. coli*, the number of absent annotations is very expressive. It is occurring probably because small ORFs are not considered in the annotation process in general, nor in homology analysis neither in studies to identify new genes. As we provide a software, and the output includes other scores (e.g. percentage of identity, percentage of gaps, percentage of positives, etc.), the user can apply more restrictive conditions if deemed necessary. For instance, in addition to considering high coverage, one could apply a percentage of identity greater than or equal to 80%. In the case of *E. coli*, it would still lead to expressive numbers: 15 AA vsORFs (all the ones considering only coverage, as shown in Figure 8) and 289 AA sORFs (still a high value). Interestingly, being even more stringent, the number of sORFs—out of the 326 reported in Figure 8—with percentage of identity equal to 100% is 177, which is still a significant value for a widely studied bacterium. For all other bacteria, the observation was the same, i.e. the majority of AA hits presented percentage of identity greater than or equal to 80%, which reinforces the evidence of homology in those hits. Thus, even being more restrictive to consider a hit query as an absent annotation, the number of small ORFs labeled AA in all eight bacteria is still high, mainly considering that most of these bacteria have been studied for decades. This fact corroborates the affirmation that the annotation processes in the case of prokaryotes have been clearly flawed for small genes.

**Table 5.** Results after applying the OCCAM pipeline on all datasets to identify missing genes. It is also shown the found hits that presented *e* > 10^−5^. It can be seen that half the MG hits reported by OCCAM would be missed by MGP.

Dataset	# MGs	# MGs with *e* > 10^−5^
Ap_vso	4	1
Ap_so	0	0
Bs_vso	0	0
Bs_so	0	0
Cc_vso	0	0
Cc_so	0	0
Ec_vso	1	1
Ec_so	0	0
Mt_vso	1	1
Mt_so	0	0
Mh_vso	0	0
Mh_so	0	0
Pa_vso	5	3
Pa_so	3	1
Sa_vso	0	0
Sa_so	0	0
Total	14	7

### Applying the whole OCCAM pipeline to identify MGs

After the comparison shown above involving absent annotations, we proceeded with the identification of MGs for the same eight bacteria. It is important to remember in this case that only hits to unannotated ORFs in a different family is considered. Furthermore, besides the ML techniques to identify significant hits, and the application of the minimum coverage criterion, it is required a high percentage of identity (80% for sORFs and 90% for vsORFs), as described in Table 1. Once again, we emphasize that the previous experiments have shown that the ANN+TSA procedure per se is an effective curation step. But the user has the chance of applying more filters if specificity is a key issue. As a general procedure for identifying MGs, we suggest to apply ANN+TSA, and then the minimum coverage as well as the minimum identity filters.

Table 5 shows the results after applying the full suggested pipeline to identify MGs on the eight datasets. The hit details of all small ORFs found as MG by OCCAM in the tested bacteria can be found in supporting File S5. Because the conditions to identify MGs are more rigorous—especially the different-family criterion—we could obtain very few MG small ORFs. This time, it was not feasible to compare OCCAM with MGP, because it is not possible to run the whole MGP pipeline without a software. Nevertheless, Table 5 shows the hits with *e* > 10^−5^—totaling 7—that would be obviously missed by MGP, i.e. certainly, 50% of MGs reported by OCCAM would not be detected by MGP.

*In vitro* validation of small ORFs is still challenging (40). However, in order to provide some evidence that the selected hits presented in Table 5 correspond in fact to MGs, we analyzed publicly available RNA-Seq data of the organisms covered in this work to at least verify whether those found small ORFs are expressed by the respective bacteria. The NCBI Sequence Read Archive (SRA) repository (64) was chosen to search for transcriptomic data of experiments involving the four organisms for which OCCAM reported MGs (Table 5). No experiments could be found for the serovar of *A. pleuropneumoniae* considered in this work. For the other three bacteria, on the other hand, the encountered experiments confirmed that all small ORFs reported for the considered strains of *E. coli*, *M. tuberculosis* and *P. aeruginosa* are expressed. It was verified by performing a BLAST search with default parameters using the online tool SRA BLAST (64). For each organism, the search procedure used as queries the DNA sequences of the ORFs reported in Table 5, while the subject sequences were the reads produced in the experiments that we could find in SRA for the given organism. Note that in SRA BLAST, the subject sequences can be defined by simply informing the accession number of the experiments. The accession numbers of the RNA-Seq studies/experiments found for each organism are listed in the supporting File S6. For each small ORF of the three analyzed organisms, the BLAST results showed a clear stack of reads aligned to the ORF sequence, covering 100% of this sequence.

A similar experiment was next performed using also the SRA repository and the SRA BLAST tool, but this time with ribosome profiling data. Unfortunately, this type of data is still scarce. Nevertheless, we could find two studies for *P. aeruginosa* and *E. coli*. The accession numbers of those studies and respective experiments can be found in the supporting File S7. The procedure was the same as reported above for RNA-Seq data, i.e. for each bacterium, we used as queries the DNA sequences of the ORFs identified as MGs, and as subject the reads pertaining to the experiments of the study found in SRA. As already seen, Table 5 shows that OCCAM could find one vsORF for *E. coli*. For *P. aeruginosa*, in turn, our method reported five vsORFs and three sORFs. The SRA BLAST searches found high-quality alignments—with 100% identity—for the vsORF of *E. coli*, for three out of the five vsORFs of *P. aeruginosa*, and for all three sORFs of this same organism. Therefore, seven out of nine small ORFs detected by OCCAM as MGs in that two bacteria were supported by the found data as translated ORFs.

The seven putative novel small ORFs identified by OCCAM, for which we could find validating RNA-Seq and ribosome profiling data, are detailed in Table 6. Note in the table that the three worst alignments (lines 1, 3 and 4), i.e. the ones with the lowest bit scores and highest *e*-values, presented *β* > 1, which means that those ORFs aligned to other intergenic ORFs that corroborate the MG classification. It shows how a multivariate analysis, as proposed here, is important. If the *e*-value is the only attribute to be checked, for instance, there is a risk of missing important ORFs. In the case of those three worst alignments, the *α* score values are also low. Therefore, the *β* values together with the values of identity, query coverage and subject coverage resulted in a high probability produced by the ML model, avoiding a false negative situation. Those three cases also indicate the importance of using the *β* score for assessing the resulting alignments. The value *β* = 106 of the ORF identified in Table 6 as Pa vsORF 3 is noteworthy, and provides a strong evidence of the correctness of the MG classification.

All performed searches using RNA-Seq and ribosome profiling data reported above can be easily reproduced by using the sequences of the identified ORFs (see supporting File 5) and the accession numbers of the studies/experiments in SRA (see supporting Files 6 and 7).

**Table 6.** Seven putative novel small ORFs identified by OCCAM. Further details, including the respective nucleotide and amino acid sequences, can be found in supporting File 5. The following abbreviations were used below: Ec (*E. coli*), Pa (*P. aeruginosa*), aa (amino acids), s_cod (start codon), st (strand), coor (coordinates), bs (bit score), *e* (*e*-value), id (identity), qc (query coverage), sc (subject coverage), *P* (probability).

	# aa	s_cod	st	coor	bs	*e*	% id	qc	sc	*α*	***β***	*P*
Ec vsORF	13	ATG	−	2 247 517	46.0	0.001	100	100	100	32.87	5	0.88
				2 247 558								
Pa vsORF 1	25	ATG	−	2 232 344	80.4	9E-14	100	100	100	52.95	1	0.99
				2 232 421								
Pa vsORF 2	14	GTG	+	2 232 413	48.1	3E-4	100	100	100	40	4	0.93
				2 232 457								
Pa vsORF 3	13	TTG	+	2 079 443	45.2	0.002	100	100	100	53.54	106	0.92
				2 079 484								
Pa sORF 1	55	ATG	+	2 232 204	84.0	2E-14	98.18	100	100	98.18	3	0.99
				2 232 371								
Pa sORF 2	43	TTG	+	2 234 617	62.8	3E-8	97.67	100	100	97.67	1	0.99
				2 234 748								
Pa sORF 3	43	ATG	−	2 234 553	52.8	3E-5	97.67	100	100	97.67	1	0.99
				2 234 684								

Unfortunately, other publicly available data of *in vitro* experiments could not be found to support further validations. Even in recent days, most studies to uncover coding genes aim for proteins composed of 100 or more residues, which means that scarce data regarding small proteins are available. We performed a deep search for small proteins throughout several public DBs of mass spectrometry, for instance, but negligible data could be found. As stated by Hemm et al. (42), even with the advancements in the sensitivity of mass spectrometers, the identification of small proteins remains a complex issue. Comprehensive searches using online tools for detecting motifs were also performed without success. The lack of recognized patterns in small ORFs are a major issue in their elucidation, as described in detail by Warren and colleagues (22). The authors show that small ORFs present a variable GC content and can rarely have an RBS assigned. Note that most of the bacteria used in our experiments are broadly covered in several studies worldwide. However, even for such organisms, data regarding small proteins are insufficient. For this reason, we presented a multifaceted series of experiments—including computational validations as well as transcription and translation analyses—that taken in conjunction point to a useful computational method for promoting advances in the elucidation of small ORFs in bacteria.

## Conclusions

If compared to studies on small ORF detection in eukaryotes, very few comprehensive works proposing computational methods with a focus on small ORFs in bacteria can be found in the current literature.

COMBREX and SearchDOGs are limited to the cases described here as absent annotations, i.e. they cannot reveal novel genes. Therefore, they are clearly less sensitive than our approach. Even if we consider only AAs, it is important to remember that SearchDOGs analyzes only the genomes that are given as input by the user, whereas OCCAM searches all bacterial genomes available. The number of candidate genes predicted by SearchDOGs for *E. coli* K-12, for instance, was 270. As reported in our results, 341 AAs could be identified by OCCAM for that same organism, which means a 26.3% increase. Note that the putative genes identified by SearchDOGs include also long genes. Therefore, if only short genes are taken into account, the OCCAM superiority is even higher.

Goli and Nair as well as Li and Chao present interesting approaches, but do not provide a computational tool. Also, the work of Warren and colleagues, termed here MGP, is an in-depth study, but the authors did not deliver a software either.

In this work, we propose an amelioration of MGP. We present the method OCCAM, which is fundamentally comprised of an ML procedure associated with the use of decoy sequences included in BLAST searches to identify small ORFs in bacterial genomes. One important improvement of our work is the effectiveness of the proposed ML algorithms to filter out spurious alignments. For this task, MGP—and so many other procedures based on alignments—uses a fixed threshold for *e*-value, namely, all alignments with *e* > 10^−5^ are eliminated from further analyses. This is a dangerous criterion in terms of sensitivity, especially in the case of small ORFs for which *e*-values are higher when compared with usually studied ORFs. In our case, instead of using a single attribute to judge an alignment, the ANN+TSA procedure applies six attributes, where one of them—the *β* score—is proposed here. Furthermore, the alignments to decoy sequences are used by the ML algorithms to both learn what is a random alignment and control the FDR, in addition to attain a probability value to each alignment. This value expresses the conjunction of six attributes and serves as an important quality parameter of a given alignment. Experiments with control datasets demonstrated that the ANN+TSA alone can reach average sensitivity and specificity of 97.06% and 99.61%, respectively. Besides providing a solution for the particular problem posed here, we conjecture that the proposed method for curating alignments might be tested as a replacement of simplistic cutoff-value approaches used in alignment-oriented solutions for other problems in biology.

Another important contribution of this work is the implementation of a set of programs. Our software provides great flexibility to the user. The effectiveness of ANN+TSA and the possibility of using different filters (parameter setting) to curate alignments enabled the elimination of the clustering phase of MGP. It means that we could implement programs that allow the analysis of specific genomes given as input. Note that MGP performed BLASTP searches for ORFs of all prokaryotic genomes. It demands a very powerful and expensive computational platform, particularly when very small ORFs are included, as in our case. In OCCAM, because the user runs our software only for genomes of interest, affordable computational platforms can handle the execution of all programs needed. Giving the user the power of choosing several parameters is a key advantage. The filtering by FDR, for instance, is an important adjustment that the user can make. Here, we have chosen to work with FDR=0, and this is the default value of our programs. However, other FDR values can be set. For example, a 1% FDR is considered an interesting choice, because it still means a low rate of false positives, but might mean a higher sensitivity (49, 54). Even the ORF size is parameterized. We focused only on small ORFs and used specific size ranges, as already described, but other range values might be used, i.e. longer or shorter ORFs can be included in the genome screening procedure.

Notably, even for a well-studied bacterium such as *E. coli*, a potential vsORF could be detected, not to mention the many absent annotations found by OCCAM. It demonstrates that small ORFs have been in fact systematically neglected.

It is clear that a combined approach that includes computational tools and *in vitro* validation is essential for successfully identifying small ORFs (40). *In vitro* analysis alone is too expensive and time-consuming, while using only computational tools is not enough for a definitive confirmation. The importance of computational procedures is to minimize the number of spurious identifications so that the *in vitro* experiments are also minimized. In this sense, accurate algorithms are fundamental, particularly in the case of methods that work specifically on small ORFs, as proposed here, for which current solutions still leave much room for improvement. We are confident that the presented study is an important step in the direction of building advanced computational tools that can strongly support the bench work in the identification of small ORFs in bacterial genomes.

## Supplementary Material

baaa067_Supp

## Supporting information

The following files are available as supporting information.

- Supporting File S1: Accession numbers of the genomes of the eight bacteria used in this work.
- Supporting File S2: Start codon distribution in the 16 ORF datasets used in our experiments.
- Supporting File S3: Plots of *α* score vs. bit score to the other seven datasets.
- Supporting File S4: Search hit details of all small ORFs found as Absent Annotation by OCCAM in the eight tested bacteria.
- Supporting File S5: Search hit details of all small ORFs found as Missing Genes by OCCAM in the eight tested bacteria.
- Supporting File S6: Accession numbers of studies and respective experiments in SRA involving RNA-Seq data for the bacteria for which OCCAM identified MGs.
- Supporting File S7: Accession numbers of studies and respective experiments in SRA involving ribosome profiling data for the bacteria for which OCCAM identified MGs.

For the sake of reproducibility, the programs, the results in all phases of our experiments, and all genomes downloaded from RefSeq are also available online at http://www.labinfo.lncc.br/occam.
