# Supplementary material for: OCCAM: prediction of small ORFs in bacterial genomes by means of a target-decoy database approach and machine learning techniques
Source: Database (Oxford). 2020 Nov 18;2020:baaa067. doi: 10.1093/database/baaa067 (PMC7673341; doi:10.1093/database/baaa067)
Supplement: baaa067_Supp [file baaa067_supp.zip › Supporting_File_S1.pdf]

Identification of the genomes of the eight bacteria used in this work

Organism	Genome identification
Escherichia coli K12 substr MG	NC_000913
Mycobacterium tuberculosis H37Rv uid57777	NC_000962
Bacillus subtilis 168	NC_000964
Pseudomonas aeruginosa PAO1	NC_002516
Caulobacter crescentus CB15 uid57891	NC_002696
Staphylococcus aureus MRSA	NC_002952
Mycoplasma hyopneumoniae 7448 uid58039	NC_007332
Actinobacillus pleuropneumoniae serovar 5b L20 uid58789	NC_009053
