# Supplementary material for: OCCAM: prediction of small ORFs in bacterial genomes by means of a target-decoy database approach and machine learning techniques
Source: Database (Oxford). 2020 Nov 18;2020:baaa067. doi: 10.1093/database/baaa067 (PMC7673341; doi:10.1093/database/baaa067)
Supplement: baaa067_Supp [file baaa067_supp.zip › Supporting_File_S3.pdf]

Plots of alpha score vs. bit score to the other seven datasets

The data points represent the query alignments found by BLAST. For each bacterium, (a) and (b) show the alignments of vsORFs and sORFs, respectively, before applying the ANN+TSA approach, while in (c) and (d) the same data is shown after relabeling the target hits that were considered random by ANN+TSA. In (a) and (b), red data points are target hits, whereas blue data points are decoy hits. In (c) and (d), red data points are those target hits considered as significant by OCCAM (label was kept), while blue data points are decoy hits plus target hits relabeled, i.e., considered random.

In all plots below, axis *y* is alpha score while axis *x* is bit score.

Actinobacillus pleuropneumoniae serovar 5b L20 uid58789

(a)

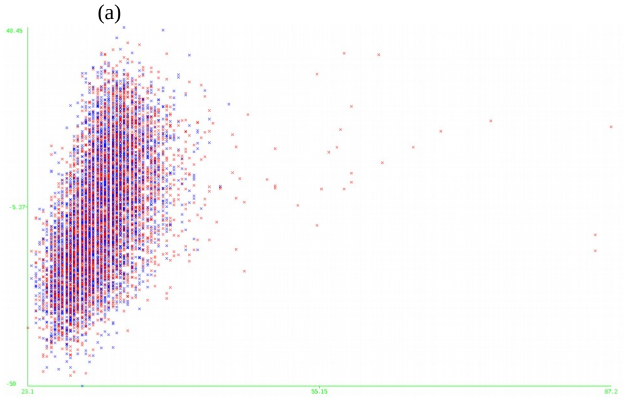

(b)

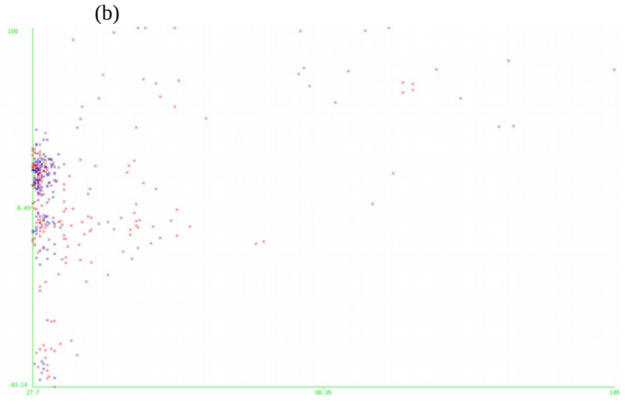

(c)

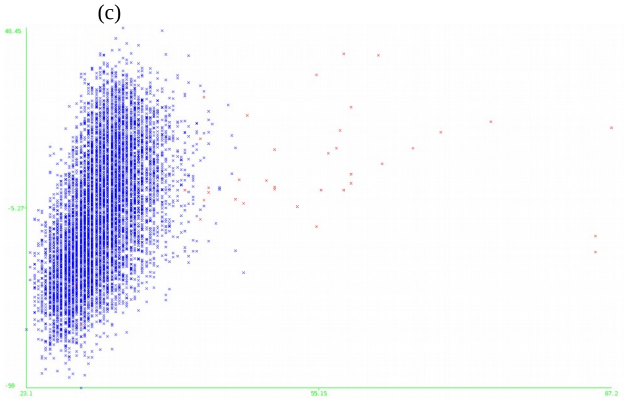

(d)

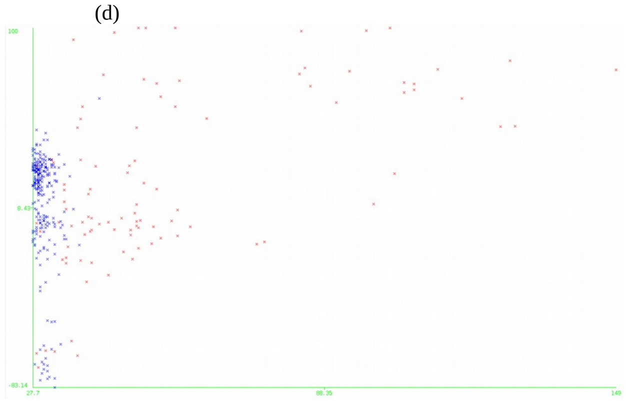

Bacillus subtilis 168

(a)

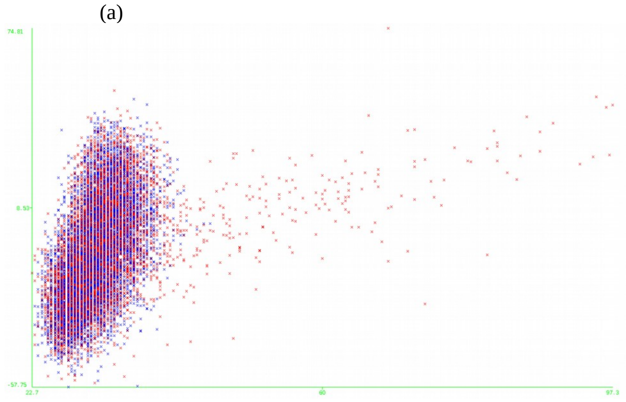

(b)

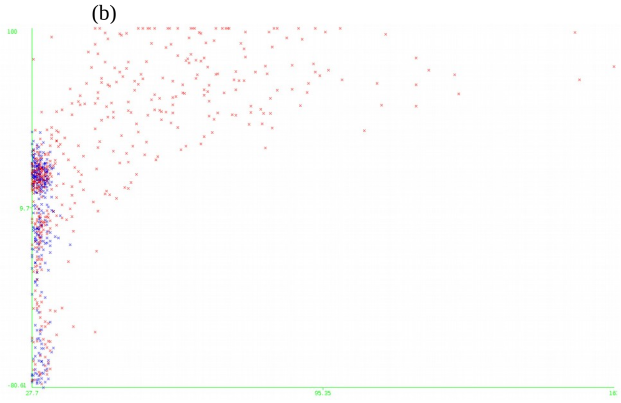

(c)

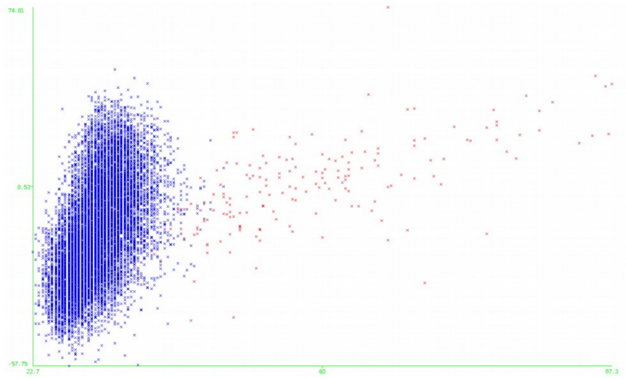

(d)

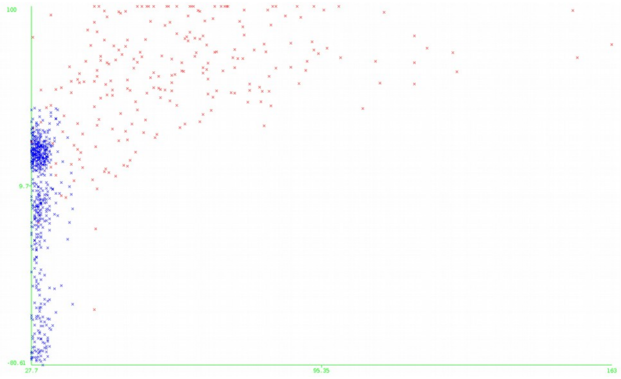

Caulobacter crescentus CB15 uid57891

(a)

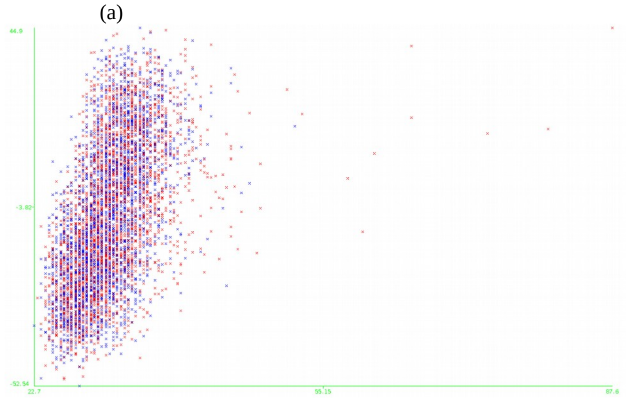

(b)

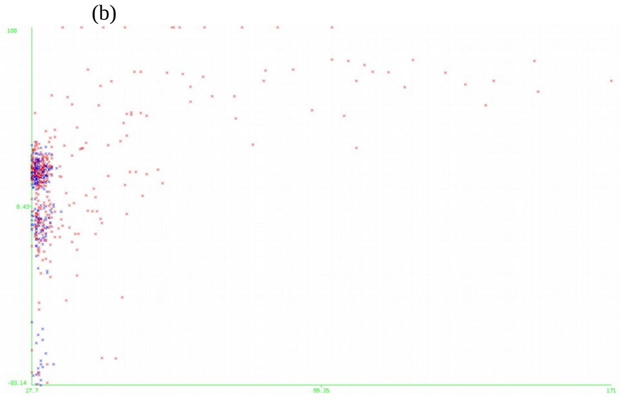

(c)

(d)

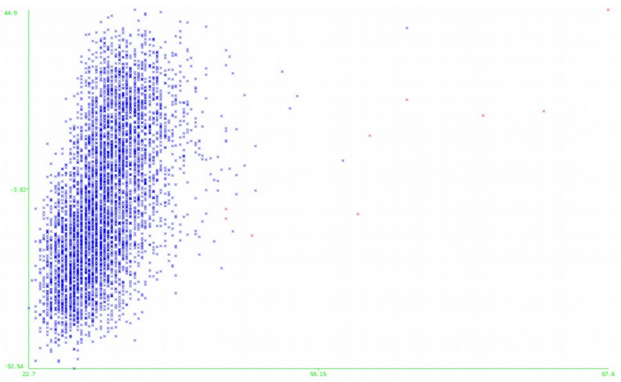

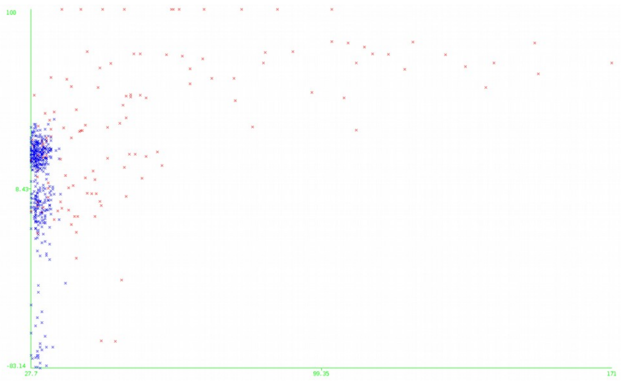

Mycoplasma hyopneumoniae 7448 uid58039

(a)

(b)

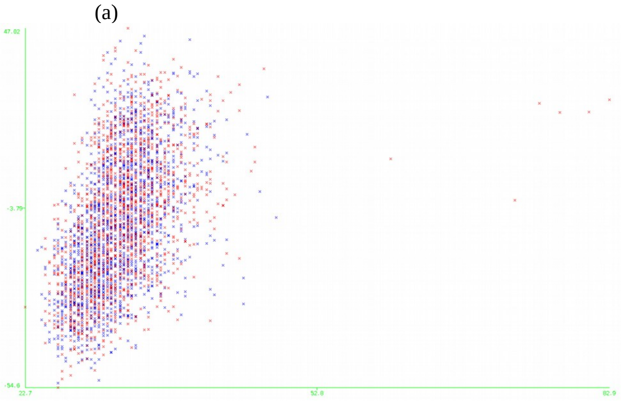

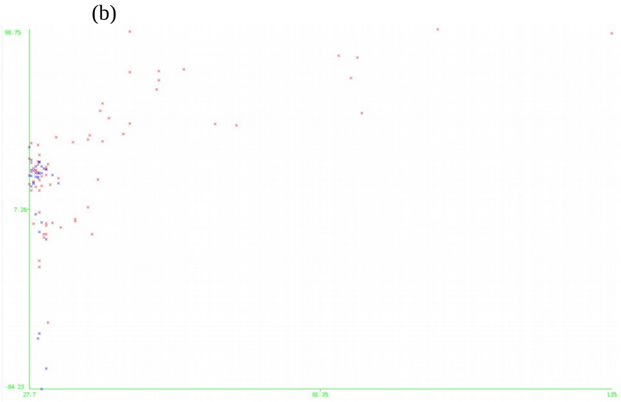

(c)

(d)

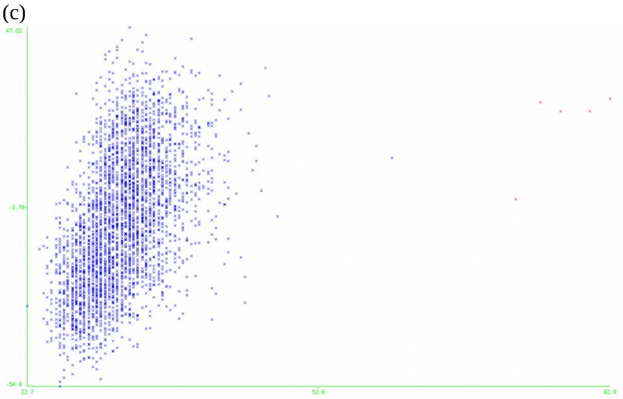

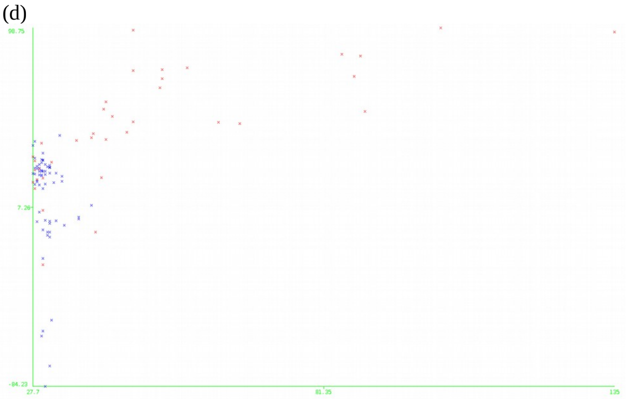

Mycoplasma hyopneumoniae 7448 uid58039

(a)

(b)

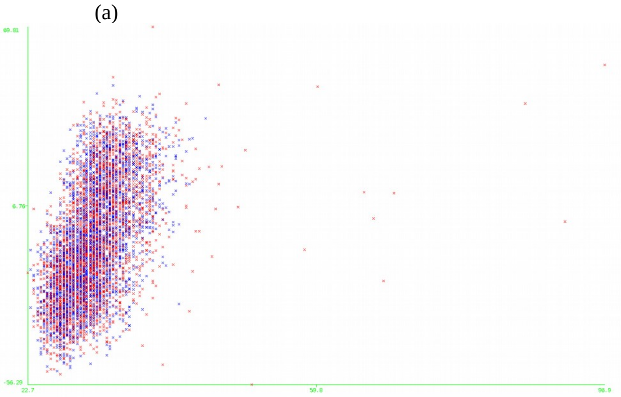

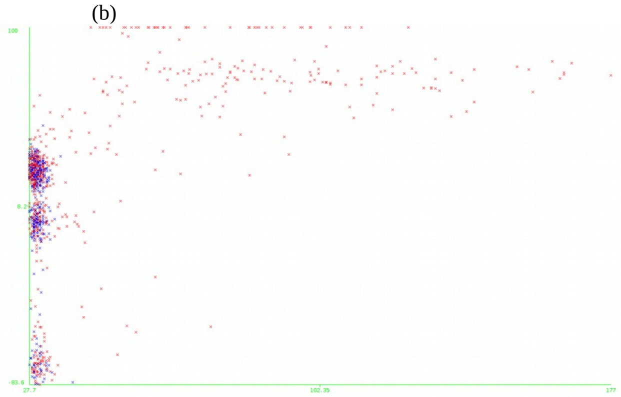

(c)

(d)

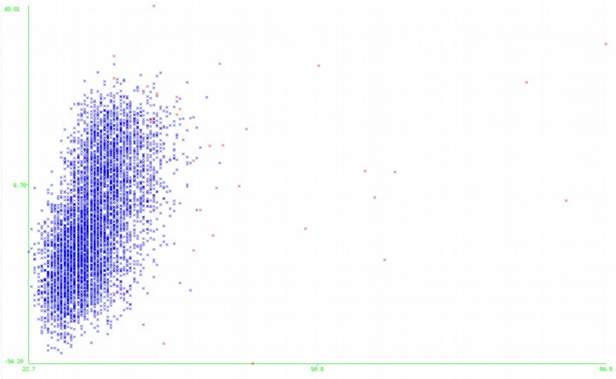

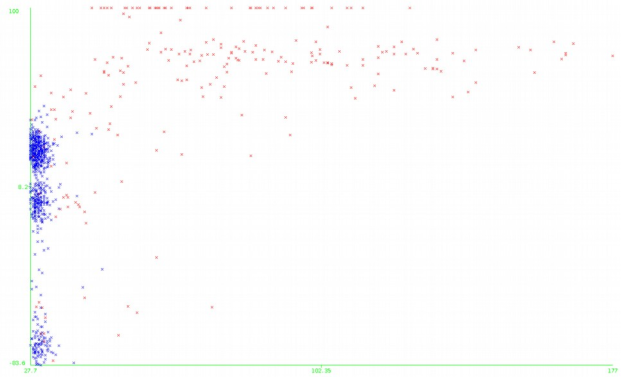

Pseudomonas aeruginosa PAO1

(a) (b)

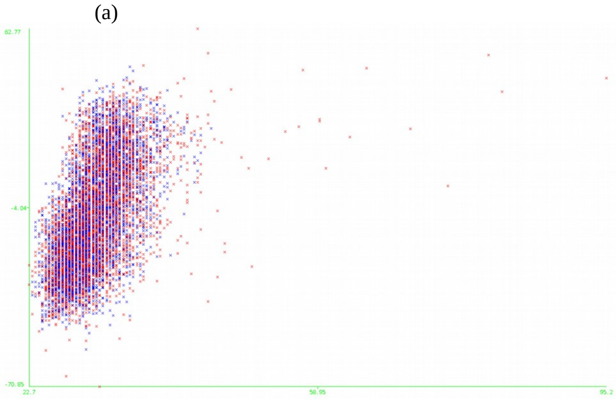

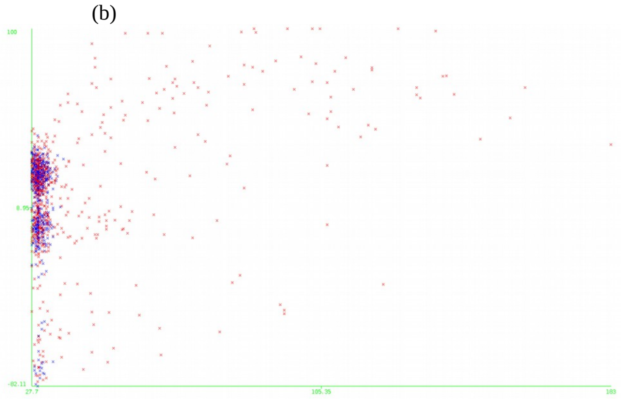

(c) (d)

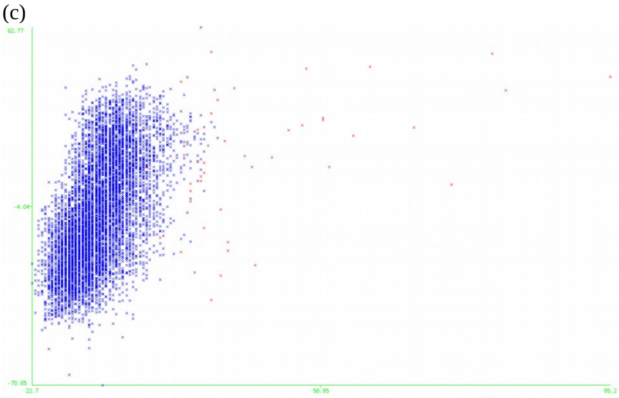

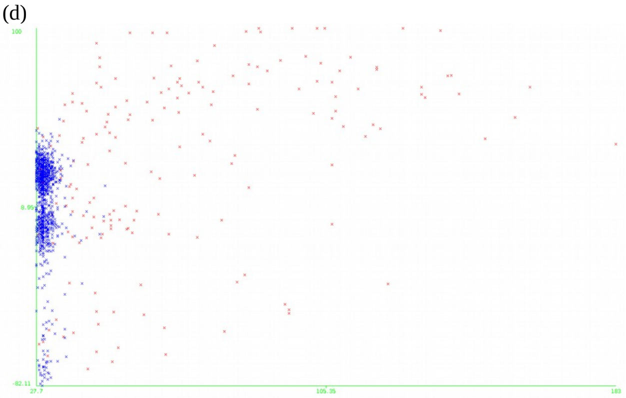

Staphylococcus aureus MRSA

(a) (b)

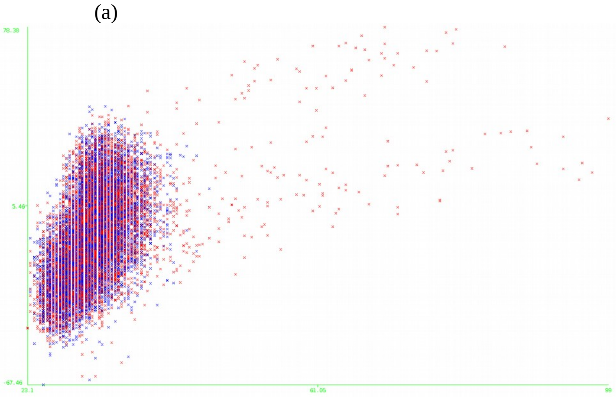

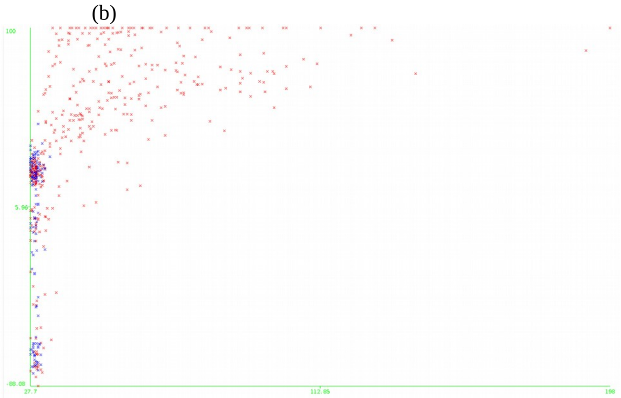

(c) (d)

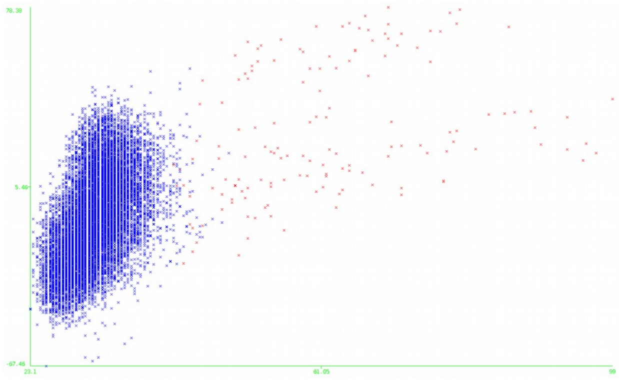
78.38
5.46
-67.46
23.1
61.05
99

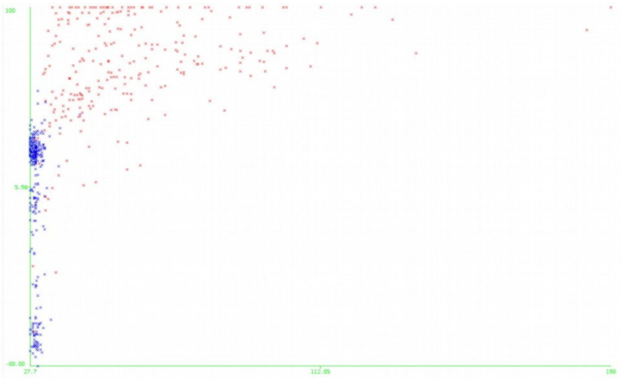
100
5.96
-88.08
27.7
112.85
198
