# Supplementary material for: OCCAM: prediction of small ORFs in bacterial genomes by means of a target-decoy database approach and machine learning techniques
Source: Database (Oxford). 2020 Nov 18;2020:baaa067. doi: 10.1093/database/baaa067 (PMC7673341; doi:10.1093/database/baaa067)
Supplement: baaa067_Supp [file baaa067_supp.zip › Supporting_File_S4.pdf]

Search hit details of all small ORFs found as Absent Annotation by OCCAM in the eight tested bacteria

The head of each search hit indicates class (AA in this case), query genome ID, query strand (positive or negative), query coordinates indicating the ORF start and end , subject (or hit) genome ID, subject strand, and subject coordinates.

Then, it is shown that the search hit was the best one found (rank=1, i.e., top-ranked), followed by scores generated by BLAST and OCCAM, including the probability generated by the ML algorithms. Below the head, it is shown the ORF DNA sequence.

- ***Actinobacillus pleuropneumoniae*** **serovar 5b L20 uid58789**

vsORFs

>AA QryID: NC_009053_(+)_364590_364640 HitID: NC_018690_(-)_1189409_1189459
rank=1 bitscore=54.9 evalue=2.0E-6 perc_ident=100.0 perc_conserv=100.0 perc_gaps=0.0
query_cvg=100.0 subj_cvg=100.0 alpha=34.38 beta=1 prob=0.990274623599495
ATGGCGGAAGAACCGATTCGTTTGACGCAATACAGTCACGGTGCCGGTTGA

sORFs

>AA QryID: NC_009053_(-)_1271425_1271694 HitID: NC_010278_(-)_1233454_1233723
rank=1 bitscore=149.0 evalue=4.0E-34 perc_ident=100.0 perc_conserv=100.0 perc_gaps=0.0
query_cvg=100.0 subj_cvg=100.0 alpha=78.67 beta=2 prob=0.9998978053064698
ATGGCATATTACACTACAGATGTATCTAGTCATTTTCAGTATGATGAGCTTAATAATGAT
AGAAAGGTACTACATACCATTCATTTTTTTATAGATGATCGGCTATCTGACCATCAAAGA
CATATCGATAAGTGGCAGAATCATATTATTATTAACAGGAGTAAGTACCCTAAATTTATT
AGTTCTATAAGAGTTAATATTTCGTTTTATGACGTCATTATAGCTGATAATGTTAGTGGA
TTAACGCTTGAACGCCATGTTTTGATGTAA
>AA QryID: NC_009053_(+)_606261_606482 HitID: NC_010278_(+)_569511_569732
rank=1 bitscore=127.0 evalue=2.0E-27 perc_ident=100.0 perc_conserv=100.0 perc_gaps=0.0
query_cvg=100.0 subj_cvg=100.0 alpha=83.14 beta=3 prob=0.9998456047621709
ATGACAGATCTTCTTAAAATTGGTGATACCGAATTTCACTCCCGTCTTTTTACCGGTACG
GGCAAATTTTCAAATCCTAAAGTGATGCAACAAAGTATTGTTACATCAGGTTCCCAGCTT
GCAACAATGGCGATGAAACGGGTGGATTTAGCACAGGAACAAGACGATATTTTAACGCCG
TTAATTCAGAGTAAAACTGTTACCGAATACTTCGGGGGCTAA
>AA QryID: NC_009053_(+)_1953792_1954010 HitID: NC_010278_(+)_1915660_1915878
rank=1 bitscore=112.0 evalue=5.0E-23 perc_ident=100.0 perc_conserv=100.0 perc_gaps=0.0
query_cvg=100.0 subj_cvg=100.0 alpha=78.87 beta=2 prob=0.9998381437853132
ATGGAGGAAAATGAGACTTTTTATATTACGTTTGATGAGCGTTTACTAGACTATTTGAAA
AATAGCGATGCTCAAACACATTTTGTCAGTTTTGTAGAAGTAGAGAATGAAGTTGATATT
CCTACGGCATTGTCCATGAAAATTAAGAAATCTGATCGCCATCAAATTTCTTCAAAGCCA
TTTAAAATTAAATCTTATTCTGAAGAAGTAAATACATAG
>AA QryID: NC_009053_(+)_1412416_1412613 HitID: NC_018690_(-)_138668_138865
rank=1 bitscore=105.0 evalue=6.0E-21 perc_ident=100.0 perc_conserv=100.0 perc_gaps=0.0
query_cvg=100.0 subj_cvg=100.0 alpha=67.0 beta=20 prob=0.9998381160497489
ATGTCTGGTAAATCGTCAGATACATCACAAAGCTGGTTCAAAAAAATGCTGGCGAAATAT
GATAAATTTTGTGAAGAACTCGGTGTAAACCAAGGTGCTTGCCGTGGCTGCGTGCCGGTG
GTGAAATTCGATCCGGAAAAAGAACCGAAATCAACAAAATCACAAGATAAAAACGCGACA
GAACAGCACTCAAGCTAG
>AA QryID: NC_009053_(-)_1103383_1103547 HitID: NC_010278_(-)_1069984_1070148
rank=1 bitscore=105.0 evalue=8.0E-21 perc_ident=100.0 perc_conserv=100.0 perc_gaps=0.0
query_cvg=100.0 subj_cvg=100.0 alpha=72.11 beta=1 prob=0.999836883418822
ATGTATGGGATTCATAAGGATACAGGATTACCTTTAACAGACAATCTTGATAGGATTTGT
ACTAAAATTTCTCTTGAAGGATTTGAAATTGGAGATAATGGCTTTCCAATAATTGTTGGA
ATAGAAAATCAGAAAAATTGGATGAACGTTATGGATCTTGTTTAA
>AA QryID: NC_009053_(-)_1338846_1339076 HitID: NC_010278_(-)_1304295_1304525
rank=1 bitscore=117.0 evalue=2.0E-24 perc_ident=94.74 perc_conserv=96.05 perc_gaps=0.0
query_cvg=100.0 subj_cvg=100.0 alpha=63.85 beta=7 prob=0.999836156208983
ATGTTTAATATATTTAAGAAACCTTTTAATAGAGAAAGCTTAGATTCTTGGTGTAAGATT

TTGGATGATATTAGTAAAATTGAGCTTTTAGCAATTCCTGTTGTACTATATAGCCTGAAT
GGTTTGTTTTATAAATTATTTAATAGCACTGTGCTTTTATTTTCAATGTATATATGTTTA
TTTATGGCAGATTTTATACGTAAAAATAAAGAGAATTTGATGGAGGAATAA
>AA QryID: NC_009053_(-)_2032829_2033032 HitID: NC_010278_(-)_1994432_1994635
rank=1 bitscore=102.0 evalue=4.0E-20 perc_ident=100.0 perc_conserv=100.0 perc_gaps=0.0
query_cvg=100.0 subj_cvg=100.0 alpha=100.0 beta=2 prob=0.9998355675176063
ATGTCAATCTCATATAGGAAGGAAATTAAAGCCAATTCGTATTCGTTATTAGATCAAGTA
TTTTTAAATGAGCGTTCAGAGAAACCGAATGAGAGCAGTTATTTTCCTTATAAACATCAT
AATGTTAGAAGTACCTATACAGGTATAAAACGTTATATGAGTTATGTTTTTATGTATGGA
GAAATACCCCGAATTAAATATTGA
>AA QryID: NC_009053_(-)_1799951_1800082 HitID: NC_010278_(-)_1760677_1760808
rank=1 bitscore=85.5 evalue=4.0E-15 perc_ident=100.0 perc_conserv=100.0 perc_gaps=0.0
query_cvg=100.0 subj_cvg=100.0 alpha=70.23 beta=2 prob=0.9998350667460614
ATGCGTAAACCCCACATGGCTCGTGTGGGGGACAACGTTTGTGCTTGTGTATTAGATTTG
TCGATAATTAAAACCTTTGCCTCTGGCTTTGGTTGGTACTCTCTTTCAAGCGTATTAGTT
TATGATGCCTGA
>AA QryID: NC_009053_(+)_2166023_2166220 HitID: NC_010278_(+)_2133993_2134190
rank=1 bitscore=97.1 evalue=2.0E-18 perc_ident=98.46 perc_conserv=98.46 perc_gaps=0.0
query_cvg=100.0 subj_cvg=100.0 alpha=98.46 beta=9 prob=0.9998350500136746
ATGAAAAAGGCATTTTTATTATTGCCGTTTATGGTAAGCCTGACCGCTTGCGGAGCAAGT
GAGAACGCACCGAAAGACGATGCGGAATTGGCGCCGAGGATTATGCAACCCGTTTCCGGA
TCAGGCGCATCGACAGGAAGCTACGGTTGGTCGTCAGACGTTGAGTCCGCACCGATGCCG
GCTTCAATGACTAAATAA
>AA QryID: NC_009053_(-)_563208_563474 HitID: NC_018690_(-)_571159_571380
rank=1 bitscore=128.0 evalue=1.0E-27 perc_ident=86.3 perc_conserv=91.78 perc_gaps=1.37
query_cvg=81.82 subj_cvg=100.0 alpha=49.91 beta=2 prob=0.9998349843651906
TTGGGGCTTTTTTTTAACAAAATTTATCCCCCTCCCGAGGACAATACTATGAGTAACTTA
TCAATCTCCCTCCTAGCTATCGGCACTGTAATGCTACTCGGAGGGCAAATCGCAACAACC
AAAGATTTAGACGGTAAAGAATTAGTAGATTCAATTCATTGCCGACAGAAAGGCGGCACG
ATGGAATGGGTACATAGTCACGCATTTGTAAAAACCCTCAAATGCCAATACCCACAAGCA
ACGCCGATCGAATTTGTAAATAACTAA
>AA QryID: NC_009053_(-)_214422_214559 HitID: NC_010939_(-)_221710_221847
rank=1 bitscore=84.3 evalue=2.0E-14 perc_ident=100.0 perc_conserv=100.0 perc_gaps=0.0
query_cvg=100.0 subj_cvg=100.0 alpha=79.44 beta=1 prob=0.999834929111767
ATGGTTTCTTGCGATAATCATACCCAATTATTTTGGGCAATTTGTGGTATTTCTTATACT
AAACATAATCGAAAAAACATTTGCAAAATTTTTCGTAAAACAGACCGCTTGCCGTTGCTA
AAAACACGAAGCAATTGA
>AA QryID: NC_009053_(+)_2128804_2128968 HitID: NC_010278_(+)_2095483_2095647
rank=1 bitscore=83.6 evalue=2.0E-14 perc_ident=98.15 perc_conserv=98.15 perc_gaps=0.0
query_cvg=100.0 subj_cvg=100.0 alpha=98.15 beta=1 prob=0.9998346887589171
ATGAGATTGTTTCACACTTTCCGTCTTAGAACAACCTCTAAAGCCAATTTTGTAGATAAC
TCTGTGGATAAGTTGTCAAATAGCTCGGTTTATAATGAGTATAACCGAGATAACCCCTTG
TTTTTATTTATAAGAGCGATGATCTTTATTTATGCGCAAAAATGA
>AA QryID: NC_009053_(-)_562929_563198 HitID: NC_018690_(-)_570879_571148
rank=1 bitscore=125.0 evalue=6.0E-27 perc_ident=75.0 perc_conserv=81.82 perc_gaps=0.0
query_cvg=98.88 subj_cvg=98.88 alpha=49.63 beta=1 prob=0.9998345816925083
ATGGCTATTGATGACTGGAATTTATATCCGCAGAAACGACCGATTAAGAGTGGCAACTAT
CTTGTAGTTGTTACCGTTAAAAATGGAGCGGCTGGCATTAGCCGCTTAGTCACACTAAGT
AACTCTGATGCTATGCGCCACGAATTTGAATTTGACGCACGCATGAAAGAACGTGGAGGT
ACTGAACACGTTTACGCATGGGTACATTACAAAGAACCACCTCCGCCACCGGGATTAACC
GCACCGAAAGGAAGTAAAAATGAACGCTAA
>AA QryID: NC_009053_(+)_899138_899350 HitID: NC_010939_(+)_940057_940269
rank=1 bitscore=90.9 evalue=2.0E-16 perc_ident=81.43 perc_conserv=81.43 perc_gaps=0.0
query_cvg=100.0 subj_cvg=100.0 alpha=61.84 beta=11 prob=0.99983454383303
ATGAGTATTATTGAATTTCTTAGCTATGGCTCAATCCTGATTCTCGCTCTTTCCTTTGTC
ATTAAAGATATGCCTAACCTTCGTATCTCTAATATTTGTTGGGGATTAGGTTTTATTTTT
TACAGTCTTAACTCAGCAATGTATATTGTTGCCGCTTTGAATTTATTGATTGTCGTTTTA
AACGGCTATTTTCTGATTAAAGAACGCCCGTAA
>AA QryID: NC_009053_(+)_546415_546708 HitID: NC_010278_(+)_553348_553641
rank=1 bitscore=103.0 evalue=3.0E-20 perc_ident=53.61 perc_conserv=70.1 perc_gaps=0.0
query_cvg=100.0 subj_cvg=100.0 alpha=25.77 beta=19 prob=0.9998344001435253
ATGCAGCATAAAATTATCTATTCAAGTAACGCTCAAAAAGATATCGAAGATATTTTTGTG

AATATTACTGAATTCACAAGTGTTTTTAGTGCTATCAATGTATTAAATGATGTAAAAGCT
TCGATTGAGTTACTCGGGTTTATGCCTCAAATGGGTGTCGAAGGGTATATAAAAGGTACA
AGGGAGATTTATTCGAGAAAATACAGAGTTGTATATGAAATAGATGATGAGAAGTCAGAA
ATTATCATCCTTACCATTTTACATACTCGCCGGTTATATCCGCCGCTTGCTTAG
>AA QryID: NC_009053_(+)_776317_776511 HitID: NC_010278_(+)_739288_739482
rank=1 bitscore=83.2 evalue=4.0E-14 perc_ident=76.56 perc_conserv=78.12 perc_gaps=0.0
query_cvg=100.0 subj_cvg=100.0 alpha=76.56 beta=16 prob=0.9998338699251865
TTGATAATAAGCGGTCAAAAACGGCCGCTTTTTTACACTATTCGAGGCAATATGAAAAAA
GTAATTTTATTAATAGCAAGCATTTTAGCAATAAGTGCTTGTTCTCAATCTAAAAACGTT
TATTTTAACGGTGCTGAAGGCTCAAACTCAGGTATCAAATACGAATCAACCAGCAAAGAG
TTTTCATTAAACTAG
>AA QryID: NC_009053_(-)_925491_925610 HitID: NC_010278_(-)_887895_888014
rank=1 bitscore=58.2 evalue=6.0E-7 perc_ident=100.0 perc_conserv=100.0 perc_gaps=0.0
query_cvg=100.0 subj_cvg=100.0 alpha=72.96 beta=1 prob=0.999832368254628
ATGTCTATTGTTGCAATTTTAGGAAATAAGTTTAGAATACAGAAAGCTAAATTATTGAAA
GGTAGTTTAGTCAGTCGTAGAGACAAAAAATTTTATTCATTATGCTTATCAAAAGGCTAA
>AA QryID: NC_009053_(+)_609851_609985 HitID: NC_010939_(+)_641303_641437
rank=1 bitscore=57.4 evalue=2.0E-6 perc_ident=100.0 perc_conserv=100.0 perc_gaps=0.0
query_cvg=100.0 subj_cvg=100.0 alpha=100.0 beta=3 prob=0.9998302896005287
ATGGAAAAGATAGCACAGGCTTTAACTGTCGCCGGTTCGGATAGCGGTGGCGGAGCGGGG
ATTCAGGCGGATTTCAAAACCTTCCAAATGCGAGGCGTTTTCGGCACATCGGTACTGACC
GCAATTACCGCGTAA
>AA QryID: NC_009053_(-)_1740497_1740613 HitID: NC_010939_(-)_1764699_1764815
rank=1 bitscore=57.4 evalue=1.0E-6 perc_ident=100.0 perc_conserv=100.0 perc_gaps=0.0
query_cvg=100.0 subj_cvg=100.0 alpha=59.72 beta=5 prob=0.9998293681780213
ATGATGAAAAAATTATTTTTAGCGGCGTTAATCGCATCGTTCGGGTTGGCTGCCTGCGGT
GTAAAAGGCCCGCTTTATTTTCCCGAGCAACAACCGGCTCAACAACAAACAAAATAA
>AA QryID: NC_009053_(-)_1351681_1351773 HitID: NC_010278_(-)_1317141_1317233
rank=1 bitscore=53.5 evalue=2.0E-5 perc_ident=100.0 perc_conserv=100.0 perc_gaps=0.0
query_cvg=100.0 subj_cvg=100.0 alpha=71.49 beta=1 prob=0.9998281140072074
GTGACACCGATTTACGGATATTGTGCAAAACAAGCGGTCAATTTTCTTCAAAAATTTACC
AGCCGTACTATTTTGTGTTGTCCGTGGTTTTAG
>AA QryID: NC_009053_(-)_2054459_2054647 HitID: NC_010278_(-)_2016142_2016330
rank=1 bitscore=93.6 evalue=3.0E-17 perc_ident=100.0 perc_conserv=100.0 perc_gaps=0.0
query_cvg=100.0 subj_cvg=100.0 alpha=77.94 beta=55 prob=0.9998234451669142
ATGAATTGTGAGCATGCCTCAGAATTAATTTCACTTAGCTGCGAACAAAAACTGAAAGTT
AAAGAAAGTGTCCGGCTTCAAGTGCATTTATGGCTTTGCCCTAAATGCCGTCATTTTAAA
CAAAATAATGATAAATTGCGCCAAATGCTGCACGAATACTGCCAACAAGATAAAACTAAT
TTGAAGTAA
>AA QryID: NC_009053_(+)_584635_584871 HitID: NC_010939_(+)_548285_548521
rank=1 bitscore=63.9 evalue=3.0E-8 perc_ident=53.85 perc_conserv=53.85 perc_gaps=0.0
query_cvg=100.0 subj_cvg=100.0 alpha=53.85 beta=1 prob=0.9998191202645645
ATGCGTGATTTTATCCAGTTTTGGGCAAAATTCTTTTTTATAATGCTTATTGCATTAGGT
GCATTATTTCTGTTCTTTGCTATTGATTTTGTCTATATTTTCGCATTTTTTAGCGTGTTT
GCTATTGTGTTTTTTTGTCCGTTTAGTAACGGTCATTAGTCAAACTCACAAACATTATGAT
GATTTAGCAAAGGCGGAAAAAGAAAAAGCCCGTGTCAAATACGTCATTATTCAGTAA
>AA QryID: NC_009053_(+)_2052542_2052637 HitID: NC_010278_(+)_2014225_2014320
rank=1 bitscore=49.7 evalue=2.0E-4 perc_ident=100.0 perc_conserv=100.0 perc_gaps=0.0
query_cvg=100.0 subj_cvg=100.0 alpha=100.0 beta=1 prob=0.9998173395719308
TTGAATATGATTTTCTATATTAAGCGGTCGGATTTAAGAAATTTTTTGCAAATTGCAAAA
TTCTGTGAAAATTCGACCGCTTTTGTTTTAAAATAG
>AA QryID: NC_009053_(+)_402286_402411 HitID: NC_010278_(+)_407116_407241
rank=1 bitscore=44.7 evalue=0.007 perc_ident=97.56 perc_conserv=97.56 perc_gaps=0.0
query_cvg=100.0 subj_cvg=100.0 alpha=97.56 beta=1 prob=0.9997708285333903
GTGTTTATACAGGTGATTTTACTGATTAAAAAAACAGTGTCAATCCTGTTTCTACTATTT
TTTGTAAAAATATCCAGTATGTTAATTCCGTTTTGGGAGGCGTATTTAGCAAGCGGTGGA
ATTTGA
>AA QryID: NC_009053_(+)_1508281_1508466 HitID: NC_010278_(+)_1457267_1457437
rank=1 bitscore=50.8 evalue=2.0E-4 perc_ident=76.79 perc_conserv=76.79 perc_gaps=0.0
query_cvg=91.8 subj_cvg=100.0 alpha=73.64 beta=2 prob=0.9997413265060235
TTGCTTGGAGGAACTATGCTCGAAATTTATCTGCTTGCCATCCCGTTTTATTTTCTGATC
GTCTTCTTTTCACAATTCTTTCGTCTTGGTTTGCTTAAAGCCGTATTGCTTAGCTTATTG

ATTGCCGTCACTTGGCCCGTTTCATTCGTCGTTCTTGGGTTTAACCGACTTTTTCGTTCT
CGTTAA
>AA QryID: NC_009053_(+)_332091_332189 HitID: NC_018690_(-)_1228563_1228661
rank=1 bitscore=42.4 evalue=0.034 perc_ident=100.0 perc_conserv=100.0 perc_gaps=0.0
query_cvg=100.0 subj_cvg=100.0 alpha=76.02 beta=9 prob=0.9994240247928476
ATGTTTTATGTAACTTGGGTATTAGGCGTTTTACTTGCGATTCTTTTTGCCACGGTAATT
ACTGTTAGCATTGAGAAAACAGGCAAATTTGACGAATAA
>AA QryID: NC_009053_(+)_1946290_1946391 HitID: NC_010278_(+)_1908160_1908261
rank=1 bitscore=36.2 evalue=3.0 perc_ident=96.88 perc_conserv=100.0 perc_gaps=0.0
query_cvg=96.97 subj_cvg=96.97 alpha=93.94 beta=1 prob=0.9992101481508742
ATGGAACAAAAAATAAAGCCGATTTTATTCAAATCGGCTTTAAGTGAGAGTATGTCTAGT
TGCATTAAAATGCAGGTTTTGCAAATTTTTATGAAAACTTAA
>AA QryID: NC_009053_(+)_546222_546425 HitID: NC_010278_(+)_553161_553358
rank=1 bitscore=48.9 evalue=9.0E-4 perc_ident=57.41 perc_conserv=75.93 perc_gaps=0.0
query_cvg=80.6 subj_cvg=83.08 alpha=32.13 beta=5 prob=0.9978556668053922
TTGTTTTTTGATAAGCTGTTTATCCTTTATAATGAAATTAAGGAGAAAATAATGACAGAA
GAATATCGTGAGTTTCTTGAGAAAAAAGTATCAGAAGCAAGAAAAAGTATTGAGCGTGGC
GATGTTTATTCCCATGAAGAAGTATGGGAACGAGCAATGAGGGCGATAGTGTTAGCTGAG
AAAAATACACAAGATGCAGCATAA
>AA QryID: NC_009053_(+)_1789810_1789986 HitID: NC_010278_(+)_1750548_1750724
rank=1 bitscore=37.7 evalue=1.9 perc_ident=53.45 perc_conserv=53.45 perc_gaps=0.0
query_cvg=100.0 subj_cvg=100.0 alpha=53.45 beta=1 prob=0.9821054163789433
ATGAAAGAGTTTATTATTTTGATTTCTATAATTATAATTCCAGTCATTATTGTTAAGTTA
TTAGATTTATCCATATTTATAAGAATATCTAAAAATAAAAAACAAAACGAACTATATCAA
ATAATATGTTACATAACTATTATATTAATGATAACCATATACAAAAATAAATTTTAA
>AA QryID: NC_009053_(-)_396904_397065 HitID: NC_010278_(-)_401734_401895
rank=1 bitscore=37.0 evalue=3.2 perc_ident=49.06 perc_conserv=49.06 perc_gaps=0.0
query_cvg=100.0 subj_cvg=100.0 alpha=49.06 beta=2 prob=0.9534079457430178
ATGTTGAATATGCCGAAACAAAAATGGATTAAAATTATAATCATCTTAATTTATTTGTTC
AGCCCGATTGATATTCTGCCCGAGGCGGTATTAGGTTCGCTCGGTTTGGTTGATGATGCG
GCGGCAATTTTGCTGTTAATTCAAACCGTGTTGAAAAAATAA

- ***Bacillus subtilis* 168**

vsORFs

>AA QryID: NC_000964_(-)_972856_972945 HitID: NC_014976_(-)_3183730_3183819
rank=1 bitscore=95.2 evalue=3.0E-18 perc_ident=96.55 perc_conserv=100.0 perc_gaps=0.0
query_cvg=100.0 subj_cvg=100.0 alpha=49.42 beta=20 prob=0.9987508243423942
GTGAAAACAATCATCATGATGGGTGTCATTGTCACTTTTTTAGTATCAATTTTTACCGCA
GGATACGAAAGCAAGCCTGGAAAAGAATAA
>AA QryID: NC_000964_(+)_820729_820800 HitID: NC_019896_(-)_3244074_3244145
rank=1 bitscore=82.1 evalue=3.0E-14 perc_ident=100.0 perc_conserv=100.0 perc_gaps=0.0
query_cvg=100.0 subj_cvg=100.0 alpha=36.8 beta=1 prob=0.9987474050202934
ATGAGAAAATTAAGAAAAAACTGGAGAAAATCGATTGTTAGCCCGGTTCTTTATACTAAT
GAAAAATGGTAA
>AA QryID: NC_000964_(+)_2299311_2299385 HitID: NC_009848_(+)_1972301_1972375
rank=1 bitscore=47.7 evalue=6.0E-4 perc_ident=62.5 perc_conserv=70.83 perc_gaps=0.0
query_cvg=100.0 subj_cvg=100.0 alpha=17.36 beta=1 prob=0.8711051052480673
ATGATTGACGCTCTGTTTGCCACAGTTTCACTCTTAACATTATTATACTTTGTCGTCATG
GAGGTCACCGATTAA
>AA QryID: NC_000964_(-)_1525119_1525190 HitID: NC_006510_(-)_1080943_1081014
rank=1 bitscore=44.8 evalue=0.005 perc_ident=65.22 perc_conserv=69.57 perc_gaps=0.0
query_cvg=100.0 subj_cvg=100.0 alpha=-4.35 beta=2 prob=0.8092166630568011
ATGCTGCGTAAATTAAGAAAGAAACTCAGAAGACAATGGAACGGAATGCTCCGTAAGAAA
GCAATTGCCTGA

sORFs

>AA QryID: NC_000964_(+)_1192627_1192797 HitID: NC_017743_(+)_1240526_1240696

rank=1 bitscore=81.3 evalue=1.0E-13 perc_ident=78.57 perc_conserv=78.57 perc_gaps=0.0
query_cvg=100.0 subj_cvg=100.0 alpha=51.78 beta=40 prob=0.998620995816329
ATGATGTACCTGCTGGCAGTTTTATGCCCGCCGCTTGCTGTTTTGTTTTCGGGAAAACCG
TTTCAGGCCTTGCTGAATCTCATATTGACCTGTATCTTTTGGCTGCCGGGCGCGATTCAC
GCCTGCTTTATCGTCGCCGACCGCCGTGCCGAAAAACGGGCAAGAAGATAA
>AA QryID: NC_000964_(-)_3119273_3119431 HitID: NC_018520_(-)_3050712_3050870
rank=1 bitscore=93.6 evalue=2.0E-17 perc_ident=100.0 perc_conserv=100.0 perc_gaps=0.0
query_cvg=100.0 subj_cvg=100.0 alpha=77.88 beta=16 prob=0.9986209406520539
TTGACATTAAGGGAGGCAGTTATGAAACATAAAAAACGAAACAAACGTAACGTGCCAATG
CTGATTGCGGCATCTGTGATCGCTTGTTCTTTTTTTCTTGTTCTTGTGATGATCGGTTTT
GAATGGCAAAAAAGTATAATTGATCATTTTTATCTATAA
>AA QryID: NC_000964_(-)_1454832_1454957 HitID: NC_014479_(-)_1410677_1410802
rank=1 bitscore=75.1 evalue=5.0E-12 perc_ident=100.0 perc_conserv=100.0 perc_gaps=0.0
query_cvg=100.0 subj_cvg=100.0 alpha=78.19 beta=14 prob=0.9986209406334523
ATGGGTTTCTTCATTTTAATTGGGTTATGTCTCACTGTTCTCGGTGGCAGCTGTCTTGCT
GTAAAACCTCTCAACCAAAAACATGAAATTAAACCCTTTCGAACATTTGATGATTTTTTT
ATTTAA
>AA QryID: NC_000964_(-)_2840803_2840925 HitID: NC_018520_(-)_2772242_2772364
rank=1 bitscore=72.0 evalue=4.0E-11 perc_ident=100.0 perc_conserv=100.0 perc_gaps=0.0
query_cvg=100.0 subj_cvg=100.0 alpha=100.0 beta=16 prob=0.9986209406319673
ATGGAAAAAACGTATTGTCATTCCGGCGAGAAGGACCCAGAAGCGGTCTACTTAACTGTG
AGAGGCTATCGAATTAAGCTGTCGCCTGAACAAATGAAACTGATTTCTGCCGTCAGAAAG
TAA
>AA QryID: NC_000964_(+)_2516215_2516367 HitID: NC_014976_(+)_495354_495506
rank=1 bitscore=82.8 evalue=4.0E-14 perc_ident=98.0 perc_conserv=98.0 perc_gaps=0.0
query_cvg=100.0 subj_cvg=100.0 alpha=98.0 beta=10 prob=0.9986209406212655
TTGAAAGAAGTTAAAGCCATGAAAAAATTCGTTCTGCTGCTGGCGGCAGCTGTCATCACA
TGCTCCATCGGCCACCAAATCAGTGGCGGCTACAAAGGCCCCGAAAAAGAGCGGCCAAAA
CATCACTCTGCACTCAAGATATTCAGTCGGTAA
>AA QryID: NC_000964_(-)_1903181_1903468 HitID: NC_019896_(+)_2166164_2166451
rank=1 bitscore=154.0 evalue=1.0E-35 perc_ident=97.89 perc_conserv=100.0 perc_gaps=0.0
query_cvg=100.0 subj_cvg=100.0 alpha=97.89 beta=4 prob=0.9986209406134114
TTGGAGCATACTGGTCATAGTTCGTTTCTTCAGCTCTTTTGTTTAAACCTAACTTTAATA
AGGGTGGAAATTGGTATGAAATATGAAAAAACAAGTCTTTTTGGAATGATTTTAGTTATT
GTAGCACTTCTAATATCTATATATTTTATGTGTAATACAAATGCGTTGATACCTGCTGGA
TATGATCTAGCTATTCATGGAATTGTAATATCTAGAACTGTAATGATTGCTTTTACTTTG
TATTTAGTTTCAAAGTTAGGATATTTTCTTCTGAATAAAAAAGATTAA
>AA QryID: NC_000964_(-)_3035518_3035700 HitID: NC_020244_(-)_2847302_2847484
rank=1 bitscore=108.0 evalue=6.0E-22 perc_ident=100.0 perc_conserv=100.0 perc_gaps=0.0
query_cvg=100.0 subj_cvg=100.0 alpha=72.32 beta=9 prob=0.9986209406099973
GTGGAAAATAAAAACCGTCTGCTCTATTCGACCATGCACATTTTGTTTTCATGTATCCTC
GCTCCAGCAGCAGAGAGTTACACGATACACCTTATTGATAAGGAACAAAATGAAAACATA
GAAGCTGAACAAAAAGGCTGCAATATTTCACACAAAGGTCATTATATAATTTATTTGTTT
TGA
>AA QryID: NC_000964_(-)_2637179_2637328 HitID: NC_017196_(-)_2440163_2440312
rank=1 bitscore=84.0 evalue=2.0E-14 perc_ident=100.0 perc_conserv=100.0 perc_gaps=0.0
query_cvg=100.0 subj_cvg=100.0 alpha=100.0 beta=5 prob=0.9986209405923908
TTGAAAAATCTAGAAGCTAAGGTAACGTTTTCTGTTCTGGTGATTATCGCCTTTTTACAA
GCTGTCCCGGGGGCTTGCGAACAGGCGTTTAATATAAATGGGGAAGCAGCAGTCAGGCTT
CCTCTTTACTATTTTGCAAACTCAATTTAG
>AA QryID: NC_000964_(-)_3054550_3054699 HitID: NC_017743_(-)_2918905_2919054
rank=1 bitscore=84.7 evalue=1.0E-14 perc_ident=100.0 perc_conserv=100.0 perc_gaps=0.0
query_cvg=100.0 subj_cvg=100.0 alpha=100.0 beta=4 prob=0.9986209405725643
ATGTACACGATGTTAAACGAAACGAGCTTGATAATGCAAATAAAATATGATAATGATAAT
GGGAAAGCCAATCTTACATATTCATTCGTTGCATATAAGTGGTTTTGCCTCAAGCAGTTT
GGTATGATCCAAATGGACGAGTATCGATAA
>AA QryID: NC_000964_(-)_2779302_2779436 HitID: NC_020244_(-)_2590780_2590914
rank=1 bitscore=70.5 evalue=2.0E-10 perc_ident=100.0 perc_conserv=100.0 perc_gaps=0.0
query_cvg=100.0 subj_cvg=100.0 alpha=100.0 beta=5 prob=0.9986209405536411
TTGCACAATTTCCGTCCATTTTTAGAAAAAGATGTTTACAAAATGGAATTTGGTGGATAT
ACTTACAAAGTAAGAAAAAAGCAAAGGAGGCTGAACCAAAATGACAATGAGACAACGACG
TCAACCTGTACGTGA

>AA QryID: NC_000964_(-)_3856782_3856937 HitID: NC_017196_(-)_3721549_3721704
rank=1 bitscore=95.9 evalue=5.0E-18 perc_ident=98.04 perc_conserv=100.0 perc_gaps=0.0
query_cvg=100.0 subj_cvg=100.0 alpha=98.04 beta=5 prob=0.9986209405499208
ATGCTTTTTACGGTTTCCAGAGAGGGAAGGACAGCTGAGATCTTCCTAACGCAGGGAAAG
CAGCTTACCGCCTTTGAGCCGTATCAGGGAAAACAAGTATCTGATACCGTCATGCTGCGT
TACAGCATCTTTAAGCCGATCATCATGTGTAATTGA
>AA QryID: NC_000964_(+)_1901781_1901945 HitID: NC_017196_(+)_1905041_1905205
rank=1 bitscore=99.4 evalue=4.0E-19 perc_ident=100.0 perc_conserv=100.0 perc_gaps=0.0
query_cvg=100.0 subj_cvg=100.0 alpha=100.0 beta=2 prob=0.9986209405481448
ATGAAATTAGAGAGAATTTATTTAAAGAAAGCCCAATTGCACATGGACAAATGACAATT
GATATTGAGGTAAAATACAATGCATTAAATCAGAAAAAGCTATTGGAGGATTTAATGTGT
TTAGACAATTTCCAATTTGGTATACACAAACACCTGACTATTTGA
>AA QryID: NC_000964_(-)_1453322_1453534 HitID: NC_020244_(-)_1455218_1455430
rank=1 bitscore=117.0 evalue=2.0E-24 perc_ident=98.57 perc_conserv=98.57 perc_gaps=0.0
query_cvg=100.0 subj_cvg=100.0 alpha=71.46 beta=7 prob=0.9986209405381934
TTGTTCCGTTTGCTGATGAATTTTTTCAGACGAAAAGCAGTTATGCTCTTAACGGTTTTG
GGAATTTCCGCTCCTTTTATCGGGATGAAGATGGTGGGCTTTTATTATTACAGCTCTATC
ATAGGCTGTGCAGCGGGTTTCCTTTGCTGCACCCTCGCTCTCATCCTAGTTATGAAAAGA
TCTGATAAAGACAAAAATGAAGGGAAAGCTTGA
>AA QryID: NC_000964_(-)_1056408_1056659 HitID: NC_019896_(+)_3018765_3019016
rank=1 bitscore=155.0 evalue=1.0E-35 perc_ident=100.0 perc_conserv=100.0 perc_gaps=0.0
query_cvg=100.0 subj_cvg=100.0 alpha=74.06 beta=2 prob=0.9986209405360805
ATGACCCTGTCTCTGTGTTTAGAACAACCTTTTCTTAAAGCAAAAAAAGCCTGGCTCAGC
AGACTTTATGTATGGCTTGTTGATAGGATACGATCGAAGCAGCCATTCTCCCTAATATCG
TTATTTCGATTTACGAAAAATCATGTATCAGGAACCCCTTGTTTGACCAATGATGCGCTC
CGAACACTTACTCTCTGTTGCTTATCCCCTCTATATAACGGCATGATATGCGACCGTATC
CATTTGAAATAG
>AA QryID: NC_000964_(+)_1447012_1447143 HitID: NC_017195_(+)_1424772_1424903
rank=1 bitscore=89.7 evalue=2.0E-16 perc_ident=100.0 perc_conserv=100.0 perc_gaps=0.0
query_cvg=100.0 subj_cvg=100.0 alpha=100.0 beta=2 prob=0.9986209405232813
ATGAGGGGGTTTTTACATAAAAAAGACGAGCGAATCCGGGGGGAATTCCACTCGCCGCTT
TCTAATGAAAAGACGGGATGCTGGATGGCAGCTCGTCTGTTTTATTATAATTTAACGAAC
GTATGTTTGTAA
>AA QryID: NC_000964_(-)_2706809_2707108 HitID: NC_019896_(+)_1519444_1519743
rank=1 bitscore=163.0 evalue=3.0E-38 perc_ident=98.99 perc_conserv=100.0 perc_gaps=0.0
query_cvg=100.0 subj_cvg=100.0 alpha=80.61 beta=1 prob=0.9986209405173104
TTGCTAAAATATAGAGTTCAGTCGTTCAGTAAAACATTCGAGCTGCTCTGCGAGGCCTTT
TCACAAAAAAGTGATTCCATCGCTCTTTCTTCCGCGGTAAATTTGGCGTTAAAATACTTC
AAAAGCTTTCTCCTGTCTTTTTGTATGATTCTTTTTCTTTGTTTCATTGGAAAACAAGCT
GCATGTAACCCTAATTTACTATTGTTCACTACCGTATTAAGTGTCGTATGCGGGATTTGT
CATCATTCTCGAATTTTTCCTGCGAAAAGTTACAAAAATAGTCGAATGAGGCGCCTTTGA
>AA QryID: NC_000964_(-)_1094734_1094925 HitID: NC_017196_(-)_1093057_1093248
rank=1 bitscore=73.6 evalue=3.0E-11 perc_ident=100.0 perc_conserv=100.0 perc_gaps=0.0
query_cvg=100.0 subj_cvg=100.0 alpha=100.0 beta=3 prob=0.9986209405070068
ATGAACAGAAATGTCGTTGTCACGATTGGTTCGATGATTTTTGGTTCTTTGACGAGTGGT
TCGATAAAGATTTCCACGACCATTGCAAGAAAAAGAAAGAATGCCATGACCCTTGCGAGA
AGAAGAAAAAGAAACACCATTGTTTCTCTTGTAAAAAACACAGACATTCTTGCTGTCATT
GCTAAAGACTGA
>AA QryID: NC_000964_(-)_3951824_3951958 HitID: NC_020244_(-)_3793182_3793316
rank=1 bitscore=72.8 evalue=4.0E-11 perc_ident=100.0 perc_conserv=100.0 perc_gaps=0.0
query_cvg=100.0 subj_cvg=100.0 alpha=100.0 beta=3 prob=0.9986209405029496
ATGGAATGTTTCGAGCTGATTCATAATAAAGGAAAAGATACCTGGAGTCAAAAGATCTTT
TTAACTTTTAAAAAGTGTAATGTACTTAAAAAAAATTTTTCTGTACCTGTTCGTTCAAGTG
AAAGCAGAAACCTAA
>AA QryID: NC_000964_(-)_3804714_3804875 HitID: NC_017196_(-)_3669468_3669629
rank=1 bitscore=82.4 evalue=5.0E-14 perc_ident=100.0 perc_conserv=100.0 perc_gaps=0.0
query_cvg=100.0 subj_cvg=100.0 alpha=76.93 beta=6 prob=0.998620940502674
ATGTTTTTGTCCTCGCTTCCTTCAAAACTCATTAAGACAATCCTTTTAACAAAAGCGCCG
ATCATGGGTGGAAGTGTTACCGATAGAAGTTCCTTTATGTTAAAAAAAGAATGTGCTTTG
GCGGATGTTGTGCAAAAGCAGCAGATGACATGTTCAGAGTAA
>AA QryID: NC_000964_(+)_1279301_1279495 HitID: NC_014976_(+)_3491841_3492035

rank=1 bitscore=110.0 evalue=2.0E-22 perc_ident=96.88 perc_conserv=98.44 perc_gaps=0.0
query_cvg=100.0 subj_cvg=100.0 alpha=96.88 beta=4 prob=0.9986209404899974
ATGAACAAAGAAACCCTAAAAAACATACTCATGATTTTTTCAATATGCGCAACACTCCGA
GAAACCGATGGTATGCGCATAGCTGATAGCTGGATTTTGAGTGAGATTATACGATTTTTG
ATTTGCATGGTTGTGTTTTTTGCCATGACATTTCTATTAGATGCTGTTTTTCCAAACAAA
AAAGAGTCACATTAA
>AA QryID: NC_000964_(+)_1094747_1094929 HitID: NC_017195_(+)_1073416_1073598
rank=1 bitscore=65.5 evalue=8.0E-9 perc_ident=100.0 perc_conserv=100.0 perc_gaps=0.0
query_cvg=100.0 subj_cvg=100.0 alpha=100.0 beta=3 prob=0.9986209404409288
ATGACAGCAAGAATGTCTGTGTTTTTTACAAGAGAAACAATGGTGTTTCTTTTTCTTCTT
CTCGCAAGGGTCATGGCATTCTTTCTTTTTCTTGCAATGGTCGTGGAAATCTTTATCGAA
CCACTCGTCAAAGAACCAAAAATCATCGAACCAATCGTGACAACGACATTTCTGTTCATG
TGA
>AA QryID: NC_000964_(+)_599684_599824 HitID: NC_014976_(+)_2789599_2789739
rank=1 bitscore=70.5 evalue=2.0E-10 perc_ident=100.0 perc_conserv=100.0 perc_gaps=0.0
query_cvg=100.0 subj_cvg=100.0 alpha=100.0 beta=2 prob=0.9986209404401633
ATGAAAACAGAAATGACAAGGCCTGTTATTAAGGCAACTGACGGTACAAAAGTTTTGGGG
ATTTTCAATAATATTTTGAGGATTTGGGTTATCAATATGATGATTGGAACAGCTATCACA
GCATCCCAAAAGTTCGTGTGA
>AA QryID: NC_000964_(+)_2898771_2898929 HitID: NC_014976_(+)_907295_907453
rank=1 bitscore=96.7 evalue=3.0E-18 perc_ident=100.0 perc_conserv=100.0 perc_gaps=0.0
query_cvg=100.0 subj_cvg=100.0 alpha=78.76 beta=3 prob=0.9986209404127278
GTGCAGGGGAAGGTCTGTCTTATCTATCTTTTTTTATATTCACACATTTTTCACCTTTAT
GATACACGCCAATGTTTTCAATTTTGTTTAATTCAAAGAACAGCGGGAATTACAAACGAC
ATGAACGATATCATGCAAATTCATAAAGGAGGAGATTGA
>AA QryID: NC_000964_(-)_1784706_1784837 HitID: NC_017195_(-)_1759162_1759293
rank=1 bitscore=73.2 evalue=2.0E-11 perc_ident=100.0 perc_conserv=100.0 perc_gaps=0.0
query_cvg=100.0 subj_cvg=100.0 alpha=100.0 beta=1 prob=0.9986209403967288
ATGTGGGAGCGATGGTTTGACAGACCGGCTGACATAGTTCAAATGTCTAACTTAATTTAT
CAGCCTCTTTATGTAACAGTATATAAAGTTATTCAATTAGAAACAGTGTCAATCGAACGA
TTGAATCTTTAA
>AA QryID: NC_000964_(+)_376560_376667 HitID: NC_014976_(+)_2591035_2591142
rank=1 bitscore=59.3 evalue=3.0E-7 perc_ident=100.0 perc_conserv=100.0 perc_gaps=0.0
query_cvg=100.0 subj_cvg=100.0 alpha=100.0 beta=6 prob=0.9986209403824597
TTGCGGCATCCCGCAAAAAATATTGCTGTAAATAAACTGGAATCTTTCGGCATCCCGCAT
GAAACTTTTCACCCATTTTTCGGTGATAAAAACATTTTTTTCATTTAA
>AA QryID: NC_000964_(-)_3317352_3317465 HitID: NC_017195_(-)_3113330_3113443
rank=1 bitscore=65.1 evalue=5.0E-9 perc_ident=100.0 perc_conserv=100.0 perc_gaps=0.0
query_cvg=100.0 subj_cvg=100.0 alpha=100.0 beta=2 prob=0.9986209403813575
ATGCTTGAGAAGTTTACGACTTTTTCTTGCGCCTTCTTCGGATTAAAATATGTATTCATA
ATTCCGGCTGTGTATGACTACGATGTGATGAGCCCTAATTCAACAGGCAGGTGA
>AA QryID: NC_000964_(+)_1300296_1300430 HitID: NC_017743_(+)_1348001_1348135
rank=1 bitscore=66.6 evalue=3.0E-9 perc_ident=97.73 perc_conserv=100.0 perc_gaps=0.0
query_cvg=100.0 subj_cvg=100.0 alpha=97.73 beta=4 prob=0.9986209403063384
GTGCTTTCTGGCAATGTCTTTTTTTATGTTTATGACAGCGTTAACATTTCATTTTCAAAA
AAGAGATTGATCCCAAAGGAATATAAAGGTAAAATAAAAACAAATCAAAATGTTAACGTT
AACATTTTGAAATAG
>AA QryID: NC_000964_(-)_4005275_4005379 HitID: NC_014976_(-)_2012143_2012247
rank=1 bitscore=64.7 evalue=8.0E-9 perc_ident=100.0 perc_conserv=100.0 perc_gaps=0.0
query_cvg=100.0 subj_cvg=100.0 alpha=100.0 beta=1 prob=0.9986209403024802
ATGTCAGTACTGATTTTTCTTCACTATAAGGTTCCAGCTCCTGGATATTTGTTGTATGAT
GGAAACGGGGAAACCCATACAGCAGAAAGGGAGTTTTTTTCATGA
>AA QryID: NC_000964_(+)_3573039_3573176 HitID: NC_017196_(+)_3433571_3433708
rank=1 bitscore=77.0 evalue=2.0E-12 perc_ident=100.0 perc_conserv=100.0 perc_gaps=0.0
query_cvg=100.0 subj_cvg=100.0 alpha=73.62 beta=4 prob=0.9986209402978412
ATGGACAAAAAAAGGACACGGATAACTGGAGAATCCGTGCCAAAAAAGTGTATTCATTAT
AAAAAGGGGGTCAATTACTTTATTAATCATAACCGAAAAGCATTACACATGTGTTACAGC
GGAATTAAAACCCAGTAA
>AA QryID: NC_000964_(-)_2302502_2302657 HitID: NC_017196_(-)_2104421_2104576
rank=1 bitscore=77.4 evalue=2.0E-12 perc_ident=98.04 perc_conserv=98.04 perc_gaps=0.0
query_cvg=100.0 subj_cvg=100.0 alpha=98.04 beta=2 prob=0.9986209402812911
TTGTTGAAAAATATCCGAAAACTATTTATAGTAAGTAACAATATTAATAGTTGGAGGGTT

CAAATCGAAAGAAAGCTATATAAATATAAAAACACGAAAGAACAAAACAAGGTGAGGGCG
GCCTGTATTTATCTTATTTGCAAAAACAGCCCATAA
>AA QryID: NC_000964_(+)_3335127_3335369 HitID: NC_019896_(-)_891612_891854
rank=1 bitscore=126.0 evalue=4.0E-27 perc_ident=98.75 perc_conserv=98.75 perc_gaps=0.0
query_cvg=100.0 subj_cvg=100.0 alpha=76.48 beta=1 prob=0.9986209402560602
TTGCCTACGAGAAAAATACCCTGTAGTCCTTTCATTTATAAATCGAGGTTTCAAACTAAT
AAGTCAGCTCTTAGCAGCCGAGCTCCCTTCTCCAATCCTATAAACAGACCGCGTTTTTTC
TCGAAAAAATACTCTGAAGACAATTGCTGCGTTTTTAATAGTTTTGATAACGCGTTCTCT
CTTACACCTTTAGACAAAATATTCATTTTTTCTTTTTTAGACTTAAATCTATGTCGAAAA
TAA
>AA QryID: NC_000964_(+)_692590_692730 HitID: NC_017743_(+)_717577_717702
rank=1 bitscore=76.3 evalue=3.0E-12 perc_ident=97.56 perc_conserv=100.0 perc_gaps=0.0
query_cvg=89.13 subj_cvg=100.0 alpha=92.26 beta=4 prob=0.9986209402439195
GTGTTGTTAGAATTAGTGAATATTATCGGAGTCTGGGAGCGAGTTGGCCTGACTCCGGCA
AACGGCCTTGCCAAAGAGGGCGGAGCGAGCTTCATATCTGTCCTCGTTTTTTTCTGTGTC
AAAAAACTTAGAGGAGATTAG
>AA QryID: NC_000964_(+)_1492057_1492185 HitID: NC_017196_(+)_1497554_1497682
rank=1 bitscore=59.7 evalue=2.0E-7 perc_ident=100.0 perc_conserv=100.0 perc_gaps=0.0
query_cvg=100.0 subj_cvg=100.0 alpha=100.0 beta=1 prob=0.9986209401718704
TTGAGACTAGTTACCCTGTACATGACCATGTTAAACGTTTTATTCAAAAATGTCGAAGCC
AAAGACATACATGATTTGTTAAAAATTGACTCAGGAAATCTGCTTTTATATAATGGCATT
GCAATATAA
>AA QryID: NC_000964_(+)_2854637_2854759 HitID: NC_020244_(+)_2666264_2666386
rank=1 bitscore=68.9 evalue=4.0E-10 perc_ident=100.0 perc_conserv=100.0 perc_gaps=0.0
query_cvg=100.0 subj_cvg=100.0 alpha=71.3 beta=4 prob=0.9986209401673999
TTGTTAGAAAAACAATTGTTTTTCCCAGAAATATCATGTTCCTTTTTTCTGGCTTTGGTT
GATTTGCAGCCAGGCAGAACATTTTATGTATTACTGCTTGGGGAACATATCCAGTGGGTT
TGA
>AA QryID: NC_000964_(-)_1443729_1443980 HitID: NC_019896_(+)_2625951_2626223
rank=1 bitscore=127.0 evalue=2.0E-27 perc_ident=98.72 perc_conserv=100.0 perc_gaps=0.0
query_cvg=93.98 subj_cvg=86.67 alpha=66.97 beta=1 prob=0.998620940167354
GTGTGGGATGCCCCACAAAAAAAGAAAGCAACCTACAACATATTTTCCTATGTGGTTATT
CAGGACATAAGGATTCCTTTAAATATTTTTAAAAGTATTAGAATACAAGGACAGGGATAT
TATTTTTTATTAAAAGTCAAGAGCCCCCGTTACTTTAAGTACATTTATTCACAGTGTAAT
CTTTTTTACAGGTCATTTAGTAGTAGCCCCAGGAAAATGCGAATTTACAACGTTAATCAA
ATTCAAATATAA
>AA QryID: NC_000964_(+)_2367708_2367854 HitID: NC_017196_(+)_2169636_2169764
rank=1 bitscore=70.1 evalue=3.0E-10 perc_ident=100.0 perc_conserv=100.0 perc_gaps=0.0
query_cvg=87.5 subj_cvg=100.0 alpha=93.75 beta=1 prob=0.9986209401646594
ATGCCCCCGGCAAATAAGATGCTTCTGTACGGAAGGGAATTATTGAGTCTTATTAGTTTA
CCAAAAGATCTTGAGATAGAAAATGAAAAAAGGAAGATCGCCCTCTGTACAGTAAAGCAG
CTCTTTGACCTGCCGGCTTGTAAGTAA
>AA QryID: NC_000964_(+)_1874014_1874172 HitID: NC_017195_(+)_1848524_1848682
rank=1 bitscore=92.0 evalue=8.0E-17 perc_ident=100.0 perc_conserv=100.0 perc_gaps=0.0
query_cvg=100.0 subj_cvg=100.0 alpha=72.12 beta=2 prob=0.9986209401630672
ATGATTAAAAACAAAATCTCATGGTTTGTCCACCCCATGTCCGTGAATACAATAAGAAAT
AAAGTATTTCTCGGGAAAGCGCAGGATTCCTTATTGTTCCCGAAAAAAGCAATTGTTTCG
ACACATGATAGGCGTTTGTCACAATCGGCATCCGCTTGA
>AA QryID: NC_000964_(+)_718436_718597 HitID: NC_014976_(+)_2941663_2941824
rank=1 bitscore=90.5 evalue=2.0E-16 perc_ident=94.34 perc_conserv=96.23 perc_gaps=0.0
query_cvg=100.0 subj_cvg=100.0 alpha=94.34 beta=5 prob=0.9986209401494872
ATGTGTGCTCCTGCCGATACAGGACTCACCATGCAGGTACTGCCATATGCTCTCTTTTTT
TATGAAAAAAGAATCAAAACACCTTTTTCAGTGAATAACGAGATTAACGGCTTTTTCCGT
TTTTCCGCGCTATGTGAATTTGTTATAATGTTTAAATGGTAA
>AA QryID: NC_000964_(-)_1874018_1874179 HitID: NC_017195_(-)_1848528_1848689
rank=1 bitscore=99.8 evalue=4.0E-19 perc_ident=100.0 perc_conserv=100.0 perc_gaps=0.0
query_cvg=100.0 subj_cvg=100.0 alpha=73.96 beta=1 prob=0.9986209401468538
ATGATATTCAAGCGGATGCCGATTGTGACAAACGCCTATCATGTGTCGAAACAATTGCTT
TTTTCGGGAACAATAAGGAATCCTGCGCTTTCCCGAGAAATACTTTATTTCTTATTGTAT
TCACGGACATGGGGTGGACAAACCATGAGATTTTGTTTTTAA
>AA QryID: NC_000964_(+)_972872_973015 HitID: NC_016047_(+)_1082937_1083113

rank=1 bitscore=61.6 evalue=1.0E-7 perc_ident=97.87 perc_conserv=100.0 perc_gaps=0.0
query_cvg=100.0 subj_cvg=81.03 alpha=100.0 beta=2 prob=0.9986209401131871
TTGCTTTCGTATCCTGCGGTAAAAATTGATACTAAAAAAGTGACAATGACACCCATCATG
ATGATTGTTTTCACCGTCTCTCCCCCTCTTCAATTTTTCCGTGCTGTCCGAGTCTGGATG
ATTCTGTATTTTTCTTTATTATGA
>AA QryID: NC_000964_(+)_3257454_3257579 HitID: NC_017196_(+)_3020116_3020259
rank=1 bitscore=68.2 evalue=6.0E-10 perc_ident=97.56 perc_conserv=100.0 perc_gaps=0.0
query_cvg=100.0 subj_cvg=87.23 alpha=92.68 beta=2 prob=0.9986209401115949
GTGCTGAAATTGAGAAAGATTCTTAATCTCCTTGATTTTAACGGGTTTCGCATTTTCACT
AAGCAAGACTATAGTCCTTCATACCGTATTTTCAAAATTGAATATGCGATTCTTGGCGTT
TCCTGA
>AA QryID: NC_000964_(+)_1744246_1744365 HitID: NC_017195_(+)_1718717_1718836
rank=1 bitscore=66.2 evalue=3.0E-9 perc_ident=100.0 perc_conserv=100.0 perc_gaps=0.0
query_cvg=100.0 subj_cvg=100.0 alpha=76.51 beta=3 prob=0.9986209401098036
ATGTTTTTTTATATGGAAAAAGCGCATCTTTTATATTCACCGGTATTTCCTTTTGATCAT
AAGATGAAGGGGAGCTTAACAACTAGAGATCCAGTATATACAAAGAAGGTGAACGTTTAG
>AA QryID: NC_000964_(-)_2403381_2403503 HitID: NC_017196_(-)_2205299_2205421
rank=1 bitscore=55.1 evalue=6.0E-6 perc_ident=100.0 perc_conserv=100.0 perc_gaps=0.0
query_cvg=100.0 subj_cvg=100.0 alpha=100.0 beta=4 prob=0.9986209400694004
ATGCAACGGAAAACTGCTGATTTCAGCAGTTTTTTTTCGTTAAAAAACATGGAAAAACAT
AACGTTTATTCTATGCACAGATCATATGAAGGTGTATACTATTCTCTGAAAGGGAGCATG
TGA
>AA QryID: NC_000964_(+)_2119207_2119317 HitID: NC_014479_(+)_2106773_2106883
rank=1 bitscore=58.2 evalue=8.0E-7 perc_ident=97.22 perc_conserv=100.0 perc_gaps=0.0
query_cvg=100.0 subj_cvg=100.0 alpha=74.88 beta=13 prob=0.9986209400606278
ATGGAAATCGAAAGAATCATTCAGTATATTTGCACCTTTTTAGGCGTGATTTCGCTTTCT
TTTATTTATGCCCAAATGTTTAGAAAAACGAAACCGACAAAAAAAGAATAG
>AA QryID: NC_000964_(+)_3343894_3344031 HitID: NC_014976_(+)_1351492_1351629
rank=1 bitscore=70.5 evalue=2.0E-10 perc_ident=100.0 perc_conserv=100.0 perc_gaps=0.0
query_cvg=100.0 subj_cvg=100.0 alpha=76.84 beta=2 prob=0.9986209400297015
ATGAACAAGCTAAGCTGGAGAGGCGATGTTCGCGGAACGGATGAAAACAGAAATGCCGGC
AGCCCGCAAAAACAGGACCTAAAGATATGGAAACAGGAGATTTCCGCCTCCTCCAAAGGG
ACTAATTTACAATTTTAA
>AA QryID: NC_000964_(-)_4066033_4066155 HitID: NC_017743_(-)_3971147_3971269
rank=1 bitscore=74.7 evalue=8.0E-12 perc_ident=100.0 perc_conserv=100.0 perc_gaps=0.0
query_cvg=100.0 subj_cvg=100.0 alpha=74.05 beta=2 prob=0.9986209400076091
ATGAAAAGAAATTCCGTCAAAGAGGAACTACAATTATTTTCCTCAGCCCCTCTATCTTTG
AATGAAAACGGTAAAGATTGTATACTGGGTACAAACCCTTTTTATGTAAATGTTCCGTTT
TAA
>AA QryID: NC_000964_(-)_4014527_4014640 HitID: NC_017743_(-)_3915857_3915970
rank=1 bitscore=62.8 evalue=3.0E-8 perc_ident=100.0 perc_conserv=100.0 perc_gaps=0.0
query_cvg=100.0 subj_cvg=100.0 alpha=67.36 beta=4 prob=0.9986209399476091
ATGGGCCTGAAAAGAATGGAATGCACGTCAATTTTTACAAAATATTTATGTTTTTTCGCT
TCAAATCATACTTTTGATTTATTGCTGGGGTTAGGTGCATCTGGTACCATTTAA
>AA QryID: NC_000964_(-)_2096129_2096311 HitID: NC_014976_(-)_207624_207806
rank=1 bitscore=87.0 evalue=2.0E-15 perc_ident=95.0 perc_conserv=98.33 perc_gaps=0.0
query_cvg=100.0 subj_cvg=100.0 alpha=95.0 beta=3 prob=0.9986209399123349
TTGTATATACACATTAATATAGAAGAAAATAATCATTCGCTTTTAGCAATAAAGGCTATT
TTTTTTATTGTAATATATTTTTTGTTTGTCGAATTGTCAGGTATTTTGGAGAAAAAACAG
TTTTATTCACTGGAAAAAGTTTTTCTAAACAATTGTTCTGACCTCTTGATTAAAAGCGCC
TGA
>AA QryID: NC_000964_(-)_1252558_1252791 HitID: NC_017196_(-)_1242453_1242686
rank=1 bitscore=120.0 evalue=3.0E-25 perc_ident=96.1 perc_conserv=97.4 perc_gaps=0.0
query_cvg=100.0 subj_cvg=100.0 alpha=78.96 beta=2 prob=0.9986209399110795
ATGCTGAGCGAGACCGCCTCGTTAAAAAAACAAAAAACCGGAGTTCTCCGGTTTCTTTGC
CAGGCTATTGAGGCTTTTTCTTTTTATTTCCGCACCCGCAGCCTTTCTTTTTTATCTTTT
TTGATTGATACGATGAATCTGTATATGGAGATTTTTGGGAAGGTTTATGATGCTGATTCA
TTTTTGTCCCCTTCCTTTCCTTATGTCATATATGCTACTTTATGCGTATTCTGA
>AA QryID: NC_000964_(-)_3775382_3775522 HitID: NC_014976_(-)_1776749_1776889
rank=1 bitscore=75.9 evalue=6.0E-12 perc_ident=97.83 perc_conserv=100.0 perc_gaps=0.0
query_cvg=100.0 subj_cvg=100.0 alpha=73.65 beta=4 prob=0.9986209398918655
ATGATCACACAAGCTGTTTATGCTTTCTGGGCGGAAATGGCTAAAAATGAACGGCATTTC

AAGCGATTTTGGACAGATCGTGAAAGCAGCACAAAAGGCAGCATGTCCTATATCTTTTTG
CATTTTCGTATTCTAGCATAA
>AA QryID: NC_000964_(+)_3615472_3615585 HitID: NC_017196_(+)_3474702_3474815
rank=1 bitscore=65.9 evalue=3.0E-9 perc_ident=100.0 perc_conserv=100.0 perc_gaps=0.0
query_cvg=100.0 subj_cvg=100.0 alpha=74.76 beta=2 prob=0.9986209398761727
TTGCATTTTGGTGCCGGTCAAAACTTATTCCATGGTGCCGCTGTTCTAATCAGCGGCACT
TTTTTTATTACATCATCAAGTCCGCCGCCAGATTCCGAGAATAAAAGAAAATAG
>AA QryID: NC_000964_(-)_3153779_3153904 HitID: NC_014976_(-)_1162091_1162216
rank=1 bitscore=70.9 evalue=1.0E-10 perc_ident=100.0 perc_conserv=100.0 perc_gaps=0.0
query_cvg=100.0 subj_cvg=100.0 alpha=76.94 beta=1 prob=0.9986209398408984
ATGGTAAGCGGATACAACCTTTGCTATCAGTGGAGAAAGAAATTTAAGCTTTGTTTCTTT
TTCATTTCTGAAATTAGGTTTATAATAGGTAAGGCAGGCCATTTGGACTGCATGATCTGT
GTTTGA
>AA QryID: NC_000964_(+)_1298374_1298499 HitID: NC_017195_(+)_1275379_1275498
rank=1 bitscore=64.3 evalue=9.0E-9 perc_ident=97.44 perc_conserv=100.0 perc_gaps=0.0
query_cvg=95.12 subj_cvg=100.0 alpha=95.06 beta=2 prob=0.998620939784634
TTGTGTTTGCTATTTTCAGAGAGAGTAAAGGATTTTGACTTATTCAATTGTTTATTTTTA
GATAAAAATAATACTGAATATGGCAAGATGAAAAACTGGGTAGTAAACATTTATTTTTGT
AAATGA
>AA QryID: NC_000964_(-)_3918318_3918431 HitID: NC_020244_(-)_3759706_3759819
rank=1 bitscore=56.2 evalue=2.0E-6 perc_ident=97.3 perc_conserv=97.3 perc_gaps=0.0
query_cvg=100.0 subj_cvg=100.0 alpha=100.0 beta=3 prob=0.9986209396692118
GTGAATAAAGTTGGAGCAATTTTTGTCGATTTTATGACATTTAAGCGAAAATGGGATGAA
GTTTATAAAAAAATAAAGCTAATAAGTGAAGTTTTTGATAGCATAACAAAGTAG
>AA QryID: NC_000964_(+)_3105954_3106109 HitID: NC_017196_(+)_2868700_2868852
rank=1 bitscore=94.7 evalue=1.0E-17 perc_ident=98.0 perc_conserv=100.0 perc_gaps=0.0
query_cvg=98.04 subj_cvg=100.0 alpha=76.18 beta=1 prob=0.9986209396239707
TTGATGCATTGGATTCATTTTTCACCCAGTGACATTGACCTAGTCACCTATTTAGCATGT
AGAAAACCTTTCCGTTCATATGTTCAAATTCTGTTCTTATTTTTAGTGTATACGAGGACA
AGCCCTAATGACAAACAACAAACTGCACTTGCTTGA
>AA QryID: NC_000964_(+)_2913361_2913486 HitID: NC_020244_(+)_2724987_2725112
rank=1 bitscore=53.1 evalue=2.0E-5 perc_ident=100.0 perc_conserv=100.0 perc_gaps=0.0
query_cvg=100.0 subj_cvg=100.0 alpha=76.79 beta=3 prob=0.9986209395025776
ATGATGATTTTTAGTATAGCATCTCCCATTCGTTCACGCTATTTTAATGCTTACAAATTA
TTTTTTCCTGATCACAGCCGGTTTACTCATTTTTATATGGTTGAATGGGATATGCTCTTT
TTTTAA
>AA QryID: NC_000964_(-)_3893184_3893300 HitID: NC_017195_(-)_3696716_3696832
rank=1 bitscore=67.0 evalue=1.0E-9 perc_ident=94.74 perc_conserv=97.37 perc_gaps=0.0
query_cvg=100.0 subj_cvg=100.0 alpha=97.37 beta=3 prob=0.9986209394879257
TTGGATTATGCTGAAAAAGAACAGCGGACCGATCAGATTTCGCTCTGGGCAGCCGCTGTT
TTTCAGAAACATACACAAACTTTTCGCATATTGGTTTGTATCCCTAGCGCAACTTGA
>AA QryID: NC_000964_(+)_1219694_1219822 HitID: NC_017196_(+)_1209633_1209761
rank=1 bitscore=62.8 evalue=3.0E-8 perc_ident=100.0 perc_conserv=100.0 perc_gaps=0.0
query_cvg=100.0 subj_cvg=100.0 alpha=71.47 beta=1 prob=0.9986209393575609
ATGAACTTGTTCACTCTATTGTTACAGCTTTTTTACAAAAATAATCAGAAAAGACGGAAC
AGAATAAAAGTTGTGGAAACTCAGGTTCATTTGTCTGATATTTCTGAGGATTTAGCCGTA
AGGAGCTGA
>AA QryID: NC_000964_(+)_2987572_2987685 HitID: NC_017743_(+)_2851901_2852014
rank=1 bitscore=52.4 evalue=3.0E-5 perc_ident=97.3 perc_conserv=97.3 perc_gaps=0.0
query_cvg=100.0 subj_cvg=100.0 alpha=100.0 beta=3 prob=0.9986209390469206
ATGATTAAACATAACATGATCATTGCCCTAAAAACAATGAAAAGGGATCTCCATCCTCCC
TATGAAACCTTACCTATTATACAATTAAATAAATTGTTTTTAAATAAAATTTAA
>AA QryID: NC_000964_(+)_2733649_2733762 HitID: NC_017196_(+)_2494860_2494994
rank=1 bitscore=55.5 evalue=4.0E-6 perc_ident=100.0 perc_conserv=100.0 perc_gaps=0.0
query_cvg=100.0 subj_cvg=84.09 alpha=64.05 beta=1 prob=0.9986209389000822
TTGGAAAATGCGTTCTATACTGCAACTGTGCCCAAGCCGTTAACCTTCAAATCTAATCTG
AGAATCACTGGCTCTACAGAGAATAATAAAAAACACTCTAATAACGAGTGCTGA
>AA QryID: NC_000964_(-)_329633_329755 HitID: NC_017743_(-)_323950_324072
rank=1 bitscore=53.5 evalue=2.0E-5 perc_ident=100.0 perc_conserv=100.0 perc_gaps=0.0
query_cvg=97.5 subj_cvg=97.5 alpha=74.62 beta=1 prob=0.998620938837357
ATGTTTCTCTTTGATGTCTTTTTGTTTGTGAAGTATTTCACATTTATATTGTGCAACACT
TCACAAACTTTTGCAAGAGAAAAGTTTTGTCTGATTTATGAACAAAAAAGAAACCATCAT

TGA
>AA QryID: NC_000964_(+)_1933117_1933281 HitID: NC_017195_(+)_1915129_1915272
rank=1 bitscore=77.0 evalue=3.0E-12 perc_ident=95.74 perc_conserv=100.0 perc_gaps=0.0
query_cvg=87.04 subj_cvg=100.0 alpha=89.54 beta=1 prob=0.9986209386600823
TTGCTTTTTAATTTTAAGATATTGTTTGCGTATTACGGTGAAACAGATACAATAAAGCTT
GAACAGCTTTTTTTGAATACCTTGTCCAGCTGGGTTTCGGCCGTTCGATTTGCGAAGGTC
TGCTGCTTTATATATAATAAACAAACATACGTTCTCATAGTGTAA
>AA QryID: NC_000964_(+)_1442020_1442151 HitID: NC_017195_(+)_1419557_1419688
rank=1 bitscore=52.4 evalue=4.0E-5 perc_ident=100.0 perc_conserv=100.0 perc_gaps=0.0
query_cvg=100.0 subj_cvg=100.0 alpha=71.86 beta=1 prob=0.9986209386438689
TTGATAGTACAGAAGCAGATTAAGGCGTACAATTTGTACGTCTTTTCTTTTTTATCCGAA
AGAAAAAATGCCTATCTAGAGATCATAGGCATTAAATATTATTATCAATATCTTCATTTA
TTTTTTGGCTAA
>AA QryID: NC_000964_(+)_282240_282386 HitID: NC_020244_(+)_275281_275427
rank=1 bitscore=84.7 evalue=1.0E-14 perc_ident=95.83 perc_conserv=95.83 perc_gaps=0.0
query_cvg=100.0 subj_cvg=100.0 alpha=68.84 beta=3 prob=0.9986209385689571
GTGCAAATCTGCTGGGGAGAGGTCAATGATGTGAATCCAGGAATTGAGGCTGCGGCGCTT
CGACAGCGGAAACGTGAAGCTTGTACTGCACCGGACGCTGCTTTGATGCCCAACAGCCCA
TTATGTTTGCTGTTTTTAGATGAATAA
>AA QryID: NC_000964_(+)_4005262_4005411 HitID: NC_017195_(+)_3806975_3807109
rank=1 bitscore=83.6 evalue=3.0E-14 perc_ident=93.18 perc_conserv=95.45 perc_gaps=0.0
query_cvg=89.8 subj_cvg=100.0 alpha=90.58 beta=3 prob=0.9986209385247876
ATGAATTGGATAATCATGAAAAAAACTCCCTTTCTGCTGTATGGGTTTCCCCGTTTCCAT
CATACAACAAATATCCAGGAGCTGGAACCTTATAGTGAAGAAAAATCAGTACTGACATGC
TGGTATCCCGCCAACATCATATCAGCATAG
>AA QryID: NC_000964_(+)_3361158_3361277 HitID: NC_017195_(+)_3158695_3158814
rank=1 bitscore=60.5 evalue=1.0E-7 perc_ident=97.44 perc_conserv=97.44 perc_gaps=0.0
query_cvg=100.0 subj_cvg=100.0 alpha=73.34 beta=2 prob=0.9986209383832925
ATGAATCAATCCCTTCATATATTTGCTTTTTATTGTACCAGTGCTGCGTTGGATGCCGCA
AAAACGATTTGGCGTGAAATAGCGGCAGATGAGACGAAAGCATCCTTTCAGAACAAATGA
>AA QryID: NC_000964_(-)_2435871_2436038 HitID: NC_017196_(-)_2236749_2236883
rank=1 bitscore=88.2 evalue=1.0E-15 perc_ident=97.73 perc_conserv=100.0 perc_gaps=0.0
query_cvg=80.0 subj_cvg=100.0 alpha=69.36 beta=1 prob=0.9986209383120702
TTGACCTCCTTCAATTTCACAAGCAGCTGGATAGTGCTGCTTTGTGAGGACCTGAATCAT
GAAAAATACAGTTCCGTTAGGTTAGACGGAGATAAAGAGATTCAGGTTTCATTTTTATTT
TTTAGTAAAGCTCGGGAGATTCGTTTTCCCCCGAAAGGTGTGGTATAA
>AA QryID: NC_000964_(-)_4166699_4166812 HitID: NC_017196_(-)_4049515_4049628
rank=1 bitscore=49.7 evalue=2.0E-4 perc_ident=97.3 perc_conserv=100.0 perc_gaps=0.0
query_cvg=100.0 subj_cvg=100.0 alpha=97.3 beta=2 prob=0.998620938037883
GTGATGGGCATTCTTTTCAAAGAATGTCTTTTCCTTTTTCATGTTTCACATGAAACATTA
GCGTCATTTTACTTGCGATTTGGACTTATGTTAAAATATAAAAGTAAAATTTAG
>AA QryID: NC_000964_(-)_2966284_2966406 HitID: NC_017195_(-)_2768241_2768363
rank=1 bitscore=47.4 evalue=0.001 perc_ident=100.0 perc_conserv=100.0 perc_gaps=0.0
query_cvg=100.0 subj_cvg=100.0 alpha=72.92 beta=1 prob=0.9986209376434203
TTGCGAACACAAAGGGAGCTGAAGCTAGAAAGCCATTATGCGCTTTTTAGCTTATGCTCC
TTTTATTTTTATAAAGAAACGAGAGTGTCAGCTTTTTCTTTTTTCATTTTTTTTGTAAAA
TAA
>AA QryID: NC_000964_(+)_1895052_1895165 HitID: NC_020244_(+)_1900917_1901054
rank=1 bitscore=63.5 evalue=2.0E-8 perc_ident=94.44 perc_conserv=94.44 perc_gaps=0.0
query_cvg=97.3 subj_cvg=80.0 alpha=83.72 beta=1 prob=0.9986209375911366
GTGGAACCGTATTTTGTCAGCGAACACTTCAAGAAGTGGGGAAGCTTAGGAATGCCAATG
GGTAAGATGTATGAAACCGCGCTAACGACGGTAGAGGGATCTCTTGATTTATGA
>AA QryID: NC_000964_(+)_3996412_3996540 HitID: NC_020244_(+)_3838259_3838387
rank=1 bitscore=48.1 evalue=7.0E-4 perc_ident=97.62 perc_conserv=100.0 perc_gaps=0.0
query_cvg=100.0 subj_cvg=100.0 alpha=77.9 beta=7 prob=0.9986209374556582
ATGACAAAACATGCAATCGCTGCCAAGAGTCTCTTGAAAAAATGGATCATTTCCAGGCCG
CTTAACATGAAAAAGAAATCCAGCCCATTTCAAAAAATCAGAAAAATGTTCAAACGCTTC
TTCAAATAA
>AA QryID: NC_000964_(+)_1533861_1533971 HitID: NC_014976_(+)_3744356_3744466
rank=1 bitscore=48.1 evalue=6.0E-4 perc_ident=97.22 perc_conserv=100.0 perc_gaps=0.0
query_cvg=100.0 subj_cvg=100.0 alpha=97.22 beta=2 prob=0.9986209372513766
TTGAATTTTTTGCTGCATTCTTGGATGAAAGTTCAAGAAAACGTGCAATTACATTATGTG

CTGTGGCATATATATCGAGTAAAGCTTCTGTATGAAGAAGATATTGTATGA
>AA QryID: NC_000964_(+)_3942101_3942229 HitID: NC_017743_(+)_3841580_3841708
rank=1 bitscore=57.4 evalue=1.0E-6 perc_ident=97.62 perc_conserv=97.62 perc_gaps=0.0
query_cvg=100.0 subj_cvg=100.0 alpha=64.78 beta=2 prob=0.9986209371840278
ATGCTCCGTCGTCGACGCGGGCATTTTTGTCATTTTCAGCCGGTATTTCTCACTTTTTTC
ATTTTGATTGTCAACTTTTCATTTGATACGATGAACAAGCCGACTTTTCTTCAGCAAAGG
AGGTTTTAA
>AA QryID: NC_000964_(-)_408912_409028 HitID: NC_017196_(-)_405244_405360
rank=1 bitscore=68.9 evalue=4.0E-10 perc_ident=97.37 perc_conserv=97.37 perc_gaps=0.0
query_cvg=100.0 subj_cvg=100.0 alpha=63.36 beta=1 prob=0.9986209366944745
ATGCAAGGGTTTGCCAACACGCTGAAACAGGGCGCCTCCGGCGGCCCTGTTTTTTTGTTG
AGCCCCTTTCGCCTATTCCGCCCTTCTGTCAGATGTGCTACAATGACAATCGACTGA
>AA QryID: NC_000964_(+)_3016411_3016593 HitID: NC_017743_(+)_2880746_2880937
rank=1 bitscore=80.9 evalue=2.0E-13 perc_ident=96.15 perc_conserv=96.15 perc_gaps=0.0
query_cvg=86.67 subj_cvg=82.54 alpha=59.08 beta=1 prob=0.9986209361852785
ATGTCGGCTTTTCAAGTCAACTTGCGTATTGATAACGCTTACAATAAGAAGTCGTTATTT
TCAGACACATTCACTATAGGTTTTGTATTGATGAACGCTTACAATTTGCGGTTTTTCCCT
CTGTATTTTGTGTTTCAAAGCTGGATTCAATTCGTCAAGTCCGGTCTTCAAAAGCATAAA
TAA
>AA QryID: NC_000964_(-)_3951940_3952053 HitID: NC_017743_(-)_3854065_3854178
rank=1 bitscore=60.1 evalue=2.0E-7 perc_ident=91.89 perc_conserv=94.59 perc_gaps=0.0
query_cvg=100.0 subj_cvg=100.0 alpha=91.89 beta=3 prob=0.9986209359125764
ATGCTCAAAAAGTTTCATTTTTTCCCTAATCTCCCTCAAATTAATATATCGGCGTATGTT
TTTGAAAATGAATCTTTATACCTGCAAGGGCAAGAATGGAATGTTTCGAGCTGA
>AA QryID: NC_000964_(+)_432090_432218 HitID: NC_017195_(+)_423438_423566
rank=1 bitscore=67.8 evalue=9.0E-10 perc_ident=95.24 perc_conserv=97.62 perc_gaps=0.0
query_cvg=100.0 subj_cvg=100.0 alpha=69.05 beta=2 prob=0.9986209358676108
TTGTATTTTTCACGAGGAATAATGTTAGGTTTGCTGACCGTATGCCTGCGTTATAAAGAT
AAATATGGTAAACAGCCTAACGTTTTGGGATGGAAAATGGTTAGAATGATTAGTAAAATT
GATAAATGA
>AA QryID: NC_000964_(+)_1422013_1422138 HitID: NC_020244_(+)_1425891_1426016
rank=1 bitscore=53.5 evalue=1.0E-5 perc_ident=100.0 perc_conserv=100.0 perc_gaps=0.0
query_cvg=100.0 subj_cvg=100.0 alpha=100.0 beta=15 prob=0.9986209357981493
TTGAAGCCGAGCCGCTCTGAGAAACTTGCCGTTTTTGCCGAACTGTTTGTGAAACTGCAT
ATCTTATCAAGCGCTCAAAAAGATGAAATCATCAATGTTAACAAAAAGAAACAAAAAAAC
CGCTGA
>AA QryID: NC_000964_(+)_1557919_1558029 HitID: NC_014976_(+)_3768415_3768525
rank=1 bitscore=43.5 evalue=0.016 perc_ident=100.0 perc_conserv=100.0 perc_gaps=0.0
query_cvg=100.0 subj_cvg=100.0 alpha=100.0 beta=1 prob=0.9986209357644825
GTGAGCCGCTCACTGATCATCAAGTTCGGCGAAGGCTGGCGATTGAAACGCATAAAGCGC
CAAAAAACAAGACATAAAAAAGACCAAAGCCGCCTAAGGCTTTGGTCTTGA
>AA QryID: NC_000964_(+)_2036875_2037015 HitID: NC_017195_(+)_2023622_2023753
rank=1 bitscore=54.3 evalue=2.0E-5 perc_ident=97.37 perc_conserv=97.37 perc_gaps=0.0
query_cvg=82.61 subj_cvg=88.37 alpha=83.24 beta=2 prob=0.9986209350912856
ATGTTATTCCTTTCATATAAAATAGTTTACATTTTTATAAAATCGCTTCACTTTTCTATT
TTGTCAGCAAAAAAACTGAACCACATTAAACTTATGAAACCTGCCGCACCTGTGGATTGC
AAATATGATATAAGCCCATAG
>AA QryID: NC_000964_(+)_1906790_1906975 HitID: NC_017196_(+)_1911627_1911812
rank=1 bitscore=79.7 evalue=5.0E-13 perc_ident=93.44 perc_conserv=93.44 perc_gaps=0.0
query_cvg=100.0 subj_cvg=100.0 alpha=78.0 beta=1 prob=0.9986209347740772
ATGCTAGAGCTGCTGTTGAACAGCGTGTCAGTCGCCAGTCTGATCTGTGAATGGGAGAAT
TCTGCATGCGCGAAAAAGAGAGAGAGGTGTATGTTGTCATTTCGTTCATTCTTGTTTGCA
GCCGCAACTGTTATGTTTGAACTGGGATTCCTGTTCAGCCAATTTATTTTGCGATTTTTG
TTTTAA
>AA QryID: NC_000964_(-)_2897372_2897494 HitID: NC_017196_(-)_2656389_2656511
rank=1 bitscore=44.7 evalue=0.008 perc_ident=97.5 perc_conserv=97.5 perc_gaps=0.0
query_cvg=100.0 subj_cvg=100.0 alpha=97.5 beta=2 prob=0.9986209347651056
ATGCTGTTGACACTGCGTCCAAAGCGGCGTAATATGAGTTCAACAAAAGATAAATGCAAG
CTTCACAAGCGAAAATCATCGCAGTATGATTCTAAAAAAATGAAAAACAAACGACCTTCT
TGA
>AA QryID: NC_000964_(-)_1035367_1035489 HitID: NC_019896_(+)_3039948_3040058

rank=1 bitscore=56.2 evalue=2.0E-6 perc_ident=97.22 perc_conserv=100.0 perc_gaps=0.0
query_cvg=90.0 subj_cvg=100.0 alpha=66.41 beta=1 prob=0.998620934435358З
TTGAGGTACGCGGTGTATTGTTGCCTAAGCAGAGGGCCCCATCATACCACCTCCAAAAGT
CAAAGTTTGATTATAAATTGTATATTTTATTGTAACACAACTCATTCCAAAAATGATTTT
TAA
>AA QryID: NC_000964_(+)_599463_599582 HitID: NC_017196_(+)_596546_596665
rank=1 bitscore=55.5 evalue=4.0E-6 perc_ident=92.31 perc_conserv=94.87 perc_gaps=0.0
query_cvg=100.0 subj_cvg=100.0 alpha=92.31 beta=2 prob=0.998620934304626
GTGAATGATCATCTTTTGAAGATGATCAACCTATCAGCAGAAATAAAGCCGCCTGATAAA
GAGGCGGCAGATCGTTATATGTCATCTATGAATTTGATGTATCGTTTTTCGTTTGCCTGA
>AA QryID: NC_000964_(-)_2316286_2316387 HitID: NC_018520_(-)_2295779_2295880
rank=1 bitscore=42.4 evalue=0.037 perc_ident=100.0 perc_conserv=100.0 perc_gaps=0.0
query_cvg=100.0 subj_cvg=100.0 alpha=100.0 beta=2 prob=0.9986209342007168
ATGGTGAAGCAAATGACAATGCTGTTTTTGCTTGGAGCGGTTTGTACATATAGTGTATTA
ATTGGTTTTGTATTAAAAGGAATCTCTAATAAATCTGTTTGA
>AA QryID: NC_000964_(-)_1233396_1233605 HitID: NC_017743_(-)_1281503_1281706
rank=1 bitscore=117.0 evalue=2.0E-24 perc_ident=92.54 perc_conserv=92.54 perc_gaps=0.0
query_cvg=97.1 subj_cvg=100.0 alpha=60.85 beta=2 prob=0.9986209340134753
GTGCATATGCTTGAGGCTGTTTTTTTATGCCATAACCAAACGTACGTTCCATTTTTAGCA
CTAAAGAAACCGCGCCGGGATAAGCGCGGTTTCATATGTCTTAACGACAACAAAGGGGAT
GGGAGAAATTTTTCACGGTCAAACAAAGGGGTAATGTTTGTTTGTGATCAATTTCACATC
CTTATGATAACAAAAGATGTCGACTTTTGA
>AA QryID: NC_000964_(-)_4004159_4004287 HitID: NC_017196_(-)_3867403_3867531
rank=1 bitscore=78.6 evalue=5.0E-13 perc_ident=92.86 perc_conserv=95.24 perc_gaps=0.0
query_cvg=100.0 subj_cvg=100.0 alpha=60.72 beta=4 prob=0.9986209334241773
ATGGTAGAGGAAAGCTCCGAAAAAAGGAGCTTTCCTTTTTTAATCGGAATAGTTGGCCCA
CGCTTCTCGTACATATGGAAAGGGGACGGAAACAGCCGATGCGGACAGGTTTTTGTAAAC
GCTTTCTAG
>AA QryID: NC_000964_(+)_1691008_1691133 HitID: NC_017743_(+)_1737638_1737763
rank=1 bitscore=58.9 evalue=4.0E-7 perc_ident=92.5 perc_conserv=92.5 perc_gaps=0.0
query_cvg=97.56 subj_cvg=97.56 alpha=90.24 beta=1 prob=0.998620933343616
TTGCATTTTTCTTCAAAAAGTTTCAAAAATGCCGAAAAGAAAGGAGAAAAAACAGAAATT
CTGCTATTTTCAGGCTTATATCAAGGCGAGAAATGTAGTTCTAACAATCTAGGACTTTAT
ACCTAG
>AA QryID: NC_000964_(+)_2136231_2136350 HitID: NC_017196_(+)_2073282_2073401
rank=1 bitscore=60.5 evalue=1.0E-7 perc_ident=94.87 perc_conserv=97.44 perc_gaps=0.0
query_cvg=100.0 subj_cvg=100.0 alpha=65.05 beta=2 prob=0.998620932876155
ATGATGAAACAAACTTCAGCATTGATTATAAAGGAATATATCGAAAAAAGAAATGTTCGA
GGATCTGTTAGGCACTTCCTCAGAAAAACGGATAATCGGGGTGTTAATTTGAAAACCTAA
>AA QryID: NC_000964_(-)_972961_973077 HitID: NC_017195_(-)_950687_950803
rank=1 bitscore=45.4 evalue=0.005 perc_ident=94.74 perc_conserv=97.37 perc_gaps=0.0
query_cvg=100.0 subj_cvg=100.0 alpha=94.74 beta=2 prob=0.998620931990654
ATGCTTTGGTTTCTTAAAAATAAAGATACTCATACATCGTTGACAAGGCAGACTGCGACC
GTTCATAATAAAGAAAAATACAGAATCATCCAGACTCGGACAGCACGGAAAAATTGA
>AA QryID: NC_000964_(+)_1105817_1105972 HitID: NC_017195_(+)_1084488_1084643
rank=1 bitscore=91.7 evalue=1.0E-16 perc_ident=92.16 perc_conserv=96.08 perc_gaps=0.0
query_cvg=100.0 subj_cvg=100.0 alpha=67.6 beta=2 prob=0.9986209318385183
ATGCTCCTGAGTCCCGGCAAATTCCTGTCGAAAAAATTCGTTCAAATGACCTGCGTGTGC
TTCCGTGAGAACAATGGATATTATCGTGATATTTTTTCAAAGCATGATGATAAAAGTATT
CTGAAAAATAAACTTTACAGAAAAGGGATAGAATGA
>AA QryID: NC_000964_(+)_3590381_3590503 HitID: NC_017196_(+)_3450895_3451017
rank=1 bitscore=51.6 evalue=6.0E-5 perc_ident=95.0 perc_conserv=95.0 perc_gaps=0.0
query_cvg=100.0 subj_cvg=100.0 alpha=73.41 beta=1 prob=0.9986209317179519
ATGCTCTATTCTGAGCAAATACGACAATATTCATGCTTTTTGTATCAATCGGTAAGATCG
ATTCATTTTCCATTTTTCACCCAAAATTGTCTTTACTTTCAGGCTGTCTGTTTTTATGAT
TAA
>AA QryID: NC_000964_(-)_3224699_3224812 HitID: NC_014976_(-)_1232066_1232164
rank=1 bitscore=56.2 evalue=3.0E-6 perc_ident=96.88 perc_conserv=96.88 perc_gaps=0.0
query_cvg=86.49 subj_cvg=100.0 alpha=58.87 beta=1 prob=0.9986209303677916
TTGGAGAATCTATACATGCATTCTCCATATGAGGGAAGCTGGCCTGCCCTCGCTTATATC
AGCTTCTTTCCCAGCACCTTGCAAAATAAAACCATCTGGTATGCTGATCGGTGA
>AA QryID: NC_000964_(-)_1784950_1785096 HitID: NC_014976_(-)_3995443_3995571

rank=1 bitscore=64.3 evalue=2.0E-8 perc_ident=92.5 perc_conserv=97.5 perc_gaps=0.0
query_cvg=83.33 subj_cvg=95.24 alpha=82.59 beta=1 prob=0.9986209298083941
TTGGATTTCTCATTTCCATGCATGTTGTTAAAGCACATAGCAGCGATTGAAATGAAATTA
ATAAATGGAAAAGACATATGTGAAAAAGTAAAGGAGATTCAACTTGGAAGGATTGCTATT
GGTGCTCTGGATGTGGTTCATTTATGA
>AA QryID: NC_000964_(-)_3302765_3303001 HitID: NC_017743_(-)_3167475_3167732
rank=1 bitscore=109.0 evalue=4.0E-22 perc_ident=90.14 perc_conserv=92.96 perc_gaps=0.0
query_cvg=91.03 subj_cvg=83.53 alpha=61.17 beta=2 prob=0.9986209281329571
ATGAAATGTTCGCCTTATATAATGTACTCTGTTTTATTTAGGATGTCAAACATTATGATT
TGCTGGCAGGTGTGGTGCAAGTGCCGAAGCTTGTCATGCAGGGTGAGTTTAGGTATCATA
GTTTTAACGAATGGTCTTAGAGAGCGGATCAAAACGCGAATGATATACGTTCAGCAAAGC
TTGGTCGTTCAGCACCAGGCTTTCTTTATGGCATGGACATGGGAAAGGAGCGAATAA
>AA QryID: NC_000964_(+)_2028845_2028976 HitID: NC_020244_(+)_2036101_2036232
rank=1 bitscore=48.5 evalue=5.0E-4 perc_ident=93.02 perc_conserv=100.0 perc_gaps=0.0
query_cvg=100.0 subj_cvg=100.0 alpha=96.51 beta=10 prob=0.998620926780577
GTGAAACCAATGAGTAAGAAAGGTATTCAAAATTCAAGTATTGAACAAATAAGAAATGAC
CATGAGACTGAAACAGCTTTTAAAGCAGATGATCCTAAAAAACACGGTTCCGATCCTAAA
AGAACGAAATAA
>AA QryID: NC_000964_(-)_3939715_3939843 HitID: NC_017195_(-)_3741412_3741546
rank=1 bitscore=61.6 evalue=6.0E-8 perc_ident=90.24 perc_conserv=92.68 perc_gaps=2.44
query_cvg=97.62 subj_cvg=90.91 alpha=86.09 beta=1 prob=0.9986209260545298
TTGATATATATATCGGAGAAAAAAAGAGAGATTAATAGTAAGAAGGAAAAAATTTTTCAT
GGGCAGGGTTGTTTAGCAAAATGCAGATTTTTTCCGCATGAAGAGGAAATTTTCCAAATG
AATTTGTAA
>AA QryID: NC_000964_(-)_568951_569082 HitID: NC_017196_(-)_564698_564826
rank=1 bitscore=60.1 evalue=2.0E-7 perc_ident=94.74 perc_conserv=94.74 perc_gaps=0.0
query_cvg=88.37 subj_cvg=90.48 alpha=52.28 beta=1 prob=0.9986209216441411
GTGGCATACAATGTTAGAAGCATTTCAGCAGTAAGTGAATGTTGTCCTAAGAAAAAAACAA
GCATCTTACTATGCTTGTTTTTCTTCTATAGAAATGATCTTATTACGCTATGATGCTATT
CCTTTAGCCTGA
>AA QryID: NC_000964_(+)_1905637_1905774 HitID: NC_017195_(+)_1887711_1887872
rank=1 bitscore=74.3 evalue=1.0E-11 perc_ident=91.3 perc_conserv=93.48 perc_gaps=2.17
query_cvg=100.0 subj_cvg=86.79 alpha=56.45 beta=2 prob=0.9986209198878979
TTGAACATTTTAAAACCAGCACAAAGACGGATTCATAAAACGGCTGCTGCTGAATATAAT
GTCCAATGCCACCTGCCTAAGAAGCAGGAGGCAGGCTGGATCAATTATGTAGCTCTGCCG
GAAATGATACAAACGTAG
>AA QryID: NC_000964_(+)_176746_176844 HitID: NC_014976_(+)_2391816_2391914
rank=1 bitscore=50.1 evalue=2.0E-4 perc_ident=93.75 perc_conserv=96.88 perc_gaps=0.0
query_cvg=100.0 subj_cvg=100.0 alpha=62.95 beta=1 prob=0.9986209187097611
ATGATGATAGGTTATTTTTTCGGAAACCAGCTCGGGTTGCCAAATGAGAGAAAAGATATT
ACTGTCATTAAAACGATTCTTGGTTTCATCCGGATATGA
>AA QryID: NC_000964_(+)_3658965_3659099 HitID: NC_017195_(+)_3457899_3458018
rank=1 bitscore=42.4 evalue=0.065 perc_ident=97.44 perc_conserv=100.0 perc_gaps=0.0
query_cvg=88.64 subj_cvg=100.0 alpha=91.9 beta=1 prob=0.9986209177564367
TTGGATGTAAATGGTATGTTAAGAAGTGTGAATTACGTGTTTTTTCTGATAAATGTTGTT
AATACTCTTTTACTATTTTATATACAAGTCCTATTTGTTTTAATCATTTCATATAAAAAA
GAGACAAATCTGTGA
>AA QryID: NC_000964_(+)_2778590_2778874 HitID: NC_019896_(-)_1448108_1448392
rank=1 bitscore=117.0 evalue=2.0E-24 perc_ident=85.11 perc_conserv=85.11 perc_gaps=0.0
query_cvg=100.0 subj_cvg=100.0 alpha=85.11 beta=1 prob=0.9986209082451467
ATGTTTTCTGCCCTCTTTCTAATGCCAATGATAAGGGAATACATACAGCCTTTGCTGCTG
CATTGCCATCAGCCTCTGCTGTCGGAATGGATTTCCATAACCATGTTTGAAGGAATGCGG
CATGGACGCATAACCATGGCCAATCGACGGCAGTCTTTTTTGATAATCGTTCATCATGCC
ATCACCTGCCTCACAATAACGTTGATTTACTATATGCAGGCGCGCATTTTCTTGACAGCT
ATTTCGCTAAACTTGGTCCTTCTCCATTGTATAATGCATCAATAG
>AA QryID: NC_000964_(-)_2712272_2712382 HitID: NC_014976_(-)_696136_696237
rank=1 bitscore=45.1 evalue=0.005 perc_ident=93.94 perc_conserv=93.94 perc_gaps=0.0
query_cvg=91.67 subj_cvg=100.0 alpha=60.6 beta=1 prob=0.9986209038509715
ATGGACGTTATGGGTTTAACAAAAGATGATTTTATGGAAGGAATCGAAGTAGGCGGGGCT
GTTACCTTTTTTGGTTTTACGAAAGATGCTGATGTATCCTTAACGTTTTAG
>AA QryID: NC_000964_(+)_624351_624467 HitID: NC_017195_(+)_613237_613353

rank=1 bitscore=72.4 evalue=3.0E-11 perc_ident=89.47 perc_conserv=94.74 perc_gaps=0.0
query_cvg=100.0 subj_cvg=100.0 alpha=67.46 beta=1 prob=0.998620896929479
TTGCCGCCTGACAGCGGCTTTTTTTATGTTTGGCTTTGCAAACAAAGGGGAATAGGACAA
ACTGCGGGAACGCTTTATGAGAATATCAGCGTATTTACATACCGTTCGGGGGTATAG
>AA QryID: NC_000964_(+)_368952_369131 HitID: NC_017195_(+)_359028_359207
rank=1 bitscore=90.1 evalue=3.0E-16 perc_ident=88.14 perc_conserv=93.22 perc_gaps=0.0
query_cvg=100.0 subj_cvg=100.0 alpha=61.04 beta=2 prob=0.9986208917611217
GTGTATACAAAAAGCATGGGTGGGTGTTTAAAAGCGTCTATGAAGGGAAGAGGTGATGAA
AAAAGGAATTGTCCAAGGATGAATGCCAGACGTACCAAGGAAACGCATGTGAAAGTGAAG
GATGCAGCAGACCCGTCACTCATCTATGCCAATACCGGAAGAAATGGTCAAGAGGCATGA
>AA QryID: NC_000964_(+)_101329_101448 HitID: NC_017743_(+)_101400_101504
rank=1 bitscore=40.8 evalue=0.11 perc_ident=94.12 perc_conserv=97.06 perc_gaps=0.0
query_cvg=87.18 subj_cvg=100.0 alpha=88.08 beta=1 prob=0.9986208814681174
TTGTGGACCGGGAAAATGGAAATAATGAAGGATAGAGCGAGAAAGTTGAAAATTCTCGAG
AAACGGCTTATAGTAAGATTAAAGTCAAATATAGTCAAAGTCAGTAAAGGAGGGGGTTGA
>AA QryID: NC_000964_(+)_1354131_1354277 HitID: NC_014479_(+)_1312684_1312830
rank=1 bitscore=77.4 evalue=2.0E-12 perc_ident=85.42 perc_conserv=87.5 perc_gaps=0.0
query_cvg=100.0 subj_cvg=100.0 alpha=85.42 beta=1 prob=0.9986208753837462
ATGCTGATTGGGAGAAATGGCTTAAAACCCTTTGCAAATATGGGTGATTGGTATGGAATG
GCCTCGGCAAAAATATTGTCTTTGTTTATTAATAGTTTATTCATATTTGCTTGTTACACT
ATTTCAAAGTTAGACAAAAAGGAGTGA
>AA QryID: NC_000964_(+)_859573_859704 HitID: NC_016047_(+)_971801_971932
rank=1 bitscore=67.8 evalue=8.0E-10 perc_ident=88.37 perc_conserv=88.37 perc_gaps=0.0
query_cvg=100.0 subj_cvg=100.0 alpha=66.26 beta=1 prob=0.9986208665637804
GTGTTTCTTTATGATTATTGTAAAAGGGAGAAAAATGAGTATTATGTAGCAGTATCAAAC
GACAAATACTGGGCATTTATGCTTATCGTTTGTCACAATCGCGGAATGTTTATTTTAGTG
TGGCTGGGGTAG
>AA QryID: NC_000964_(-)_1279267_1279479 HitID: NC_014479_(-)_1236388_1236615
rank=1 bitscore=88.2 evalue=1.0E-15 perc_ident=84.29 perc_conserv=92.86 perc_gaps=0.0
query_cvg=100.0 subj_cvg=93.33 alpha=81.48 beta=1 prob=0.998620862423736
TTGTTTGGAAAAACAGCATCTAATAGAAATGTCATGGCAAAAAACACAACCATGCAAATC
AAAAATCGTATAATCTCACTCAAAATCCAGCTATCAGCTATGCGCATACCATCGGTTTCT
CGGAGTGTTGCGCATATTGAAAAAATCATGAGTATGTTTTTTAGGGTTTCTTTGTTCATA
AAATACCATCGCTTTCTCGATAGGATGACTTAA
>AA QryID: NC_000964_(-)_339773_339886 HitID: NC_017195_(-)_329737_329850
rank=1 bitscore=58.9 evalue=4.0E-7 perc_ident=89.19 perc_conserv=91.89 perc_gaps=0.0
query_cvg=100.0 subj_cvg=100.0 alpha=57.79 beta=1 prob=0.9986208385779176
TTGTACAACATTTCTGCAGGTATTTTAATACTTCGACGCAAAAAAGCGTTAAAACCTTCC
GCCTTTATTGTTTTTCCAGCTGACTCTTCGATGGAAGGGTCTTTTTCTGTATGA
>AA QryID: NC_000964_(+)_320729_320887 HitID: NC_017743_(+)_315054_315212
rank=1 bitscore=63.9 evalue=2.0E-8 perc_ident=84.62 perc_conserv=86.54 perc_gaps=0.0
query_cvg=100.0 subj_cvg=100.0 alpha=84.62 beta=1 prob=0.9986208224037367
GTGTTGAAAAAGCAACTTATTTTTCCAGTAAAGGAGCAGGAAGTCATAAGAGGAAGGAGG
AATTCAATTCCAATAAAAGCGTTTTCAACTAAAGGTAGGAGAAAAATCACCCGTAAAATA
CAATCATATAGGAGGATTACAGAGCATTTAGAAGCATAA
>AA QryID: NC_000964_(+)_2488832_2488933 HitID: NC_014976_(+)_467989_468081
rank=1 bitscore=32.3 evalue=39.0 perc_ident=100.0 perc_conserv=100.0 perc_gaps=0.0
query_cvg=90.91 subj_cvg=100.0 alpha=95.45 beta=2 prob=0.9986207807474023
GTGAAAATCATGTCTCAAAAACTGATGAAAAGCGTTTTGATCGTGATTTTGGGAGTTTTT
ATTCTTTCAACTGTACTCTCAGGAATCGTCATGTTTCTATAA
>AA QryID: NC_000964_(+)_644379_644477 HitID: NC_016047_(+)_773329_773442
rank=1 bitscore=48.9 evalue=4.0E-4 perc_ident=83.87 perc_conserv=96.77 perc_gaps=0.0
query_cvg=96.88 subj_cvg=83.78 alpha=75.76 beta=2 prob=0.9986207510444594
TTGATGGATAAAGGGCTGAGGTCACTGTTAATAAAGTGGATAAATTCAGAATATTTTGTG
GATAGTGTGTATAAAGCGCTTACTTTGTTTATTTATTAG
>AA QryID: NC_000964_(-)_211194_211340 HitID: NC_016047_(-)_323597_323752
rank=1 bitscore=78.2 evalue=1.0E-12 perc_ident=86.05 perc_conserv=95.35 perc_gaps=0.0
query_cvg=89.58 subj_cvg=84.31 alpha=51.58 beta=1 prob=0.9986207312506677
ATGAAACTGAAGACAGCATATCAATTTTGTTCACCGCTTAAATTTATCAGCAAGCTTTAC
GAAGTTGGTCTCATAATCATGTCACTAAACACTTGGGGAAACCCGGCAGGTGACAAGGAT
GTTTTCAAATATTGTTCAAGTGACTAA
>AA QryID: NC_000964_(+)_202126_202266 HitID: NC_019896_(-)_3841518_3841658

rank=1 bitscore=68.6 evalue=8.0E-10 perc_ident=80.43 perc_conserv=82.61 perc_gaps=0.0
query_cvg=100.0 subj_cvg=100.0 alpha=80.43 beta=2 prob=0.9986205276058345
ATGTCCAAAAGCGAAAGAACGTCTACAGTGGAAAAATGGATTAAAGAAGTTGATGGTACA
GGACCCGACTATCGATCGACCATGGCTATAGATTTAAGATGTTTCCTCATTAGATCGGTC
AATCAGGTTAAAAGGCATTAA
>AA QryID: NC_000964_(-)_2778608_2778766 HitID: NC_017196_(-)_2535893_2536051
rank=1 bitscore=61.6 evalue=1.0E-7 perc_ident=75.0 perc_conserv=75.0 perc_gaps=0.0
query_cvg=100.0 subj_cvg=100.0 alpha=50.13 beta=11 prob=0.998619891337275
ATGATGAACGATTATCAAAAAAGACTGCCGTCGATTGGCCATGGTTATGCGTCCATGCCG
CATTCCTTCAAACATGGTTATGGAAATCCATTCCGACAGCAGAGGCTGATGGCAATGCAG
CAGCAAAGGCTGTATGTATTCCCTTATCATTGGCATTAG
>AA QryID: NC_000964_(-)_3700806_3700913 HitID: NC_017743_(-)_3577969_3578076
rank=1 bitscore=47.0 evalue=0.002 perc_ident=80.0 perc_conserv=85.71 perc_gaps=0.0
query_cvg=100.0 subj_cvg=100.0 alpha=80.0 beta=1 prob=0.9986198741288563
ATGCATATTAAATACAAACAGCAGGGTGTGACTATACGTCCAGAAGGATATCAGGAGAAA
AATGAAAGCGCATTCTCTTTGATTTTTTTAAAAAGTGATAAGGTATAA
>AA QryID: NC_000964_(-)_2720273_2720428 HitID: NC_017196_(-)_2483127_2483300
rank=1 bitscore=70.9 evalue=2.0E-10 perc_ident=80.7 perc_conserv=80.7 perc_gaps=15.79
query_cvg=94.12 subj_cvg=100.0 alpha=57.44 beta=2 prob=0.9986197125541261
TTGAAGACTATGGGAACCACTCTCCATACGGAAAAAGATGATTATTTACATCATTCAGAG
TTGATTTTAGGTTCTTTTGAACTTATAATGTTATATATACTGTTTTTTGTTTATTCAATA
AATATTTACATCGGAGGTGTACGCCTTACTAGGTAA
>AA QryID: NC_000964_(+)_613445_613618 HitID: NC_017195_(+)_602396_602569
rank=1 bitscore=81.6 evalue=1.0E-13 perc_ident=78.95 perc_conserv=78.95 perc_gaps=0.0
query_cvg=100.0 subj_cvg=100.0 alpha=57.21 beta=1 prob=0.9986186629100899
ATGATGCTGGTCACGATGATTCCCTGCATCATTCCCATTTATAGGTGTTCGGAATATCAC
TTTGCCGGCCCGTGCTCAAGCGGCTTTCACAGGCTGAAAGTTATGATACAATATACAAAT
GTTTCACAGCTTCAATCCATAGTTGGAGCTGACATCCATTATATAAGGGACTAG
>AA QryID: NC_000964_(-)_3036354_3036473 HitID: NC_017196_(-)_2799485_2799613
rank=1 bitscore=43.1 evalue=0.02 perc_ident=80.95 perc_conserv=88.1 perc_gaps=7.14
query_cvg=100.0 subj_cvg=100.0 alpha=87.18 beta=14 prob=0.9986182538635218
GTGAATCTCATGTGTACGATTGCAAAAGAACGGCTGCAGCGGGATCATTGGGAGCAGCAG
GCGCAAGATAGTGTTGGCCAGCAAGAGGCGAAAGCCGATAAAAAAACCCCGACCGCATAG
>AA QryID: NC_000964_(+)_3035508_3035609 HitID: NC_017188_(+)_2777773_2777868
rank=1 bitscore=42.4 evalue=0.037 perc_ident=80.65 perc_conserv=80.65 perc_gaps=0.0
query_cvg=93.94 subj_cvg=100.0 alpha=49.31 beta=3 prob=0.9986181668375754
ATGGTTGACTTCAAAACAAATAAATTATATAATGACCTTTGTGTGAAATATTGCAGCCTT
TTTGTTCAGCTTCTATGTTTTCATTTTGTTCCTTATCAATAA
>AA QryID: NC_000964_(+)_2354700_2354846 HitID: NC_016047_(+)_2321228_2321389
rank=1 bitscore=67.8 evalue=1.0E-9 perc_ident=79.55 perc_conserv=84.09 perc_gaps=0.0
query_cvg=91.67 subj_cvg=83.02 alpha=45.44 beta=1 prob=0.9986176194203762
ATGATCTGCCGTTTTCTTCTGCAAGCCAAAAAACCTTCCGTTACAACGAGAAGGATTCTT
CACTTTCTAAAGTTCGGCGAGTTTCATCCCTCTGTCCCAGTCCTTTTTTGGATCAAGGCA
GACTGCTGCAATGTCTATCTATTTTAA
>AA QryID: NC_000964_(+)_3532107_3532298 HitID: NC_014976_(+)_1545107_1545298
rank=1 bitscore=83.6 evalue=3.0E-14 perc_ident=69.84 perc_conserv=69.84 perc_gaps=0.0
query_cvg=100.0 subj_cvg=100.0 alpha=49.87 beta=11 prob=0.9986166309230213
ATGGGGAAAACAGGATACATTGGTGCTGCCATTGTTGTGGCAGCTTGTATCATTATTTTG
TCGGCCGTGGTATGTTTACGGGACACAGTCTATTACCAGCCTATGCGCTGGACTGGCATC
ATACTGTTTTTCGCGGGAATTGTGATGGTTCCCGCATACTCAGCAAAAAGAAAACCCGGA
AAAGAGAAGTAA
>AA QryID: NC_000964_(+)_2953633_2953791 HitID: NC_014976_(+)_962150_962308
rank=1 bitscore=57.8 evalue=2.0E-6 perc_ident=78.85 perc_conserv=84.62 perc_gaps=0.0
query_cvg=100.0 subj_cvg=100.0 alpha=53.85 beta=1 prob=0.998616012525819
ATGAGCGCAAGAGAGAAAAAGAAAATCTTTCATCCCCACCCAGAATACAAAGAACCAACA
ATTCCCCCTTATTACGCAATTTACAACAAAACCACCGACAGGAAGAAATGTAAAACAAAG
TTAATCTCTGACCGATATAAGGAGCAGAAGGGAGCCTGA
>AA QryID: NC_000964_(-)_3546001_3546183 HitID: NC_017195_(-)_3340288_3340434
rank=1 bitscore=69.7 evalue=5.0E-10 perc_ident=79.17 perc_conserv=85.42 perc_gaps=0.0
query_cvg=80.0 subj_cvg=100.0 alpha=47.53 beta=1 prob=0.9986143846121802
TTGGTCAATAAAGGTCAAACAAAAAGCTGGCCTGATATGCAAAGTCGTCTCTTTTTCCCA
TTTTCCCCAAAAATACAGGGGTTCAAACCATCGTATGTCAGATTGCCAATTAAGATGCTT

TGTCTATTTAAAAAACGGCCTCTCGAAATAGAGGGTTGTTATTTGAAAGGAATTATCGTA
TAA
>AA QryID: NC_000964_(-)_376767_376928 HitID: NC_017195_(-)_368303_368470
rank=1 bitscore=57.0 evalue=3.0E-6 perc_ident=78.26 perc_conserv=86.96 perc_gaps=0.0
query_cvg=86.79 subj_cvg=83.64 alpha=35.49 beta=1 prob=0.9986062364326919
GTGCAACGCATTTTCTCTTTCTTATCCATATCAGCTTTTAATTCTTATGAACTAACAGCC
GAAATAGCCCAAAGTACTACACTTAAAAAGCAGTGCTTCCAATCAAAAAACAGATGGCCG
CTATTAAAGCAGGCTGCCGTCAGTCAGCATTGCGGCGTTTAA
>AA QryID: NC_000964_(+)_2070028_2070129 HitID: NC_017196_(+)_2008261_2008374
rank=1 bitscore=38.5 evalue=0.52 perc_ident=76.67 perc_conserv=83.33 perc_gaps=0.0
query_cvg=90.91 subj_cvg=81.08 alpha=40.97 beta=1 prob=0.9985865057400618
TTGATGGGCAACTTTTTGTACAGAGCCGGGGTGTTGGTAGCACCTCGGTCTTTTTTATTT
TATGCATTTCTCATTAAAGAAATGCACTGTAATGTATTGTAA
>AA QryID: NC_000964_(+)_3404546_3404695 HitID: NC_017195_(+)_3202093_3202242
rank=1 bitscore=67.0 evalue=3.0E-9 perc_ident=73.47 perc_conserv=73.47 perc_gaps=0.0
query_cvg=100.0 subj_cvg=100.0 alpha=41.88 beta=3 prob=0.9985779518352697
GTGCTGATCAAACAGGCAGGTATCCTGGCTCTTGGCGCTTGGCCAATTACAGTGGCGGGA
CCGCACCGGCTTTTACCGGTTTCCCTTTTAAGCCGGCTGTTGTTCACGGACCGGCACCTA
TTTGACATTCGTATCCGTTTTTCGGAGTAA
>AA QryID: NC_000964_(-)_1493344_1493469 HitID: NC_017196_(-)_1498851_1498964
rank=1 bitscore=36.6 evalue=2.0 perc_ident=72.97 perc_conserv=72.97 perc_gaps=0.0
query_cvg=90.24 subj_cvg=100.0 alpha=69.41 beta=2 prob=0.9985726548289531
GTGTTTTTTAGCGTATCAATCAAACAAATCATCAATTCCTATACCCAAATTTGGAAAAAT
AAGCTTCTTACTCTTCTTCCATTGCTTTTTGCACAAATAAAAAACCTGATGAGGTCAGGT
TTTTAG
>AA QryID: NC_000964_(+)_1560268_1560381 HitID: NC_017195_(+)_1534750_1534863
rank=1 bitscore=43.1 evalue=0.022 perc_ident=67.57 perc_conserv=67.57 perc_gaps=0.0
query_cvg=100.0 subj_cvg=100.0 alpha=67.57 beta=7 prob=0.9985627004945132
ATGACAACTAGTAAATGCCGCCTTGACCTCTTGTTTAATCAGGCGGCTTTACTTTTATAC
ATAGATGGCATTTTGAGTAAAAAAACTCATAATTTAAGAGAAGAGGAAATGTAA
>AA QryID: NC_000964_(-)_2778456_2778617 HitID: NC_020244_(-)_2589962_2590096
rank=1 bitscore=46.2 evalue=0.005 perc_ident=68.18 perc_conserv=68.18 perc_gaps=0.0
query_cvg=83.02 subj_cvg=100.0 alpha=62.39 beta=3 prob=0.9982244917373558
TTGGCATTAGAAAGAGGGCAGAAAACATTATGTTTTCTGCTTTTTTTTCGGAAAAAAATG
AAAAAGTTTAGTTCTATTGAAACATTTTTCAATACATTGCCGTCTAGTTGGTACCTTCCT
TCACTTTTAGAAATGAAGATGATGTTTATTTTTTTAAGATAA
>AA QryID: NC_000964_(-)_1190234_1190407 HitID: NC_017195_(-)_1163316_1163468
rank=1 bitscore=68.6 evalue=9.0E-10 perc_ident=100.0 perc_conserv=100.0 perc_gaps=0.0
query_cvg=87.72 subj_cvg=100.0 alpha=68.19 beta=54 prob=0.9982178299233269
ATGGAAGGGGGCAGTCTCAAAATGGGTAAACGAAGCAAATCCAGAAGGTTTATTCAGCAG
GGTAAAGACTCCATCGGGCTACACGCCGCAAGATTTCCATATCGGTCTACTTTAGAAGAA
GCTCACCAGCATGCCGCGGCACGAGACTCGGCTGAAAGGCAGTATGAATATTAA
>AA QryID: NC_000964_(-)_527912_528025 HitID: NC_017743_(-)_524747_524860
rank=1 bitscore=54.7 evalue=7.0E-6 perc_ident=100.0 perc_conserv=100.0 perc_gaps=0.0
query_cvg=100.0 subj_cvg=100.0 alpha=100.0 beta=38 prob=0.9981739581084974
ATGCCAAATTGGCTTAAAAAGCAGATGCAAAAAGCGTTCCTTGAAAAAGATAATTACCAA
ATTAAACTGCTGAATCAATGCTGGTATTTCTATAGGAAAAAACACTGCTCGTAA
>AA QryID: NC_000964_(-)_1357602_1357796 HitID: NC_017743_(-)_1406480_1406656
rank=1 bitscore=57.4 evalue=2.0E-6 perc_ident=58.62 perc_conserv=63.79 perc_gaps=0.0
query_cvg=90.62 subj_cvg=100.0 alpha=58.78 beta=9 prob=0.9974362691541365
TTGCTAGGAGGCATGAACATGAAAACCGTGGGACTGGGTATGATCTGCGCAGGATTGGCT
TTTCTTTTGCTGAGCTTTCTTCTCTCTGAATCGACGTTGACGTGGGGAATTGTATTGGGT
ACGAGTATTCTATTAAACATAACTGGTACGGCTCTAATCATGCGTTTCTTAAAAGCCCCC
CAATATAACAGCTGA
>AA QryID: NC_000964_(-)_1900640_1900813 HitID: NC_016047_(-)_2001986_2002159
rank=1 bitscore=60.5 evalue=3.0E-7 perc_ident=61.4 perc_conserv=64.91 perc_gaps=0.0
query_cvg=100.0 subj_cvg=100.0 alpha=61.4 beta=4 prob=0.9973763100094993
ATGTATATAAGTATTATAATTTTAGCATCCATGCTTTTATGGGGGGCAGTTATCCACGAG
CTAAGTAAACCTTCAAAAAAGCAAAGCACCAGAAAAATTGTTTCTTTGACATCTTTAGGT
TCTTTATCGACTTTAGTTATTACTATTTCACTTTTTCAAAACTTCCCTTTTTAA
>AA QryID: NC_000964_(-)_154695_154865 HitID: NC_017196_(-)_154905_155075

rank=1 bitscore=54.3 evalue=2.0E-5 perc_ident=64.29 perc_conserv=66.07 perc_gaps=3.57
query_cvg=100.0 subj_cvg=96.43 alpha=42.82 beta=1 prob=0.996996814779757
TTGAATAAGAATGAAATCTACCATGATTATTACAAAGAGACCGTTTATAAACAAACAATT
TGCTTAACCCCGTCAAGCGTCCATTCAGTTGTTATTTTTATGCGGGACTGCCACGTTTTA
TTGAAAGAAAAAAGCTCCCAACCCAAGGTCGAGAGCTTTTTGAAAACGTAA
>AA QryID: NC_000964_(-)_2333095_2333223 HitID: NC_017195_(-)_2178191_2178319
rank=1 bitscore=40.0 evalue=0.2 perc_ident=61.9 perc_conserv=61.9 perc_gaps=0.0
query_cvg=100.0 subj_cvg=100.0 alpha=61.9 beta=5 prob=0.9969076357241591
ATGACGTTAGTTATTTTTAAAGGGAGAGTTGGTATGTATAAGGGTGTTTTTTACTTGTTC
TTTGTTGTGGTATTTTTGATTGCTTTTGTGTTTTCAAACTTTAAAGACAGCTTAATTGCA
CACTTTTAA
>AA QryID: NC_000964_(+)_3941890_3942006 HitID: NC_020244_(+)_3783248_3783364
rank=1 bitscore=38.5 evalue=0.55 perc_ident=63.16 perc_conserv=63.16 perc_gaps=0.0
query_cvg=100.0 subj_cvg=100.0 alpha=63.16 beta=2 prob=0.9968169810001837
TTGTACAGGCTTTTCATACTATTGCTATACAGCCATGAACAGCATAAAATGAACGTTATT
ACAGTTATCACCACATATGGCGGGATTGTGACTGGGCAGGCAGGCAAGACCCAATGA
>AA QryID: NC_000964_(-)_2779220_2779336 HitID: NC_014639_(-)_2196832_2196957
rank=1 bitscore=38.5 evalue=0.54 perc_ident=55.0 perc_conserv=67.5 perc_gaps=7.5
query_cvg=97.37 subj_cvg=97.56 alpha=27.98 beta=1 prob=0.9952296816234919
ATGACAATGAGACAACGACGTCAACCTGTACGTGATACACATGTAAAAGTTCATGGTTTA
TTCAGCCTGTGGAAGAGTTCGTGTCCTTCCTTAACCGATGCTTATCGCACGCATTAA
>AA QryID: NC_000964_(+)_2002370_2002504 HitID: NC_017743_(+)_2054792_2054908
rank=1 bitscore=37.0 evalue=2.8 perc_ident=60.53 perc_conserv=60.53 perc_gaps=0.0
query_cvg=86.36 subj_cvg=100.0 alpha=56.4 beta=1 prob=0.9928896050011202
TTGAAATCGTTTTTAAACATGCCCTCATTTTTTGACAAAAACAGGATATGTAACATGTTA
TTATTCATCACTGCACTTATGAAACCCTTCCCGATTCTTCTAATTTTACACATCTTTTTA
GCTTATGAAGAATAA
>AA QryID: NC_000964_(+)_320906_320998 HitID: NC_017188_(+)_269903_270013
rank=1 bitscore=33.5 evalue=17.0 perc_ident=63.33 perc_conserv=80.0 perc_gaps=0.0
query_cvg=100.0 subj_cvg=83.33 alpha=58.06 beta=1 prob=0.991956862581278
ATGGATGTTTATAAAGAAATGGTACAGAATAAAAGAGAATATGCTGTTTGTGTGGGAAGT
TACATAAATGTTACGGTAATAAAGATTGCTTAA
>AA QryID: NC_000964_(+)_1252611_1252727 HitID: NC_014976_(+)_3463518_3463634
rank=1 bitscore=30.0 evalue=212.0 perc_ident=57.89 perc_conserv=57.89 perc_gaps=0.0
query_cvg=100.0 subj_cvg=100.0 alpha=57.89 beta=1 prob=0.947301527621885
ATGAATCAGCATCATAAACCTTCCCAAAAATCTCCATATACAGATTCATCGTATCAATCA
AAAAAGATAAAAAAGAAAGGCTGCGGGTGCGGAAATAAAAAGAAAAAGCCTCAATAG

- ***Caulobacter crescentus* CB15 uid57891**

No vsORFs

sORFs

>AA QryID: NC_002696_(+)_1948167_1948394 HitID: NC_011916_(+)_1974143_1974370
rank=1 bitscore=122.0 evalue=4.0E-26 perc_ident=100.0 perc_conserv=100.0 perc_gaps=0.0
query_cvg=100.0 subj_cvg=100.0 alpha=83.14 beta=40 prob=0.9999618469937064
ATGACCGCATTCTCTCCCGCCACCATGCCCGCCACGGACGACGACAAGCGCGCCGTCGAA
CGGCTCGCCGAGGCCTCCCACCGCCTGAACGACGCCGTGCGTCGCGCCATCGCCGCCGGC
TATTCGGTGGAGCTGGTGCGCACCTCGCGCCATCATGACGGCGCAGGCAACTGGGGCGAT
CAGATCGTCCCGACCGTTCGTGAAAAAGGCGCCGAAAAGAGCGCCTAG
>AA QryID: NC_002696_(+)_513706_513996 HitID: NC_011916_(+)_539769_540026
rank=1 bitscore=82.4 evalue=8.0E-14 perc_ident=65.88 perc_conserv=65.88 perc_gaps=0.0
query_cvg=88.54 subj_cvg=100.0 alpha=39.88 beta=34 prob=0.9999618432096337
ATGCGCGCGCCCATTCGCGGGCATGGATTACCGATGCACAGCCAGAACCCTTTCCTCGAC
GAATTCGCCAAGCTGACCCAGGCGGCCATGGGCATCGCCCAGACGGCCGGCGAGGAGGCC
AAGACCGCGATGCGGGCCCAGGCGGACCGTCTGGCGGCCGAGTTCGACCTGATCCGTCGC
GACGATTTCGAGGCCCTGAAGGCCGAGGTCGCGGCGCTGCGCGAGGAAGTGGCGGCCTTG
AAGGCGGCCAAGAAGGCGCCCGCCAAGAAAACCGCGAGTTCAGGCGAATAG
>AA QryID: NC_002696_(-)_1205285_1205545 HitID: NC_011916_(-)_1231262_1231522

rank=1 bitscore=171.0 evalue=9.0E-41 perc_ident=100.0 perc_conserv=100.0 perc_gaps=0.0
query_cvg=100.0 subj_cvg=100.0 alpha=72.57 beta=1 prob=0.9999617690031452
GTGGTTAAGAAGTGTTTACAGCGCGTTAACCATAAATATTTCCTTTGTTTTTACACCGCG
GTCGAGATCCCAACCATTATGGTTGAAGGCGTATTGAATTGGCACATTTCTTGCTTTGTC
GAATACGCGCACGGCGGCCCCGCGCCCGCCGTTCTTAGAACTTTGTTCACCGACCTTAAA
ATGACGTTAACTCTAAGTTTTAGGCGTCCGTTCACCCTTCATTCCACCCCACCCTCCCCC
AAACGGCGCGAAGCAGGCTAG
>AA QryID: NC_002696_(+)_1722766_1723059 HitID: NC_011916_(+)_1748742_1749035
rank=1 bitscore=153.0 evalue=4.0E-35 perc_ident=88.66 perc_conserv=88.66 perc_gaps=0.0
query_cvg=100.0 subj_cvg=100.0 alpha=67.01 beta=1 prob=0.9999617651431557
GTGGCCGAGTACATCCTGCTGATGCATGACGACGGCGGGGAGGAGCGGGCGGCCGACTGG
GAGGCCTATCTGGATGGGCTGGCCGGCGCGGGCCGCCTGCGCGGCGGCAGCGCCATGGGC
GAAGGCGCCTGTTACCGGAAGGTCGGCGCTCCAGGGCCGGTCTCCGGGCATGTGACGGGC
TTTGTCCGCATTGTCGCCGAAAGCCTCGAAGACGCGGCGCGTTGTCTGGCCGGCAACCCG
GTCTATGAGGCCGGCGGTACGGTGGAAATTCGCCTGTTGCCCGAGGACGTTTGA
>AA QryID: NC_002696_(+)_3458076_3458342 HitID: NC_011916_(+)_3484065_3484331
rank=1 bitscore=152.0 evalue=6.0E-35 perc_ident=100.0 perc_conserv=100.0 perc_gaps=0.0
query_cvg=100.0 subj_cvg=100.0 alpha=82.75 beta=2 prob=0.999961755029225
GTGTGCCTTTCGGCCGGGGCGCTTCAGGTCCTTGTCTTTGTTTTTCGGCTTTTCAAGGCT
CGGCTAAGAGACCCGATAAACACGAAACTCGCCGGAGCGCGGGAAGCACACCGGCGGGTT
CAAGATCCCTTCGGACCCGAAAGCCCGGAAGGAACGCAAGCTCATCAGGACGGACGCGAA
CGACCGCCTCGAAAAAGAGCGCGGTATTTACTCGCGAAGTGGTCCAGCGTCAACCGCCTG
TTCCCGCGTCGGTTAACGCCCTCTTAA
>AA QryID: NC_002696_(-)_3624608_3624829 HitID: NC_011916_(-)_3650597_3650818
rank=1 bitscore=130.0 evalue=2.0E-28 perc_ident=100.0 perc_conserv=100.0 perc_gaps=0.0
query_cvg=100.0 subj_cvg=100.0 alpha=76.66 beta=3 prob=0.9999617437688421
TTGCGCGGGCGGCCCGGCGGTGTGAAGCTTCCTCCCACGGCAGCCTGGGCAAAAGATCCG
GGCGCCGACGAGGAAACGAGAGAGATGACGCCCCAAGACGTTGTCAGCCTGGCCCCGCCG
CGCGGCTTGCTGTTGATGCTGGCCCTGGCCTTCACCAGCATGATGGCCTGTTTCTGGACG
CATGGGGCGGACCTGGAAGCCGAGCTCGTCCGGCGCGACTGA
>AA QryID: NC_002696_(+)_2969289_2969528 HitID: NC_011916_(+)_2995269_2995508
rank=1 bitscore=135.0 evalue=8.0E-30 perc_ident=100.0 perc_conserv=100.0 perc_gaps=0.0
query_cvg=100.0 subj_cvg=100.0 alpha=70.77 beta=3 prob=0.999961743542296
ATGGTTCAGCTTTCACGTGAGGGGACGACGACGCTCCGCACCTGCGGCGGACGAATGGGT
CCCGCGCATGAAGCGCGGGATGACGGCGGAGTGGAGTTTTGGAATGGGGGGAGGTCGGAC
GCCCCCTCCGTCTCGCTGCGTATCCGCAGCGATCCACCTCCCCCGCAAGCGGGGCAGGAG
GGCTGCGGCTTCCTCCTCACCCGCAGCGCGGGGGAGGTGGCGTGCGGCAAAGCCGCGTGA
>AA QryID: NC_002696_(+)_1186395_1186613 HitID: NC_011916_(+)_1212372_1212590
rank=1 bitscore=120.0 evalue=2.0E-25 perc_ident=100.0 perc_conserv=100.0 perc_gaps=0.0
query_cvg=100.0 subj_cvg=100.0 alpha=69.34 beta=6 prob=0.9999617434197152
GTGCGCGTCACCTGGACCGATAGCGCATCGCGCGATCTGAGGTTCGCGTACGCCTGGATT
GCGCAGGATCGGCCTACGGCGGCGCTTAAGCAGGTTCGCAGAATTTGGGAGTTCGCGGCA
AAGCTGTCCGACTTTCCCAATCTTGGAAGGCCAGGACGTCGCCCTGGTACGCGTAACTGG
TCGTGCCGGGCGGCCCCTTCATTGTCGCTTACAGAGTGA
>AA QryID: NC_002696_(+)_2910412_2910666 HitID: NC_011916_(+)_2936392_2936646
rank=1 bitscore=142.0 evalue=4.0E-32 perc_ident=100.0 perc_conserv=100.0 perc_gaps=0.0
query_cvg=100.0 subj_cvg=100.0 alpha=72.62 beta=1 prob=0.9999617432595498
TTGCTCACATGGCGCACCGCCACGCTCGTTGATCGGCCCACTGGCCACCTATACGTCGCC
GACAGGTTGGCGGGGTTTTTCGGCCGACCGCGCGCGTCTGTGAGCGCTGGTGCGCGCCTC
TGCGCCTTGGTCGCTTGGACCAGGCGGCGGCACAAGCCGAACCAAGCCCTGGGCAAGGTC
GCGGCTCGCACAACCCAAAACATCACCCCGTTTAAACTATCGCTTGAATTGACTTCCTCC
ACTCGCGGTTCATAG
>AA QryID: NC_002696_(-)_197070_197267 HitID: NC_011916_(-)_197070_197267
rank=1 bitscore=102.0 evalue=5.0E-20 perc_ident=100.0 perc_conserv=100.0 perc_gaps=0.0
query_cvg=100.0 subj_cvg=100.0 alpha=100.0 beta=1 prob=0.9999617404367486
TTGGAGGGTTCCATCCAAACCAGACGGCTCCAAGCCCCGGGCCTGCCGGGGATCATAGGC
TATCGGCCGGGCATGGGTGGCGCGCGCGCACAGACCCATGACCCGGCGCGGCCGATCGGC
GAGCACGCCCATCGAGTCAGCCACCGCTGCGTGACTCCCAATCCCGATGCGACGAAAACT
GGGCCTCGCATCGCTTAG
>AA QryID: NC_002696_(-)_2654495_2654698 HitID: NC_011916_(-)_2680477_2680680
rank=1 bitscore=79.7 evalue=4.0E-13 perc_ident=100.0 perc_conserv=100.0 perc_gaps=0.0
query_cvg=100.0 subj_cvg=100.0 alpha=100.0 beta=3 prob=0.9999617378372871

TTGCTGACCGAGTGCGCAACAGATGAGCCGCCTCGAAGGACGCATAGTGCTAGCCCTGCG
GAGATCCGACCTCCCCGGCGAATGCCGGGGTCCAGATCGAACCCACCGACTATCGTCGGC
TTAGCGCGGAGCGTTGGCGCGTCATCCCCAGCCGCTCCGGATGAATCTGGACCCCGGCGT
TCGCCGGGGAGGTCGGGGTGTTAG
>AA QryID: NC_002696_(+)_2302707_2302961 HitID: NC_011916_(+)_2328683_2328937
rank=1 bitscore=106.0 evalue=5.0E-21 perc_ident=100.0 perc_conserv=100.0 perc_gaps=0.0
query_cvg=100.0 subj_cvg=100.0 alpha=82.83 beta=2 prob=0.9999617369337677
TTGTCTCACAATCCGGACGCTGTCCGGCGCTCCGCTTCCCTTTCCGAACCTTCAGCGGGA
GCCCGCGTGAAGTGGTGGTGGCGTGCTCAGAGCGCCCCCTCCGTCTCGTCGCGTATCCGC
GCCGATCCACCTCCCCCGCGCGCGGGGGAGGATGACAGGCCGACCTCCTCACCCGTGCAG
CGGGGGAGGTGGCGCGATGCGAAGCATCGCGACGGAGGGGGCGCTCATCAAATCCAGACA
GGAGCCTCCCCTTGA
>AA QryID: NC_002696_(-)_2230563_2230784 HitID: NC_011916_(-)_2256539_2256760
rank=1 bitscore=140.0 evalue=2.0E-31 perc_ident=100.0 perc_conserv=100.0 perc_gaps=0.0
query_cvg=100.0 subj_cvg=100.0 alpha=60.02 beta=1 prob=0.9999617365985606
TTGCGCGCAAAGATTGTGATCTGGCCTCTGAAAGGGGCGTCAGATGCCGTCTTGCAGCGT
TTACGAGGGGTGCGCGCAGGTTCCGAGCGCCGGGTTTGTTTCTCGCCTGAAACATCGTGT
CGTGTTGCACACTCGCACATATCGAATATGTCTATTTTTCAAAGCCTAGGCCGCATTTGG
CGCGGATTGGGACACACCCCTGTGTGCTTTCTGCTGCAATAG
>AA QryID: NC_002696_(+)_3461706_3461933 HitID: NC_011916_(+)_3487695_3487922
rank=1 bitscore=88.6 evalue=1.0E-15 perc_ident=100.0 perc_conserv=100.0 perc_gaps=0.0
query_cvg=100.0 subj_cvg=100.0 alpha=100.0 beta=1 prob=0.9999617356499785
TTGGACGGCTGGGCGGGATCAAAGACGCAAGCCGGGCGTTGTGGGTCCATGCCGAGCCTA
TCAAACCCGCCCACCGAGGAGCGCGCCTCGAATCGCGAGGTTGAGCACGAGACCATGCGC
CCCGCCCGCGAGGACGAAGCGCCTTGCCCGGCGACTGAGCCGCCGGGATCGGCAGACAGC
GTCCGCTCGCCGAAGACCCGGACGGATCCGGGCTCCAGCGAAAGCTAG
>AA QryID: NC_002696_(-)_615970_616203 HitID: NC_011916_(-)_642000_642233
rank=1 bitscore=116.0 evalue=3.0E-24 perc_ident=100.0 perc_conserv=100.0 perc_gaps=0.0
query_cvg=100.0 subj_cvg=100.0 alpha=76.82 beta=1 prob=0.9999617348022812
ATGACCCGCGCATCTCTGGCCAGCCTACGGCGAGGCGCGTCGCCGAAGCGCTGGCCAGAC
TTGGACGCCCGACCACCTGAACGAAACAGGAGGCGTTTCCAGTATTCGCCAACCTCTTCG
GAGGTTCAGGCGCTGTCGACCCTGAGGCCAGCGCTGGATGGTCCCTACGGTTTGTGGATC
GCGCCGGGCGAGATGAACGCTATCCCGGCGCCGTCGCGCTCAATTGACGTCTGA
>AA QryID: NC_002696_(+)_361909_362088 HitID: NC_011916_(+)_361909_362088
rank=1 bitscore=110.0 evalue=1.0E-22 perc_ident=100.0 perc_conserv=100.0 perc_gaps=0.0
query_cvg=100.0 subj_cvg=100.0 alpha=80.65 beta=1 prob=0.9999617341487228
TTGGATCCCTGGGGGGCGGTCGCGCGTCCACAGAAAAAGCTGATCACATTGGATGTGCAT
GCGTCGTGGCGGCCGCCCTCGCCCTTCGACAAGCTCAGGGTGAGGACGACTGTTGGCCTC
GGTTCGCACAAAATCCTCATCCTGAGCTTGTCGAAGGACGAGGATTTCCAATCCGCGTGA
>AA QryID: NC_002696_(+)_271669_271875 HitID: NC_011916_(+)_271669_271875
rank=1 bitscore=112.0 evalue=8.0E-23 perc_ident=100.0 perc_conserv=100.0 perc_gaps=0.0
query_cvg=100.0 subj_cvg=100.0 alpha=77.26 beta=1 prob=0.9999617328308107
GTGGCAGCTGGAGGGGCGTGGCATTGGCTTTGCCGCCTTCTTCTCGTCCCCGAGAAGGAG
CCCTCCTTACATCTCACGGCGAGACGATCGCAGGGCTGGGCAGGTCACTCAAGGACGACG
CTCCGCGTCGCCGCCGCGCGGCCGTCCGCAGGGCGGTCCCTGACTGACCTGCCCAGCCCT
GCACGCTGCAGCCTCCAAGAGATTTAA
>AA QryID: NC_002696_(+)_1225215_1225391 HitID: NC_011916_(+)_1251192_1251368
rank=1 bitscore=70.5 evalue=3.0E-10 perc_ident=100.0 perc_conserv=100.0 perc_gaps=0.0
query_cvg=100.0 subj_cvg=100.0 alpha=100.0 beta=2 prob=0.9999617289001757
GTGCGCGCAGGACTCCAAAGTCGTCCTCACCCTGAGCCTGTCGAAGGGCGAGGGCGGCTC
CCACGATCTCATCAACACTCAACGCGAAACAGCCAAAGAAAAAGGGCCGAGGATCGCCCC
GGCCCTTCGTTGTTTCAGACGGTGAGCGAGCAGGCCGCCCACCACCGTGAGTGTTGA
>AA QryID: NC_002696_(-)_3408850_3409053 HitID: NC_011916_(-)_3434839_3435042
rank=1 bitscore=108.0 evalue=7.0E-22 perc_ident=100.0 perc_conserv=100.0 perc_gaps=0.0
query_cvg=100.0 subj_cvg=100.0 alpha=72.5 beta=1 prob=0.9999617249812318
TTGGCGTCAAACATCGCCGCCACGGTCGCGCTGGGCGTGCCCGGCGCGATGGGAGCGAGC
GGTTCGATCAGACGGGACAGCGTTTCCATACGGCGCTTCGTCAACGGCTTGGGGTTTGCA
ACTTGCCTCGGCCGCCCTTGCAGAGTCGTTAAGGTGGTACCGCTCTCATTGTCGCGGCCC
GAGGCGGCGGGGCTTCAAGATTGA
>AA QryID: NC_002696_(-)_489255_489374 HitID: NC_011916_(-)_515285_515404
rank=1 bitscore=50.8 evalue=1.0E-4 perc_ident=100.0 perc_conserv=100.0 perc_gaps=0.0
query_cvg=100.0 subj_cvg=100.0 alpha=100.0 beta=4 prob=0.9999617237537175

ATGCCGGGATCCAGATCGAACCCACTGACGCTTATCCTTTCAGCGCGGACCGAGGGCGCG
TCATCCCCAGCCGTTCTGGATGAATCTGGACCCCGGCATGCGCCGGGGAGGTCGGTATAG
>AA QryID: NC_002696_(+)_1459904_1460059 HitID: NC_011916_(+)_1485880_1486035
rank=1 bitscore=92.4 evalue=5.0E-17 perc_ident=100.0 perc_conserv=100.0 perc_gaps=0.0
query_cvg=100.0 subj_cvg=100.0 alpha=78.23 beta=1 prob=0.9999617148498033
GTGTTGAAGCCGCCTGCGTCATGGCGACGCGGGCGGTTTTTTTCATGCGCCATGCTCCTG
AACCATTCGCCTCAGGATCAGCTGACGCGGGTTTCATCGCCCGCCAAGCACGACAAGGCG
TTCGCCCATATAGGCCGTCTACGGATTAAAAGCTGA
>AA QryID: NC_002696_(+)_1024154_1024339 HitID: NC_011916_(+)_1050185_1050370
rank=1 bitscore=85.5 evalue=8.0E-15 perc_ident=100.0 perc_conserv=100.0 perc_gaps=0.0
query_cvg=100.0 subj_cvg=100.0 alpha=77.96 beta=2 prob=0.999961714729455
TTGCGCTGGGGTTCTCCAGTACCCGAGGGAGAACCGGCCATGGCGGCGGACGCGGCGATT
TTTGTATCAAAACGGCTCCAGCGCGCGGCCTTCGCCATCCTGTGTATCATGCTGGCCCAG
TTCGTCCTCGCCAGCTGCGAGGCCGAACCGACGCCGATGGCGGCGCGCGAGTCCGCCGCT
TTCTAG
>AA QryID: NC_002696_(-)_2029331_2029450 HitID: NC_011916_(-)_2055307_2055426
rank=1 bitscore=64.3 evalue=1.0E-8 perc_ident=100.0 perc_conserv=100.0 perc_gaps=0.0
query_cvg=100.0 subj_cvg=100.0 alpha=100.0 beta=1 prob=0.999961714015798
ATGGTCTTCTCAGCTTGGCCAGTAGCGACCGCCGCGCGCGGCAAGGCCTTCGCGGAGAAT
AGACCAGACGCTCGCCATCAGTCTGGACAGCATGTTGATGCGCTCCGCCTCTTGGCCTGA
>AA QryID: NC_002696_(+)_2948881_2948994 HitID: NC_011916_(+)_2974861_2974974
rank=1 bitscore=62.8 evalue=2.0E-8 perc_ident=100.0 perc_conserv=100.0 perc_gaps=0.0
query_cvg=100.0 subj_cvg=100.0 alpha=100.0 beta=1 prob=0.9999617116166721
TTGTTCAGCGAGGCGCGCCAGAGCGCCTTTGTCGCCCTCAATCCGCTGTTTCGGATTTTC
GCCTTAGTTTTCCGGAATATCGGAAATTGTCCGAAACATCGGATGAATAGGTAG
>AA QryID: NC_002696_(+)_437383_437550 HitID: NC_011916_(+)_437383_437550
rank=1 bitscore=62.4 evalue=6.0E-8 perc_ident=100.0 perc_conserv=100.0 perc_gaps=0.0
query_cvg=100.0 subj_cvg=100.0 alpha=100.0 beta=1 prob=0.9999617109444994
GTGTCATCCCGGCCGCAGCGCGCAGCGCGGAGCGCCGGGACCCAGGGGGTGACCAAGCGC
GGCGCTCTTGCAACCCCTGGGTCCCGGGTCGGCTTCGCCGCCCGGGAGGACATGAGCTTA
CGGGGATTGGGCGGCGTGAACGCTCGGAATTGGCGGGGGCGGGCCTAA
>AA QryID: NC_002696_(+)_2278974_2279204 HitID: NC_011916_(+)_2304950_2305180
rank=1 bitscore=97.1 evalue=3.0E-18 perc_ident=100.0 perc_conserv=100.0 perc_gaps=0.0
query_cvg=80.26 subj_cvg=80.26 alpha=57.44 beta=2 prob=0.9999617026454743
ATGCGACCTCCCCGGCGAATGCCGGGGCCCAGATCGAACCCACGGACGCCAATCTGGCAA
GCGCCGCACGATATCGGATCATCCCCAGCCGCTCCGGATGCATCTGGGCCCCGGCATTCG
CCGGGGAGGTCGGTTAATTTGAAGCATCCGTCTGGGGTGAGCTTCTACCGCAATGCGTCC
TTCGAGGCTCGCCTTCGGCTCACACCTCAGGATGAGGAAATCCGTTGCTGA
>AA QryID: NC_002696_(-)_2971285_2971503 HitID: NC_011916_(-)_2997265_2997483
rank=1 bitscore=102.0 evalue=8.0E-20 perc_ident=83.33 perc_conserv=83.33 perc_gaps=0.0
query_cvg=100.0 subj_cvg=100.0 alpha=83.33 beta=1 prob=0.9999616856716493
ATGAACAAGCCTGCCAAGCCTGCGGCGGATGACGTCGACGACCTCTTTGGCCGCCCGCTG
ACGCCGGCCGAAGAGGACACATGGTTCGAACATAATCGCGAAGCCATCGGTCAACTCGTC
GATGAGGCTTGGGCTGAGTTCGAGCGTGGCGAATATGACGAACGCAGCTTCGCTGAGATC
ATCGCGCAGGGCGTTGCAGAGCATAACGCCAAACGCTGA
>AA QryID: NC_002696_(+)_1897353_1897460 HitID: NC_011916_(+)_1923329_1923436
rank=1 bitscore=45.4 evalue=0.004 perc_ident=100.0 perc_conserv=100.0 perc_gaps=0.0
query_cvg=100.0 subj_cvg=100.0 alpha=100.0 beta=1 prob=0.9999616632451411
ATGGCTTCGGCGGCGCGGTATCTAGATGCGGATGAAACCATCTCAGATGAAAGCATCGCT
CGCCTGGCGGGCGATCGGCTGATAACCCTGCTCAACGCCCGGATTTAG
>AA QryID: NC_002696_(-)_2842412_2842507 HitID: NC_011916_(-)_2868392_2868487
rank=1 bitscore=40.0 evalue=0.19 perc_ident=100.0 perc_conserv=100.0 perc_gaps=0.0
query_cvg=100.0 subj_cvg=100.0 alpha=100.0 beta=1 prob=0.9999616350250295
GTGGTCCGCGTTCTGAGCGAGGCGCGGTCGCCGCGCAAGTCTTGCATGACGTTGTTCATG
CACAGGGAATGCGGGCGGATGGGGATGAAACGCTGA
>AA QryID: NC_002696_(+)_800330_800452 HitID: NC_011916_(+)_826360_826482
rank=1 bitscore=70.1 evalue=2.0E-10 perc_ident=100.0 perc_conserv=100.0 perc_gaps=0.0
query_cvg=100.0 subj_cvg=100.0 alpha=74.66 beta=1 prob=0.999961625189151
GTGGGGGCGGACATAACAATCACCGTCATCCCGCGCTTTATGCGCGGGACCCATTTGTCC
GCCGCAGGTGCGGTACGGCGTGAATCTCTCACGTGGAAGCTGAACCATGGGTCCCGCGCA
TAA
>AA QryID: NC_002696_(-)_2071359_2071451 HitID: NC_011916_(-)_2097335_2097427

rank=1 bitscore=35.4 evalue=4.0 perc_ident=100.0 perc_conserv=100.0 perc_gaps=0.0
query_cvg=100.0 subj_cvg=100.0 alpha=100.0 beta=1 prob=0.9999616014408779
ATGAAGGGCGCGCGCGTCGCCGACCGTCGCACCGTCAGCGCGGTTGCGAAGGGTCAGGTG
CCAAAGGCGGTCGGGTGCGGCGCAGCCCATTAA
>AA QryID: NC_002696_(-)_3734233_3734358 HitID: NC_011916_(-)_3760215_3760340
rank=1 bitscore=61.2 evalue=8.0E-8 perc_ident=100.0 perc_conserv=100.0 perc_gaps=0.0
query_cvg=100.0 subj_cvg=100.0 alpha=76.76 beta=1 prob=0.9999615753338398
TTGAGAGGGGCGGCGCGGGTGATCGCGCCGCCCTTTTCACGTTTCCGGAACCTTGATGGC
GCAGGATCCATTCACGGGCTGCGCGCTTTCGCCGCCGTGACGGGTGGCTGCAAGGGCGTA
GGCTGA
>AA QryID: NC_002696_(+)_3014045_3014341 HitID: NC_011916_(+)_3040024_3040320
rank=1 bitscore=105.0 evalue=7.0E-21 perc_ident=71.43 perc_conserv=71.43 perc_gaps=0.0
query_cvg=100.0 subj_cvg=100.0 alpha=54.59 beta=6 prob=0.9999615752342418
GTGGAAGGCGGACCATGCTCCCTCTCCCAAAGGGAGAGGGTTGGGGAGAGGGGTTACGGT
GTCCGACGGAGCGCCGGCACGCCGTCAAACCTCGTAACCCCTCTCCCTCCCACCGCTTCG
CGGCGGGCCCCTCCCTCTCCCTTTGGAAGAGGGATTCTGTTTGGCGCCAAGGTCCGCAAC
GGGCCAAAAGCCGCCTATAGCCTCCCTCCCAAAACCCGACCTTCCCGGCGACACGTCAGT
GCGTCCTTCGAGGCTCGCCTACGGCTCACACCGCAGGATGAGGAGGCCCGTTGCTGA
>AA QryID: NC_002696_(-)_3000810_3000905 HitID: NC_011916_(-)_3026790_3026885
rank=1 bitscore=41.6 evalue=0.065 perc_ident=100.0 perc_conserv=100.0 perc_gaps=0.0
query_cvg=100.0 subj_cvg=100.0 alpha=78.38 beta=2 prob=0.9999614282297874
ATGCCGTTCGGCTGGCCCCTCCACCGCCGTTCGGCGGTCCCCCTCCCCCGATGGGGGAGG
AGAGGTGTTCGCCGGGGGCAAATCTCCCGCAGTTAG
>AA QryID: NC_002696_(-)_1693007_1693252 HitID: NC_011916_(-)_1718983_1719228
rank=1 bitscore=108.0 evalue=8.0E-22 perc_ident=79.01 perc_conserv=79.01 perc_gaps=0.0
query_cvg=100.0 subj_cvg=100.0 alpha=38.2 beta=2 prob=0.9999604613127009
ATGACAGGCCTTCGCAAACGCTCGGTGAACCTTGTTGGCCACGCCACCTCCCTGGCGCTG
GAGCCAGAGTTCTGGGCGGTCCTGGAAGCCGCGGCGGCGGCGGGCGGCCAGAGTCTCGCG
GCCTTGATCCAAAGCGTAGACCGGGAACGGGGCGAGCGGCCGCTGGCTTCTGCGTGCCGG
GTCTTCGCGCTTGAGCACGTCGCTCAAACCACAGCGTCGGGGCGAGCCACGCGCGCTATC
GACTGA
>AA QryID: NC_002696_(-)_105028_105237 HitID: NC_011916_(-)_105028_105237
rank=1 bitscore=85.1 evalue=1.0E-14 perc_ident=72.46 perc_conserv=72.46 perc_gaps=0.0
query_cvg=100.0 subj_cvg=100.0 alpha=72.46 beta=1 prob=0.9999598250740378
ATGGGCCTGGATATCCGTGTCATGACCCTGGAGGCCAAGGAGCGCTCCAGCTTCGCCCAT
CGGGCCGTGGCCGCTCTGGGCCTGGTCAGCCTGGGCCTGGCCTGGGCGGTGCTGCTGGTC
CCGTGCATGCTCTGGCTCGCCGTCACCCACCTGGGACGCCGCATGGAGCCCCTCGCTCGC
CGTCCCGCGCCTGAAGGCCTCAAAAGCTGA
>AA QryID: NC_002696_(-)_1006121_1006255 HitID: NC_011916_(-)_1032152_1032286
rank=1 bitscore=54.7 evalue=1.0E-5 perc_ident=77.27 perc_conserv=77.27 perc_gaps=0.0
query_cvg=100.0 subj_cvg=100.0 alpha=77.27 beta=1 prob=0.9999499406838015
TTGCGGCCCGCCGCCATCTTGTTAACCAAATTTTTCGCGGCGCGGCGCCGGACCGATGGT
CGCGGCGGCGGCCAGATCGGCCCGGAGCTTTGCGTCGTCCTCGCCCTTCGACAGGCTCAG
GGTGAGGACGAATGA
>AA QryID: NC_002696_(-)_437556_437741 HitID: NC_011916_(-)_437556_437741
rank=1 bitscore=53.1 evalue=4.0E-5 perc_ident=77.05 perc_conserv=77.05 perc_gaps=0.0
query_cvg=100.0 subj_cvg=100.0 alpha=77.05 beta=1 prob=0.9999457675436461
GTGAACCCAGCAAGCTCAGCCCCGGACCGTTCGAGTATCCCCCTCGAACCTCATCCCGCT
CAACCGCCCCCCGCCGCCGCAAATGGTCGCTGGCCACGCGCCCTTTCCTCGCGACGAGGT
GGAACCAGTATGGGGCGGGTTGAAACGCCGGGGACAAATCGGCGCGTAAAAGCGACAAGC
GGTTGA
>AA QryID: NC_002696_(-)_1924064_1924282 HitID: NC_011916_(-)_1950040_1950258
rank=1 bitscore=67.0 evalue=3.0E-9 perc_ident=69.44 perc_conserv=69.44 perc_gaps=0.0
query_cvg=100.0 subj_cvg=100.0 alpha=69.44 beta=1 prob=0.9999042172874794
ATGATTCCACCTCGTCGCGAGGAAAGGGCGCATGGCCAGCGACCATTTGCGGCGGCGGGG
GGCGGTCGAGCGGGGACAGGTTCGAGGGGGGTACTCGGACGGTCCGGGGATCTGGTTGCT
GGCCAGACCCGCCCGCCGTCGGCGTGGTCGGGGGAAAGGGTTTCGTCCCTCTTCGATCGC
CGGGCGCTGCCGGAAACGGAAGCGCCCGGGTCCCGGTAA
>AA QryID: NC_002696_(-)_3590465_3590704 HitID: NC_011916_(-)_3616454_3616693
rank=1 bitscore=77.8 evalue=2.0E-12 perc_ident=64.56 perc_conserv=64.56 perc_gaps=0.0
query_cvg=100.0 subj_cvg=100.0 alpha=64.56 beta=1 prob=0.9998668056129876
ATGGCGTGGCCAGAGGCAGATTGTCGTTGCGAAGCCGAGGGCGGCTTTCGACCCGTTGCG

GACCTTGGCGCCAAACAGAATCCCTCTTCCAAAGGGAGAGGGAGGGGCCCGCCGCGAAGC
GGTGGGAGGGAGAGGGGTTACGAGGTTAGACGGCGTGCCGGCGCTCCGTCGGGCGCCGTA
ACCCCTCTCCCCAACCCTCTCCCTTTGGGAGAGGGAGCATGGTCCGCCTTCCACCCTTAG
>AA QryID: NC_002696_(-)_866642_866887 HitID: NC_011916_(-)_892673_892915
rank=1 bitscore=72.4 evalue=9.0E-11 perc_ident=65.0 perc_conserv=65.0 perc_gaps=0.0
query_cvg=98.77 subj_cvg=100.0 alpha=64.6 beta=1 prob=0.9998101434041744
TTGATGGACGATCGCCGGGCTCTGGAGCGACGGCAAGTCCGGGTTTGCTCACCGGTCAAA
AAAATCCGCGCACCGTCGCAGGTGACGATGCGCGGGGGAGGACGTCGCGCGACCTCGGGG
AGAGGCGCCGCGCGAAGCGCTCGAAGGCCGGTGAGGCCCTCCTCGCGCCACCGAACCGGT
AAATGGCCCCGATCCGGCAGTGACCGAAACTATAATCATATAATTCAGACCAGCAAGCGG
ATTTGA
>AA QryID: NC_002696_(+)_3657309_3657452 HitID: NC_011916_(+)_3683299_3683442
rank=1 bitscore=47.4 evalue=0.002 perc_ident=72.34 perc_conserv=72.34 perc_gaps=0.0
query_cvg=100.0 subj_cvg=100.0 alpha=72.34 beta=1 prob=0.9997815486620503
ATGGCCTACGTCTCCTATGATCAGGCTGTTCACGCGACCGCAATTCGACCGCAACGCCGC
CTCGCCTGGGGCCGGCTTCTGGCGCTCGCCGCCACGGTCGGGCTTTGGATTGGCCTGATC
GCAGCGGTTCGGGCGATTCTCTGA
>AA QryID: NC_002696_(+)_437761_437883 HitID: NC_011916_(+)_437761_437883
rank=1 bitscore=44.7 evalue=0.009 perc_ident=70.0 perc_conserv=70.0 perc_gaps=0.0
query_cvg=100.0 subj_cvg=100.0 alpha=70.0 beta=1 prob=0.999597645527617
TTGAGGGCAAAAGGATGTGTCTTAGCCTGTATTGGCTCGTCCTCCTCGATCGCTGGGGCT
TCTGGAAACAGAAGCGCCCAGGTCCCGGTAAGGCCTGAACAGGGCCACCTGACGGGACGC
TGA
>AA QryID: NC_002696_(+)_3356854_3356985 HitID: NC_011916_(+)_3382840_3382971
rank=1 bitscore=50.4 evalue=1.0E-4 perc_ident=69.77 perc_conserv=69.77 perc_gaps=0.0
query_cvg=100.0 subj_cvg=100.0 alpha=51.0 beta=1 prob=0.9994488282327081
GTGTCGGCTCGAAGAGACGACGGAGGGGGAAGGGGCAGGGTCGGCAGGACTTCCCCCTCC
GGCGCTTCGCGCCACCTCCCCCGCCAGGGGGAGGATTTGGGCTGCCCCCTTGCCTATTAT
ACGATCGTATAG
>AA QryID: NC_002696_(-)_3330675_3330869 HitID: NC_011916_(-)_3356661_3356855
rank=1 bitscore=52.4 evalue=8.0E-5 perc_ident=56.25 perc_conserv=56.25 perc_gaps=0.0
query_cvg=100.0 subj_cvg=100.0 alpha=56.25 beta=4 prob=0.9993021338254658
TTGAGGGTGGTTTTCGCACCATGCTCCCTCTCCCAAAGGGAGAGGGTTGGGGAGAGGGGT
TACGGTGTCCGACGGAGCGCCGGCACGCCGTCAAACCTCGTAACCCCTCTCCCTCCCACC
GCTTCGCGGCGGGCCCCTCCCTCTCCCTTTGGAAGAGGGATTCTGTTTGGCGCCAATGTC
CGCCCAGGGTCGTGA
>AA QryID: NC_002696_(-)_2371097_2371249 HitID: NC_011916_(-)_2397073_2397225
rank=1 bitscore=44.3 evalue=0.019 perc_ident=60.0 perc_conserv=60.0 perc_gaps=0.0
query_cvg=100.0 subj_cvg=100.0 alpha=60.0 beta=1 prob=0.9991577409006407
GTGTTAGGGGAATGGCAACTAGCGCGGGTGTTTCCTCTCCGACGAGGGACAGGAGGGGTC
ACCATGAAAGCCGGCTATGCCGCCTTGGGCGTGGGGATGTTGTTCGTTGGACTAGTCGGC
GGTCTGCTGCTGGCGCTGCGTCCGATCGGATAG
>AA QryID: NC_002696_(-)_2290920_2291141 HitID: NC_011916_(-)_2316896_2317117
rank=1 bitscore=56.2 evalue=5.0E-6 perc_ident=54.79 perc_conserv=54.79 perc_gaps=0.0
query_cvg=100.0 subj_cvg=100.0 alpha=54.79 beta=1 prob=0.9991553305741938
GTGGCCGGGACGCTATTCGAAGCGGCGTATCGCCTTCGCAGGCGCGTATTTCGCCGCCGG
ATTCGCGCCCTGCCGCGGCTTCCGCGCAGCCTCGAGGACACACAGGCCCCTGTCCTGGCC
CTGATTCGCGCTGCCGTACTGGGGGCGGTGGACGACAAGCGGACGTCCCGCAGCAGGCGA
CGTCGCCTGCGCACCGGCGCCTGTTCACCGTTAGGCGTGTGA
>AA QryID: NC_002696_(+)_841545_841664 HitID: NC_011916_(+)_867576_867695
rank=1 bitscore=36.6 evalue=2.2 perc_ident=64.1 perc_conserv=64.1 perc_gaps=0.0
query_cvg=100.0 subj_cvg=100.0 alpha=64.1 beta=3 prob=0.9990356384695004
ATGTGGTACTTCACCTGGATCCTAGGCCTCGGCCTGGCCGTCGCCTTCGGCGTGCTCAAC
GGCATGTGGTACGAGTTCAGCCTGTCCGACGAGGGCGATGAAGGCCTGGACGAGGCCTGA
>AA QryID: NC_002696_(-)_772223_772354 HitID: NC_011916_(-)_798253_798384
rank=1 bitscore=32.7 evalue=33.0 perc_ident=65.12 perc_conserv=65.12 perc_gaps=0.0
query_cvg=100.0 subj_cvg=100.0 alpha=65.12 beta=1 prob=0.9984835750201871
GTGTCGCCGGCTCGGCTGGCCCCTCCACCGCCGTTCGGCGGTCCCCCTCCCCCAGAGGGG
GAGGAGAAAGACGCACAGAACCCCGCTCCCGCCGTCGGAGAAATCCTTTCCTCCCGCCGC
CCCACCCGTTAA
>AA QryID: NC_002696_(+)_1056506_1056748 HitID: NC_011916_(+)_1082537_1082725

rank=1 bitscore=38.9 evalue=1.1 perc_ident=42.5 perc_conserv=42.5 perc_gaps=22.5 query_cvg=100.0 subj_cvg=100.0 alpha=48.67 beta=3 prob=0.993633932435128
GTGGTTTTCGCACCATGCTCCCTCTCCCAAAGGGAGAGGGTTGGGGAGAGGGGTTACGGT
GTCCGACGGAGCGCCGGCACGCCGTCAAACCTCGTAACCCCTCTCCCTCCCACCGCTTCG
CGGCGGGCCCCTCCCTCTCCCTTTGGAATAACCCCTCTCCCTCCCACCGCTTCGCGGCGG
GCCCCTCCCTCTCCCTTTGGAAGAGGGATTCTGTTTGGCGCCAATGTCCGCCCAGGGTCG
TGA

- ***Escherichia coli K12*** **substr MG**

vsORFs

>AA QryID: NC_000913_(-)_3150129_3150215 HitID: NC_020163_(-)_3905743_3905829
rank=1 bitscore=99.0 evalue=2.0E-19 perc_ident=100.0 perc_conserv=100.0 perc_gaps=0.0
query_cvg=100.0 subj_cvg=100.0 alpha=52.73 beta=3 prob=0.9987535491415732
ATGAATATTCGCGGGCGCATCCTGAAGAATTGGCATAAAGCAGAAAATTTTCGCAAATGG
CGTGCTGGTGACTTTTTTACTGCGTAA
>AA QryID: NC_000913_(+)_2854932_2855018 HitID: NC_017663_(+)_3089823_3089909
rank=1 bitscore=95.2 evalue=3.0E-18 perc_ident=100.0 perc_conserv=100.0 perc_gaps=0.0
query_cvg=100.0 subj_cvg=100.0 alpha=50.0 beta=1 prob=0.9987509021163812
TTGCTTCATAAGCATCACGCAAAAGCTGCAAAACAGCATCTTTCCCGGAACCAGCATCAA
GAACTCGCCGTTCGCTTCTTCCCCTGA
>AA QryID: NC_000913_(+)_1553743_1553832 HitID: NC_011750_(+)_1832861_1832950
rank=1 bitscore=92.7 evalue=2.0E-17 perc_ident=96.55 perc_conserv=96.55 perc_gaps=0.0
query_cvg=100.0 subj_cvg=100.0 alpha=58.48 beta=4 prob=0.9987493133241968
TTGCTTTACCGCCAGGGCGTCGGCCTCAAGTATAAAGCAGATAAAAACAAAAACACCATT
GCGCAGGCAATGGTGTTTAATCGTCATTGA
>AA QryID: NC_000913_(+)_2989805_2989894 HitID: NC_017663_(+)_3216955_3217044
rank=1 bitscore=95.2 evalue=3.0E-18 perc_ident=100.0 perc_conserv=100.0 perc_gaps=0.0
query_cvg=100.0 subj_cvg=100.0 alpha=41.53 beta=1 prob=0.9987480423837592
TTGCTGGTTATAGTGCAGTTAACCACTATAACCAGCACCTCTTATGTTTTCTTAAATCAG
CATCTTAAAACAGTTAAGAGCATACTATAA
>AA QryID: NC_000913_(-)_4372284_4372367 HitID: NC_017663_(-)_4688173_4688256
rank=1 bitscore=90.6 evalue=8.0E-17 perc_ident=100.0 perc_conserv=100.0 perc_gaps=0.0
query_cvg=100.0 subj_cvg=100.0 alpha=61.48 beta=3 prob=0.9987477250374495
GTGAGTACGGCTATTTATGTTATTTTAACAACTATTACTACCAAGCCCATTTATCTGGCT
TATAAATTAAAATTAATTATTTAA
>AA QryID: NC_000913_(-)_2334699_2334779 HitID: NC_011741_(-)_2387843_2387923
rank=1 bitscore=91.4 evalue=4.0E-17 perc_ident=100.0 perc_conserv=100.0 perc_gaps=0.0
query_cvg=100.0 subj_cvg=100.0 alpha=48.29 beta=1 prob=0.9987449188307742
TTGTTTGAATTGAAAAGTCCAGGCTGCAAAGTCTGGGCTTTTGTCGTATTAGGGCACGGT
AAAGTTTGGCTGTGCCCGTAA
>AA QryID: NC_000913_(+)_1480983_1481057 HitID: NC_011750_(+)_1799895_1799969
rank=1 bitscore=85.9 evalue=2.0E-15 perc_ident=100.0 perc_conserv=100.0 perc_gaps=0.0
query_cvg=100.0 subj_cvg=100.0 alpha=65.28 beta=1 prob=0.9987434770809678
ATGTTCGAATTTATTGAACAACGCATAGAAAGCCGCGATGTGGTACTCTATATCTATCAT
TTAAAAGAAAATTAA
>AA QryID: NC_000913_(-)_2493548_2493622 HitID: NC_011748_(-)_2727904_2727978
rank=1 bitscore=84.6 evalue=5.0E-15 perc_ident=100.0 perc_conserv=100.0 perc_gaps=0.0
query_cvg=100.0 subj_cvg=100.0 alpha=58.14 beta=3 prob=0.9987300248562967
ATGTCGACAGCGGTACACTCAATTGGTTGGGCCATCATGTACGACAATGGTTCCTGTAAA
GAGTGTAAAGACTAG
>AA QryID: NC_000913_(+)_921963_922037 HitID: NC_011750_(-)_2334605_2334679
rank=1 bitscore=80.8 evalue=7.0E-14 perc_ident=100.0 perc_conserv=100.0 perc_gaps=0.0
query_cvg=100.0 subj_cvg=100.0 alpha=62.98 beta=1 prob=0.998724685206511
ATGAATCAAAAATTTGAAGCAGTTAACGCTATTGACAGGAATGTGACAGATGTCGCTGAT
GCCAACGATAGATGA
>AA QryID: NC_000913_(-)_3182615_3182689 HitID: NC_011741_(-)_3267419_3267493
rank=1 bitscore=80.8 evalue=7.0E-14 perc_ident=100.0 perc_conserv=100.0 perc_gaps=0.0
query_cvg=100.0 subj_cvg=100.0 alpha=46.5 beta=1 prob=0.998693520421577
TTGAAAGCCCGCGAGCGCTTTGGGTGCGAACTCAAAGGACAGCAGATCCGGTGTAATTCC

GGGGCCGACGGTTAG
>AA QryID: NC_000913_(+)_1800879_1800950 HitID: NC_011741_(+)_1846895_1846966
rank=1 bitscore=76.1 evalue=2.0E-12 perc_ident=100.0 perc_conserv=100.0 perc_gaps=0.0
query_cvg=100.0 subj_cvg=100.0 alpha=55.53 beta=1 prob=0.9986708278352675
GTGCGTCTGAAAGGTTTACTGCGAGCGGATTTACAGCAGCATAAACTGAATCCAGCAAAG
TTCGTTGCGTGA
>AA QryID: NC_000913_(-)_4499791_4499853 HitID: NC_011750_(-)_4953920_4953982
rank=1 bitscore=71.9 evalue=2.0E-11 perc_ident=100.0 perc_conserv=100.0 perc_gaps=0.0
query_cvg=100.0 subj_cvg=100.0 alpha=51.43 beta=1 prob=0.9985807409085907
GTGTATATGAACGATTGCACTCTGACGATGGTTGATTTATTTGGTTGCGTAGGGGCTAGC
TAG
>AA QryID: NC_000913_(+)_2901921_2901998 HitID: NC_002655_(+)_3691310_3691387
rank=1 bitscore=65.1 evalue=4.0E-9 perc_ident=80.0 perc_conserv=80.0 perc_gaps=0.0
query_cvg=100.0 subj_cvg=100.0 alpha=47.5 beta=2 prob=0.9984231878034715
GTGAACCTCTCTGGCATGGAGAACTATTTTGAACATGAGGTGTTACGTGGATATGTTGCT
TATTACAAGTACTGCTAA
>AA QryID: NC_000913_(-)_4529979_4530035 HitID: NC_020163_(-)_553108_553164
rank=1 bitscore=64.3 evalue=4.0E-9 perc_ident=100.0 perc_conserv=100.0 perc_gaps=0.0
query_cvg=100.0 subj_cvg=100.0 alpha=54.98 beta=1 prob=0.9983830437671237
ATGTTTTCTTACATTATGCTGGGAACCAACGACCTGCCACGCGCCATTACATTCTAA
>AA QryID: NC_000913_(-)_3921709_3921756 HitID: NC_017663_(-)_4233077_4233124
rank=1 bitscore=53.2 evalue=8.0E-6 perc_ident=100.0 perc_conserv=100.0 perc_gaps=0.0
query_cvg=100.0 subj_cvg=100.0 alpha=41.88 beta=1 prob=0.9978171318512199
ATGCCTGGTAAGCACCCGCTTACCAGGCAACGCATCAAGAACAGGTAA

sORFs

>AA QryID: NC_000913_(-)_3656917_3657213 HitID: NC_017625_(+)_224591_224869
rank=1 bitscore=174.0 evalue=1.0E-41 perc_ident=100.0 perc_conserv=100.0 perc_gaps=0.0
query_cvg=93.88 subj_cvg=100.0 alpha=72.24 beta=10 prob=0.9980195598050714
TTGCGCGCGAGGAGAGATATGAGGGATTTACAGACCAGCGGCATAGTGGGGTTGTCAGCC
AGTAAGGTGGGATACAGGCACAGTGATCGACATGGTGAGGTCAACGACAAAAAAAATGTT
GCCGTTCTGCCAACAGTTCCTGCCAGCATTCGGGCAACGAAGGGTTCTACTGGTGGATAC
ACATACCAGGGGAATAAAGTCGTTTTAGCCTCTCCTTTAGTAATCACCGGGGGTAATGAA
ATCAGCATCTGCATCCCTCGTCATGCCAGCCATCAATTTCAGTTGCTTATGTCCTGA
>AA QryID: NC_000913_(+)_2404903_2405157 HitID: NC_012947_(-)_1445517_1445771
rank=1 bitscore=149.0 evalue=6.0E-34 perc_ident=97.62 perc_conserv=97.62 perc_gaps=0.0
query_cvg=100.0 subj_cvg=100.0 alpha=71.65 beta=3 prob=0.9980195597616466
ATGACATGCGCGTGTTTGTATAAATGTAAATGTGAGTCCTTGTTCCACTCTCGTGCAGCA
TCGCTGGTCATACGCGAACACGTACCAACAGCAATGGTGAGGCGGCATCAAGAGCAGGGA
TCCGTCATTTATCCGAGCATTTTACCCCAAAAACCTTTTATTTATAAGGGTCATTGCGAA
TTATCTGATGCAAAGTTATGTTATGTTAGGCAAAGTAATCTTCTATTATTAATAAGCACA
TCAAAACCTTTTTGA
>AA QryID: NC_000913_(+)_3656960_3657253 HitID: NC_011415_(+)_3904079_3904372
rank=1 bitscore=165.0 evalue=9.0E-39 perc_ident=98.97 perc_conserv=98.97 perc_gaps=0.0
query_cvg=100.0 subj_cvg=100.0 alpha=78.98 beta=10 prob=0.9980195597560617
ATGCAGATGCTGATTTCATTACCCCCGGTGATTACTAAAGGAGAGGCTAAAACGACTTTA
TTCCCCTGGTATGTGTATCCACCAGTAGAACCCTTCGTTGCCCGAATGCTGGCAGGAACT
GTTGGCAGAACGGCAACATTTTTTTTGTCGTTGACCTCACCATGTCGATCACTGTGCCTG
TATCCCACCTTACTGGCTGACAACCCCACTATGCCGCTGGTCTGTAAATCCCTCATATCT
CTCCTCGCGCGCAATTTAAAGAACCGTTATTTCTCAAGAATTTTCAGGGACTAA
>AA QryID: NC_000913_(-)_2055643_2055939 HitID: NC_007946_(-)_2109296_2109583
rank=1 bitscore=149.0 evalue=4.0E-34 perc_ident=87.37 perc_conserv=87.37
perc_gaps=1.05 query_cvg=95.92 subj_cvg=100.0 alpha=58.11 beta=7
prob=0.9980195597431551
GTGCGCTTAATCGAGTCTCACGCTTCAGTGAGCAAATTGTCCCTCCCCAACAGACATCAC
GATTCGCAAGGTCAAAAACTCTGTTCCATGAAGTACCGTACAAAGATAAAGAGAAAAATG
CCTTATGCGGGATGTCGCTATCGCGTCTTTATCGGGAAACTATGCGTAATTGCCACTTTG
CCTTCTGTAATCAGAAAAAGAAGAACTGGAATTTTAATAAATATTATTTCTCTGGGAACG
GGGGGGTATAATCAATATGACGAGTTGAATATTATATTTTTTATAAGAATTATATGA
>AA QryID: NC_000913_(-)_2459041_2459292 HitID: NC_017651_(-)_2680311_2680562

rank=1 bitscore=152.0 evalue=5.0E-35 perc_ident=97.59 perc_conserv=98.8 perc_gaps=0.0
query_cvg=100.0 subj_cvg=100.0 alpha=68.8 beta=6 prob=0.9980195597362511
ATGTTACGTGCTGTAACAGGAGCGCGAAGTGTAGGGTCTGAGGTACATCTTAGAAATCAG
ACCAGTGGCGAGAGTATAGGTCGGACCAGCTGGAATGTTGCAAGTATGTTTCTATCTTTT
TTCAAATATGATCTAAGAAACGCGATCCAGCTGGCAAAATTTGGAGGTGGCGCACTCATG
GATTCGGGTGAATTTTGTTTTAGATCATTTTTAAGTGTGATTTCGGTCACTTATCCGATT
TTGCAGAATTAA
>AA QryID: NC_000913_(-)_3077362_3077613 HitID: NC_017626_(-)_3356105_3356356
rank=1 bitscore=151.0 evalue=1.0E-34 perc_ident=100.0 perc_conserv=100.0 perc_gaps=0.0
query_cvg=100.0 subj_cvg=100.0 alpha=72.21 beta=5 prob=0.9980195597346901
ATGTCCGTTTGCGGACAAGCAATAGATAAGGCGTGTTGTAGATCACAAATATTTATATGC
AATAAATATCAATTATGTAATATGCATCACGATATGCGTATTGACATTTGTTGTTATATC
TATAACTCAATGTTATATAAGAAATTAATAATTCACTGTTTTCAAAACACCGGTTTCCCT
GCTCAATTGCTTTCATTAAACCGCACGAGTATTTTATTTTTACCGCCGGACGGTACCCTA
AAAATAAAATAA
>AA QryID: NC_000913_(-)_3319807_3320031 HitID: NC_017626_(-)_3690621_3690845
rank=1 bitscore=134.0 evalue=2.0E-29 perc_ident=95.95 perc_conserv=95.95 perc_gaps=0.0
query_cvg=100.0 subj_cvg=100.0 alpha=68.47 beta=1 prob=0.9980195594948527
TTGTCAGGTATCTGTATCTCGTCCAGTTGCTTTACTCGTAGCTGGTACCTTGAAACGATG
GTGCCGGTACGCCTTAGTTATAAATTTTTCCCTTCTACTGACATAATTGGCACTTTATTG
TGTCAACTCCAGTTCCGGTACGAATACGGATATGTTTACTTCTCGATAAACCATCCTGCT
CCTGTCAGTATAACGACTGATGGCTACGTTCTGCGTAATGTTTAG
>AA QryID: NC_000913_(+)_2040945_2041187 HitID: NC_013941_(+)_2437248_2437490
rank=1 bitscore=138.0 evalue=1.0E-30 perc_ident=93.75 perc_conserv=96.25 perc_gaps=0.0
query_cvg=100.0 subj_cvg=100.0 alpha=93.75 beta=3 prob=0.9980195594945888
GTGAACCATGGACATCAACGCGTCATAAATGTTTACGCAAGTGCACAATGCACCCTTTCC
ACTATCAACAAGGAGGAGCAGAAACTGTGTAAATCGTACAATGGTAAGACTTATCAAAAA
TACAGGTTAATTTTGGTATTTAAACCTTTTTTACTTGTTAGTATGATACAAAGGCTTTCA
AAAAAGCTGCGAAATTCAACAACTCTTGGAAATTATGGACTTTGTCCCGCGGCTTTAATC
TGA
>AA QryID: NC_000913_(-)_2033476_2033712 HitID: NC_013008_(-)_2566583_2566786
rank=1 bitscore=127.0 evalue=1.0E-27 perc_ident=94.03 perc_conserv=95.52 perc_gaps=0.0
query_cvg=85.9 subj_cvg=100.0 alpha=65.75 beta=3 prob=0.9980195592618971
GTGTTGAATATTATTCACGCCAAATTTTCCTTATTGAGCAACGTCGTCGCAAGTGGCTTC
AATGATTTTAGACCATTATCAGAGGTACGGGGTGGCGGGGAGAGATTTAGTGCTGTGGAA
TATTGTGGTGCTGATGAATATTTATATCCCGGATTGGTAGAGAAAAAGCTTATTTCATGC
ACTATTTTTGCAAATATTTCTATATGGGAAGCGTGTTTGTTACGGATTAATTCATAA
>AA QryID: NC_000913_(-)_2288233_2288490 HitID: NC_011741_(-)_2349336_2349593
rank=1 bitscore=145.0 evalue=6.0E-33 perc_ident=98.82 perc_conserv=98.82 perc_gaps=0.0
query_cvg=100.0 subj_cvg=100.0 alpha=78.46 beta=9 prob=0.9980195585945825
ATGTTTGAGCGTAGCGTTGGTAACGCTACCAGGCGCGTGAATGTTACCCCATTTTATGTA
TGGACATGTAGTGAAAACTACAACCTTGTCGTTCGCTTTGGCATCATTTTCATTATAAAG
ACGGAATTTTGCATCAGCTTCTCATCAAGAAAAACAACTAAAAAATCAATCACAATACTT
GTGCATTTCTTTAAACAGTTTTTAAAAACTGACTCCGGGTATGGAGCTATGGGTATTTTC
TGTACCCAATGCTTTTAA
>AA QryID: NC_000913_(-)_4518447_4518638 HitID: NC_011751_(-)_5058584_5058775
rank=1 bitscore=125.0 evalue=5.0E-27 perc_ident=100.0 perc_conserv=100.0 perc_gaps=0.0
query_cvg=100.0 subj_cvg=100.0 alpha=76.03 beta=2 prob=0.9980195575470634
GTGACGGCAACCGTCTGCACGGTGACGAATCGCTGGCGATCAAAAAGGGGCGATATTGAA
CCTTCGATTATGCTTGATGGGTGTATTCTCAGTAATACTGTAAAGCCGGAGCATGTCTCC
GGCTTTACAGTATGGGGGAACAGCGGGCGATCGCTCAAATGGTATGCTGGTTTATTTCAT
GTAAAAGAATAA
>AA QryID: NC_000913_(-)_924842_925081 HitID: NC_011415_(-)_1009493_1009732
rank=1 bitscore=121.0 evalue=1.0E-25 perc_ident=86.08 perc_conserv=87.34 perc_gaps=0.0
query_cvg=100.0 subj_cvg=100.0 alpha=61.37 beta=3 prob=0.9980195574335429
GTGCGAGCTTTTTTTTCGCATTTTATCTGTCTGTACGGCAGTGAACATGGTGGGCGGGTG
GAGTATGTTACCTGTGAATTTCTAAGCTGCCTGTACGGCAGTGAACTCAAATACTACACG
TTTAGCCCGTTGTTAATAAAGCAAAACCATCCCATTCTCCTGCAGCACCCTTTTTTATCC
TTACCTTCCCGCAGCACTAATTATCAACAACTTACCATCATCATTAAAAAAAGGGTCTGA
>AA QryID: NC_000913_(+)_1285932_1286207 HitID: NC_007779_(+)_1288286_1288561
rank=1 bitscore=154.0 evalue=1.0E-35 perc_ident=100.0 perc_conserv=100.0 perc_gaps=0.0
query_cvg=100.0 subj_cvg=100.0 alpha=79.44 beta=14 prob=0.9980195574084554

ATGGTGGTGGGGGAAGGATTACTCAGCGCTGCGCGCTTCGCCCTTCGGGTCGTTGCCTGC
GGCAACGCTCTCTCGCTGGCGCTCAAGTCGAACCTTGGTCGAAGCTTCTCATCCTTCCCC
GCTTGGGCAGAATATTTGATTGCGGATTCGTTTGAGAATTCCGGGGCTTTTGAAAGTGAT
GGTGGTGGGGGAAGGATTACTCAGCGCTGCGCGCTTCGCCCTTCGGGTCGTTGCCTGCGG
CAACGCTCTCTCGCTGACGCTCGAGCCGAACCTTAG
>AA QryID: NC_000913_(+)_1328737_1328949 HitID: NC_011742_(+)_1393671_1393883
rank=1 bitscore=143.0 evalue=4.0E-32 perc_ident=100.0 perc_conserv=100.0 perc_gaps=0.0
query_cvg=100.0 subj_cvg=100.0 alpha=73.95 beta=10 prob=0.9980195571890661
TTGCAGACAAATATACCACGCTGGTGGCAAGAGCGCCTTACTGGCAACTTTGGATTTTGC
ATGCTAATAAAGTTGCGTATCGGATTTTATCAGGTACAGTGTGACGCTTTCGTCAATCTG
GCAATAGATTTGCTTGACATTCGACCAAAATTCCGTCGTGCTATAGCGCCTGTAGGCCAA
GACCTGTTAACTCAGTCACCTGAATTTTCGTGA
>AA QryID: NC_000913_(-)_1407287_1407523 HitID: NC_013008_(-)_1883413_1883649
rank=1 bitscore=120.0 evalue=3.0E-25 perc_ident=96.15 perc_conserv=97.44 perc_gaps=0.0
query_cvg=100.0 subj_cvg=100.0 alpha=77.65 beta=1 prob=0.9980195569647516
GTGGTCATCGACGCAAAGTTAGCAGGCGCGTATCCTCGCAGATCTACGCTCACGATGCGA
CAATTTAATCGGTTCTTCATCGGGTGGTGGGTCAGGCATGGGTTGCGGGCGAGGGATCGG
ATCGGGCACTGGAACAGGATCGCCAGGAATCGGTTCAGGGACAGGAATTTGCAAATAAAT
AAGTGTCGTCATATTTCCCTCTGGTCATTGGGTGGACTCTTAAAGGGTAGACGCTGA
>AA QryID: NC_000913_(-)_623737_623946 HitID: NC_012947_(+)_3197177_3197383
rank=1 bitscore=114.0 evalue=2.0E-23 perc_ident=100.0 perc_conserv=100.0 perc_gaps=0.0
query_cvg=98.55 subj_cvg=100.0 alpha=73.13 beta=1 prob=0.9980195512160394
TTGATGGATTCGCATAAGCGCAATGTGATGTCCTGCGCCGTTCTGCCCCCTCTCCCTTCC
AGGGTGAGGGCTGGGGTGAGGGTTAATGTTCGCACCAGTGCTGGCTGTTCCCCTCACCCT
AACCCTCTCCCCAAAGGGGCGAGGGGACGGATTGTGCGCTTTGTCGAATTTGTCATTACG
CCCTTAACCTTATTAATAACAGGAAGCTGA
>AA QryID: NC_000913_(+)_3483920_3484135 HitID: NC_011750_(+)_3985169_3985384
rank=1 bitscore=132.0 evalue=7.0E-29 perc_ident=100.0 perc_conserv=100.0 perc_gaps=0.0
query_cvg=100.0 subj_cvg=100.0 alpha=78.67 beta=9 prob=0.9980195462481621
ATGCTGAAGCGAGACACCAGGAGACACAAAGCGAAAGCTATGCTAAAACAGTCAGGATGC
TACAGTAATACATTGATGTACTGCATGTATGCAAAGGACGTCACATTACCGTGCAGTACA
GTTGATAGCCCCTTCCCAGGTAGCGGGAAGCATATTTCGGCAATCCAGAGACAGCGGCGT
TATCTGGCTCTGGAGAAAGCTTATAACAGAGGATAA
>AA QryID: NC_000913_(+)_4499057_4499251 HitID: NC_011745_(+)_5068276_5068470
rank=1 bitscore=116.0 evalue=3.0E-24 perc_ident=100.0 perc_conserv=100.0 perc_gaps=0.0
query_cvg=100.0 subj_cvg=100.0 alpha=70.08 beta=3 prob=0.9980195454065102
TTGTTGCACGATCGATCAATTGTTATGCATGCTGACACCACAACCCCGGTGGGTATGACC
GATGCCTTTCTCACTCTCTTCCCGGTCATCGTCGGCCTTCCCCGTTATTGTGGCGTGTCA
GCACCCTCGACGGCATTTCGATGGCTGCTCAGTGTTCACTCACGTTACGGCCCGCATAAC
CCGTTGACTTCCTGA
>AA QryID: NC_000913_(-)_4276247_4276465 HitID: NC_009801_(-)_4617308_4617526
rank=1 bitscore=127.0 evalue=2.0E-27 perc_ident=100.0 perc_conserv=100.0 perc_gaps=0.0
query_cvg=100.0 subj_cvg=100.0 alpha=72.75 beta=8 prob=0.9980195413767872
ATGCGTGGCGGACAATCCCCTGCCAGCGAAACGTTATCTTCCTGCTCCAGGTTTGCGACA
ATTTGCTGCGTCGCAAAAAAGCAAACGTTTAGCTCCGCGCATACAAAACGGGTGGTAAAA
AAAGCAAACGATTATCCGGCGATCATAGCGGCTTTTTCAACCGAAGTTTGCTGCTTTTTT
CTCTTTTATTGTCTTGACGATGAAAAACCAGGCAATTGA
>AA QryID: NC_000913_(-)_331090_331275 HitID: NC_012947_(+)_3480705_3480890
rank=1 bitscore=115.0 evalue=9.0E-24 perc_ident=100.0 perc_conserv=100.0 perc_gaps=0.0
query_cvg=100.0 subj_cvg=100.0 alpha=69.32 beta=4 prob=0.9980195341700309
ATGAGCAAAGGCGCATTATATGAATTTAACAATCCAGATCAACTGAAAATACCTCTCCCT
CATAAACACATAGCGTCAACATTCAATGACATAATGAGTAAAGATGTTGGTTATGCATAC
GTATCATTACTCTATGCCTGTCCCTTAAAAACCCACTCATTAAGACTGAATCCATTCAGC
AAATGA
>AA QryID: NC_000913_(+)_1333619_1333801 HitID: NC_011748_(+)_1477322_1477504
rank=1 bitscore=112.0 evalue=6.0E-23 perc_ident=100.0 perc_conserv=100.0 perc_gaps=0.0
query_cvg=100.0 subj_cvg=100.0 alpha=74.55 beta=3 prob=0.998019527466388
TTGGTCCTGGATGGGCGCGTTTTCTCTGTCGATGCTCTTCTGGGCCGAACTCCTCTGGAT
CATTACTCACTGATCCTTGACCCCGCTGCGGCGGGGTTGTCATTTGCTTTGCCACAAGGT
TTCTCCTCTTTTATCAATTTGGGTTGTTATCAAATCGTTACGCGATGTTTGTGTTATCTT
TAA
>AA QryID: NC_000913_(-)_389121_389390 HitID: NC_010473_(-)_328452_328721

rank=1 bitscore=139.0 evalue=8.0E-31 perc_ident=100.0 perc_conserv=100.0 perc_gaps=0.0
query_cvg=100.0 subj_cvg=100.0 alpha=84.67 beta=14 prob=0.9980195202604232
ATGCTGCGTTACAGACGAATGGTTCAATTATTCTTTCCCCTAAGTCCGAATGTGGACATA
TATAATTTATGGAGAAAAACACGCATCAAATTCATAAAAGCACAGTATTTCAACATGTTG
CAGATAATTTTGTTTTTGAAGCATACGCATAATAAATATCACAAACACAAACCAGATAAA
TATCCTATGAATATGCAACAAAGTTTTAATTATTCAATTTATAGCAGCGCAACCAAACTC
CAAAAAATAATAACCAACAGCTATAAATAA
>AA QryID: NC_000913_(-)_3107257_3107460 HitID: NC_017652_(-)_3292215_3292418
rank=1 bitscore=117.0 evalue=2.0E-24 perc_ident=95.52 perc_conserv=95.52 perc_gaps=0.0
query_cvg=100.0 subj_cvg=100.0 alpha=71.94 beta=7 prob=0.9980195114878191
TTGCGCGAATTTTCTCCTCTCTGTACGGAGTTTGCCCGATGCACGCCATCTCCTTACATT
CTCTCGCTTATCGCCGTTTCGCGCGAAACGTTTCCCTTTCTATGTTACTGCTCATGCGGT
GAAGTTAACGCACGCTCACTGCAGGACAACAGTAAAATCAGAGCGTTTCTGCTTTTACTG
ATGTCTGGCGGTCGGAGCTGGTGA
>AA QryID: NC_000913_(-)_4540736_4540924 HitID: NC_008253_(-)_4847951_4848166
rank=1 bitscore=109.0 evalue=6.0E-22 perc_ident=95.08 perc_conserv=96.72 perc_gaps=0.0
query_cvg=98.39 subj_cvg=85.92 alpha=55.9 beta=2 prob=0.998019507143106
ATGTTTATATTGCATGAGGTGGTTTTTGGAGAGAAGAATGAGGAAGATGCGTCGAGCCAC
AGAAACGTTAGCTTTACATATAGCGGAGGTGATGTGAAATTAATTTACAATAGAAATAAT
TTACATATCAAACAGTTAGATGCTTTTTGTCGTTTTTTAATATTTTTATGCTTGAGAAAA
AATACGTAA
>AA QryID: NC_000913_(-)_4570184_4570381 HitID: NC_017638_(-)_4551939_4552136
rank=1 bitscore=110.0 evalue=3.0E-22 perc_ident=100.0 perc_conserv=100.0 perc_gaps=0.0
query_cvg=100.0 subj_cvg=100.0 alpha=100.0 beta=2 prob=0.9980195032143049
TTGAAAGAAAACCTTTCAGAATATTGTATAGTTACTCAGGCGGCAACAACTATTTTTTCA
GGTCGATACTATAAATCAATTTTAGATAAAAATGATATCAACCATCTATTAATCACAACT
CATTTATCACCTGATTTTATAATTTCAAATGAAAATAAATCTCAAACAATCAAAAAGTAT
TCAGTAGCTGACATTTAA
>AA QryID: NC_000913_(-)_3646125_3646286 HitID: NC_009800_(-)_3690613_3690774
rank=1 bitscore=103.0 evalue=3.0E-20 perc_ident=100.0 perc_conserv=100.0 perc_gaps=0.0
query_cvg=100.0 subj_cvg=100.0 alpha=69.56 beta=1 prob=0.9980194986255106
TTGCAAGAATTAGCGATTTCTTCAGCTGGCGGAGGGAGCACTTCGGTATATATCGCAGTG
ACGATTTATATACTTTCACTGGGTCATCGTCATATTAAGCCTTGTCCGAATCCGGATGTA
AAAAAACCGACTTTGCGTCGGTTTTTTACTTTCCAGCCCTGA
>AA QryID: NC_000913_(-)_3403155_3403322 HitID: NC_017625_(+)_478470_478631
rank=1 bitscore=105.0 evalue=5.0E-21 perc_ident=100.0 perc_conserv=100.0 perc_gaps=0.0
query_cvg=96.36 subj_cvg=100.0 alpha=84.66 beta=4 prob=0.9980194009893304
GTGTCAATGTTAGACTTGACGGACATTGTGCAGCGATCTGCTGCTATCCTCCGGCAAAAA
ACAAAATATACCTTCGTTGCGCCAATGTCACCTTTTCCGCAGCGCAAAAACTGCGTCAGG
GAGGACGAGGTCGCACATTATAACGATTTCGTAGCAATTGGCAGCTAA
>AA QryID: NC_000913_(+)_668335_668505 HitID: NC_013654_(+)_640618_640788
rank=1 bitscore=99.4 evalue=5.0E-19 perc_ident=100.0 perc_conserv=100.0 perc_gaps=0.0
query_cvg=100.0 subj_cvg=100.0 alpha=68.58 beta=2 prob=0.9980193896502585
TTGTCGCCGAATCGGCTTTCGAAAGTGGGCTATCATCCCACCCCGCGCCGCAGATTGCAT
CGCCATTTTTGTAAAACAATTTCTACAAAGTCGTGTCTGGCGGAAAAAGTCTGGCTGCGG
AGAATATCAGCCTGCCCGGGTCTGTCAATGGTCGTTGCGGCTTACCCGTAA
>AA QryID: NC_000913_(+)_4558715_4558924 HitID: NC_011415_(+)_4809653_4809862
rank=1 bitscore=119.0 evalue=4.0E-25 perc_ident=98.55 perc_conserv=98.55 perc_gaps=0.0
query_cvg=100.0 subj_cvg=100.0 alpha=76.34 beta=10 prob=0.998019383333812
TTGTTTTTTTGTGTTTGTGTGTTGCTTGAGCCAGGAAAATTCCTGGAACTGAAAAACCTT
ATCAGCCATGTGGGTATGGGGAAAATGCAAGTATGTTTCTGGCTATGCGTTTTTTGCATA
GCGGAAAAAATTACGCTGTGTGGAAAAGGCTCTGCGCAGGATTGAGTTGCAGAAAATTCAT
AAACCAAGTGACAATGACATCAGTAAATAA
>AA QryID: NC_000913_(-)_344278_344448 HitID: NC_017641_(-)_383799_383969
rank=1 bitscore=95.5 evalue=6.0E-18 perc_ident=100.0 perc_conserv=100.0 perc_gaps=0.0
query_cvg=100.0 subj_cvg=100.0 alpha=66.43 beta=1 prob=0.9980193475353165
ATGTCAACCAACACATTCACAAAACATCTGCATGAGATCATTTTTTATACACCGCATGGC
TGCGAAACCACTACTGGTGCTTTTTTAAGCGAAGTGTCGCCGATCACAACAAAATATCGG
CGAAAAAAGAGATGGAATGCCAGTTGGCATACAGATATACAGATGAAATGA
>AA QryID: NC_000913_(-)_320463_320633 HitID: NC_011353_(-)_367834_368004
rank=1 bitscore=100.0 evalue=2.0E-19 perc_ident=100.0 perc_conserv=100.0 perc_gaps=0.0
query_cvg=100.0 subj_cvg=100.0 alpha=78.57 beta=3 prob=0.9980192608824537

ATGGGGAAATCGACGATCATTTTTGGCATAAGAAATCCATTTAAAAATGGCTACATCCCT
TATACCTATTCAGAGCTACTTATTCAGGGGTTATTGAGATATACACTGATGGTGTACAAA
AAAATCACCGACAATAATCACGTATCAAGTAATAGTCGGGATTTTATTTAA
>AA QryID: NC_000913_(+)_3783062_3783259 HitID: NC_011415_(+)_4036203_4036400
rank=1 bitscore=105.0 evalue=7.0E-21 perc_ident=100.0 perc_conserv=100.0 perc_gaps=0.0
query_cvg=100.0 subj_cvg=100.0 alpha=76.58 beta=6 prob=0.9980191341172087
GTGATTAAAAAAACCAGGAAAGGAGTATACCTGCGCGGTGCGGCAAATACAGCCAGCGCG
TTAACTGGAATGCAATTTTGCGGGGCGCGACGAAAAAAAAGCGTAAATCTGGCATCGGCT
TGCCGCGAGCGGTCGTATATTTTGATCTTCAGAGGCTATTTTATCGATTCAGCTGTAGTA
AAATTACGCAAATTTTGA
>AA QryID: NC_000913_(+)_2967311_2967505 HitID: NC_017634_(+)_2956289_2956483
rank=1 bitscore=82.8 evalue=5.0E-14 perc_ident=76.56 perc_conserv=76.56 perc_gaps=0.0
query_cvg=100.0 subj_cvg=100.0 alpha=55.98 beta=2 prob=0.998019098079953
GTGACCCAGGTCACACCTCTCATTTACGGGTTGGCGAAAGATCAACCAATGCCGTATTTA
TCCACAGAATGTGCCACTAAGTTAAGCACTGAACCACTAAAAACTGGAGTTTCGTCGCAC
GTCAAGGCTGTAAATGGAAACAGTAGTGGAGGTTTTTCACAGTTATCCCAGCTTTCTGTG
GATAACATGGTGTAA
>AA QryID: NC_000913_(+)_2613934_2614110 HitID: NC_007946_(+)_2751736_2751912
rank=1 bitscore=102.0 evalue=6.0E-20 perc_ident=100.0 perc_conserv=100.0 perc_gaps=0.0
query_cvg=100.0 subj_cvg=100.0 alpha=75.95 beta=5 prob=0.9980190963604579
TTGCGATCCGCAATTGTATCGAAATGTCACAAAAAAGACTTCGCTTTTTATGACGGATTC
AGGAAACTGAAAAGTCATTTGAGTGGGCTAATCTTCGCCGTTACACTCAAAGGCGGCGCG
GTGGGAACGATATTTCACAGTATCGGTCAAATGACTATCTCCAGAAATACAGGATAG
>AA QryID: NC_000913_(-)_1078257_1078448 HitID: NC_017651_(-)_1079160_1079351
rank=1 bitscore=109.0 evalue=3.0E-22 perc_ident=100.0 perc_conserv=100.0 perc_gaps=0.0
query_cvg=100.0 subj_cvg=100.0 alpha=74.19 beta=8 prob=0.998019093162009
ATGTTTTACCAGGGCGACCGTATCCTGCCGGAAGCGCTGGTTATTCACAATCGATTTAAC
ACACCATTTACATTAAATTTTAGTGCTCAGCGACACTATTTTTCATCAGGTTGCACTCTC
TCACATTTTTTGCGGTTGCACCTTTCAAAAATGTTAACTGCCGCAGAGAAAAAGTCTGAG
TTATTTTTTTAA
>AA QryID: NC_000913_(+)_2492469_2492645 HitID: NC_008253_(+)_2517891_2518052
rank=1 bitscore=84.0 evalue=2.0E-14 perc_ident=88.68 perc_conserv=94.34 perc_gaps=0.0
query_cvg=91.38 subj_cvg=100.0 alpha=84.86 beta=1 prob=0.9980190903547581
TTGGGTGAGCATGAAATGAGAAATCAGAGAGCAACGGTCATTTTGAAAGGGTTATCTTAC
AGTTGTAGTTATTATCGCAAGAAGTATTTTAGTCTTACAATTGTAGGAATATGCTTCCTG
TCGCAGAGAACTATTCTTATATACCACAAAGAGATGTCATTTATTATATCGATGTAA
>AA QryID: NC_000913_(+)_4566811_4566984 HitID: NC_012947_(-)_3864233_3864406
rank=1 bitscore=94.4 evalue=2.0E-17 perc_ident=100.0 perc_conserv=100.0 perc_gaps=0.0
query_cvg=100.0 subj_cvg=100.0 alpha=74.15 beta=2 prob=0.9980190898776995
TTGCGGTTAATCAAAAGAGCGCGGTGGAAAGGGGAATATCTGCCGGGTACAGGACATGTA
AAACGCGAGCTTGTTTCCGGAAAAAATGTATCGTTTGCAGAAGAATAAAAATAGCCTGGAA
AGCGCCTCGGGGAATGGGAAATTGCCGGGTGAGAATGGTTTTGTTAGTCGCTAA
>AA QryID: NC_000913_(-)_384090_384305 HitID: NC_017633_(-)_445730_445945
rank=1 bitscore=92.0 evalue=9.0E-17 perc_ident=100.0 perc_conserv=100.0 perc_gaps=0.0
query_cvg=100.0 subj_cvg=100.0 alpha=77.79 beta=1 prob=0.9980190470823385
TTGGGAAAAATTACTACACTTCATTTTAGTGATGCATAAACAACCACATTACGAGATTTAT
TACTTCATTATTTCTATCAATATCAATACATATAATAAAACTATTATATTCGCATTGTAT
ATATCACTGTATCATTCTCAATCACTCGCATATTACATTCACATAATGTATATAAACAAT
GCTTGTTCACAGAACATATTTTTTAAATGCAATTAA
>AA QryID: NC_000913_(+)_1225443_1225670 HitID: NC_011415_(+)_1293947_1294174
rank=1 bitscore=110.0 evalue=3.0E-22 perc_ident=100.0 perc_conserv=100.0 perc_gaps=0.0
query_cvg=100.0 subj_cvg=100.0 alpha=81.81 beta=9 prob=0.9980188316436415
ATGGCGCTGATTCATAGCTTAAAAAATACCCTTGTCAATCAACCCATTGCCGTCGTACTT
TTGATTGTTCTTATTTACGCTTCCTTTTTCCGCACCCTAATCGTGACAGCATCTCCAGAC
CTGTTTAAATATCCAGTATTGAGGCAAGTGTATTTTTTCACCACGCCAGGCTTTCTTAAC
TTTCCATTTTTTGCGGCATCATTAAAGAACAGATACCCGTTATCTTAA
>AA QryID: NC_000913_(-)_11921_12070 HitID: NC_013008_(-)_11938_12087
rank=1 bitscore=94.7 evalue=1.0E-17 perc_ident=97.96 perc_conserv=97.96 perc_gaps=0.0
query_cvg=100.0 subj_cvg=100.0 alpha=69.72 beta=4 prob=0.9980187646224501
TTGCAGACTCACTCACTGCGGTTGACTACTAAATGGGGTCGTAAACCACGTCATCAAGGG
GGAGATGCAAAAATTTTTTGTGCTTTTGCGCAGAAATTTCGCGATATGGCAGGACAAGAC
GTAAAATTGACAGCAATTTTATTCATATAA

>AA QryID: NC_000913_(-)_3948013_3948198 HitID: NC_011415_(-)_4204140_4204325
rank=1 bitscore=107.0 evalue=2.0E-21 perc_ident=100.0 perc_conserv=100.0 perc_gaps=0.0
query_cvg=100.0 subj_cvg=100.0 alpha=100.0 beta=7 prob=0.9980187301855183
GTGAAAAAAATTCAATTTCAGTATGAATGTTGTTCCCTTCCTCGTAGTTCATCCTTTGTC
CGCTGCATTTTTGCGAGCATCATTCCACCGCCAGAACAACTCAACTTCACTTGCACAAAT
AAACAGGAATCAGCCACTAAAATTCACGAATTAGCGGGCTACAGCAGCGATATCTTATGC
ACATAA
>AA QryID: NC_000913_(-)_3663918_3664103 HitID: NC_012947_(+)_245620_245790
rank=1 bitscore=85.5 evalue=7.0E-15 perc_ident=98.21 perc_conserv=98.21 perc_gaps=0.0
query_cvg=91.8 subj_cvg=100.0 alpha=70.7 beta=2 prob=0.9980186983420329
GTGTTGATAAGATATATGTTATATGAATGTTTATTAAATGTAGCGATTACCTTTAATGTC
ACGAAAAGGGATTATTTGCTTACTATTAATTTCCCTGTGTTCTTAAACACGTTTTTGTAT
TATAAATCCAGTCATCCTGCCCGTAGAACATCACACATTATCATCCTGTTCTCCCGCTTC
GTTTAA
>AA QryID: NC_000913_(+)_2734935_2735141 HitID: NC_013941_(+)_3239568_3239774
rank=1 bitscore=103.0 evalue=4.0E-20 perc_ident=95.59 perc_conserv=95.59 perc_gaps=0.0
query_cvg=100.0 subj_cvg=100.0 alpha=71.88 beta=8 prob=0.998018670218997
TTGAGCTGCCGTTTTTTTATTCTGTCAGTTGTGAAACTGAAGCGATTTAGTCGCTATCGA
TCTCATCAAATATGGCTCGCTTTGAGATATTCCTCAAGTAAAAAAACATCTCTTCCTGCG
ATTTCTCACAAAAAAGATTCGTTGACAAAAAGTGACAAAATTATGAGATTTTCATCACAC
ATTTTGACATCAGGAACGGTATGCTGA
>AA QryID: NC_000913_(+)_3416859_3417035 HitID: NC_011748_(+)_3768466_3768642
rank=1 bitscore=108.0 evalue=1.0E-21 perc_ident=100.0 perc_conserv=100.0 perc_gaps=0.0
query_cvg=100.0 subj_cvg=100.0 alpha=76.57 beta=9 prob=0.9980186530265531
TTGCGCCAAAATGTGGCGCATGTTTCATTTTCGCACCATTGCGGGGCGCTGTTTTTATTA
TTTCCTGTCTACACTCAGCTTATTGCGACGCGAAATCGTGCGCAAAAGCTGGCACTACTT
TTGCTTATAAGAGGATGGCCACAGACAGGTAAAAGACGTTTCCCAAACGTCCTATAA
>AA QryID: NC_000913_(-)_3717184_3717336 HitID: NC_012947_(+)_191405_191557
rank=1 bitscore=92.8 evalue=4.0E-17 perc_ident=100.0 perc_conserv=100.0 perc_gaps=0.0
query_cvg=100.0 subj_cvg=100.0 alpha=78.0 beta=3 prob=0.9980185124381105
ATGCCGATATATCTCTTACCACTCCTGTTTTGTAAAGATTTCGAAAATCTAACCTTCTGC
GAAGGCGTAAATCTCTGGCTACAAAAAAGGCAACGGCACTATCATTTTTTAACAATGAAA
CAACAAATTAACCTTGTTTCATTTTCTCCATGA
>AA QryID: NC_000913_(-)_1355177_1355377 HitID: NC_011751_(-)_1609010_1609210
rank=1 bitscore=79.0 evalue=7.0E-13 perc_ident=75.76 perc_conserv=75.76 perc_gaps=0.0
query_cvg=100.0 subj_cvg=100.0 alpha=75.76 beta=2 prob=0.9980184739164875
ATGACACGCACTGCAGCGTGCTTTAATGGGTTTCTCACGATCGTACCGCTCTGCTCCTCG
CTAAGTTGTTCTGTGAGTTATAGTTATAGAGAAATCACGGGTACGAATGTTGAACAGATG
GCGACGTCACCTGTTCAACGACCAACCACACGGCCCTCATTACGACCCCTGAACACAAAC
GGTAATGGCAAATGTCATTAA
>AA QryID: NC_000913_(-)_3199004_3199210 HitID: NC_011745_(-)_3650061_3650267
rank=1 bitscore=119.0 evalue=6.0E-25 perc_ident=100.0 perc_conserv=100.0 perc_gaps=0.0
query_cvg=100.0 subj_cvg=100.0 alpha=83.04 beta=14 prob=0.9980182539821786
ATGGAAAGTGGAACGATAGTAGTGGCATCAGTGTCGCTACGCAAAGCATTCGACATCAAT
CGGAATCCTCTGTGTCAGGTCTTGCCACTGCTTCAAACTTTTACGCCCGTCAAATTGCCC
GTTGCATGGCAATTTGGCGCAAAATACTATCTACTGACAAAAAAGATCGCATTAAGTTCG
CCGTTCATCGTCACTTATGTGACATAA
>AA QryID: NC_000913_(-)_3048697_3048852 HitID: NC_007946_(-)_3228320_3228475
rank=1 bitscore=92.8 evalue=5.0E-17 perc_ident=96.08 perc_conserv=98.04 perc_gaps=0.0
query_cvg=100.0 subj_cvg=100.0 alpha=69.1 beta=5 prob=0.998018125203886
GTGATGATCAATTTTACCTTATGGTTAACAGTCTGTTTCGGTGGTAAGTTCAGGCAAAAG
AGAACGATTGCGTTGGGGACCGGGAGTGGCTCCGATGCTGGGTTTCGTGGTGATAATTTC
ACCATGAAAAAGTTGTCAGCCCCGCTTATTCAATGA
>AA QryID: NC_000913_(+)_3851285_3851443 HitID: NC_011751_(+)_4336482_4336640
rank=1 bitscore=93.2 evalue=3.0E-17 perc_ident=100.0 perc_conserv=100.0 perc_gaps=0.0
query_cvg=100.0 subj_cvg=100.0 alpha=76.84 beta=4 prob=0.9980181118871658
TTGAGTATCGATCACAGTTTGCGTTTTGTCCAAATATTACTGTTTATTTATACAGTAAAC
TTCTATAATATCACTGTACGCAATGTGTTATGCGGGGGCCGCATCGTTACCCGGCGCACT
AAGTCCTGGCTGAAACGGGTGGTGCCGTCAGCGCCTTAA
>AA QryID: NC_000913_(-)_1327180_1327344 HitID: NC_011748_(-)_1470884_1471048
rank=1 bitscore=93.6 evalue=3.0E-17 perc_ident=100.0 perc_conserv=100.0 perc_gaps=0.0
query_cvg=100.0 subj_cvg=100.0 alpha=65.28 beta=5 prob=0.998018012574563

TTGGTTAAACAACGCTTTGGCTCACAGTTTACAACAGGCATGTTAAGCCGTCTCGCAGAG
ATGTTATTGCAGCGAAAGTATGACAAAGTATCCATTTTGCGGTGTTATCGCCCCAGGCTG
ATTGAAATGCCCTTCTGTTTCAGGCATAAAGCCCCAAAGTCATAA
>AA QryID: NC_000913_(+)_344240_344404 HitID: NC_008253_(+)_426652_426816
rank=1 bitscore=76.3 evalue=5.0E-12 perc_ident=79.63 perc_conserv=83.33 perc_gaps=0.0
query_cvg=100.0 subj_cvg=100.0 alpha=79.63 beta=1 prob=0.9980178759238272
TTGTTATTCCACCTGAGAGTGAATCGCAATGCAGGTAATCATTTCATCTGTATATCTGTA
TGCCAACTGGCATTCCATCTCTTTTTTCGCCGATATTTTGTTGTGATCGGCGACACTTCG
CTTAAAAAAGCACCAGTAGTGGTTTCGCAGCCATGCGGTGTATAA
>AA QryID: NC_000913_(-)_2342616_2342765 HitID: NC_017328_(-)_2400305_2400448
rank=1 bitscore=87.8 evalue=1.0E-15 perc_ident=100.0 perc_conserv=100.0 perc_gaps=0.0
query_cvg=95.92 subj_cvg=100.0 alpha=76.96 beta=3 prob=0.998017866526212
GTGAAAATGACCCTCTTGCAAGTGCATAACTTTGTGGATAACTCAGGAAGGAAAAAGTGG
CTTTCGCGCACCTTAGGTCAGACAAGGTGTCCGGGAAAGTCAATGGGAAGAGAAAAATTT
GTTAAAAATAACTGTTCGGCGATTTCTTGA
>AA QryID: NC_000913_(+)_2244933_2245067 HitID: NC_011353_(+)_3023253_3023387
rank=1 bitscore=82.0 evalue=7.0E-14 perc_ident=100.0 perc_conserv=100.0 perc_gaps=0.0
query_cvg=100.0 subj_cvg=100.0 alpha=70.37 beta=1 prob=0.9980171271773565
TTGTCAGCCTTTTTTGATTGCGGCGTAACGATAATGCAAACGATAACAATTATCAATCCA
AATGTTAAATTTTATATCCGCATGATTTGTCAGGGAAATGGAATTGAAAAAGCCCTCTCG
GTTGAGAGGGCTTAG
>AA QryID: NC_000913_(-)_2693580_2693720 HitID: NC_017631_(-)_2929064_2929204
rank=1 bitscore=85.5 evalue=7.0E-15 perc_ident=100.0 perc_conserv=100.0 perc_gaps=0.0
query_cvg=100.0 subj_cvg=100.0 alpha=80.14 beta=2 prob=0.998017009504103
ATGGCAACTTATTACAGCCATTGGCGGCACGCGTTGCTAATTCACGATGGTGATTTTATT
TCCACGCAAACGGTTTCGTCAGCGCATCAGATTCTTTATAATGACGCCCGTTTCCCCCCC
TTGGGTACACCGAAAGCTTAG
>AA QryID: NC_000913_(+)_2040159_2040308 HitID: NC_013941_(+)_2436461_2436610
rank=1 bitscore=87.4 evalue=2.0E-15 perc_ident=97.96 perc_conserv=100.0 perc_gaps=0.0
query_cvg=100.0 subj_cvg=100.0 alpha=69.05 beta=4 prob=0.9980168091214316
ATGACGTGTCTGTATATTAATAAGCTAACCCGCATTGAGTTAACCAATAACGGATTCCAT
ACACAATACGGCCAACAGAGAAAGATACCTGTGCTCACGCCATTGCTTATATTGGCTGGT
TACAATATGCACTATCAATTTTTTAAATAA
>AA QryID: NC_000913_(+)_406425_406622 HitID: NC_017634_(+)_406548_406727
rank=1 bitscore=66.2 evalue=5.0E-9 perc_ident=71.19 perc_conserv=71.19 perc_gaps=0.0
query_cvg=90.77 subj_cvg=100.0 alpha=67.9 beta=2 prob=0.998016774776253
GTGCCACTACAAGGGATCTGCGTCACATTTTTCATAATTCATGTTTTTCTAATAATTAGA
ATATTAAACAATAACAATCCATTACTGGAATCATTTGGAATCTTTACATTATGCCGTGCA
CGTCTGCTGCTACGCTTTTTGTCATTTGTAGCACAAGTAAGTGTCAGCAGTGGTGCTTCA
CACTTGCCCGGTAATTAA
>AA QryID: NC_000913_(+)_2051381_2051539 HitID: NC_008253_(+)_2006300_2006458
rank=1 bitscore=78.2 evalue=1.0E-12 perc_ident=94.23 perc_conserv=98.08 perc_gaps=0.0
query_cvg=100.0 subj_cvg=100.0 alpha=67.29 beta=1 prob=0.998016773553605
ATGCAATGCAGTCTATCAGACTCATCAGGGTGCCATTTTGTGCATATGCGGACTTTTATG
TTTCATATCTCTAACCTGTGGGTCCTCTGCTTAATCCTTAAACAACACCAGCAACTCCTG
CGCTTTCATCTTCCATCGAATTTTTCATGTTGCCGCTAA
>AA QryID: NC_000913_(+)_3079700_3079864 HitID: NC_011740_(+)_2934585_2934749
rank=1 bitscore=88.2 evalue=1.0E-15 perc_ident=98.15 perc_conserv=98.15 perc_gaps=0.0
query_cvg=100.0 subj_cvg=100.0 alpha=98.15 beta=3 prob=0.9980163121638808
TTGACGACAGCGCGTTTTGGGCTACGCCGGAAAATTTGCCAACAATTTACCGCAAGCCGC
GCGTCATGTACATGGAACATCCTTTTGCCGCTTCAGAAATCTCTGGATCATGCTCGCATG
TTGCGCAATCTACTCGCCCGTCCGCTGCGCTTTTCCTTATACTGA
>AA QryID: NC_000913_(-)_3882046_3882246 HitID: NC_011353_(-)_4773360_4773560
rank=1 bitscore=85.5 evalue=8.0E-15 perc_ident=74.24 perc_conserv=74.24 perc_gaps=0.0
query_cvg=100.0 subj_cvg=100.0 alpha=74.24 beta=8 prob=0.9980159984675764
TTGTACACTCCGGAGTCAATTCTCTTTCCTTATTTACCGCGCTTTTCCGCACCTTTTCGC
AGGGAAAATGTACGACCTCACACCAGTGGAAACCAGCATGGCGCGCCGGGTGGAGGATTA
TACGGGCTGATGGGTAAAGCGCAAGGATCGTCCTGGATCTTTATTAGATCGATTAAGCCA
ATTTTTGTCTATGGTCATTAA
>AA QryID: NC_000913_(-)_2783540_2783692 HitID: NC_009800_(-)_2802963_2803115
rank=1 bitscore=90.9 evalue=2.0E-16 perc_ident=100.0 perc_conserv=100.0 perc_gaps=0.0
query_cvg=100.0 subj_cvg=100.0 alpha=100.0 beta=4 prob=0.9980158386061413

GTGCGGTGGGTGGTTTCGAATGAGGGCTGGTGTTTTGGTACGTGGCACGCCACTGCAATG
TATTTATCCATCAGTACTCATTCAAAAGTACCTGGAAAAAATGCCTGCTATGCCAAGCGG
GCAAATTGCTTACATGTGGAATTAAGTAATTAA
>AA QryID: NC_000913_(-)_4056185_4056337 HitID: NC_011748_(-)_4441822_4441974
rank=1 bitscore=88.6 evalue=9.0E-16 perc_ident=100.0 perc_conserv=100.0 perc_gaps=0.0
query_cvg=100.0 subj_cvg=100.0 alpha=73.87 beta=5 prob=0.9980152021270376
TTGCAGAGTCCCTTTGTGATCGCTTTCACGGAGCATAAAAAGGGTTATCCAAAGGTCATT
GCACCAACATGGTGCTTAATGTTTCCATTGAAGCACTATATTGGTGCAACATTCACATCG
TGGTGCAGCCCTTTTGCACGATGGTGCGCATGA
>AA QryID: NC_000913_(-)_2261548_2261691 HitID: NC_011741_(-)_2323850_2323993
rank=1 bitscore=69.7 evalue=4.0E-10 perc_ident=78.72 perc_conserv=78.72 perc_gaps=0.0
query_cvg=100.0 subj_cvg=100.0 alpha=78.72 beta=1 prob=0.998014951231352
GTGCAGCACATCAAACTTTTGCTCATAACTTTACGGCTTTCCTTGCGTGCTGAAACAGAA
GTATTATGCTTTCTTGAAACGTTTCAGCGCGATCTTGTCTTTAACCCTAAGCCAGGTTGG
CGCTTTTTTTCTCATAGAGGCTGA
>AA QryID: NC_000913_(+)_2342663_2342806 HitID: NC_011750_(+)_2460516_2460659
rank=1 bitscore=77.0 evalue=3.0E-12 perc_ident=97.87 perc_conserv=97.87 perc_gaps=0.0
query_cvg=100.0 subj_cvg=100.0 alpha=73.07 beta=1 prob=0.9980145907604905
TTGACTTTCCCGGACACCTTGTCTGACCTAAGGTGCGCGAAAGCCACTTTTTCCTTCCTG
AGTTATCCACAAAGTTATGCACTTGCAAGAGGGTCATTTTCACACTATCTTGCAGTGAAT
CCCAAACATACCCCCTATATATAG
>AA QryID: NC_000913_(+)_728564_728719 HitID: NC_011415_(+)_813178_813333
rank=1 bitscore=81.3 evalue=1.0E-13 perc_ident=100.0 perc_conserv=100.0 perc_gaps=0.0
query_cvg=100.0 subj_cvg=100.0 alpha=83.1 beta=2 prob=0.9980142123875447
TTGCTGAGTTTTGAATACATTGAAAACCGCCAGCGGAAACGCTGGCGGTTTTTTGTATCA
GGAAGATGTTCAGAGGAATATTTAGACATCGTTATACCAATCGAAACATATATTCATGAA
ATATATATAAATATTTTCCTAATTGTTCTTATCTGA
>AA QryID: NC_000913_(+)_4499955_4500098 HitID: NC_011751_(+)_5010044_5010187
rank=1 bitscore=87.8 evalue=1.0E-15 perc_ident=100.0 perc_conserv=100.0 perc_gaps=0.0
query_cvg=100.0 subj_cvg=100.0 alpha=100.0 beta=4 prob=0.9980135628543814
ATGTCTTTAGATCCGCTGGCCTGCGGGCATGACAATGTCAAACAGTCAAAGATTGTGGCG
TTGACGGTCAGTCTTGGGGACAAAAAAGCTCAGTATTCTCGTTGGCAACCAGATAATTTA
ACAGTTGATGCTGAAACGAAATAG
>AA QryID: NC_000913_(+)_660659_660808 HitID: NC_020163_(+)_1381157_1381306
rank=1 bitscore=90.1 evalue=3.0E-16 perc_ident=100.0 perc_conserv=100.0 perc_gaps=0.0
query_cvg=100.0 subj_cvg=100.0 alpha=100.0 beta=5 prob=0.9980134466358079
ATGTCTAATTGGAACGGAACGCTTTTGCTCACCATAATCAACTATTTCAATAGGTTAATC
AATGGGGTGAGTTACGGAGCAAGGCGTGGCACCGCATTTCTGCAAGTGATAAAGAAAATA
TACAAACGCGAAGGAGATGTAAAGCATTAG
>AA QryID: NC_000913_(-)_1218429_1218587 HitID: NC_011751_(-)_1465475_1465618
rank=1 bitscore=64.7 evalue=1.0E-8 perc_ident=87.23 perc_conserv=91.49 perc_gaps=0.0
query_cvg=90.38 subj_cvg=100.0 alpha=63.61 beta=1 prob=0.9980130887073162
TTGTTAATAGTTGCGATGAGGATTATTTCGGTGATATATAAAAACAACCGTGATAACACC
AGGGTTGACAACTATTTGCGCAATAATGCCTTTCCTTCAATGATCCAGGCGTTCACTGCC
AGCAACCCATTCAGCCGCATACTGACAGTGAGAAACTGA
>AA QryID: NC_000913_(+)_3236372_3236509 HitID: NC_012947_(-)_669235_669372
rank=1 bitscore=79.0 evalue=6.0E-13 perc_ident=97.78 perc_conserv=97.78 perc_gaps=0.0
query_cvg=100.0 subj_cvg=100.0 alpha=66.21 beta=3 prob=0.9980130480121164
ATGGTCTGTCATACTTTGTTACCTGCAAAGGGGAGTAACTTCATTGCCGGTCGATCGTCA
TTACGATGTGTGAAAAAACACATCCGGTCACCGGGCAACCCGAAAGGAATACGCAGACGT
ATTCCTTTTTTGTTGTAA
>AA QryID: NC_000913_(+)_1830269_1830439 HitID: NC_010658_(+)_1820800_1820970
rank=1 bitscore=110.0 evalue=2.0E-22 perc_ident=100.0 perc_conserv=100.0 perc_gaps=0.0
query_cvg=100.0 subj_cvg=100.0 alpha=75.68 beta=15 prob=0.9980125764772307
TTGACGAAATTTACTGGAAATTACTGCGCCATTCTGACGCAGCGCGCACCAAAAGCGGGC
ATTTTTTGCGCCATCGTTGACATCATTAACAACCATCGATCAAATCACTTAACAACAGGC
GGTAAGCAACGCGAAATTCTGCTACCATCCACGCACTCTTTATCTGAATAA
>AA QryID: NC_000913_(-)_753897_754055 HitID: NC_011750_(-)_716763_716921
rank=1 bitscore=92.4 evalue=6.0E-17 perc_ident=100.0 perc_conserv=100.0 perc_gaps=0.0
query_cvg=100.0 subj_cvg=100.0 alpha=75.44 beta=8 prob=0.9980120409091624
TTGACAATCATTCAACAAAGTTGTTACAAACATTACCAGGAAAAGCATATAATGCGTAAA
AGTTATGAAGTCGGTATTTCACCTAAGATTAACTTATGTAACAGTGTGGAAGTATTGACC

AATTCATTCGGGACAGTTATTAGTGGTAGACAAGTTTAA
>AA QryID: NC_000913_(+)_2220012_2220170 HitID: NC_017641_(+)_2575738_2575896
rank=1 bitscore=90.5 evalue=2.0E-16 perc_ident=100.0 perc_conserv=100.0 perc_gaps=0.0
query_cvg=100.0 subj_cvg=100.0 alpha=100.0 beta=6 prob=0.9980114668524738
ATGGATTTTTCTTCCTTTGTTGCCCGACGTGGCAGCGAAAATGGTGCAAAAACCGTAGTT
TGCCATAAGCATGATGGAGAGAGAAAAAGAATGCTCAGTTTATTGTCTGAATTTTCAAAA
TATTCACTCGCTGAATTGTTATACAAGGCGCTATTCTAG
>AA QryID: NC_000913_(+)_400225_400428 HitID: NC_011748_(+)_425440_425643
rank=1 bitscore=120.0 evalue=3.0E-25 perc_ident=98.51 perc_conserv=100.0 perc_gaps=0.0
query_cvg=100.0 subj_cvg=100.0 alpha=81.21 beta=20 prob=0.9980101673940256
TTGTTGCAATCTTCTGCTGACAAAGCGTGCAACGTACTGGTGAAGAAAGTGCGTTATCTC
AAAGATGTGCGCAAGATCACAAAAATGATGAACGGGAAGCTAATTTATTCCTGGCTTAAA
TGGCCATGCGGTGAGTTTTTTTCTCTTAATTATAAGTTAACGAAGAGAATATATTTCATA
ACTTTTATTTATAATAAAGGTTGA
>AA QryID: NC_000913_(+)_1524074_1524223 HitID: NC_011353_(+)_1956406_1956555
rank=1 bitscore=84.3 evalue=1.0E-14 perc_ident=100.0 perc_conserv=100.0 perc_gaps=0.0
query_cvg=100.0 subj_cvg=100.0 alpha=100.0 beta=4 prob=0.9980099924285886
ATGATAGTTATCACCATTATTCTTTATGGGTATAAAACTATCGGCGCAGCATCCTACCCG
TTATCGATAAACGATGCAAAACATCCCCTTACAATCCTGAAGGGGATTAATACAACTGAC
GAAAAAATGACAAATCCTTTTGCTGGTTAA
>AA QryID: NC_000913_(+)_4205764_4205904 HitID: NC_009792_(-)_2762980_2763120
rank=1 bitscore=83.2 evalue=3.0E-14 perc_ident=95.65 perc_conserv=95.65 perc_gaps=0.0
query_cvg=100.0 subj_cvg=100.0 alpha=79.03 beta=6 prob=0.9980088398842202
TTGTGGATAACTCTGTGCGTAAAAAGGTATAAAGCGGGCTTTTGCTGGGGAATGCAGCAG
TCAGTCATTTTTCTGCAATTTTTCTATTGCGGCCTGCGGAGAACTCCCTATAATGCGCCT
CCATCGACACGGCGGATGTGA
>AA QryID: NC_000913_(-)_4549380_4549514 HitID: NC_017641_(-)_5114874_5115008
rank=1 bitscore=72.4 evalue=5.0E-11 perc_ident=100.0 perc_conserv=100.0 perc_gaps=0.0
query_cvg=100.0 subj_cvg=100.0 alpha=74.53 beta=1 prob=0.9980087753921844
ATGTCAACCCGAATTCTGAAATTGGTTAACCACATCACAAGAATTTCACTCTCATGGTTG
TTGCTGCAAATTGCAGCAAAGGGTAATCGATGGTTATTGATCAGAGGATGTGACATTCAT
CGCAACAATGGTTGA
>AA QryID: NC_000913_(+)_606618_606767 HitID: NC_011751_(+)_723367_723543
rank=1 bitscore=81.6 evalue=1.0E-13 perc_ident=89.58 perc_conserv=93.75 perc_gaps=0.0
query_cvg=97.96 subj_cvg=82.76 alpha=45.73 beta=5 prob=0.9980085350907948
ATGTTGGCGTTTTCTATTCAGTATAGAAGTCGGAGCGGCTGGGCGAGATGCGGAAGTTCT
GGAATGTTTCTTTTTTTTGGTGATGGTGAACTGATGGTGCCTGAAGCAATTTGGCTACTT
TTGCAATGTGACAAGTTATGGCAGTGCTGA
>AA QryID: NC_000913_(+)_4601017_4601151 HitID: NC_017641_(+)_5148936_5149070
rank=1 bitscore=71.6 evalue=1.0E-10 perc_ident=93.18 perc_conserv=93.18 perc_gaps=0.0
query_cvg=100.0 subj_cvg=100.0 alpha=75.15 beta=2 prob=0.9980085087773359
GTGATAAAAAGCGAATTATCTCTCGCCGCAAACAAACTATCACATGAATATTATCATCAT
AATGAATTTATTGTTTGGCCTTTACGAATCAGGATAATAGATAACCGGGCACGTTTTTTA
ATGTTAACAAGCTAA
>AA QryID: NC_000913_(-)_2496504_2496641 HitID: NC_004431_(-)_2780865_2781002
rank=1 bitscore=78.2 evalue=1.0E-12 perc_ident=93.33 perc_conserv=93.33 perc_gaps=0.0
query_cvg=100.0 subj_cvg=100.0 alpha=71.63 beta=5 prob=0.9980082688800289
ATGACCAGCAGGCCGTTTTTGAGGAAAAACCACAATTTTAAAAGGGGATTGATAGATAAA
TGGTGTATTAGCAATGCATCTCTTGATAATGCACTTCTGGTTAGTAAATTTATTGAAATT
ATTATGTTAATAAAATGA
>AA QryID: NC_000913_(+)_395649_395843 HitID: NC_017641_(+)_432527_432721
rank=1 bitscore=119.0 evalue=4.0E-25 perc_ident=100.0 perc_conserv=100.0 perc_gaps=0.0
query_cvg=100.0 subj_cvg=100.0 alpha=83.0 beta=20 prob=0.9980078822121314
ATGCCTGCATTTGTGATCCTGCGCGTGTTAATCCGCTTATGTAAACAATCTTTGGTTTGT
GAAATCCGTCGAAACAATTCTTATGGTCAGGCTGGCGAACTAAGCGCCTTGCTATGGGTC
ACAATGGGCGGTTTCATTTGGCTGACGCTTTGTAGCGGTCATGCGGTTAATACACAGCAG
TTGTTAAAACGATAA
>AA QryID: NC_000913_(-)_4518385_4518507 HitID: NC_017644_(-)_4483690_4483812
rank=1 bitscore=72.0 evalue=5.0E-11 perc_ident=97.5 perc_conserv=100.0 perc_gaps=0.0
query_cvg=100.0 subj_cvg=100.0 alpha=67.72 beta=2 prob=0.9980078407770367
ATGGGGGAACAGCGGGCGATCGCTCAAATGGTATGCTGGTTTATTTCATGTAAAAGAATA
AAATATCTGTATAAAATCATGCCACTGGCAATCTGCATCAACTCTCCGGGTAAACAGGGT

TAA
>AA QryID: NC_000913_(+)_989687_989809 HitID: NC_017641_(+)_1090479_1090601
rank=1 bitscore=77.8 evalue=7.0E-13 perc_ident=100.0 perc_conserv=100.0 perc_gaps=0.0
query_cvg=100.0 subj_cvg=100.0 alpha=68.09 beta=4 prob=0.998007651751599
ATGCCACCTTTCTGGTCGATGGCATTACGCGGTGTATGTTATAAAAATGTAGCAATAAAG
GCGTTTGTACCTGAAAAGATGAAGATTCTGCATAGCGCGATTTACGCAACAGGAATAGAC
TGA
>AA QryID: NC_000913_(+)_4273151_4273285 HitID: NC_017652_(+)_4649511_4649645
rank=1 bitscore=67.8 evalue=1.0E-9 perc_ident=86.36 perc_conserv=88.64 perc_gaps=0.0
query_cvg=100.0 subj_cvg=100.0 alpha=54.17 beta=3 prob=0.9980075383048329
TTGCAGAACACAAAAATGAAACACTCTGTTTGTTTCATTAATTTTGTGAACTATATCACA
ATTGATTGTTTGTTAGCCATATTAGGCCGTGACTTTTATTGCTGTACAGATTATGTGGTT
TTTCAGTGGCATTAA
>AA QryID: NC_000913_(+)_2411322_2411483 HitID: NC_011741_(+)_2455821_2455982
rank=1 bitscore=95.9 evalue=5.0E-18 perc_ident=100.0 perc_conserv=100.0 perc_gaps=0.0
query_cvg=100.0 subj_cvg=100.0 alpha=78.84 beta=11 prob=0.9980071371949051
ATGCCATTGGCTGAAAATTACGCAAAATGGCATAGACTCAAGATATTTCTTCCATCATGC
AAAAAAAAATTTGCAGTGCATGATGTTAATCATAAATGTCGGTGTCATCATGCGCTACGCT
CTATGGCTCCCTGACGTTTTTTTAGCCACGTATCAATTATAG
>AA QryID: NC_000913_(+)_2459063_2459236 HitID: NC_011083_(+)_2546901_2547041
rank=1 bitscore=53.9 evalue=2.0E-5 perc_ident=67.39 perc_conserv=78.26 perc_gaps=0.0
query_cvg=80.7 subj_cvg=100.0 alpha=40.53 beta=3 prob=0.9980070068082852
GTGACCGAAATCACACTTAAAAATGATCTAAAACAAAATTCACCCGAATCCATGAGTGCG
CCACCTCCAAATTTTGCCAGCTGGATCGCGTTTCTTAGATCATATTTGAAAAAAGATAGA
AACATACTTGCAACATTCCAGCTGGTCCGACCTATACTCTCGCCACTGGTCTGA
>AA QryID: NC_000913_(-)_395603_395725 HitID: NC_013008_(-)_461336_461458
rank=1 bitscore=69.3 evalue=3.0E-10 perc_ident=100.0 perc_conserv=100.0 perc_gaps=0.0
query_cvg=100.0 subj_cvg=100.0 alpha=66.25 beta=1 prob=0.9980062354471697
TTGTTTCGACGGATTTCACAAACCAAAGATTGTTTACATAAGCGGATTAACACGCGCAGG
ATCACAAATGCAGGCATTGGTGAGCAGGAAAAAGATCGTGTTTACGCGCCGTTACGTTTT
TAA
>AA QryID: NC_000913_(+)_655550_655657 HitID: NC_009801_(+)_679000_679107
rank=1 bitscore=69.3 evalue=3.0E-10 perc_ident=100.0 perc_conserv=100.0 perc_gaps=0.0
query_cvg=100.0 subj_cvg=100.0 alpha=68.71 beta=1 prob=0.9980058585545412
TTGATTGCATTTTGTCATCAAAAAAGACTTGGTTTTTCTTTTTTGACTATTCCCATCGCA
GAAAACGACGCATCATCTTTAATCGATGCGCGGAAATATTTAACTTGA
>AA QryID: NC_000913_(-)_4470595_4470732 HitID: NC_020163_(-)_471019_471156
rank=1 bitscore=81.3 evalue=1.0E-13 perc_ident=100.0 perc_conserv=100.0 perc_gaps=0.0
query_cvg=100.0 subj_cvg=100.0 alpha=70.16 beta=6 prob=0.998005658890217
ATGTCTGCAATTATTGATACCGAAGGACAGTTCCCCTGCAGAATCACATCAAATAAAAAT
GCATATACCTTGACTTTTAATTCAAATAAACCGTTTGCGCTGACAAAATATTGCATCAAA
TGCTTGCGCCGCTTCTGA
>AA QryID: NC_000913_(-)_1785190_1785357 HitID: NC_017631_(-)_1941762_1941929
rank=1 bitscore=100.0 evalue=2.0E-19 perc_ident=100.0 perc_conserv=100.0 perc_gaps=0.0
query_cvg=100.0 subj_cvg=100.0 alpha=100.0 beta=12 prob=0.9980054738048598
ATGAAAACAGCATTTCATTTTTATGGTTTCGTTTATACCGATGGTTTATGTGGAAATTGT
CGAAGAGAGCAGATTTGCGCAACGCTGGGATCAGTCTTAAAAAGTAAAAAAATATATTTG
CTTGAACGATTCACCGTTTTTTTCATCCGGTTAAATATGCAAAGATAA
>AA QryID: NC_000913_(-)_1108356_1108514 HitID: NC_011741_(-)_1150952_1151110
rank=1 bitscore=99.8 evalue=3.0E-19 perc_ident=100.0 perc_conserv=100.0 perc_gaps=0.0
query_cvg=100.0 subj_cvg=100.0 alpha=100.0 beta=12 prob=0.9980052325337423
GTGCTTATATCGATCCGATCTATTTTAATGGATAACCAGCCATTCCGACAACAGAATCCC
GGTATCGTTCAGCACGCTGGCTCATGGATTAAGCACCTGATTGCAACCGTTTTCACTTTC
CGTGCTTCTCCTAAAGACAGAAGCATCGACTTCGTGTAG
>AA QryID: NC_000913_(-)_1438818_1438949 HitID: NC_009801_(-)_1549695_1549826
rank=1 bitscore=70.5 evalue=1.0E-10 perc_ident=97.67 perc_conserv=97.67 perc_gaps=0.0
query_cvg=100.0 subj_cvg=100.0 alpha=72.61 beta=2 prob=0.9980050861915521
TTGATCAAAACATTCATTACGCTGATGTGGGGGACACAAAAGCGAAAATGCAGAAGAAAG
CCATTTGCTAAAATTGAAAGATTACTACTGGGCGCGCAGCAATTTCGTGCGCCCCTCATT
TGCGCAATGTAA
>AA QryID: NC_000913_(+)_4259385_4259510 HitID: NC_009800_(+)_4293106_4293231

rank=1 bitscore=72.0 evalue=4.0E-11 perc_ident=97.56 perc_conserv=97.56 perc_gaps=0.0
query_cvg=100.0 subj_cvg=100.0 alpha=67.88 beta=3 prob=0.9980048363950997
TTGGGTTCTTTTTCTCTATCAATACTATTAGCAGGGAGATATATCACCAGAGTTTAATG
TGTGATTTTTTATTTATCGTCGAACCTGGATTGTTTATCATTGGCCTTAACAAAGTTAAC
GGCTAA
>AA QryID: NC_000913_(-)_4173106_4173267 HitID: NC_011415_(-)_4444654_4444815
rank=1 bitscore=87.0 evalue=2.0E-15 perc_ident=100.0 perc_conserv=100.0 perc_gaps=0.0
query_cvg=100.0 subj_cvg=100.0 alpha=100.0 beta=7 prob=0.9980044707852118
GTGCTCAACAATGCGCTCCTGCTAAACCATAATTCTTTTTATCAGATGGAATATCTGTCA
CATTGCTTTTCAACGATAGCTTCCTGGCAGAGATTTTTTCTTATTATTCCTCCCCATCTG
GTGTTACCCTCCTGCCCATTAACCCATTCAACAGAACTGTGA
>AA QryID: NC_000913_(+)_1864616_1864717 HitID: NC_009800_(+)_1884569_1884670
rank=1 bitscore=68.2 evalue=7.0E-10 perc_ident=100.0 perc_conserv=100.0 perc_gaps=0.0
query_cvg=100.0 subj_cvg=100.0 alpha=69.99 beta=1 prob=0.9980042880788149
ATGACACCGCGCTTACAGCCAAATGAATTTAGCGTTACTGGCGAGCCTGGTCTTTACATT
AATTATGCAAAATTTATGGATGAGTTGTTGATATGCCATTGA
>AA QryID: NC_000913_(+)_2902141_2902272 HitID: NC_017641_(+)_3330679_3330810
rank=1 bitscore=80.1 evalue=2.0E-13 perc_ident=100.0 perc_conserv=100.0 perc_gaps=0.0
query_cvg=100.0 subj_cvg=100.0 alpha=69.79 beta=6 prob=0.9980042804909965
ATGGACTATTGCTATTCGGTTTATCCCCGCTGGCGCGGGGAACACGGGCGCACGGAATAC
AAAGCCGTGTATCTGCTCGGTTTATCCCCGCTGGCGCGGGGAACACTGGCTCTGCAACAG
CAGCACCCATGA
>AA QryID: NC_000913_(+)_3245475_3245606 HitID: NC_017641_(+)_3733911_3734042
rank=1 bitscore=73.6 evalue=2.0E-11 perc_ident=100.0 perc_conserv=100.0 perc_gaps=0.0
query_cvg=100.0 subj_cvg=100.0 alpha=100.0 beta=2 prob=0.998003032729557
ATGCGCCTGTTTGTCTCGTCGGTAAGCGAAAAGTATAAAGTGTCAGCCTGTGTAAATCCT
CTCGCCACCCTCCCCTGCATTCAGCAAAATCAGACTCCACGGACATGTAATTTTGATAAC
GAACAACGTTGA
>AA QryID: NC_000913_(+)_3667392_3667511 HitID: NC_017628_(+)_4049046_4049165
rank=1 bitscore=68.9 evalue=3.0E-10 perc_ident=92.31 perc_conserv=94.87 perc_gaps=0.0
query_cvg=100.0 subj_cvg=100.0 alpha=74.28 beta=3 prob=0.9980029303177347
ATGGCATATCATGATTGCGGCGAGAGCAGGATTGAATGTTCTGAAAATGAAAAACAACAC
CAAATCGGTATTTTTATAAATTCCATTGATATTAGTGCGTTATATTTTTTGACAGGTTAA
>AA QryID: NC_000913_(+)_341955_342092 HitID: NC_010498_(+)_367651_367788
rank=1 bitscore=69.7 evalue=4.0E-10 perc_ident=97.78 perc_conserv=97.78 perc_gaps=0.0
query_cvg=100.0 subj_cvg=100.0 alpha=97.78 beta=1 prob=0.9980028596995525
ATGGGGAACGTTGCTGCTCTGTTTCATTCAGGCTCGCGGCATGTTGGGGCTGGATCGGGT
GGTTTAAATATTGGCTATATTCAATGGACGCGTTTTGCCGCGATGACATATCAGGCGTTG
CCAAATACACATAGCTAA
>AA QryID: NC_000913_(-)_3677283_3677405 HitID: NC_010498_(-)_3899875_3899997
rank=1 bitscore=70.9 evalue=1.0E-10 perc_ident=97.5 perc_conserv=97.5 perc_gaps=0.0
query_cvg=100.0 subj_cvg=100.0 alpha=73.41 beta=3 prob=0.9980027118100279
TTGCGCTGTGGTGGTTTGCTGGTCAACCATATTAAAACAGTGTTCCATTATTTTTTTGAG
CCAGAACGTATTCCTGAAAGATTCCGTTGTGGAGTGAGGAAATTTACCCCGGCCAGGCGG
TAA
>AA QryID: NC_000913_(-)_3103540_3103674 HitID: NC_017641_(-)_3539039_3539173
rank=1 bitscore=75.5 evalue=7.0E-12 perc_ident=100.0 perc_conserv=100.0 perc_gaps=0.0
query_cvg=100.0 subj_cvg=100.0 alpha=100.0 beta=3 prob=0.9980024499299693
ATGATGGGATTCTATCAACTTCGCGGACTGGTTTTTTGTTACCTGTGGCAAATAATTGCA
AACATTCCCAATTTGCAAAACGAAATTGTGATGTGGATAACATTTTTGCCCCTGAGCATC
GTCAGGGGCGGTTAA
>AA QryID: NC_000913_(-)_655330_655476 HitID: NC_009800_(-)_692188_692334
rank=1 bitscore=58.5 evalue=9.0E-7 perc_ident=79.17 perc_conserv=79.17 perc_gaps=0.0
query_cvg=100.0 subj_cvg=100.0 alpha=79.17 beta=1 prob=0.9980019736362398
ATGCTCATATTAGATTATTTTTTGAGCGCACAGCCGCAGCTAACACCATTGCAATTAACA
AATTTGCATCAATCCACCATCAATTTGCACACATTATTATGTGATAATTGCCAACCGCTA
AATATGCGTTTTGTTATCTATGTATAA
>AA QryID: NC_000913_(-)_642553_642741 HitID: NC_002695_(-)_724684_724872
rank=1 bitscore=92.0 evalue=9.0E-17 perc_ident=100.0 perc_conserv=100.0 perc_gaps=0.0
query_cvg=100.0 subj_cvg=100.0 alpha=83.8 beta=11 prob=0.9980017800122893
ATGCACTCGCATCCTCTTTACGCAAAATCAGCAATTTTTCCTGGTTCTCACTGTTCACTT
TTGCTGCAAAATTATTCCGTTTCGTTTGTGGGAATAACCATGGCAGAAGCATTTTACATC

CTGATAGGATTTCTGATTATGGCGGCGATTATCGTCATGGCTGTCCTTTACCTCGAAAAC
CACTCCTGA
>AA QryID: NC_000913_(+)_3705917_3706072 HitID: NC_009801_(+)_4020723_4020878
rank=1 bitscore=77.4 evalue=2.0E-12 perc_ident=98.04 perc_conserv=98.04 perc_gaps=0.0
query_cvg=100.0 subj_cvg=100.0 alpha=73.38 beta=6 prob=0.9980016920519956
GTGAAAACGTCAATACAGCCAACCGGGATTTACACCAACGGTGAGAATCCACACACAAAG
ATTAAAAAAACTTCAAACAGCTATTTGCAGCAGAGAAATTTGTGCTACTCCAACATGACC
AGAACAATCAGCTTAATATTTAGCAACATTTGGTGA
>AA QryID: NC_000913_(-)_2786856_2786990 HitID: NC_009792_(-)_3729899_3730045
rank=1 bitscore=59.3 evalue=5.0E-7 perc_ident=78.05 perc_conserv=87.8 perc_gaps=0.0
query_cvg=93.18 subj_cvg=85.42 alpha=45.9 beta=1 prob=0.9980015018460815
ATGAGATGTAGGGTGACATGGCGATGCTCATTTCGTAGCCATAATCTAAAAATATCTACA
TTTCTGAAAAATGCGCACAAAAGCGACATATTGTTTTCTTATTGTGATCAAAAGCAACAA
ATTAGTAACATTTAA
>AA QryID: NC_000913_(-)_471655_471795 HitID: NC_011353_(-)_542080_542220
rank=1 bitscore=77.0 evalue=2.0E-12 perc_ident=97.83 perc_conserv=97.83 perc_gaps=0.0
query_cvg=100.0 subj_cvg=100.0 alpha=73.15 beta=6 prob=0.9980014108935578
ATGGTTCTGCTACACGAAGAAGGTATTGCAAGCGGTGTGCCAGAAATGAAAAACGAGAAA
GAGAGCAGGAAGTTAAGAAGTTTGAAAATGAAAACGCACTATGACAGTGCACAGTGCGTT
ACATTTTTGCACCAACGATGA
>AA QryID: NC_000913_(-)_3728871_3729008 HitID: NC_011415_(-)_3975453_3975590
rank=1 bitscore=68.2 evalue=1.0E-9 perc_ident=97.78 perc_conserv=100.0 perc_gaps=0.0
query_cvg=100.0 subj_cvg=100.0 alpha=97.78 beta=1 prob=0.9980012637882983
ATGACCGAGATCTTACTTTTGTTGCGCAATTGTACTTATTGCATTTTTCTCTTCGAGGAA
TTACCCAGTTTCATCATTCCATTTTATTTTGCGAGCGAGCGCACACTTGTGAATTATCTC
AATAGCAGTGTGAAATAA
>AA QryID: NC_000913_(+)_3752565_3752777 HitID: NC_017635_(+)_3956991_3957203
rank=1 bitscore=124.0 evalue=2.0E-26 perc_ident=100.0 perc_conserv=100.0 perc_gaps=0.0
query_cvg=100.0 subj_cvg=100.0 alpha=75.99 beta=25 prob=0.9980008426104051
ATGTGCTGTCTGGTTTTTCAACAGGACGAGGTTTATCATATTGAAATAGTGGAACTATTG
GCCAAACTAATGAATAACTCCAGTAAAACATCCACAGTACAGATTAAGCGTATTAAACCT
TCAATTATCTACCGTTTATTGCTGATTGGCCTCGGATCACCAATGGTGATTTACGGCCTG
GTTCGCCCGCTCACCATCGAAACGCGAGATTAA
>AA QryID: NC_000913_(-)_1229754_1229864 HitID: NC_009801_(-)_1321212_1321322
rank=1 bitscore=66.6 evalue=2.0E-9 perc_ident=97.22 perc_conserv=97.22 perc_gaps=0.0
query_cvg=100.0 subj_cvg=100.0 alpha=74.49 beta=2 prob=0.9980006175196339
GTGCTCACGTGGCCTTCTGGCGACGACGCTCATCCAGCAGAAATGAAAAATATCACCCGG
CTAAAAAATAGAATAGAAGCATTCGCCATAATGACATTAAACATTGTTTGA
>AA QryID: NC_000913_(+)_2031510_2031617 HitID: NC_009801_(+)_2163070_2163177
rank=1 bitscore=61.2 evalue=7.0E-8 perc_ident=91.43 perc_conserv=91.43 perc_gaps=0.0
query_cvg=100.0 subj_cvg=100.0 alpha=69.32 beta=1 prob=0.9980002786986362
TTGGATGAGCCAGTCAAGACTCAATCATTTTTTGAACAAATAGATAAATTTTGCGCAACG
GCAGTGAATACAGGAAAAATCGTTTTGAGCGATTTTATTTATCTTTGA
>AA QryID: NC_000913_(-)_4090871_4091023 HitID: NC_011415_(-)_4354556_4354708
rank=1 bitscore=68.2 evalue=1.0E-9 perc_ident=100.0 perc_conserv=100.0 perc_gaps=0.0
query_cvg=100.0 subj_cvg=100.0 alpha=100.0 beta=1 prob=0.9980000098216022
ATGAATAACCCATTAATGATTCATGTGGTTTATTTAAATAACCATTATGTGCATTACTCC
GCAAATCTGACCTTTCACTCTGTCTCAAATTGTCATTGTTTACCGACATATCGCCTCTCA
TCACTTAATCGCTCCACCATTACAATTGATTAA
>AA QryID: NC_000913_(+)_2033695_2033838 HitID: NC_012947_(-)_1801523_1801666
rank=1 bitscore=78.2 evalue=1.0E-12 perc_ident=100.0 perc_conserv=100.0 perc_gaps=0.0
query_cvg=100.0 subj_cvg=100.0 alpha=70.65 beta=7 prob=0.9979996680712581
GTGAATAATATTCAACACCAGTGCAGCATCATCATTACATACTTACAGCCGCCGACTACC
GCTTTCGATATATTGACTCAATTTTCCAGCCTGGTTAAAAATAGCGACCAGCTCAGTCGC
AAATATAGTGACTACCCTAACTAA
>AA QryID: NC_000913_(+)_1797111_1797239 HitID: NC_017641_(+)_2103263_2103391
rank=1 bitscore=67.8 evalue=8.0E-10 perc_ident=100.0 perc_conserv=100.0 perc_gaps=0.0
query_cvg=100.0 subj_cvg=100.0 alpha=100.0 beta=1 prob=0.9979996574039421
GTGTCCAGTCTGGCTGCGACAATTACACCTGATGAGACAGGCTTTTTATTTTTCAAAACG
CGCATACAAAAAAAGCCTCCACTGGGAGGCTTTCAGGCGCTGTTTTCCGTTTCTCTTCTC
ACGCGCTAG
>AA QryID: NC_000913_(+)_4422883_4423017 HitID: NC_017644_(+)_4571303_4571422

rank=1 bitscore=56.2 evalue=4.0E-6 perc_ident=87.18 perc_conserv=87.18 perc_gaps=0.0
query_cvg=88.64 subj_cvg=100.0 alpha=82.23 beta=2 prob=0.9979992819676866
ATGATAAAGAAGTTGATGGTGACCATTTCTGATGCAGTTGTTCAAAAAAACACCATGATG
AAGTGTGATGAACTTCAAATCAGCGTGTTAGAGGTTAATTGCGAAAGGGGAGATTTATTT
CGGCTCTGCCCTTGA
>AA QryID: NC_000913_(-)_4048807_4048947 HitID: NC_017646_(-)_4612142_4612282
rank=1 bitscore=73.2 evalue=3.0E-11 perc_ident=97.83 perc_conserv=97.83 perc_gaps=0.0
query_cvg=100.0 subj_cvg=100.0 alpha=97.83 beta=4 prob=0.9979991781961254
ATGCATCGCAGAATTTTCTCTGCTATTTCGCATAAAGACTATCGGCAATGTCTCACAAAG
CGTATGGCTTTTACTTACAACGCTCACGCCGAAAAATCCGCCTTTTATTATGGTTCAGGT
ATAATCGCCACAAAGCTTTGA
>AA QryID: NC_000913_(-)_4447687_4447833 HitID: NC_017651_(-)_4836367_4836492
rank=1 bitscore=71.6 evalue=9.0E-11 perc_ident=97.56 perc_conserv=97.56 perc_gaps=0.0
query_cvg=85.42 subj_cvg=100.0 alpha=71.75 beta=8 prob=0.9979991302967417
ATGAAAAATGCGGCTACGGTTATGGATTTTCCTGCTCTGTATACCGTCTTAAAACTGGTG
AAAAAGGAAAATGAAGACGAAAACAAGCGAAGACATTCGGCGCGAGTTGGCTATAATACT
CGGCACTTGTTTGCCACATATTTTTAA
>AA QryID: NC_000913_(+)_1298495_1298650 HitID: NC_017635_(+)_1390700_1390855
rank=1 bitscore=81.3 evalue=1.0E-13 perc_ident=100.0 perc_conserv=100.0 perc_gaps=0.0
query_cvg=100.0 subj_cvg=100.0 alpha=100.0 beta=7 prob=0.9979987983269877
ATGCTCCGTTTACTTCGTCATTATTTTTACTTTGTTCCCGCGCAGTTATCAAAAGCAAAA
GGAATAGGTAAAAATATTCTTCTCAAATTACAGTTAGTTATAAGGATTTCCTTAACTGCT
TCTCCTCACCATCATGTTATTTTCGCCACATCATAA
>AA QryID: NC_000913_(+)_2042772_2042921 HitID: NC_012947_(-)_1789764_1789913
rank=1 bitscore=73.6 evalue=2.0E-11 perc_ident=100.0 perc_conserv=100.0 perc_gaps=0.0
query_cvg=100.0 subj_cvg=100.0 alpha=100.0 beta=4 prob=0.9979984896993503
ATGAACGCCATCACCATTTTCCCCTCGATTATAAAACTTGAGTTATTCAGTAGTCTCCCC
TCTTGCAACTCACACCCAAAACTGCCTAACGAAAAGTTATTAATTTTCAATCATATTGCT
ATCAGTATTTACATTTTTTTCGCTGTGCTAG
>AA QryID: NC_000913_(-)_522152_522274 HitID: NC_011415_(-)_566506_566628
rank=1 bitscore=62.8 evalue=2.0E-8 perc_ident=100.0 perc_conserv=100.0 perc_gaps=0.0
query_cvg=100.0 subj_cvg=100.0 alpha=72.76 beta=1 prob=0.9979982795559654
ATGGATAATATCCATTCAACGCATATGATTTATGATTTTATTAGTCTGAGTTATTTTTGT
AGGGCTATATTTAAAATCAATAAACTCCACCGATTCAGATTAATATCAGCAATAGAAAGA
TAG
>AA QryID: NC_000913_(+)_3809787_3809909 HitID: NC_011741_(+)_3882447_3882569
rank=1 bitscore=65.9 evalue=3.0E-9 perc_ident=100.0 perc_conserv=100.0 perc_gaps=0.0
query_cvg=100.0 subj_cvg=100.0 alpha=100.0 beta=1 prob=0.9979981699496556
GTGAACTCTCGATTGCCAGGCCCAAATGCCAAACCCGAGATTCTCAAAGGTGGCGTAGTA
TACGCTGACTCAGCGATGTGCTCAAGTCCCGAACAGACAAAGATCCCGAAGGATCGCGCA
TAG
>AA QryID: NC_000913_(-)_2428801_2428938 HitID: NC_016856_(-)_2527947_2528102
rank=1 bitscore=67.4 evalue=2.0E-9 perc_ident=82.22 perc_conserv=86.67 perc_gaps=0.0
query_cvg=100.0 subj_cvg=88.24 alpha=50.1 beta=5 prob=0.9979979606727243
ATGCCGCAAAGGCATAAAAAGTCGATGGCGTTGAATATTTTTTCAGCGCCATTTTTATTG
ATGCGCGGGAAGGAAATCCCTACGCAAACGTTTTCTTTTTCTGTTAGAATGCGCCCCGAA
CAGGATGACAGGGCGTAA
>AA QryID: NC_000913_(+)_3204297_3204431 HitID: NC_009801_(+)_3535941_3536075
rank=1 bitscore=73.6 evalue=3.0E-11 perc_ident=100.0 perc_conserv=100.0 perc_gaps=0.0
query_cvg=100.0 subj_cvg=100.0 alpha=74.74 beta=6 prob=0.9979976037761628
GTGATAAGCGCAGTGTATTTCGCAAAACATTGCCATTCCCTGCTCTGGCGCAATATTCAT
TCTTATTTTCGCGTTGATTCACGAACTGTTAATTACATATTCACGGTGGCAAAAAATATA
AAACCACATTTTTAG
>AA QryID: NC_000913_(-)_4244482_4244601 HitID: NC_017663_(-)_4555892_4556011
rank=1 bitscore=63.5 evalue=1.0E-8 perc_ident=97.44 perc_conserv=100.0 perc_gaps=0.0
query_cvg=100.0 subj_cvg=100.0 alpha=97.44 beta=1 prob=0.9979975638337861
ATGTGCGCATCTCCACATTACCGCCAATTCTGTAACAGAGATCACACAAAGCGACGGTGG
GGCGTAGGGGCAAGGAGGATGGAAAGAGGTTGCCGTATAAAGAAACTAGAGTCCGTTTAG
>AA QryID: NC_000913_(-)_2920217_2920342 HitID: NC_017651_(-)_3085547_3085672
rank=1 bitscore=81.3 evalue=7.0E-14 perc_ident=100.0 perc_conserv=100.0 perc_gaps=0.0
query_cvg=100.0 subj_cvg=100.0 alpha=61.73 beta=10 prob=0.9979975241676802
ATGCCAAAATCGGGCCTCATTGAACGCATTAATGTTGTGTTGTTGCACGGTGAGCCGCTA

TGGCGCGCTTTTTATACTGCTATTGCCAGATATAAACACGCGCCGTATTCGGCGAACGAC
CTATAA
>AA QryID: NC_000913_(+)_2967444_2967605 HitID: NC_017641_(+)_3395995_3396156
rank=1 bitscore=95.9 evalue=5.0E-18 perc_ident=100.0 perc_conserv=100.0 perc_gaps=0.0
query_cvg=100.0 subj_cvg=100.0 alpha=80.23 beta=15 prob=0.9979974207830294
ATGGAAACAGTAGTGGAGGTTTTTCACAGTTATCCCAGCTTTCTGTGGATAACATGGTGT
AAGATCCTGTTTATTTTCAGTGACCAGATTTGGAAAACCCGTTGCAGTGTTGCGCAACTC
GATTACCGGCAACCTAAAAAGCAATATAAATCAGAGAATTGA
>AA QryID: NC_000913_(-)_4044745_4044987 HitID: NC_009801_(-)_4371935_4372177
rank=1 bitscore=143.0 evalue=4.0E-32 perc_ident=98.75 perc_conserv=100.0 perc_gaps=0.0
query_cvg=100.0 subj_cvg=100.0 alpha=76.49 beta=35 prob=0.997997162262257
ATGTCCGTGCCTGTTTATCAGATTATGGGTAAAGGATGCCACAGACAGGATGAAAACATG
AGTTTTTCACCGCTTTTCGTGAAAATCTTTTGTAGATCTTCTGGATCGCTCGCAAATCGT
CATGTGGATAACTTTGTGCATAAATTATTTACATCGCTTTTAATTTATTATCATGCTTTT
TACAGCATGATGTTTGTCTTTGTTTTTCATGCAGTTAAAAATAAATCCATCCCTAATAAT
TGA
>AA QryID: NC_000913_(-)_2149055_2149165 HitID: NC_009800_(-)_2195272_2195382
rank=1 bitscore=64.3 evalue=1.0E-8 perc_ident=100.0 perc_conserv=100.0 perc_gaps=0.0
query_cvg=100.0 subj_cvg=100.0 alpha=100.0 beta=1 prob=0.9979971486538753
GTGGAAGTTAATCATTATCAGGCCGAAATTGAATTGTATCCTGATAAAATTGCATTATCA
TTTTTATTCTTGAGTGGGTTTTCCCCATATAGAAATAATTTTAATGGTTAG
>AA QryID: NC_000913_(-)_1525588_1525734 HitID: NC_009801_(-)_1627539_1627685
rank=1 bitscore=78.6 evalue=9.0E-13 perc_ident=100.0 perc_conserv=100.0 perc_gaps=0.0
query_cvg=100.0 subj_cvg=100.0 alpha=100.0 beta=7 prob=0.9979970316795543
ATGCAAAGTCAAACCAATATTCATAATGTCTTTTTATTCACAACAATAAATAATTCAATA
ACAATCATTGAAATGAATGATGGTTTGTATAAGGAATATACGCTACCGCCCCACCCTGAT
AACATGATCTGCACATTGCCATCTTAA
>AA QryID: NC_000913_(-)_3939573_3939686 HitID: NC_013008_(-)_4794132_4794245
rank=1 bitscore=62.4 evalue=4.0E-8 perc_ident=97.3 perc_conserv=97.3 perc_gaps=0.0
query_cvg=100.0 subj_cvg=100.0 alpha=97.3 beta=1 prob=0.9979969553735206
GTGCCGCTTTGCTTTTTCTCAGCGGCGCGGGGTGTGCATAATACGCTTTCCCGCTACAGA
GTCAAGCATTTATTTTTGCTTTCTCTGCCGGAGTTCTCAGGAGAACCCCGCTGA
>AA QryID: NC_000913_(-)_312591_312710 HitID: NC_020163_(-)_1025499_1025618
rank=1 bitscore=69.7 evalue=2.0E-10 perc_ident=100.0 perc_conserv=100.0 perc_gaps=0.0
query_cvg=100.0 subj_cvg=100.0 alpha=72.13 beta=5 prob=0.9979968946075191
ATGATGAGATGCGATATGCTCCTCAGCACCAATCAACGCCCGGACTGGGCCAGTCTTGAG
ATCAGACTCATTCCCGGAACTGACTTCAGGCGACATATATCAATGACGTTGGTGCAATAA
>AA QryID: NC_000913_(+)_3752494_3752613 HitID: NC_013008_(+)_4554950_4555069
rank=1 bitscore=56.6 evalue=2.0E-6 perc_ident=87.18 perc_conserv=92.31 perc_gaps=0.0
query_cvg=100.0 subj_cvg=100.0 alpha=87.18 beta=1 prob=0.9979966843273071
ATGAAATGCCATGATGCAGTGCAATTTCCTTATTTATATAACGACATATTGTTTTTTATA
TACTTTCTTAAATGTGCTGTCTGGTTTTTCAACAGGACGAGGTTTATCATATTGAAATAG
>AA QryID: NC_000913_(-)_3265702_3265839 HitID: NC_009801_(-)_3602557_3602694
rank=1 bitscore=64.3 evalue=2.0E-8 perc_ident=100.0 perc_conserv=100.0 perc_gaps=0.0
query_cvg=100.0 subj_cvg=100.0 alpha=77.72 beta=3 prob=0.9979961406741116
ATGCTTATTTTCCTCGCAGGGTTCTTTCGCACTATCAAAAAACGCTCATTTACCCGGCGT
TACAGTAAACGAAGCAACATTCTGTGGCCTACGCCGATCAAAAGCTCTGTGAAAATTTCC
CTTGTTGTCTTTGTCTGA
>AA QryID: NC_000913_(+)_3698294_3698410 HitID: NC_017641_(+)_4190385_4190501
rank=1 bitscore=62.0 evalue=5.0E-8 perc_ident=100.0 perc_conserv=100.0 perc_gaps=0.0
query_cvg=100.0 subj_cvg=100.0 alpha=77.54 beta=2 prob=0.9979960892309463
TTGCCGCTGATTCTACGATTGCTTTTTACTGTCAGCACCCGGCAATCTTCCTCATTGCGC
GATGCCTTCCAGCTTAATTTCTTTATTTGCAATAGTTGCAGTTCATATCCGGGCTGA
>AA QryID: NC_000913_(-)_3628688_3628828 HitID: NC_017641_(-)_4120489_4120602
rank=1 bitscore=57.8 evalue=2.0E-6 perc_ident=100.0 perc_conserv=100.0 perc_gaps=0.0
query_cvg=80.43 subj_cvg=100.0 alpha=90.22 beta=4 prob=0.9979959308338656
GTGTTTCATTTACGGCCAGGAATGCTGATGATGACAACATTACTACCCGTTTTCACTAAG
CCTTCCCCGTTGGCGCTCAATGCTCTGCGCGCTGGTCGTATTTGCCGTTTCCTTCTTATC
CCGGATGGAAGAATCCGCTGA
>AA QryID: NC_000913_(-)_1031203_1031343 HitID: NC_017652_(-)_1042400_1042540
rank=1 bitscore=70.1 evalue=3.0E-10 perc_ident=97.83 perc_conserv=97.83 perc_gaps=0.0
query_cvg=100.0 subj_cvg=100.0 alpha=97.83 beta=5 prob=0.9979959266475051

TTGCGACACCGGCAGGACACCGGGCGCTTATGGTCGGCAAGCGACGTCCCTGTAGCAAGG
CAAACCATCGCTTCACGTGAGATGCTGAAAACGAAAGCTCATCCTTCTGCACTGGCGCAC
GTCGCCAGAAAGTATTGTTAA
>AA QryID: NC_000913_(+)_2683633_2683809 HitID: NC_008253_(+)_2691007_2691183
rank=1 bitscore=78.6 evalue=9.0E-13 perc_ident=100.0 perc_conserv=100.0 perc_gaps=0.0
query_cvg=100.0 subj_cvg=100.0 alpha=100.0 beta=8 prob=0.9979959019576108
TTGACAAAGGAATTTACGCAAACGATTACCTTCAGGCTACGCAAGGCTTTGGAGAATAAA
GAGCTTGCAACCGGAAACGGATTTCTTTTCAGGTTTGTGATGCAAATTTTTCACTTCATC
ACATTCTTTCTGAAAAACACCAAAGAACCATTTACATTGCAGGGCTATTTTTTATAA
>AA QryID: NC_000913_(-)_4091036_4091146 HitID: NC_009801_(-)_4420895_4421005
rank=1 bitscore=59.3 evalue=3.0E-7 perc_ident=100.0 perc_conserv=100.0 perc_gaps=0.0
query_cvg=100.0 subj_cvg=100.0 alpha=73.5 beta=1 prob=0.9979958171295622
TTGCCCAATGTGCCAGAGATGCCTCCGGCACAATATTTTAAATTTCGATTTGAAAAATTA
AACATATTACATCTCCCGGTAAGTTATATTTCCCTGATACATTGTGAGTAA
>AA QryID: NC_000913_(+)_2627565_2627711 HitID: NC_012947_(-)_1245735_1245881
rank=1 bitscore=61.2 evalue=1.0E-7 perc_ident=100.0 perc_conserv=100.0 perc_gaps=0.0
query_cvg=100.0 subj_cvg=100.0 alpha=100.0 beta=1 prob=0.9979956910344902
TTGGGATTATCAAAGCGGCAGATATTCTTTCATCTTAAATTTTACGTCTTTATCCTGACT
GATGTTTATCCTGTTTGGCTGCGAAATAAATATAAAATTAATATATATGTTGTAATGATA
TATTTTTATAAATTATTCCCTGCGTGA
>AA QryID: NC_000913_(-)_4033296_4033409 HitID: NC_013008_(-)_4794132_4794245
rank=1 bitscore=59.7 evalue=2.0E-7 perc_ident=97.3 perc_conserv=97.3 perc_gaps=0.0
query_cvg=100.0 subj_cvg=100.0 alpha=97.3 beta=1 prob=0.99799569039389
GTGCCGCTTCGCTTTTTCTCAGCGGCGCGGGGTGTGCATAATACGCCTTCCCGCTACAGA
GTCAAGCATTTATTTTTGCTTTCTCTGCCGGAGTTCTCAGGAGAACCCCGCTGA
>AA QryID: NC_000913_(-)_4164424_4164537 HitID: NC_013008_(-)_4794132_4794245
rank=1 bitscore=59.7 evalue=2.0E-7 perc_ident=97.3 perc_conserv=97.3 perc_gaps=0.0
query_cvg=100.0 subj_cvg=100.0 alpha=97.3 beta=1 prob=0.99799569039389
GTGCCGCTTCGCTTTTTCTCAGCGGCGCGGGGTGTGCATAATACGCCTTCCCGCTACAGA
GTCAAGCATTTATTTTTGCTTTCTCTGCCGGAGTTCTCAGGAGAACCCCGCTGA
>AA QryID: NC_000913_(+)_1114743_1114847 HitID: NC_009801_(+)_1192984_1193088
rank=1 bitscore=63.5 evalue=1.0E-8 perc_ident=100.0 perc_conserv=100.0 perc_gaps=0.0
query_cvg=100.0 subj_cvg=100.0 alpha=100.0 beta=2 prob=0.9979956696562573
GTGAACATAAGAAGAAAAGATAAGCACACTAATTATGCGCCCGACTTCCAGGGGGCGCAA
TCCAGAGAGCTTTTATCGCTAAATCAGGGGGATTTGCTGTGGTAA
>AA QryID: NC_000913_(-)_812337_812474 HitID: NC_017328_(-)_768901_769038
rank=1 bitscore=82.8 evalue=5.0E-14 perc_ident=100.0 perc_conserv=100.0 perc_gaps=0.0
query_cvg=100.0 subj_cvg=100.0 alpha=100.0 beta=10 prob=0.9979956579511406
TTGCATTTATCTAACCAGTTAAAAAATAAAAGATATTTCTTTGAGCCGTCTTTAACGCCA
ATTCGCGTGCAAGCCGCGTATTCTCTCGCTTGCCTCGTGTTTTCTAACTCTAATACACAT
GGTTATCCACAGGAATAG
>AA QryID: NC_000913_(+)_2212755_2212883 HitID: NC_011353_(+)_2891223_2891351
rank=1 bitscore=62.0 evalue=5.0E-8 perc_ident=97.62 perc_conserv=100.0 perc_gaps=0.0
query_cvg=100.0 subj_cvg=100.0 alpha=97.62 beta=2 prob=0.9979956176068143
GTGAGTACGATCACTCAAAATCGCCTGGCAAAAATAAAATCACCCTATAGATGCACAAAA
AACGGGCAAAACTACCTGGTTCGCAAAACTGCGTCTAAAGTTAAACCGGGACCTCGCGAG
CAAGGGTGA
>AA QryID: NC_000913_(-)_237140_237289 HitID: NC_009800_(-)_236738_236887
rank=1 bitscore=58.2 evalue=1.0E-6 perc_ident=97.96 perc_conserv=97.96 perc_gaps=0.0
query_cvg=100.0 subj_cvg=100.0 alpha=74.13 beta=1 prob=0.9979955846812237
TTGATAACGGACTCTAATTATATACCTCACTTAAATAATAACAAACAAAATCAATTTGAA
ATATACATTCATTTAATCAATATAAATTTATTCTCTAGAAATATATTTAAGCTGAATGGT
TCGATATCATTATTGAATTTTAATGAATGA
>AA QryID: NC_000913_(+)_4537763_4537891 HitID: NC_011353_(+)_5468295_5468423
rank=1 bitscore=59.3 evalue=3.0E-7 perc_ident=97.62 perc_conserv=97.62 perc_gaps=0.0
query_cvg=100.0 subj_cvg=100.0 alpha=97.62 beta=1 prob=0.9979954781285105
ATGTTCGTAAGCTATATCACTTACTCAATCCATTTTACCCAGAGTCATAAAATAGAATGT
CTGTTGATCTCATTTTCATATTCCTTTCAGACTGAAGTGCGTTTTCATCACCCGATGAGA
AAGAACTGA
>AA QryID: NC_000913_(+)_1391003_1391128 HitID: NC_017656_(+)_1706598_1706723
rank=1 bitscore=70.5 evalue=1.0E-10 perc_ident=90.24 perc_conserv=90.24 perc_gaps=0.0
query_cvg=100.0 subj_cvg=100.0 alpha=63.09 beta=9 prob=0.997995474844487

ATGGCGACAGATGCTGATTATGATACCGAAAACGGGTTTGAACGTGCGAAGCCCGAACGT
AGTGTTCGGAGTTCTATGTGCTTTACCGCATTTTGGAGACTATTATTTACACTAAATCTG
ATTTGA
>AA QryID: NC_000913_(-)_2986272_2986403 HitID: NC_017635_(-)_3178351_3178482
rank=1 bitscore=64.3 evalue=1.0E-8 perc_ident=100.0 perc_conserv=100.0 perc_gaps=0.0
query_cvg=100.0 subj_cvg=100.0 alpha=71.19 beta=4 prob=0.9979954521575259
ATGTTGCAACGTAAAACTTTACGTAAATTAACATGGTTAACATTTATGCCACTATTGTTT
GTAAATTCATATTTCGTAATGCTTCTGAATTTTTCGTGTGATGGTTTTAAATACTATGGT
GTTTACTCTTGA
>AA QryID: NC_000913_(+)_3838114_3838233 HitID: NC_017625_(-)_43552_43671
rank=1 bitscore=70.9 evalue=1.0E-10 perc_ident=100.0 perc_conserv=100.0 perc_gaps=0.0
query_cvg=100.0 subj_cvg=100.0 alpha=72.37 beta=7 prob=0.9979953757733274
GTGGAATATAAAGTCTGGCACTTCTTACTGACCACGCAAGCGCGTTTCGTACAGCATGAT
GAGAGCGATGAAAGCAAACTACACTTATGCTTCATTCGATACACATTTGTAAAAGGTTGA
>AA QryID: NC_000913_(+)_3882239_3882346 HitID: NC_017663_(+)_4192046_4192153
rank=1 bitscore=58.9 evalue=4.0E-7 perc_ident=100.0 perc_conserv=100.0 perc_gaps=0.0
query_cvg=100.0 subj_cvg=100.0 alpha=100.0 beta=1 prob=0.9979949593598629
GTGTACAATTATTACAATCCGGCCTCTTTAATCACCCATGGCTTCGGTGTCCATCGTTTC
ATTTTTCGGCGGATATCCAATAAAGCCATTGAATTTATTCAAGTTTAG
>AA QryID: NC_000913_(-)_4077032_4077136 HitID: NC_009800_(-)_4105917_4106021
rank=1 bitscore=63.2 evalue=2.0E-8 perc_ident=100.0 perc_conserv=100.0 perc_gaps=0.0
query_cvg=100.0 subj_cvg=100.0 alpha=77.83 beta=4 prob=0.9979949104902236
ATGAATTATGGGTGCTATGAATATATTATATTCAGAGATATTTATTTGCATAATCTGAAT
GGTGTAGCCGAAAAACGTCAGCATCCTAACAGCACCAGGATTTAA
>AA QryID: NC_000913_(+)_3769793_3769885 HitID: NC_009801_(+)_4084932_4085024
rank=1 bitscore=60.8 evalue=1.0E-7 perc_ident=100.0 perc_conserv=100.0 perc_gaps=0.0
query_cvg=100.0 subj_cvg=100.0 alpha=100.0 beta=2 prob=0.9979948601353199
GTGTTGTTTTTGGGCTGGCAGCCAGAAGGGAGTCAGGCTGATATTTTGACAATAATCCGG
GTTCGCGATTCTCGCCATAACACCAAAGAATAA
>AA QryID: NC_000913_(-)_3984173_3984310 HitID: NC_011353_(-)_4882887_4883024
rank=1 bitscore=67.8 evalue=1.0E-9 perc_ident=100.0 perc_conserv=100.0 perc_gaps=0.0
query_cvg=100.0 subj_cvg=100.0 alpha=82.11 beta=6 prob=0.9979948298779326
ATGATAAAATTCATTCAATTAATAACACATATATTAATTGCCGTTAAAACTAAAAACAGC
ATCAATAATCAACGCGATATAATAAACCTGCCTTACATATCAACTGCGCCAGAGGTAGGA
TTGAAAACGCTCTCCTGA
>AA QryID: NC_000913_(+)_1276867_1277085 HitID: NC_017625_(-)_2600616_2600834
rank=1 bitscore=140.0 evalue=1.0E-31 perc_ident=100.0 perc_conserv=100.0 perc_gaps=0.0
query_cvg=100.0 subj_cvg=100.0 alpha=78.77 beta=36 prob=0.9979948172898279
ATGAGCAATAACCTTAATGAATGTGACGATACATTCTGGAATGGCAGTATTCTCGGCTAT
TGGCTGAAGTATACCCATACCCGGAAAGAGTTACTCCTTATTTGCCGTGTGGTTAGTCGC
TTTACATCGGTAAGGGTAGGGATTTTACAGCACCGTGAAAAATCTCATAATTTTTATGAA
GTCACTGTACTCACTATGGGTAATGATAAATATCAATGA
>AA QryID: NC_000913_(-)_453441_453572 HitID: NC_009800_(-)_520282_520413
rank=1 bitscore=74.7 evalue=7.0E-12 perc_ident=100.0 perc_conserv=100.0 perc_gaps=0.0
query_cvg=100.0 subj_cvg=100.0 alpha=81.3 beta=9 prob=0.9979948059821977
ATGAAGTTTACTGATTTAAAGATTGAAACACAGGCCTCGCATCAGTGTTTCTTTTACCAG
GGGCGATTATTTCGTCAACATGATGAATTTACATGCCTTACCCACTTCCCCTCGCCGTTG
GCAATGTTATGA
>AA QryID: NC_000913_(-)_1891885_1892016 HitID: NC_017646_(-)_2112791_2112922
rank=1 bitscore=58.9 evalue=4.0E-7 perc_ident=88.37 perc_conserv=90.7 perc_gaps=0.0
query_cvg=100.0 subj_cvg=100.0 alpha=89.23 beta=4 prob=0.9979947641207033
GTGTTATCCAGTAAGATGGAGAAATTTAACGGTCAGTCCGCACGCTATAACCAGATTGTT
CAGCTAAGAAAGAAAACCAGAGTTGCTAATCAAAGGGCGAACACAACGGCATGTTTATGT
TTATTTATATAG
>AA QryID: NC_000913_(-)_1160807_1160977 HitID: NC_010658_(+)_2019961_2020131
rank=1 bitscore=93.2 evalue=4.0E-17 perc_ident=100.0 perc_conserv=100.0 perc_gaps=0.0
query_cvg=100.0 subj_cvg=100.0 alpha=77.04 beta=17 prob=0.9979947455482483
TTGCTGCCGTATATATCGCCATTATTCCCATTTCTGCCTGCGAATGCGCTGAAAAGACAT
TCTCTGTTTGGTTTACATGACAAATATGAATGCGTATATTTCTCATTTGCATTTACAAAC
AAAATTATTCGCACACTAAATATAATCGCTCATCACCCTCCTCCGGCATGA
>AA QryID: NC_000913_(+)_3904656_3904778 HitID: NC_009801_(+)_4223402_4223524

rank=1 bitscore=70.9 evalue=9.0E-11 perc_ident=100.0 perc_conserv=100.0 perc_gaps=0.0
query_cvg=100.0 subj_cvg=100.0 alpha=73.04 beta=8 prob=0.997994728086394
TTGCCTGCGAATGCAGTAACAATCCAGTCATTTATTAATGGTTTTTATAACGAACATCCA
GGTTCGGAAATTAATTTAATTGCGTGCAGAGAAAATAGCAATGCGCTATTGATAAAAATA
TGA
>AA QryID: NC_000913_(-)_1993622_1993750 HitID: NC_009801_(-)_2122419_2122547
rank=1 bitscore=74.3 evalue=9.0E-12 perc_ident=100.0 perc_conserv=100.0 perc_gaps=0.0
query_cvg=100.0 subj_cvg=100.0 alpha=100.0 beta=8 prob=0.9979946815094807
TTGTTTTTTGAGCGTGATATCGGCAGTGCTATAAAATACGTTAATATAATGACCAATAAA
TATTTTTATCATGAATGTTTTTTGCCCGTATTGTTCGGGTTAATTAATGTTACATATTCA
GCGGGCTGA
>AA QryID: NC_000913_(+)_3399217_3399405 HitID: NC_011748_(+)_3750824_3751012
rank=1 bitscore=108.0 evalue=9.0E-22 perc_ident=100.0 perc_conserv=100.0 perc_gaps=0.0
query_cvg=100.0 subj_cvg=100.0 alpha=100.0 beta=22 prob=0.9979946175583984
GTGCAGTTCGTTTCCTTTGTTACAAATCTCTGCCTGAGTCCAGAAAGGCTTAATGCATCA
GCAGCATTCCTCGCCTGTTTGCAACCGCGTAAGGTCATTCATCTGCATATGTGCTGCAAC
AATCTGGCGAGCAGACAAGCACACTCCCATGAGACGCAGCGCGCATTATTCTACGTGAAA
ACGGATTAA
>AA QryID: NC_000913_(+)_3717222_3717371 HitID: NC_017651_(+)_4029288_4029437
rank=1 bitscore=53.1 evalue=3.0E-5 perc_ident=67.35 perc_conserv=67.35 perc_gaps=0.0
query_cvg=100.0 subj_cvg=100.0 alpha=67.35 beta=3 prob=0.9979946120633316
TTGTTAAAAAATGATAGTGCCGTTGCCTTTTTTGTAGCCAGAGATTTACGCCTTCGCAGA
AGGTTAGATTTTCGAAATCTTTACAAAACAGGAGTGGTAAGAGATATATCGGCATTTATC
AGATCGTTAGGTGGCTATAAGTCACGCTAA
>AA QryID: NC_000913_(+)_2925778_2925909 HitID: NC_017644_(+)_2893347_2893478
rank=1 bitscore=62.0 evalue=5.0E-8 perc_ident=90.7 perc_conserv=93.02 perc_gaps=0.0
query_cvg=100.0 subj_cvg=100.0 alpha=95.35 beta=5 prob=0.9979946080541888
ATGTTTCTTACCACCCTCACGCGACAAATATCATCACAGTTAATATGTCATACAATTTAT
GTTGCAACGCAAACGTTTCCCTATTTTCATAAACCGTATTTTATCGCTATGAAAAAGAAA
TTATCGCCATGA
>AA QryID: NC_000913_(-)_1976252_1976350 HitID: NC_009801_(-)_2105825_2105923
rank=1 bitscore=59.3 evalue=3.0E-7 perc_ident=100.0 perc_conserv=100.0 perc_gaps=0.0
query_cvg=100.0 subj_cvg=100.0 alpha=100.0 beta=2 prob=0.997994535583796
GTGGTTCTGCTTATTGCAGCTTATCGCAACTATTCTAATGCTAATTATTTTTTACCGGGG
CTTCCCGGCGACATCACGGGGTGCGGTGAAACCGCATAA
>AA QryID: NC_000913_(-)_1986004_1986117 HitID: NC_017631_(-)_2135278_2135391
rank=1 bitscore=55.1 evalue=5.0E-6 perc_ident=94.59 perc_conserv=100.0 perc_gaps=0.0
query_cvg=100.0 subj_cvg=100.0 alpha=75.81 beta=1 prob=0.9979945277641877
TTGATTATCATTTTTATAACATTAAAGGGGTATTTATGTGATTTGTATCACATAAAGATT
GATTTTTGTGATGGTTGTCATATTATAAGCGCAAAGACAATAACACCTGTATAA
>AA QryID: NC_000913_(+)_347728_347826 HitID: NC_009801_(+)_384584_384682
rank=1 bitscore=55.5 evalue=4.0E-6 perc_ident=96.88 perc_conserv=96.88 perc_gaps=0.0
query_cvg=100.0 subj_cvg=100.0 alpha=96.88 beta=1 prob=0.9979945119671679
ATGAAACGCGGTGAAACATTGCCTGAAACGTTAACTGAAACGCATATTTGCGGATTAGTT
CATGACTTTATCTCTAACAAATTGAAATTAAACATTTAA
>AA QryID: NC_000913_(-)_770163_770267 HitID: NC_009800_(-)_797793_797897
rank=1 bitscore=58.9 evalue=4.0E-7 perc_ident=100.0 perc_conserv=100.0 perc_gaps=0.0
query_cvg=100.0 subj_cvg=100.0 alpha=100.0 beta=2 prob=0.9979944610036118
GTGAGTTATAAGGCTGAAATTAGTTTCGGTTGGCAATTAAAGATAAAAAGAGACGAAGCA
AAAACAGCACGGTTGACCTTAATTCTTTTTTTGGTCACTGGCTAA
>AA QryID: NC_000913_(-)_2083584_2083697 HitID: NC_010498_(+)_1066986_1067099
rank=1 bitscore=54.3 evalue=1.0E-5 perc_ident=94.59 perc_conserv=97.3 perc_gaps=0.0
query_cvg=100.0 subj_cvg=100.0 alpha=94.59 beta=1 prob=0.9979944551417748
ATGAACATTTGGCTCCGGTTTTTTTATTCTTTTGTATCGAACTCCTCATTAATAACCATT
TGTTCTAATTTAGAGAATTTGGTTATTGGTCTGCATTCCACCAGTCATGAATAA
>AA QryID: NC_000913_(+)_2817278_2817394 HitID: NC_017646_(+)_3174125_3174241
rank=1 bitscore=69.3 evalue=3.0E-10 perc_ident=100.0 perc_conserv=100.0 perc_gaps=0.0
query_cvg=100.0 subj_cvg=100.0 alpha=75.26 beta=8 prob=0.9979943784322741
ATGGCATTACATTCTGTTAAACCTAAGTTTAGCCGATATACACAACTTCAACCTGACTTT
ATCGTTGTCGATAGCGTTAATGCGAATGCCGTGAAGCGAGTCCACGGCATTGCCTGA
>AA QryID: NC_000913_(+)_3210888_3211010 HitID: NC_013008_(+)_4011521_4011643

rank=1 bitscore=74.3 evalue=9.0E-12 perc_ident=100.0 perc_conserv=100.0 perc_gaps=0.0
query_cvg=100.0 subj_cvg=100.0 alpha=100.0 beta=9 prob=0.9979942871968177
GTGCCGAAGAGCGATCGGGAAGCCCCCGACAGCCGCACTGAGAGGCAGCGGCAAATATAT
AAGTACGCCCTCGTAATTATCGTTGGCGGTAAACAACCGTTGGATTTCAGCGTTAACGGC
TGA
>AA QryID: NC_000913_(+)_1525601_1525714 HitID: NC_017641_(+)_1767634_1767747
rank=1 bitscore=54.3 evalue=9.0E-6 perc_ident=97.3 perc_conserv=97.3 perc_gaps=0.0
query_cvg=100.0 subj_cvg=100.0 alpha=97.3 beta=1 prob=0.997994237785609
GTGCAGATCATGTTATCAGGGTGGGGCGGTAGCGTATATTCCTTATACAAACCATCATTC
ATTTCAATGATTGTTATTGAATTATTTATTGTTGTGAATAAAAAGACATTATGA
>AA QryID: NC_000913_(+)_1349078_1349176 HitID: NC_009801_(+)_1482781_1482879
rank=1 bitscore=57.4 evalue=1.0E-6 perc_ident=100.0 perc_conserv=100.0 perc_gaps=0.0
query_cvg=100.0 subj_cvg=100.0 alpha=100.0 beta=2 prob=0.9979942188344448
TTGTTGATGCTTGTTGTGCCTGAAAATCAGGCGATTCGTCGTTTTAGTAAACAGTACGAA
CAGATAAACGGTTATTATAATCAACCTGGCTGTGAGTAG
>AA QryID: NC_000913_(-)_1948736_1948831 HitID: NC_009800_(-)_1969883_1969978
rank=1 bitscore=57.4 evalue=1.0E-6 perc_ident=100.0 perc_conserv=100.0 perc_gaps=0.0
query_cvg=100.0 subj_cvg=100.0 alpha=100.0 beta=2 prob=0.9979942188344448
GTGCCTCACACCTTAATGCGATTAGCCGATGGCGGAACGACAAATTTTGTTAATTTTTAT
GAGTTCGGGCAACACCTTTATAGCAAACTGGCGTAG
>AA QryID: NC_000913_(-)_3245636_3245749 HitID: NC_010498_(-)_3477676_3477792
rank=1 bitscore=54.7 evalue=8.0E-6 perc_ident=97.22 perc_conserv=97.22 perc_gaps=0.0
query_cvg=97.3 subj_cvg=94.74 alpha=93.35 beta=1 prob=0.9979941905181845
ATGCACTACCGCAATGTTATTGCTGTTTTGGTATGTTCTGCGGGTTTGATACATCAACAC
AGCCTGAACTATTCCCTGTTAAATCGTGAGATAAGAAAGCATTCAAAAACATAA
>AA QryID: NC_000913_(-)_3698275_3698436 HitID: NC_009800_(-)_3729723_3729884
rank=1 bitscore=90.5 evalue=2.0E-16 perc_ident=100.0 perc_conserv=100.0 perc_gaps=0.0
query_cvg=100.0 subj_cvg=100.0 alpha=100.0 beta=16 prob=0.997994188718062
TTGCGCATTTCACTACCAGTCTGGAGTCAGCCCGGATATGAACTGCAACTATTGCAAATA
AAGAAATTAAGCTGGAAGGCATCGCGCAATGAGGAAGATTGCCGGGTGCTGACAGTAAAA
AGCAATCGTAGAATCAGCGGCAAGCCGGAAAGGTTCCGGTGA
>AA QryID: NC_000913_(+)_986529_986657 HitID: NC_011415_(+)_1071255_1071410
rank=1 bitscore=62.8 evalue=3.0E-8 perc_ident=95.12 perc_conserv=95.12 perc_gaps=0.0
query_cvg=97.62 subj_cvg=80.39 alpha=96.43 beta=2 prob=0.9979941046731353
TTGTTTCTGATGGAAACTTTATCGACCTGGCACAAAATCTTCTTTCAGACATCCAGAATG
CCAAAAAATAGTATGAAAATTGTGCTATTAGCTAAGAAAAAACTAATCCGCAATAAGAAT
GCGGATTAG
>AA QryID: NC_000913_(-)_3268064_3268180 HitID: NC_020163_(-)_4036264_4036380
rank=1 bitscore=70.5 evalue=1.0E-10 perc_ident=97.37 perc_conserv=97.37 perc_gaps=0.0
query_cvg=100.0 subj_cvg=100.0 alpha=74.26 beta=10 prob=0.9979940968370145
ATGACTGTCCACGACGCTATACCCAAAAGAAAGCGGCTTATCGGTCAGTTTCACCTGGTT
TACGTAAAAACCCGCTTCGGCGGGTTTTTGCTTTTGGAGGGGCAGAAAGATGAATGA
>AA QryID: NC_000913_(+)_3117285_3117467 HitID: NC_017652_(+)_3404748_3404948
rank=1 bitscore=92.4 evalue=7.0E-17 perc_ident=96.67 perc_conserv=96.67 perc_gaps=0.0
query_cvg=100.0 subj_cvg=90.91 alpha=100.0 beta=16 prob=0.997994023337484
ATGTCTATCTGGCTATTTTTACGAAATTTCAACAAACAAAAAATTAACAACATTTCACAA
CGTAACTATATAAATTTGTTGGTAGTTTTAACGATTAAGATTGCAAAAGCAACAGATAGA
ACGGTGTTTGCTCATCAAGCGAACATAATTAATACAGACTTGTTTTGCGTGATAAAAAAA
TAG
>AA QryID: NC_000913_(-)_3267951_3268067 HitID: NC_017641_(-)_3756388_3756504
rank=1 bitscore=68.6 evalue=5.0E-10 perc_ident=97.37 perc_conserv=97.37 perc_gaps=0.0
query_cvg=100.0 subj_cvg=100.0 alpha=65.74 beta=10 prob=0.9979939789939309
ATGACTGTCCACGACACTATACCCAAAAGAAAGCGGCTTATCGGTCAGTTTCACCTGTTT
TACGTAAAAACCCGCTTCGGCGGGTTTTTACTTTTGGAGGGGCAGAAAGATGAATGA
>AA QryID: NC_000913_(+)_29474_29611 HitID: NC_011353_(+)_33849_33986
rank=1 bitscore=72.0 evalue=8.0E-11 perc_ident=100.0 perc_conserv=100.0 perc_gaps=0.0
query_cvg=100.0 subj_cvg=100.0 alpha=69.18 beta=11 prob=0.997993968139657
ATGCATGAGCCACAAAATAATATAAAAAATCCCGCCATTAAGTTGACTTTTAGCGCCCAT
ATCTCCAGAATGCCGCCGTTTGCCAGAAATTCGTCGGTAAGCAGATTTGCATTGATTTAC
GTCATCATTGTGAATTAA
>AA QryID: NC_000913_(+)_4311075_4311179 HitID: NC_017660_(-)_398603_398707

rank=1 bitscore=53.5 evalue=2.0E-5 perc_ident=100.0 perc_conserv=100.0 perc_gaps=0.0
query_cvg=100.0 subj_cvg=100.0 alpha=100.0 beta=1 prob=0.9979939676370273
ATGCTGGCGACTTCACAAAAAAATAAAATTTATAAAGTTTATTTTATTGTGAACAATGGC
GAGTGGCTTAAAATTTTATTTCATTTAATTAGTTTAAAAACATGA
>AA QryID: NC_000913_(-)_3978790_3978888 HitID: NC_009800_(-)_4009180_4009278
rank=1 bitscore=55.1 evalue=5.0E-6 perc_ident=96.88 perc_conserv=100.0 perc_gaps=0.0
query_cvg=100.0 subj_cvg=100.0 alpha=78.27 beta=2 prob=0.9979939412426332
ATGCTTTCCGGTGTTACCGGTTATCGTTATGGGTACATCGAGTGTTGCAAATGTTTTCGT
AATTCAGGAGAAATGGCAAATAAAGCATTAAAAATTTGA
>AA QryID: NC_000913_(-)_3999300_3999434 HitID: NC_016825_(+)_104704_104838
rank=1 bitscore=53.1 evalue=4.0E-5 perc_ident=100.0 perc_conserv=100.0 perc_gaps=0.0
query_cvg=100.0 subj_cvg=100.0 alpha=100.0 beta=1 prob=0.9979939202073584
ATGCTTAAAATATCGGACAGTTCGGTGGGCACACAGAAACAAATTGGGTTTCAGACCATT
AAACAGCCTGACTCAGCGCGACGGGAAAAATTGAGGTCGCTGACAACCGCTAAGGCTATC
AGCAAAAAGGGATAG
>AA QryID: NC_000913_(+)_2354758_2354883 HitID: NC_010468_(-)_1539659_1539784
rank=1 bitscore=85.1 evalue=4.0E-15 perc_ident=100.0 perc_conserv=100.0 perc_gaps=0.0
query_cvg=100.0 subj_cvg=100.0 alpha=69.58 beta=17 prob=0.997993887156836
ATGTGGTTACCGGGCATTTTTGCGTACACGATTCCGTGCCCAATGTATGCGTTGCAACGC
AGTGAAAATTCCTCTGAAAACGTCTCGCAAAGGCTGAAACTGGCAGATGTCAAAGGCCTG
GGATAA
>AA QryID: NC_000913_(-)_4538190_4538327 HitID: NC_011741_(-)_4600787_4600948
rank=1 bitscore=74.7 evalue=1.0E-11 perc_ident=97.73 perc_conserv=97.73 perc_gaps=0.0
query_cvg=97.78 subj_cvg=83.02 alpha=100.0 beta=8 prob=0.997993877149425
GTGAAACATCATCCTGAAAACACCAACATCAACAAGCCTCTCCAGATCGACTTCAGAAGT
GACCAGTTACAAGCCACAAACCAGGAAACATATTTCATGATGAGAATTATGCTCAGCCTG
ATGGCGGGTAAACACTGA
>AA QryID: NC_000913_(+)_4554677_4554820 HitID: NC_010658_(-)_4461173_4461316
rank=1 bitscore=73.6 evalue=2.0E-11 perc_ident=97.87 perc_conserv=97.87 perc_gaps=0.0
query_cvg=100.0 subj_cvg=100.0 alpha=100.0 beta=11 prob=0.9979938687237806
ATGGCTGATTATGCGGAAATAAATAATTTCCCACCGGAATTAAGTAGCAGCGGTGACAAG
TATTTTCATCTACGTAACTATTCGGAATATTCAGAATATACTAGCGGTTTTTTTTTGAGT
TTGATGATTTTTATCAAATCATGA
>AA QryID: NC_000913_(+)_3544473_3544565 HitID: NC_009800_(+)_3590792_3590884
rank=1 bitscore=54.3 evalue=1.0E-5 perc_ident=100.0 perc_conserv=100.0 perc_gaps=0.0
query_cvg=100.0 subj_cvg=100.0 alpha=100.0 beta=2 prob=0.9979938616100085
GTGGGTATCAGTCGTGGTGCCGCAATATCTCTGTTCCCGATTGGGATAATTAGAGTTTGT
CGTCAGAAAATTGACGTTACCCATAACAAATGA
>AA QryID: NC_000913_(+)_1710310_1710510 HitID: NC_017664_(+)_1855149_1855349
rank=1 bitscore=103.0 evalue=2.0E-20 perc_ident=100.0 perc_conserv=100.0 perc_gaps=0.0
query_cvg=100.0 subj_cvg=100.0 alpha=80.53 beta=24 prob=0.9979938479768912
ATGCAGGTTAATTGTTTTTTTAATAGCGAAATTTTCTATTCATTCGTGATCAAGATCACG
ATGCTGTCGCAATGTAAAAACATTGCAAGAATCAGGTTAAAATCATTTAAATTTACACAC
GCAACAAATATTGACCTACAAAACATTACACTGGCTATTTTTCAGAAACTGGACTATCTC
ACTAGTAAAAACGCTAAATAG
>AA QryID: NC_000913_(+)_931342_931455 HitID: NC_017641_(+)_994695_994808
rank=1 bitscore=67.0 evalue=1.0E-9 perc_ident=100.0 perc_conserv=100.0 perc_gaps=0.0
query_cvg=100.0 subj_cvg=100.0 alpha=100.0 beta=8 prob=0.9979938465704294
ATGACGATTTTGTTAACAATTTGTGCAATCGGCAGCATCGATAAGCAGGTCAAATTCTCC
CGTCATTATCACCTCTGCTACTTAAATTTCCCGCTTTATAAGCCGATTAAATGA
>AA QryID: NC_000913_(-)_1777428_1777604 HitID: NC_018661_(+)_2406738_2406914
rank=1 bitscore=95.9 evalue=5.0E-18 perc_ident=100.0 perc_conserv=100.0 perc_gaps=0.0
query_cvg=100.0 subj_cvg=100.0 alpha=100.0 beta=20 prob=0.9979938391094558
ATGGAAGGTAAAAATGGCCTGAAAATAGTGTGCGATAGCGGTGCGGGGTTTTCGACGGAG
CAAGCTGTTTTCCTTGCCAACAATCTTGCAAAAAAAATGCGATCGCTAAAACAAAAGTGC
AATTTGCGGCAATATAGTTATTCCATGACCGTACAATCTTGCATTCAGTGTAAATAA
>AA QryID: NC_000913_(-)_1333556_1333681 HitID: NC_020163_(-)_2102430_2102555
rank=1 bitscore=70.9 evalue=9.0E-11 perc_ident=100.0 perc_conserv=100.0 perc_gaps=0.0
query_cvg=100.0 subj_cvg=100.0 alpha=75.69 beta=11 prob=0.9979938383920322
ATGATCCAGAGGAGTTCGGCCCAGAAGAGCATCGACAGAGAAAACGCGCCCATCCAGGAC
CAATATTTCATATTTGTGTTGATCATAGGCTACACCCTTAACGGTTATTATTTTGTGTTG
AGATAA

>AA QryID: NC_000913_(-)_702883_703017 HitID: NC_004431_(-)_736394_736528
rank=1 bitscore=77.4 evalue=2.0E-12 perc_ident=97.73 perc_conserv=100.0 perc_gaps=0.0
query_cvg=100.0 subj_cvg=100.0 alpha=65.06 beta=15 prob=0.9979938115633888
GTGTCTTTTGTGAGTTTTGTCACCAAATATCGTTATTATCACTCCCTTTTACTGGCTAAA
CCAGAAAACTTATTTTATCATTCAAAAAATCAGGTCGGATTGACGCCTGTCTGCGCAAAT
CCAGGTTACGCTTAA
>AA QryID: NC_000913_(-)_1732207_1732359 HitID: NC_017638_(-)_1716365_1716517
rank=1 bitscore=62.4 evalue=6.0E-8 perc_ident=100.0 perc_conserv=100.0 perc_gaps=0.0
query_cvg=100.0 subj_cvg=100.0 alpha=81.86 beta=7 prob=0.9979938040038903
TTGTTAAAAAATACGTTTCGTTGGCGAGCGCAAAAAAATGCAGCAGTGAAAGCTAAAAAT
GGTTATAAATACGCCAGTTTGCCGGCATCAGAGAAGAAACGAATATTTGAAATGCAACAA
ATAGAGCAAAGGGATAAAATAGCAAAAGCGTGA
>AA QryID: NC_000913_(-)_1718873_1718992 HitID: NC_017651_(-)_1823824_1823943
rank=1 bitscore=61.2 evalue=7.0E-8 perc_ident=100.0 perc_conserv=100.0 perc_gaps=0.0
query_cvg=100.0 subj_cvg=100.0 alpha=100.0 beta=6 prob=0.9979937814440598
GTGCGAAAAATTTACGCGCAATTTCTGAAAAATATGTCTGACGGTAACCAAATGCAGCAA
TACATTTGTTTTAGCAATACAATTGCTGCACACTATTCTAAAAGCCGCATAATATCTTAG
>AA QryID: NC_000913_(-)_2063940_2064062 HitID: NC_017651_(-)_2261858_2261980
rank=1 bitscore=68.9 evalue=4.0E-10 perc_ident=97.5 perc_conserv=97.5 perc_gaps=0.0
query_cvg=100.0 subj_cvg=100.0 alpha=97.5 beta=10 prob=0.997993775176161З
TTGCTGGTTGTTCCGGCAGTAGCATTAATTCATTATCTGAATGAAATGTGTCAATCCACA
GAAACCGCTGTCAATGACGCATTTTGTCGGACGTTAACACGAGAAAGTCTGGCTATTCCG
TAA
>AA QryID: NC_000913_(+)_3303855_3303992 HitID: NC_011740_(+)_3225573_3225725
rank=1 bitscore=68.9 evalue=6.0E-10 perc_ident=91.11 perc_conserv=95.56 perc_gaps=0.0
query_cvg=100.0 subj_cvg=90.0 alpha=97.78 beta=9 prob=0.9979937651979054
GTGAAAAATAGTGCGACTGCGTTGTTATGCATTGCACTGTACCAGTACACGAGTACAAAA
GACAGAAAAAAAGCCCCGATGGTAAAAATCGGGGCTGTATATATTATTTTACAGATTGTG
TTCGCTGTTCAGCGATGA
>AA QryID: NC_000913_(-)_1891769_1891906 HitID: NC_017638_(-)_1877126_1877263
rank=1 bitscore=53.5 evalue=3.0E-5 perc_ident=80.0 perc_conserv=80.0 perc_gaps=0.0
query_cvg=100.0 subj_cvg=100.0 alpha=80.0 beta=5 prob=0.9979937184952907
ATGTTTATGTTTATTTATATAGTTAGTTTTATAGATAAAATTGAAATTATAAATATAAAT
AATGAATTAGCAGAAGCATCAACAGTAATATCTTATTCTCTGGTCGATTTATGTAAAACC
GACCAGTATTATCGATAA
>AA QryID: NC_000913_(-)_1102886_1103080 HitID: NC_009800_(-)_1166559_1166753
rank=1 bitscore=92.8 evalue=5.0E-17 perc_ident=100.0 perc_conserv=100.0 perc_gaps=0.0
query_cvg=100.0 subj_cvg=100.0 alpha=100.0 beta=20 prob=0.9979937092091071
ATGGAAAGTATGTCTGCGGAAATATTTTTAATAACTCACCCGCGCGTTGTATTTTTCTTT
TTTAGTTCATACCAAAGTATTAAAACCCACGTAAATACGCTGTTATCTACGCAAAAAAAT
ATTTTGTTTTCTTTTAAATCTCCGTTTTCCGCTATCAAAAAGCACCAGACAGTCATTCTT
CTTGCCCGTCGCTGA
>AA QryID: NC_000913_(-)_707256_707366 HitID: NC_009800_(-)_738387_738497
rank=1 bitscore=52.4 evalue=3.0E-5 perc_ident=100.0 perc_conserv=100.0 perc_gaps=0.0
query_cvg=100.0 subj_cvg=100.0 alpha=100.0 beta=2 prob=0.9979937074885568
TTGTTACAAGGCTTAAGTTTGCGGTTGAAAGGCTTGCAGAAGAATAAAAGCGTGAAATTC
CAGCAAGATGAAAGCGGTTACATTTTATTTAATTATGTTTTGTTATTTTAA
>AA QryID: NC_000913_(-)_4199714_4199842 HitID: NC_009801_(-)_4538717_4538845
rank=1 bitscore=69.3 evalue=3.0E-10 perc_ident=97.62 perc_conserv=97.62 perc_gaps=0.0
query_cvg=100.0 subj_cvg=100.0 alpha=100.0 beta=11 prob=0.9979936896426722
ATGACTCGCTACCCGCAGCAGGCGAGTCATTTTTACTCGTTTATCATGCCAGATTACCCG
TCATATCAGCGTTTCATCGTTGGCACGGAAGATGCAATACCCGAAGTAAGACAACCACTG
GAGGATTAA
>AA QryID: NC_000913_(-)_2651718_2651834 HitID: NC_017641_(-)_3065664_3065780
rank=1 bitscore=67.4 evalue=1.0E-9 perc_ident=100.0 perc_conserv=100.0 perc_gaps=0.0
query_cvg=100.0 subj_cvg=100.0 alpha=72.09 beta=11 prob=0.9979936836404683
TTGACAAAAAGAGAGTTACTGGCGAGTATTGTCTCCGCTGAGACGCGCTTGCATTACTAC
GGTAATACAACCTGCTGGAAAGGCCGCGAACCGGACCAGCAACAAAAAACCGCCTGA
>AA QryID: NC_000913_(-)_1744353_1744448 HitID: NC_009801_(-)_1872147_1872242
rank=1 bitscore=52.0 evalue=5.0E-5 perc_ident=100.0 perc_conserv=100.0 perc_gaps=0.0
query_cvg=100.0 subj_cvg=100.0 alpha=100.0 beta=2 prob=0.9979936793915098
GTGACAACGGGGACGAATATTAGCGGCAGAGTGGGAAGGTGGCAAGAGGCAAAACGTAAT

TTTCTGCGCTATTTCGACCGTTTGCAGAGCTTTTAA
>AA QryID: NC_000913_(+)_400451_400564 HitID: NC_011353_(+)_464582_464704
rank=1 bitscore=54.7 evalue=7.0E-6 perc_ident=91.89 perc_conserv=94.59 perc_gaps=0.0
query_cvg=100.0 subj_cvg=92.5 alpha=100.0 beta=4 prob=0.9979936687747427
GTGGGTAATTATTTAAATAAGAGAAACGTTTCGCTGGTAATCAAACAAAAAATATTTGCG
CAAAGTATTTCCTTTGTCATAAAAATAATACTTCCAGACACTATGAAGTTGTGA
>AA QryID: NC_000913_(-)_1108209_1108352 HitID: NC_017644_(-)_1146429_1146572
rank=1 bitscore=87.4 evalue=2.0E-15 perc_ident=100.0 perc_conserv=100.0 perc_gaps=0.0
query_cvg=100.0 subj_cvg=100.0 alpha=75.69 beta=20 prob=0.9979936574290964
TTGCCAGCAAATTTATTTATCAACCGTCTTACAGACGCTTCCATTCTGGTTAGTGGTAGC
GATGATGCTATGCATAAAAACCTTGAAGCCTGCGCCTGGCGCGGTCAGGTCGGTGTCACT
ATGGCAACAAGCCTAAAGGCATAA
>AA QryID: NC_000913_(-)_3595691_3595819 HitID: NC_017644_(-)_3654618_3654731
rank=1 bitscore=55.1 evalue=5.0E-6 perc_ident=91.67 perc_conserv=94.44 perc_gaps=0.0
query_cvg=85.71 subj_cvg=97.3 alpha=83.88 beta=9 prob=0.997993652607105
GTGCCGTTATTTTATGCTGACAAAGGCACTTTTTTCTGTTTATCTATCAATAAATTCAGA
ATATTATCTGTTCTTAATCGACTGAAAAATAGGGATTTTAATCGCTATTATCACAAAATA
CTGCGCTAA
>AA QryID: NC_000913_(+)_1823784_1823885 HitID: NC_009800_(+)_1843842_1843943
rank=1 bitscore=51.6 evalue=6.0E-5 perc_ident=100.0 perc_conserv=100.0 perc_gaps=0.0
query_cvg=100.0 subj_cvg=100.0 alpha=100.0 beta=2 prob=0.9979936525363279
TTGCGCCAATTATGGAGAAAAAAAGAAAATTTGATGGAGAGTGATGAGAGAATATTACAA
CACGATGATTTTGCAGAGATTATGAAGAACTATACCGGATGA
>AA QryID: NC_000913_(-)_2244884_2244988 HitID: NC_009800_(-)_2289089_2289193
rank=1 bitscore=57.4 evalue=1.0E-6 perc_ident=100.0 perc_conserv=100.0 perc_gaps=0.0
query_cvg=100.0 subj_cvg=100.0 alpha=100.0 beta=6 prob=0.9979936354745325
TTGATAATTGTTATCGTTTGCATTATCGTTACGCCGCAATCAAAAAAGGCTGACAAATCA
GAGGCTGTTCCGGCTTTCTGGGATGATCACCTGCATAAAAAAATAA
>AA QryID: NC_000913_(+)_4205673_4205795 HitID: NC_017641_(+)_4726125_4726247
rank=1 bitscore=67.4 evalue=1.0E-9 perc_ident=95.0 perc_conserv=97.5 perc_gaps=0.0
query_cvg=100.0 subj_cvg=100.0 alpha=95.0 beta=12 prob=0.9979936306308397
TTGCGCAACGCTCGCGAATTTTTCTCTTTCAATGGTGATCACAATTTTGACTGTGGTTAC
CGTGGGCAAAATACAGAAATTACATTGATGATTGTGGATAACTCTGTGCGTAAAAAGGTA
TAA
>AA QryID: NC_000913_(-)_4336084_4336188 HitID: NC_009800_(-)_4362082_4362186
rank=1 bitscore=50.1 evalue=2.0E-4 perc_ident=100.0 perc_conserv=100.0 perc_gaps=0.0
query_cvg=100.0 subj_cvg=100.0 alpha=100.0 beta=1 prob=0.9979936296505357
TTGCTGCAAACAAAAATGTCATACTTTTTGCGCGGCCCCACCCCGCGCTTTTTTTTGCCT
GTTATTTATCCTGTAAAAAAAATATGTACATGAGAAATTACTATAA
>AA QryID: NC_000913_(+)_3595610_3595756 HitID: NC_017652_(+)_3907137_3907283
rank=1 bitscore=61.2 evalue=1.0E-7 perc_ident=95.83 perc_conserv=97.92 perc_gaps=0.0
query_cvg=100.0 subj_cvg=100.0 alpha=95.83 beta=9 prob=0.9979936282670286
GTGTTGCTTTGTTTTTATGTGTTAACAAATCAGACTGTTCTTTTTTTATACTGCACTGTT
TTTGCCTGTCTGATTAAGGGGTTAGCGCAGTATTTTGTGATAATAGCGATTAAAATCCCT
ATTTTTCAGTCGATTAAGAACAGATAA
>AA QryID: NC_000913_(-)_1671632_1671802 HitID: NC_016902_(+)_2285796_2285966
rank=1 bitscore=90.9 evalue=2.0E-16 perc_ident=100.0 perc_conserv=100.0 perc_gaps=0.0
query_cvg=100.0 subj_cvg=100.0 alpha=77.46 beta=22 prob=0.9979936260569089
GTGTTCAACTACCGAGGACAATTATCATCCGCGATGACGAGAAGCAACACTGCGGATAAT
TGTAATATTATGGACAATTTGTTCAGGACGTTATTTTCTACATTTACCCATCTTCGTACC
TCATCCACTATCTTGCTCGTAGGTGAGCAGCACTGGCGCAACGCGCTTTAG
>AA QryID: NC_000913_(-)_3530467_3530595 HitID: NC_011741_(-)_3598376_3598522
rank=1 bitscore=73.9 evalue=1.0E-11 perc_ident=97.62 perc_conserv=97.62 perc_gaps=0.0
query_cvg=100.0 subj_cvg=87.5 alpha=71.86 beta=13 prob=0.9979936179233877
ATGAAATTGATGCAAATTCAACCGGTAAAGTATCGATTCACCACGCTAACTTTTTTTGAT
TATTTTCTGAAAAAAGCAAAATACTACTGCCCGGATGCGATAATTAACCCACCTTTTGAT
TCTGGATAA
>AA QryID: NC_000913_(-)_34086_34265 HitID: NC_017641_(-)_37343_37522
rank=1 bitscore=113.0 evalue=3.0E-23 perc_ident=100.0 perc_conserv=100.0 perc_gaps=0.0
query_cvg=100.0 subj_cvg=100.0 alpha=100.0 beta=30 prob=0.9979936167659101
ATGACTCGTTTTGAAGCAATTAAACAAGGCCATATTAAAATTGTGGATATATCGATAGTA
TGCAACTTCACCGTCGATAAGTGTGAGCTAAATCCTGCTTATGTCATCAAAAATATCGAT

TCCCCAAAAGATTTACTTAATGGGCAAAAAAAAACGGTCCTCATCAGAGAACCGTATTAG
>AA QryID: NC_000913_(-)_2967352_2967498 HitID: NC_013361_(-)_3857866_3858012
rank=1 bitscore=95.5 evalue=7.0E-18 perc_ident=100.0 perc_conserv=100.0 perc_gaps=0.0
query_cvg=100.0 subj_cvg=100.0 alpha=63.24 beta=25 prob=0.9979936155273434
ATGTTATCCACAGAAAGCTGGGATAACTGTGAAAAACCTCCACTACTGTTTCCATTTACA
GCCTTGACGTGCGACGAAACTCCAGTTTTTAGTGGTTCAGTGCTTAACTTAGTGGCACAT
TCTGTGGATAAATACGGCATTGGTTGA
>AA QryID: NC_000913_(-)_1339786_1339932 HitID: NC_011415_(-)_1404828_1404974
rank=1 bitscore=80.1 evalue=3.0E-13 perc_ident=97.92 perc_conserv=97.92 perc_gaps=0.0
query_cvg=100.0 subj_cvg=100.0 alpha=98.96 beta=17 prob=0.997993613687292
TTGATGATGGGCACCGGAAGTGGCGCGAAACAGGTAAACGGCGAGCATTCTACCCGTGGA
AATAAAAAATTAACAGTTGCGATTTCTCCCCTGGCAGGCTTTTTCTGCGCCGTCGTTGCC
GGAGCACAATGGAACATTAATAATTAG
>AA QryID: NC_000913_(-)_2531521_2531649 HitID: NC_017651_(-)_2751466_2751594
rank=1 bitscore=75.5 evalue=4.0E-12 perc_ident=100.0 perc_conserv=100.0 perc_gaps=0.0
query_cvg=100.0 subj_cvg=100.0 alpha=77.53 beta=16 prob=0.9979936096238844
GTGTTGACCGCTGACGTTTCGTACGTCATTAAACATTATGCCGCTAAAGGAAGAATTGAA
CCAAATCATAAAATCGATTCAGCTTTGGCTTTTCCTGTAACGATTAATTTCGCGCATCAA
AATAATTAG
>AA QryID: NC_000913_(-)_3467960_3468118 HitID: NC_017651_(-)_3772384_3772542
rank=1 bitscore=57.8 evalue=2.0E-6 perc_ident=96.15 perc_conserv=98.08 perc_gaps=0.0
query_cvg=100.0 subj_cvg=100.0 alpha=96.15 beta=8 prob=0.9979935921560056
TTGCATTTGTTGACGGCATATCTTATTCGTACTGTCAGAACGAATAAATTATTCATATTT
AAAATATCAATTCAATTTAACATTTTAAATATGAATGTTTCTGTTTTTAAAATGCTTCAT
TTGTCAGGCTTTTTTCTTATTTTTAATGCCGTAATTTAA
>AA QryID: NC_000913_(+)_4095550_4095699 HitID: NC_011353_(+)_5000600_5000749
rank=1 bitscore=103.0 evalue=3.0E-20 perc_ident=100.0 perc_conserv=100.0 perc_gaps=0.0
query_cvg=100.0 subj_cvg=100.0 alpha=77.57 beta=28 prob=0.9979935919478957
ATGGGCCATTGGCAACCAGGGAAAGATGAACGTGATGATGTTCACAATTTGCTGAATTGT
GGTGATGTGATGCTCACCGCATTTCCTGAAAATTCACGCTGTATCTTGAAAAATCGACGT
TTTTTACGTGGTTTTCCGTCGAAAATTTAA
>AA QryID: NC_000913_(-)_707189_707299 HitID: NC_016822_(-)_677671_677781
rank=1 bitscore=53.9 evalue=1.0E-5 perc_ident=100.0 perc_conserv=100.0 perc_gaps=0.0
query_cvg=100.0 subj_cvg=100.0 alpha=100.0 beta=5 prob=0.9979935830684774
ATGAAAGCGGTTACATTTTATTTAATTATGTTTTGTTATTTTAAATTTATTTTATGGATA
AATTGCGAAGCGGAATTAATTCGCGAAGAAAAGCAGAAAAAAACGCCGCTGA
>AA QryID: NC_000913_(+)_1218517_1218660 HitID: NC_009800_(+)_1267743_1267886
rank=1 bitscore=59.7 evalue=4.0E-7 perc_ident=100.0 perc_conserv=100.0 perc_gaps=0.0
query_cvg=100.0 subj_cvg=100.0 alpha=100.0 beta=9 prob=0.9979935744081404
TTGTCAACCCTGGTGTTATCACGGTTGTTTTTATATATCACCGAAATAATCCTCATCGCA
ACTATTAACAATTTTGATGTCGAAGAGTTATTTGTTAAACAAAATCGTCACCTCAAAGTG
ATCAATGTCATGAAAATAAGGTGA
>AA QryID: NC_000913_(-)_1840244_1840363 HitID: NC_011353_(-)_2348026_2348139
rank=1 bitscore=48.9 evalue=4.0E-4 perc_ident=100.0 perc_conserv=100.0 perc_gaps=0.0
query_cvg=94.87 subj_cvg=100.0 alpha=97.44 beta=2 prob=0.9979935727460982
GTGCTTATGTTGTTTATGTCATGTGCTTTTGCAGTTTTCGACTATACCATCAAGAATGTG
ACGGTAAGCAAGAAAAAAATGACCCAGGAAAGCAAATTAATAACGAGAGTAATCTCATAA
>AA QryID: NC_000913_(+)_3273138_3273257 HitID: NC_017641_(+)_3761462_3761581
rank=1 bitscore=62.8 evalue=2.0E-8 perc_ident=100.0 perc_conserv=100.0 perc_gaps=0.0
query_cvg=100.0 subj_cvg=100.0 alpha=81.43 beta=11 prob=0.9979935680662986
TTGCGCTTTACCTTACCGGGCGGCACTGCAATCCCTGAAATGATTGACATTGATCACATT
TCTGCGTTTAAACTCCTGACATTCTTATTTCACCCAATGAAGTCATTTATTTTTAAATGA
>AA QryID: NC_000913_(-)_3595735_3595872 HitID: NC_009801_(-)_3914765_3914902
rank=1 bitscore=74.7 evalue=1.0E-11 perc_ident=97.78 perc_conserv=97.78 perc_gaps=0.0
query_cvg=100.0 subj_cvg=100.0 alpha=97.78 beta=17 prob=0.9979935634118504
ATGAAAAATGTCCAGCATAATCCCCTGAATGATAGTGAATTATTCCGCCCTTTGTGCCGT
TATTTTATGCTGACAAAGGCACTTTTTTCTGTTTATCTATCAATAAATTCAGAATATTAT
CTGTTCTTAATCGACTGA
>AA QryID: NC_000913_(-)_2463055_2463225 HitID: NC_009800_(-)_2506653_2506823
rank=1 bitscore=95.1 evalue=1.0E-17 perc_ident=100.0 perc_conserv=100.0 perc_gaps=0.0
query_cvg=100.0 subj_cvg=100.0 alpha=100.0 beta=25 prob=0.9979935618547315
TTGCCTTTTTCTGTTTCTATAGAATCAAGTAGCCTACAGGGCGGCGATTACCAGGCTATG

ATCAAATCAGCAAATCAGGGCGTCTGGACATCAGTTGACGTGCTGTTACAATCGCCCACA
CCTAAACAGGCGGATACGGTATCGTTCCGTCATGGATGGCAAACTGCATAA
>AA QryID: NC_000913_(+)_2796932_2797060 HitID: NC_009800_(+)_2816353_2816481
rank=1 bitscore=55.5 evalue=5.0E-6 perc_ident=90.48 perc_conserv=92.86 perc_gaps=0.0
query_cvg=100.0 subj_cvg=100.0 alpha=90.48 beta=8 prob=0.9979935618254006
GTGATATTAAAGGTAAGGCCATATCTAATTGATTTAATTAAAAATAAATCATATCGGCAT
GTAAAAATCCACAATGTACAAAAAACGAGCCGTTACGGAATATTTTATCTACAAAAACTG
ACTAAATAA
>AA QryID: NC_000913_(+)_2920336_2920464 HitID: NC_004431_(+)_3197131_3197259
rank=1 bitscore=59.3 evalue=3.0E-7 perc_ident=100.0 perc_conserv=100.0 perc_gaps=0.0
query_cvg=100.0 subj_cvg=100.0 alpha=100.0 beta=10 prob=0.9979935539553972
TTGGCATTAGCCCTGGACGGTGGAATCCACTTCACGGAAATGAAAACAAGAACAAGAAAG
GAAGGGTTAAAACGAAGAAATAAAGAAGAGTATGAAATGGATCGCTTGACTCCAGGCAAA
CGCCAGTAA
>AA QryID: NC_000913_(-)_4173236_4173391 HitID: NC_002655_(-)_5052976_5053131
rank=1 bitscore=85.9 evalue=6.0E-15 perc_ident=100.0 perc_conserv=100.0 perc_gaps=0.0
query_cvg=100.0 subj_cvg=100.0 alpha=100.0 beta=22 prob=0.9979935529116379
ATGGAGACATGCGAGTTCATGCAACTAAAAAATTGCATAATTTGTTTTATTGGTCACATT
TTATGCGACACGATGAAGAAACAGCCGAACGGAGGGCAGATTAGCACACTTTTTCAACAT
CATTGTGCTCAACAATGCGCTCCTGCTAAACCATAA
>AA QryID: NC_000913_(-)_848304_848402 HitID: NC_009801_(-)_897114_897212
rank=1 bitscore=50.8 evalue=1.0E-4 perc_ident=100.0 perc_conserv=100.0 perc_gaps=0.0
query_cvg=100.0 subj_cvg=100.0 alpha=100.0 beta=3 prob=0.9979935519049273
ATGCAGAATTTCTCGCTACTTTTCCTCTACACCGTCTTTATATATCGAATTATGCAAAAG
CATATTTATTCCGAAAATTCCTGGCGAGCAGATAAATAA
>AA QryID: NC_000913_(+)_1500330_1500449 HitID: NC_009800_(+)_1522736_1522855
rank=1 bitscore=59.3 evalue=3.0E-7 perc_ident=100.0 perc_conserv=100.0 perc_gaps=0.0
query_cvg=100.0 subj_cvg=100.0 alpha=71.6 beta=7 prob=0.9979935501046289
ATGATTGACTTAGCCCCTTTTTCGGCATTGACTATGTCGTCTGAAAAGGGGCTGAAAAAT
TTATTTTCACCAACACTTTTTTTGCCACAACACGAAGCGGCGCTTTTTGCTATAACTTAG
>AA QryID: NC_000913_(-)_2780813_2780932 HitID: NC_010498_(-)_2856313_2856432
rank=1 bitscore=62.0 evalue=5.0E-8 perc_ident=100.0 perc_conserv=100.0 perc_gaps=0.0
query_cvg=100.0 subj_cvg=100.0 alpha=100.0 beta=12 prob=0.9979935489224816
ATGCCTCATTCTTATGCGGAATATCATCATTTCATCATGATGTCTTTGATGAGCGGTGAA
CACAATACACTTGCGCTGTCTCTTCAGGATGAATCCCCTGGTCTGGTGCCTGTGGGCTGA
>AA QryID: NC_000913_(-)_1934354_1934479 HitID: NC_011750_(+)_1267264_1267416
rank=1 bitscore=58.5 evalue=5.0E-7 perc_ident=92.68 perc_conserv=95.12 perc_gaps=0.0
query_cvg=100.0 subj_cvg=82.0 alpha=61.44 beta=4 prob=0.9979935462654259
TTGCGCGCTTTTCCCGTAATCGCACGGGTGGATAAGCGTTTACAGTTTTCGCAAGCTCGT
AAAAGCAGTACAGTGCACCGTAAGAAAATTACAAGTATACCCTGGCTTAAGTACCGGGTT
AGTTAA
>AA QryID: NC_000913_(-)_3530537_3530668 HitID: NC_004337_(-)_3501801_3501932
rank=1 bitscore=71.6 evalue=7.0E-11 perc_ident=100.0 perc_conserv=100.0 perc_gaps=0.0
query_cvg=100.0 subj_cvg=100.0 alpha=100.0 beta=18 prob=0.9979935400938758
GTGGAAAATGTTCTCAAAAGGTTAAATCTTGGTTTATATGGCGGGAATATTGTGAGTGGA
ATCGCATTCCTGAATGAAATTGATGCAAATTCAACCGGTAAAGTATCGATTCACCACGCT
AACTTTTTTTGA
>AA QryID: NC_000913_(-)_4292296_4292427 HitID: NC_011415_(-)_4566118_4566249
rank=1 bitscore=75.1 evalue=5.0E-12 perc_ident=100.0 perc_conserv=100.0 perc_gaps=0.0
query_cvg=100.0 subj_cvg=100.0 alpha=73.07 beta=21 prob=0.9979935389577259
GTGTTGCGTTGGTTTGTAGATTTTTTTAACGCTTATGATCAGTATCATTTGCGCGGTATG
TTGCGCAACCCTTTAACAACAGAGCTTTTCGCTGCATTAATGCATAATAACGCGAGTTTA
TGGCAGGATTAA
>AA QryID: NC_000913_(-)_1507145_1507267 HitID: NC_009800_(-)_1529551_1529673
rank=1 bitscore=60.8 evalue=9.0E-8 perc_ident=100.0 perc_conserv=100.0 perc_gaps=0.0
query_cvg=100.0 subj_cvg=100.0 alpha=100.0 beta=13 prob=0.9979935381026177
GTGTTTACCTCCGTGTGTAATTTGCTCGAAAATAGCACTAAATCGCCGCAACGGCAGAAT
GCAATGCAAAATCAGGAAGGAAGATTCCAGAATTTCTTTGCGCTAATTTGCAAAACTGAA
TAA
>AA QryID: NC_000913_(-)_418621_418770 HitID: NC_009801_(-)_452272_452421
rank=1 bitscore=87.8 evalue=1.0E-15 perc_ident=100.0 perc_conserv=100.0 perc_gaps=0.0
query_cvg=100.0 subj_cvg=100.0 alpha=100.0 beta=26 prob=0.99799353632191

GTGCCAATCCCTGGGGATGATACAGCGGTATATTTAAACAATTTTAGCGGGAAATACTCC
CAACCAGGCGCGGCGAAGAATAGTTTTTCGATTTACCGCGTTAATGCAGTCAGTATGACA
GCACCTGAAGCGCAATTTAAACGCTTATAA
>AA QryID: NC_000913_(-)_1096975_1097088 HitID: NC_017328_(-)_1130150_1130263
rank=1 bitscore=64.3 evalue=8.0E-9 perc_ident=100.0 perc_conserv=100.0 perc_gaps=0.0
query_cvg=100.0 subj_cvg=100.0 alpha=100.0 beta=16 prob=0.9979935352926184
GTGCGAAATTTATTCGCACTTATCGTTATGATCTACAAAGGCCACCAGCATAACAAATCC
GTGGTCGGTGGCAAAAAAAGCAGATTTCGCTTATTAAAACCACACATTGATTGA
>AA QryID: NC_000913_(+)_1078160_1078369 HitID: NC_017906_(+)_1321696_1321905
rank=1 bitscore=101.0 evalue=1.0E-19 perc_ident=100.0 perc_conserv=100.0 perc_gaps=0.0
query_cvg=100.0 subj_cvg=100.0 alpha=100.0 beta=32 prob=0.997993535130572
ATGATGTTGCAACTTTGTGCAACCATGTTAAATGTGACATGCGTAGCAAGCTTAAAAATG
AATGAAATGTTAATAAAAGAAATCGATATGACAGGGATTAAAAAAATAACTCAGACTTTT
TCTCTGCGGCAGTTAACATTTTTGAAAGGTGCAACCGCAAAAAATGTGAGAGAGTGCAAC
CTGATGAAAAATAGTGTCGCTGAGCACTAA
>AA QryID: NC_000913_(+)_688304_688420 HitID: NC_011415_(+)_772773_772895
rank=1 bitscore=55.8 evalue=3.0E-6 perc_ident=86.49 perc_conserv=89.19 perc_gaps=0.0
query_cvg=97.37 subj_cvg=92.5 alpha=82.11 beta=9 prob=0.9979935348871944
TTGCTGTTTTATTGTTTGTTTTTGCGAGATGCGCCGCACCATTCCGAAGCAAAATTCTTA
AAATGCACTCTTTTAGTGCTACCGCTGGATTACTGTGGTGCAACTAGGTTGTACTGA
>AA QryID: NC_000913_(-)_2411223_2411354 HitID: NC_017664_(-)_2561425_2561556
rank=1 bitscore=78.2 evalue=6.0E-13 perc_ident=100.0 perc_conserv=100.0 perc_gaps=0.0
query_cvg=100.0 subj_cvg=100.0 alpha=77.01 beta=25 prob=0.9979935332085959
ATGCCATTTTGCGTAATTTTCAGCCAATGGCATAATGTATATGAATTTGACGACTCAATG
AATATGTACTACTTAGGCCTTCAGGCTGCTAAAGGATACCTGATAGTGGGTATGGAAGAC
AAAATGTTTTGA
>AA QryID: NC_000913_(-)_1934504_1934653 HitID: NC_017660_(+)_2872753_2872902
rank=1 bitscore=83.6 evalue=2.0E-14 perc_ident=100.0 perc_conserv=100.0 perc_gaps=0.0
query_cvg=100.0 subj_cvg=100.0 alpha=100.0 beta=28 prob=0.9979935319162261
ATGACGATTTCCGCACTGAAAGAAATCGAAATGCAGTTTTGTCAGATATTACGCCTGTGT
GCCGTGTTAATGACAAAAGCAGATAAAAAAGTTGTTATTTTTTTTCATAACATGATCAGT
GTCAGATTTTTACCCAATGGAAAACGATGA
>AA QryID: NC_000913_(+)_1927791_1927901 HitID: NC_017664_(+)_2074479_2074589
rank=1 bitscore=59.7 evalue=3.0E-7 perc_ident=100.0 perc_conserv=100.0 perc_gaps=0.0
query_cvg=100.0 subj_cvg=100.0 alpha=100.0 beta=15 prob=0.997993531077410б
ATGTATATCGAGACAAAAGATGCGCAAATAAGCGGGGCGGTAGGCCGTTATTCAAAGAAA
TTCGCGCCAGGTAAGGTATTTACTGTATATCAGGTGATAAGGGTATTTTAG
>AA QryID: NC_000913_(+)_3965709_3965927 HitID: NC_012947_(-)_4485666_4485884
rank=1 bitscore=130.0 evalue=2.0E-28 perc_ident=100.0 perc_conserv=100.0 perc_gaps=0.0
query_cvg=100.0 subj_cvg=100.0 alpha=100.0 beta=51 prob=0.9979935310423848
ATGCCAAAAACGCCACGTGTTTACGTGGCGTTTTGCTTTTATATCTGTAATCTTAATGCC
GCGCTGGCGATGTTAGGAAAATTCCTGGAATTTGCTGGCATGTTATGCAATTTGCATATC
AAATGGTTAATTTTTGCACAGGACTGGTGGGTTTGGAACGGACTTTCCCTTCTGAATAAA
GGTCTTCGTGGTTATACTTCTGCTAATAATTTTCTCTGA
>AA QryID: NC_000913_(-)_2627142_2627294 HitID: NC_016902_(+)_1304864_1305016
rank=1 bitscore=77.0 evalue=3.0E-12 perc_ident=100.0 perc_conserv=100.0 perc_gaps=0.0
query_cvg=100.0 subj_cvg=100.0 alpha=100.0 beta=33 prob=0.9979935298752107
ATGTCCCACGCTGTGGACGGTACTTACATTAAGGCACAACAGGACAAAATGGTCAATTCT
TCGTTATGTAAAAAAGCGTCAGTGGATACATATTTTAATGTTATGGAAGTTAATTTAATT
ATTTACAATGATGATGTAATAATGATGAATTAA
>AA QryID: NC_000913_(-)_3181403_3181597 HitID: NC_011751_(-)_3644809_3645003
rank=1 bitscore=115.0 evalue=5.0E-24 perc_ident=100.0 perc_conserv=100.0 perc_gaps=0.0
query_cvg=100.0 subj_cvg=100.0 alpha=75.24 beta=70 prob=0.9979935294324305
TTGTTAAATATTAAGGGTAAAAGGATGTTTATCGCCTGGTACTGGATTGTATTGATTGCT
CTGGTTGTGGTGGGTTATTTCCTGCATTTGAAACGTTATTGTCGGGCGTTTCGCCAGGAC
AGAGACGCACTGCTTGAAGCGCGGAACAAATACTTAAACAGTACGAGAGAAGAGACAGCC
GAAAAGGTAGAATAA
>AA QryID: NC_000913_(+)_2902327_2902434 HitID: NC_017641_(+)_3330865_3330987
rank=1 bitscore=64.3 evalue=9.0E-9 perc_ident=100.0 perc_conserv=100.0 perc_gaps=0.0
query_cvg=100.0 subj_cvg=87.5 alpha=60.12 beta=13 prob=0.9979935182618911
ATGCCGCAAACACAGGTTTATCCCCGCTGGCGCGGGGAACACATTACGCCTTTTTGCGAT
TGCCCGGTTTTTGCCGGTTTATCCCCGCTGGCGCGGGGAACACTCTAA

>AA QryID: NC_000913_(+)_3408038_3408130 HitID: NC_009801_(+)_3739820_3739912
rank=1 bitscore=49.3 evalue=3.0E-4 perc_ident=100.0 perc_conserv=100.0 perc_gaps=0.0
query_cvg=100.0 subj_cvg=100.0 alpha=100.0 beta=2 prob=0.9979935171386478
TTGCCGATTATTTACGCAAATTTGCGTGCCAAAATTTTCATTCATAAAGAAAAATTGAGA
ACTTACTCAAATTTCTTTGAGTGTAAATTTTAG
>AA QryID: NC_000913_(-)_1211763_1211873 HitID: NC_017634_(-)_1232031_1232156
rank=1 bitscore=50.4 evalue=1.0E-4 perc_ident=94.44 perc_conserv=94.44 perc_gaps=0.0
query_cvg=100.0 subj_cvg=87.8 alpha=88.69 beta=1 prob=0.9979935156477107
ATGGAAATAAAAGTGCAGAGACTATCTCTATGGATGATTAATACTGTCTTTTTATTGTCA
CCCATAAATAATCACCAGACTAATACTATCAACTTGATATTTGAAATGTGA
>AA QryID: NC_000913_(-)_2282014_2282133 HitID: NC_009800_(-)_2326170_2326289
rank=1 bitscore=51.6 evalue=6.0E-5 perc_ident=100.0 perc_conserv=100.0 perc_gaps=0.0
query_cvg=100.0 subj_cvg=100.0 alpha=100.0 beta=13 prob=0.9979935134809279
TTGATGAAAGGGCAACATATTACCGTAGCAACGGTTTTTTTTCTGCTTTTTTATGCGTTA
AATCATTCTCGTTACGCTGCCTTCTGTAAAATGACCGCCATCTTTTATTGCGTCGCTTAA
>AA QryID: NC_000913_(-)_1669025_1669144 HitID: NC_017644_(-)_1652033_1652152
rank=1 bitscore=52.0 evalue=4.0E-5 perc_ident=100.0 perc_conserv=100.0 perc_gaps=0.0
query_cvg=100.0 subj_cvg=100.0 alpha=100.0 beta=14 prob=0.9979935134083698
ATGACACACAGCAAAATGAATCCGTTTATTTGGGTACTTATAATCCTGATGACGCTAGAC
GCGCTGCGGGAATTGGCTGGCGCTTCGTCTATTTTAGGATGGCTATTAACGTTGGTTTGA
>AA QryID: NC_000913_(-)_944950_945063 HitID: NC_017641_(-)_1045742_1045855
rank=1 bitscore=61.2 evalue=8.0E-8 perc_ident=100.0 perc_conserv=100.0 perc_gaps=0.0
query_cvg=100.0 subj_cvg=100.0 alpha=73.55 beta=14 prob=0.9979935132561297
ATGCGCATCATGCCACCGCCAAAAACATTGTCAATGGGTGAAAATACGCGATATCCCAGC
GGCGGTATTATCGATTTATATTACTCATTCATAACGCTAATGCTGAAGAGATGA
>AA QryID: NC_000913_(-)_85348_85470 HitID: NC_017641_(-)_87340_87462
rank=1 bitscore=58.5 evalue=5.0E-7 perc_ident=100.0 perc_conserv=100.0 perc_gaps=0.0
query_cvg=100.0 subj_cvg=100.0 alpha=72.73 beta=7 prob=0.9979935120826234
TTGATAATAAATCGGCTGAATCCCACAACTTACAGCAATTTCTCTATTTTTTTAATTAAT
CCTAAAATAGAGAGCCCACACATTCAGAATAAAATTCTACATTCAATAAAAAAATGCAGAA
TAA
>AA QryID: NC_000913_(+)_4615137_4615241 HitID: NC_017664_(+)_4874043_4874144
rank=1 bitscore=50.1 evalue=2.0E-4 perc_ident=100.0 perc_conserv=100.0 perc_gaps=0.0
query_cvg=97.06 subj_cvg=100.0 alpha=98.53 beta=5 prob=0.9979935060901379
GTGATGTTCACTTTGATCCTGATGCGTTTGCCACCACTGACGCATTCATTTGAAAGTGAA
TTATTTGAACCAGATCGCATTACAGTGATGCAAACTTGTAAGTAG
>AA QryID: NC_000913_(-)_29438_29533 HitID: NC_018650_(+)_4396799_4396894
rank=1 bitscore=49.7 evalue=2.0E-4 perc_ident=100.0 perc_conserv=100.0 perc_gaps=0.0
query_cvg=100.0 subj_cvg=100.0 alpha=100.0 beta=11 prob=0.9979935040947242
ATGGGCGCTAAAAGTCAACTTAATGGCGGGATTTTTTATATTATTTTGTGGCTCATGCAT
GTTTTACAGGCATTAAGCGACAAAAAGATCATCTAA
>AA QryID: NC_000913_(-)_4366386_4366502 HitID: NC_007946_(-)_4640475_4640591
rank=1 bitscore=50.8 evalue=1.0E-4 perc_ident=97.37 perc_conserv=100.0 perc_gaps=0.0
query_cvg=100.0 subj_cvg=100.0 alpha=97.37 beta=9 prob=0.9979934968867805
GTGATCCAGATTACGGTAGAAATCCTCAAGCAGCATATGATCTCGGGTATTCGGTCGATG
CAGGGGATAATCGTCGGTCGAAAAACATTCGAAACCACATATATTCTGTGTGTTTAA
>AA QryID: NC_000913_(-)_4099494_4099610 HitID: NC_007946_(-)_4378421_4378549
rank=1 bitscore=52.0 evalue=5.0E-5 perc_ident=100.0 perc_conserv=100.0 perc_gaps=0.0
query_cvg=100.0 subj_cvg=90.48 alpha=100.0 beta=16 prob=0.9979934964586658
GTGATCAAAACCACATCAATTGAAACGCTGTTTTATTTTTATCGGATCATTGTTTTTATT
TTGAAATTGATCACAAAAAAAACCACCGCCGTTGGCGATGGTTCATTTTTTAAGCTGA
>AA QryID: NC_000913_(+)_4116350_4116451 HitID: NC_009801_(+)_4446001_4446102
rank=1 bitscore=49.7 evalue=2.0E-4 perc_ident=100.0 perc_conserv=100.0 perc_gaps=0.0
query_cvg=100.0 subj_cvg=100.0 alpha=100.0 beta=5 prob=0.9979934883706802
GTGGATTATGTCACCAGGAGAAGGATGAAAAGTGTGGCAAACCGTAATCTGCAAGGCGTG
CCGCTGGACAGCGATGGCGCGGCTTCATACAATCGGAGCTAA
>AA QryID: NC_000913_(-)_3217289_3217402 HitID: NC_009801_(-)_3548922_3549035
rank=1 bitscore=47.8 evalue=0.001 perc_ident=100.0 perc_conserv=100.0 perc_gaps=0.0
query_cvg=100.0 subj_cvg=100.0 alpha=100.0 beta=6 prob=0.9979934616080649
GTGCAATATTTAATGCTATCTGTTAACATTTGTTGTTATTTTGCACGGTTTTTGTCGGGT
ATGGATTTGATGATTGCTGCGCAAATGCACGTTTTCAGGGCAATAATTGCCTGA
>AA QryID: NC_000913_(-)_1050408_1050506 HitID: NC_013008_(-)_1234858_1234956

rank=1 bitscore=47.8 evalue=9.0E-4 perc_ident=100.0 perc_conserv=100.0 perc_gaps=0.0
query_cvg=100.0 subj_cvg=100.0 alpha=100.0 beta=1 prob=0.9979934586008614
TTGAGATTAAGCAAATTTTATTTTAGCCGTCCGGCGGTCTTAAATATCGCTGGCTTTATT
TTATTAACCCCGTATAGTGCAGCGCAGTATTTTCAGTAA
>AA QryID: NC_000913_(-)_4038703_4038813 HitID: NC_016856_(-)_4413499_4413630
rank=1 bitscore=51.6 evalue=6.0E-5 perc_ident=83.33 perc_conserv=91.67 perc_gaps=0.0
query_cvg=100.0 subj_cvg=83.72 alpha=88.89 beta=8 prob=0.997993453470564
ATGGCGGGCGTCCTGCCCGCCACCCTCCGGGCCGTTGCTTCGCAACGTTCAAATCCGCTC
CCGGCGGATTTGTCCTACTCAGGAGAGCGTTCACCGACAAACAACAGATAA
>AA QryID: NC_000913_(-)_4169823_4169933 HitID: NC_016856_(-)_4413499_4413630
rank=1 bitscore=51.6 evalue=6.0E-5 perc_ident=83.33 perc_conserv=91.67 perc_gaps=0.0
query_cvg=100.0 subj_cvg=83.72 alpha=88.89 beta=8 prob=0.997993453470564
ATGGCGGGCGTCCTGCCCGCCACCCTCCGGGCCGTTGCTTCGCAACGTTCAAATCCGCTC
CCGGCGGATTTGTCCTACTCAGGAGAGCGTTCACCGACAAACAACAGATAA
>AA QryID: NC_000913_(-)_1570188_1570301 HitID: NC_017641_(-)_1815439_1815552
rank=1 bitscore=48.9 evalue=4.0E-4 perc_ident=97.3 perc_conserv=97.3 perc_gaps=0.0
query_cvg=100.0 subj_cvg=100.0 alpha=97.3 beta=4 prob=0.9979934461564657
GTGATAATTCAGGAGACACAGAATGCGCATAAAAATAACAGCATAAAACACCTTACCACC
ACCCAAGAATTTCATATTGTATTGTTTTTCAATGAAAAAATATTATTCGCGTAA
>AA QryID: NC_000913_(-)_395557_395658 HitID: NC_009801_(-)_429313_429414
rank=1 bitscore=47.4 evalue=0.001 perc_ident=100.0 perc_conserv=100.0 perc_gaps=0.0
query_cvg=100.0 subj_cvg=100.0 alpha=100.0 beta=5 prob=0.9979934442083906
ATGCAGGCATTGGTGAGCAGGAAAAAGATCGTGTTTACGCGCCGTTACGTTTTTAACACT
ATTATTGCACTAATTCGCCCTTTGCAATCTATCAATGAGTAG
>AA QryID: NC_000913_(+)_42224_42334 HitID: NC_009800_(+)_45484_45594
rank=1 bitscore=52.0 evalue=4.0E-5 perc_ident=94.44 perc_conserv=94.44 perc_gaps=0.0
query_cvg=100.0 subj_cvg=100.0 alpha=94.44 beta=17 prob=0.9979934414016234
TTGCTACGCCGTGTTTTCAATATTGGTGAGGAACTTAACAATATTGAAAGTTGGATTTAT
CTGCGTGTGACATTTTCAATATTGGTGATTAAAGTTTTATTTCAAAATTAA
>AA QryID: NC_000913_(-)_655236_655346 HitID: NC_009801_(-)_678686_678796
rank=1 bitscore=47.4 evalue=0.001 perc_ident=100.0 perc_conserv=100.0 perc_gaps=0.0
query_cvg=100.0 subj_cvg=100.0 alpha=100.0 beta=1 prob=0.9979934315356347
TTGTTATCTATGTATAAAAACAGCAACTTCAATGTCTTAATGGCAGTTTTTCTTGATTTT
AATCAGCATTCATCGCCAATTTATTGGGCATATTTTTTCCTTAAGCTTTAG
>AA QryID: NC_000913_(-)_1830266_1830364 HitID: NC_017656_(-)_2183690_2183788
rank=1 bitscore=55.8 evalue=3.0E-6 perc_ident=100.0 perc_conserv=100.0 perc_gaps=0.0
query_cvg=100.0 subj_cvg=100.0 alpha=72.99 beta=4 prob=0.9979934193445767
ATGGTTGTTAATGATGTCAACGATGGCGCAAAAAATGCCCGCTTTTGGTGCGCGCTGCGT
CAGAATGGCGCAGTAATTTCCAGTAAATTTCGTCAATGA
>AA QryID: NC_000913_(-)_1797080_1797175 HitID: NC_012947_(+)_2038913_2039008
rank=1 bitscore=42.4 evalue=0.04 perc_ident=100.0 perc_conserv=100.0 perc_gaps=0.0
query_cvg=100.0 subj_cvg=100.0 alpha=100.0 beta=9 prob=0.9979933885421901
ATGCGCGTTTTGAAAAATAAAAAGCCTGTCTCATCAGGTGTAATTGTCGCAGCCAGACTG
GACACGGCCAGCGCGGAGAAACCGGAGCGTATTTAA
>AA QryID: NC_000913_(+)_3250784_3250876 HitID: NC_009801_(+)_3587561_3587653
rank=1 bitscore=46.6 evalue=0.002 perc_ident=100.0 perc_conserv=100.0 perc_gaps=0.0
query_cvg=100.0 subj_cvg=100.0 alpha=100.0 beta=2 prob=0.9979933835327789
ATGTCGTTATATAAATTACCTATAAAAAATAACCATGTATTTATCTTAAATATCAAAACC
TTTATAACATCCGAAAAAGAAAGATACGAATAG
>AA QryID: NC_000913_(+)_1064566_1064667 HitID: NC_009800_(+)_1126179_1126280
rank=1 bitscore=46.6 evalue=0.002 perc_ident=100.0 perc_conserv=100.0 perc_gaps=0.0
query_cvg=100.0 subj_cvg=100.0 alpha=100.0 beta=1 prob=0.997993378945282
TTGGTAAATATTGTCTCTGTATTGGTAACGCCGCAGATATTCTGTTTAGCCACAGGTGCA
ATTATCAGCGGCGTACGCGAGGCAGGGGCTAATCAGGCATAG
>AA QryID: NC_000913_(-)_4276159_4276272 HitID: NC_009801_(-)_4617220_4617333
rank=1 bitscore=45.8 evalue=0.004 perc_ident=100.0 perc_conserv=100.0 perc_gaps=0.0
query_cvg=100.0 subj_cvg=100.0 alpha=100.0 beta=3 prob=0.997993368804496
TTGACGATGAAAAACCAGGCAATTGATGCGCAAACGTTATCGTCGGTGCGCAAGATTGCA
GCGTTATCGAGCCGTATACCGTTCAGGATTGCGATAAAAAATCGTCTGAGATGA
>AA QryID: NC_000913_(-)_3662714_3662821 HitID: NC_009800_(-)_3706002_3706109
rank=1 bitscore=44.7 evalue=0.007 perc_ident=100.0 perc_conserv=100.0 perc_gaps=0.0
query_cvg=100.0 subj_cvg=100.0 alpha=100.0 beta=3 prob=0.9979933305185549

GTGCGAAAATATATTCGTGCTGCATTTACTTATTATCAATTAACTGTTATGCAAAACTAC
TTTGTGGATAAATTTTGGTCCTACCAAATCTGGCAGTTTTTGCGCTAA
>AA QryID: NC_000913_(-)_418419_418529 HitID: NC_009800_(-)_482987_483097
rank=1 bitscore=42.7 evalue=0.033 perc_ident=97.22 perc_conserv=97.22 perc_gaps=0.0
query_cvg=100.0 subj_cvg=100.0 alpha=97.22 beta=6 prob=0.9979933091165099
ATGGCTAATCATGCGAACAAAAAATCACCAATCGTTGTAAAATCTAACGCCCGTATTTTG
ACTGCAAAGAAAATAAGCCAGCATCGACTGGCTTATGGCAATAAAAGATGA
>AA QryID: NC_000913_(-)_2815555_2815680 HitID: NC_017631_(-)_3022336_3022461
rank=1 bitscore=46.2 evalue=0.002 perc_ident=95.12 perc_conserv=97.56 perc_gaps=0.0
query_cvg=100.0 subj_cvg=100.0 alpha=95.12 beta=4 prob=0.9979932966213234
ATGCACCATCTTCTCCAAAAAAATTTCCTTCAAAAATATCTCATTTCTAAATAACTCCTTT
ATCCATTTCCTGAATTTTCTTCTCCTTCGCCATCAGCGACAAAATCAAGCAATTACGCTA
AAGTAG
>AA QryID: NC_000913_(-)_3352118_3352222 HitID: NC_020163_(-)_4122839_4122943
rank=1 bitscore=45.8 evalue=0.004 perc_ident=97.06 perc_conserv=97.06 perc_gaps=0.0
query_cvg=100.0 subj_cvg=100.0 alpha=97.06 beta=2 prob=0.9979932647118321
GTGATTTATGTGATTATGCATGAAATTTCGGTAATTTTGATTGATTATGCAGTTGATAAA
AGCAGGCCGGGACGATTGATCCAGGTCATCGCCGAATTGAGCTAA
>AA QryID: NC_000913_(+)_4593887_4593979 HitID: NC_009801_(+)_4935205_4935297
rank=1 bitscore=45.8 evalue=0.003 perc_ident=96.67 perc_conserv=100.0 perc_gaps=0.0
query_cvg=100.0 subj_cvg=100.0 alpha=96.67 beta=2 prob=0.9979932515324008
GTGAGTCCATTAAAACCCGATTTAAAACATTTTTATGCGTGTGTTTTCACAAAAAAGATT
TTCCGTTCCGCTGCCATTTTTCATTTTCATTGA
>AA QryID: NC_000913_(-)_3913278_3913370 HitID: NC_009800_(-)_3942434_3942520
rank=1 bitscore=44.3 evalue=0.01 perc_ident=100.0 perc_conserv=100.0 perc_gaps=0.0
query_cvg=93.33 subj_cvg=100.0 alpha=96.67 beta=1 prob=0.997993231744092
GTGACGATGGTCGAAAATGGCGCTTTCGTCAGCGGGGATAATCCGTTATTGAACAATTTA
TCCTCTGTCCATTTCACGATGAAAAAAATGTAG
>AA QryID: NC_000913_(-)_3634010_3634108 HitID: NC_009801_(-)_3947676_3947789
rank=1 bitscore=46.6 evalue=0.002 perc_ident=81.25 perc_conserv=90.62 perc_gaps=0.0
query_cvg=100.0 subj_cvg=86.49 alpha=87.05 beta=8 prob=0.997993208270975
GTGGATGGAAAGGTGATTGAAAACTCACTCAGTGGCCAGATCTTTATGAAATATGAACAG
GTTGAAAAAAACAGTAACTTTGCTGTTTTTTATACTTAA
>AA QryID: NC_000913_(-)_3465063_3465179 HitID: NC_017628_(-)_3837937_3838053
rank=1 bitscore=49.7 evalue=2.0E-4 perc_ident=97.37 perc_conserv=97.37 perc_gaps=0.0
query_cvg=100.0 subj_cvg=100.0 alpha=77.54 beta=7 prob=0.9979931531474431
GTGTTGGCGATACCTTCCAGTATCGCCGCATTTTTTGTCAATGGGCTACTTTTGGTAGCC
CATTATTTTTATTCATATTTCTATTCTTTGCTTAAAAAAGAGTCTTTCCGTAATTGA
>AA QryID: NC_000913_(+)_3421291_3421401 HitID: NC_016856_(-)_4413499_4413630
rank=1 bitscore=55.8 evalue=3.0E-6 perc_ident=86.11 perc_conserv=94.44 perc_gaps=0.0
query_cvg=100.0 subj_cvg=83.72 alpha=53.54 beta=8 prob=0.9979931430298756
ATGGCGGGCGTCCTGCCCGCCACCCTCCGGGCCGTTGCTTCGCAACGTTCAAATCCGCTC
CCGGCGGATTTGTCCTACTCGGGAGAGTGTTCACCGACAAACAACAGATAA
>AA QryID: NC_000913_(-)_1391114_1391224 HitID: NC_009800_(-)_1440541_1440651
rank=1 bitscore=42.7 evalue=0.027 perc_ident=100.0 perc_conserv=100.0 perc_gaps=0.0
query_cvg=100.0 subj_cvg=100.0 alpha=100.0 beta=1 prob=0.9979931323842831
TTGATGCGTAATTATTTGTTTCGCCTTAAAGAAATGCAAACAACTTCCATCAAAAAAGTG
ACAAAGGAAGCATCAAATGTTTTAAGTATCAATATATCAAATCAGATTTAG
>AA QryID: NC_000913_(-)_1328800_1328916 HitID: NC_013716_(+)_1841973_1842110
rank=1 bitscore=49.7 evalue=2.0E-4 perc_ident=81.08 perc_conserv=86.49 perc_gaps=0.0
query_cvg=97.37 subj_cvg=82.22 alpha=72.81 beta=1 prob=0.9979930881655081
TTGGCCTACAGGCGCTATAGCACGACGGAATTTTGGTCGAATGTCAAGCAAATCTATTGC
CAGATTGACGAAAGCGTCACACTGTACCTGATAAAATCCGATACGCAACTTTATTAG
>AA QryID: NC_000913_(+)_3416869_3416988 HitID: NC_017652_(+)_3721431_3721550
rank=1 bitscore=53.9 evalue=1.0E-5 perc_ident=100.0 perc_conserv=100.0 perc_gaps=0.0
query_cvg=100.0 subj_cvg=100.0 alpha=71.03 beta=2 prob=0.9979930684733717
ATGTGGCGCATGTTTCATTTTCGCACCATTGCGGGGCGCTGTTTTTATTATTTCCTGTCT
ACACTCAGCTTATTGCGACGCGAAATCGTGCGCAAAAGCTGGCACTACTTTTGCTTATAA
>AA QryID: NC_000913_(-)_4244535_4244633 HitID: NC_017625_(+)_4267863_4267958
rank=1 bitscore=43.9 evalue=0.012 perc_ident=100.0 perc_conserv=100.0 perc_gaps=0.0
query_cvg=96.88 subj_cvg=100.0 alpha=98.44 beta=14 prob=0.9979930665665008
GTGATGTTGCTTGCAAAAATCGTGGCGATTTTATGTGCGCATCTCCACATTACCGCCAAT

TCTGTAACAGAGATCACACAAAGCGACGGTGGGGCGTAG
>AA QryID: NC_000913_(+)_1255517_1255699 HitID: NC_012947_(-)_2537761_2537940
rank=1 bitscore=64.7 evalue=1.0E-8 perc_ident=67.8 perc_conserv=67.8 perc_gaps=0.0
query_cvg=98.33 subj_cvg=100.0 alpha=67.23 beta=24 prob=0.9979930452773607
TTGATGTATCTGCATAACAAAAACAATATGCGCCATTTTTGCATTACTAATAAGAAAAAG
CAAAAAACATCGATATTCATTTTTGGTCAATGGGTTTTCTGGCCTATCGTTGTTATTGTG
CTACTGGTTATCATTTTCATCTTGCCAGCATATTGGAGCGTGATCAATTTTGATCAGCTG
TGA
>AA QryID: NC_000913_(-)_2360248_2360343 HitID: NC_009800_(-)_2403013_2403108
rank=1 bitscore=41.2 evalue=0.088 perc_ident=100.0 perc_conserv=100.0 perc_gaps=0.0
query_cvg=100.0 subj_cvg=100.0 alpha=100.0 beta=1 prob=0.997993032415294
GTGCGTAATTTCTCCAGTCTGCTGGACGAGGAGCGGGATTTGATGCGCTTCACAATTTTA
CTCCCCCTGAGCAACGAGACTGATTCCAGTATCTAA
>AA QryID: NC_000913_(-)_4470660_4470764 HitID: NC_009801_(-)_4813195_4813299
rank=1 bitscore=45.1 evalue=0.006 perc_ident=94.12 perc_conserv=94.12 perc_gaps=0.0
query_cvg=100.0 subj_cvg=100.0 alpha=94.12 beta=1 prob=0.9979930202196187
TTGCGTGAAAGTGAAAAAGGAAAAAGCAGGGAATGTCTGCAATTATTGATACCGAAGGAC
AGTTCCCCTGCAGAATCACATCAAATAAAAATGCATATACCTTGA
>AA QryID: NC_000913_(-)_4601105_4601197 HitID: NC_009800_(-)_4606397_4606489
rank=1 bitscore=45.4 evalue=0.004 perc_ident=93.33 perc_conserv=100.0 perc_gaps=0.0
query_cvg=100.0 subj_cvg=100.0 alpha=93.33 beta=1 prob=0.9979930083391093
ATGCAAAATCACACAACCAATATTTATTCAATGAAATCTGATGGTTTTAGCTTGTTAACA
TTAAAAAACGTGCCCGGTTATCTATTATCCTGA
>AA QryID: NC_000913_(-)_310759_310854 HitID: NC_009801_(-)_335050_335145
rank=1 bitscore=38.5 evalue=0.55 perc_ident=100.0 perc_conserv=100.0 perc_gaps=0.0
query_cvg=100.0 subj_cvg=100.0 alpha=100.0 beta=2 prob=0.9979929580742656
ATGCAAGTAAGAATTGAACGTTATATTGCCAATAACCTTATGAAACCAAATGTCTTTTTC
TTCTTATCAAAAAAGCAATATTTTCAGTTTTTCTAA
>AA QryID: NC_000913_(-)_2589147_2589248 HitID: NC_009800_(-)_2613032_2613133
rank=1 bitscore=40.0 evalue=0.18 perc_ident=100.0 perc_conserv=100.0 perc_gaps=0.0
query_cvg=100.0 subj_cvg=100.0 alpha=100.0 beta=1 prob=0.9979929464809324
ATGAGAATAAATACTCATTTAAGGGTGAAAACATTTTGCCTGCAAATGATTATCAGGAAA
TATAACATTATCGAAGTATTCAAATTAACAACTATTAAATAA
>AA QryID: NC_000913_(+)_1084010_1084111 HitID: NC_017646_(+)_1159064_1159180
rank=1 bitscore=37.7 evalue=0.84 perc_ident=96.97 perc_conserv=100.0 perc_gaps=0.0
query_cvg=100.0 subj_cvg=86.84 alpha=90.59 beta=1 prob=0.9979929298032992
ATGCAAATCCTGCTGAAAGCACACAGCTTTTTTCATCACTGTCATCACTCTGTCATCTTT
CCAGTAGAAACTAATGTCACTGAAATGGTGTTTTATAGTTAA
>AA QryID: NC_000913_(+)_2288318_2288413 HitID: NC_009801_(+)_2477387_2477482
rank=1 bitscore=36.6 evalue=2.0 perc_ident=100.0 perc_conserv=100.0 perc_gaps=0.0
query_cvg=100.0 subj_cvg=100.0 alpha=100.0 beta=2 prob=0.9979928108228346
GTGATTGATTTTTTAGTTGTTTTTCTTGATGAGAAGCTGATGCAAAATTCCGTCTTTATA
ATGAAAATGATGCCAAAGCGAACGACAAGGTTGTAG
>AA QryID: NC_000913_(-)_3865644_3865736 HitID: NC_009801_(-)_4184383_4184475
rank=1 bitscore=38.1 evalue=0.63 perc_ident=100.0 perc_conserv=100.0 perc_gaps=0.0
query_cvg=100.0 subj_cvg=100.0 alpha=100.0 beta=1 prob=0.9979927939806704
TTGATCGTGGGTGTTGGCCTGATGAGTTATAGCGATCCCTTGCTGAAAATAACATCATCA
TTACGTCGCACTGTGGCGGCTATCGCACTTTAA
>AA QryID: NC_000913_(+)_474446_474586 HitID: NC_011353_(+)_544889_545011
rank=1 bitscore=49.7 evalue=4.0E-4 perc_ident=67.5 perc_conserv=70.0 perc_gaps=0.0
query_cvg=86.96 subj_cvg=100.0 alpha=63.1 beta=6 prob=0.9979927297222496
TTGCTACTGGTCCGATGGGTGCAATGGTCTGAATTACGGGCTAATTACAGGCAGAAATGC
GTGATGTGTGCCACACTTGTTGACGTTACTATTTTGTTAACCACTCTTCCGGCGAGGAAA
GTTAGCCCGCTGGTGCATTGA
>AA QryID: NC_000913_(-)_29237_29404 HitID: NC_017638_(-)_29238_29405
rank=1 bitscore=57.8 evalue=2.0E-6 perc_ident=67.27 perc_conserv=67.27 perc_gaps=0.0
query_cvg=100.0 subj_cvg=100.0 alpha=67.27 beta=13 prob=0.9979927168735294
TTGCATGAAATAAAAACGAGCAGAAAAAAGTGGACCAAATGGTCAAAATTTACATTAAAA
CAACAAAAACAGAAGACAAAACATGTAATTTTTGGCACTTATAGACAATTTAAAAACATA
ACACCAAAAATAAAAGGGCCAAAAATTAAAGCCCTTTATAATCAATAA
>AA QryID: NC_000913_(-)_3427065_3427229 HitID: NC_017664_(-)_3638505_3638669

rank=1 bitscore=52.0 evalue=8.0E-5 perc_ident=70.37 perc_conserv=70.37 perc_gaps=0.0 query_cvg=100.0 subj_cvg=100.0 alpha=72.22 beta=6 prob=0.9979926876642108
ATGACTTTGGGGGCATTATTGGCCTTGTGCAAGTCTTTTAGTATGCAAAAAAGCACCGTT
TTGTGTGCGATTGCAGCAAAAAGGGTGAAAAAACAACAAACAGAAAAAAAGATCAAAAAA
ATACTTGTGCAAAAAAATTGGGATCCCTATAATGCGCCTCCGTTGA
>AA QryID: NC_000913_(+)_312165_312257 HitID: NC_009801_(+)_336456_336548
rank=1 bitscore=39.7 evalue=0.24 perc_ident=96.67 perc_conserv=100.0 perc_gaps=0.0 query_cvg=100.0 subj_cvg=100.0 alpha=96.67 beta=1 prob=0.9979926303485227
GTGATTGATGATGGTTTTGCTGAGAGTATGGATTTTTCGAGAATGGAGGTTATCTCCACT
TTACTGGCCGATAATTTATCGCAGTATGATTGA
>AA QryID: NC_000913_(-)_985006_985104 HitID: NC_009801_(-)_1051696_1051794
rank=1 bitscore=51.6 evalue=6.0E-5 perc_ident=100.0 perc_conserv=100.0 perc_gaps=0.0 query_cvg=100.0 subj_cvg=100.0 alpha=71.57 beta=2 prob=0.9979924976231794
TTGTTAAATGCCGAAAAAACAGGACTTTGGTCCTGTTTTTTTATACCTTCCAGAGCAAT
CTCACGTCTTGCAAAAACAGCCTGCGTTTTCATCAGTAA
>AA QryID: NC_000913_(+)_440655_440747 HitID: NC_009801_(+)_474306_474398
rank=1 bitscore=36.6 evalue=1.8 perc_ident=96.67 perc_conserv=96.67 perc_gaps=0.0 query_cvg=100.0 subj_cvg=100.0 alpha=96.67 beta=2 prob=0.9979924619137766
GTGCACAGCAAAGCGATGTTAGTGGTATACTTCCGCGCCTGGATGCAGCCGCAGGTGTGG
GCTGCTGTATTTTTCCCTATACAAGTCGCTTAA
>AA QryID: NC_000913_(-)_3670097_3670210 HitID: NC_017651_(-)_3981871_3981999
rank=1 bitscore=45.4 evalue=0.005 perc_ident=81.08 perc_conserv=91.89 perc_gaps=0.0 query_cvg=100.0 subj_cvg=88.1 alpha=76.25 beta=2 prob=0.9979922616245072
GTGCTTATTTTTGTCTCCTTGCCGTTTGTTATTTTGGCTTGTGGTCGCGGAATTTCATTT
TTAATTGCATGCGTACTAACCACCAATATAAGGAAATCCTCAAAATTGTTTTAA
>AA QryID: NC_000913_(-)_3550755_3550856 HitID: NC_013008_(-)_4323962_4324063
rank=1 bitscore=35.8 evalue=3.7 perc_ident=93.94 perc_conserv=96.97 perc_gaps=0.0 query_cvg=100.0 subj_cvg=100.0 alpha=93.94 beta=2 prob=0.9979919008825642
ATGAAAGTGCCATCTAAAAAGATGATCTTAATAAATCTATTAAGAATGAGATGGAGCACA
CTGGATATTTTACTTATGAAACTGTTTCACTCCTTTACTTAA
>AA QryID: NC_000913_(-)_2362418_2362519 HitID: NC_009800_(-)_2405183_2405284
rank=1 bitscore=35.0 evalue=6.1 perc_ident=93.94 perc_conserv=93.94 perc_gaps=0.0 query_cvg=100.0 subj_cvg=100.0 alpha=93.94 beta=1 prob=0.9979913764032202
GTGAAAGTGACAGTAATCTCTTTTGCGATGATAACTGTACTTATTATTATTTTTGTGTGT
ATCGATAAAGTGTGTTTATTTTTTCGGCATTTAATTATTTAA
>AA QryID: NC_000913_(+)_2261683_2261814 HitID: NC_009801_(+)_2451950_2452081
rank=1 bitscore=50.8 evalue=1.0E-4 perc_ident=72.09 perc_conserv=72.09 perc_gaps=0.0 query_cvg=100.0 subj_cvg=100.0 alpha=72.09 beta=16 prob=0.9979913737813663
GTGCTGCACAATTTTTTGCCATTTTTCCTAATTGCTGATGGGAAAATCCGTTATCAGCAA
TTTCATTTCAGCAGCGTCAGCAGCCAATCAGCAACAGTGAAATGGCATTTTGACTATTCG
TGGCGCTTTTGA
>AA QryID: NC_000913_(+)_1804248_1804355 HitID: NC_013008_(+)_2311213_2311320
rank=1 bitscore=52.8 evalue=3.0E-5 perc_ident=100.0 perc_conserv=100.0 perc_gaps=0.0 query_cvg=100.0 subj_cvg=100.0 alpha=61.73 beta=2 prob=0.9979913356213466
TTGAATAAGCATGTGGCGAAAACAGATTTTTATTTATATATATTTATCTGCAAAATTTTA
AATAAAGCTCCAATAAATCATATTGTTAATTTCTTCACTTTCCGCTGA
>AA QryID: NC_000913_(+)_384012_384119 HitID: NC_009801_(+)_417643_417750
rank=1 bitscore=42.7 evalue=0.028 perc_ident=100.0 perc_conserv=100.0 perc_gaps=0.0 query_cvg=100.0 subj_cvg=100.0 alpha=74.64 beta=3 prob=0.9979911940661221
ATGTTAAGTGTTTGTATGTTTGGTATGTATAGTTATTTTGTTTTATACATTGTATTTAGA
TGTGATTTAGTTTGTCAATTAATTGCATTTAAAAAATATGTTCTGTGA
>AA QryID: NC_000913_(-)_1211527_1211640 HitID: NC_017641_(-)_1419454_1419567
rank=1 bitscore=48.1 evalue=6.0E-4 perc_ident=97.3 perc_conserv=97.3 perc_gaps=0.0 query_cvg=100.0 subj_cvg=100.0 alpha=71.85 beta=1 prob=0.9979910969418503
ATGTATTTTAGCCAGGAACAGGTTAAATCATTCCTATATAACTCAAAAATTGAAACCTTA
TTCTCATGTCATGCTTATATTCATTATTATCGTTATATAAAAAGGCAACCATAA
>AA QryID: NC_000913_(+)_85445_85570 HitID: NC_010658_(+)_76414_76539
rank=1 bitscore=47.4 evalue=0.001 perc_ident=92.68 perc_conserv=92.68 perc_gaps=0.0 query_cvg=100.0 subj_cvg=100.0 alpha=68.69 beta=3 prob=0.9979907214614223
GTGGGATTCAGCCGATTTATTATCAATTTAATCCTCTGTAATGGAGGATTTTATCGTTTC
TTTTCACCTTTCCTCCTGTTTATTCTTATTACCCCGTGTTTATGTCTCTGGCTGCCAATT
GCTTAA

>AA QryID: NC_000913_(-)_3524281_3524406 HitID: NC_016856_(-)_3663870_3663983
rank=1 bitscore=45.8 evalue=0.003 perc_ident=69.44 perc_conserv=83.33 perc_gaps=0.0
query_cvg=87.8 subj_cvg=97.3 alpha=64.27 beta=2 prob=0.9979901726909457
GTGGAGTTTAACTTGAGCATCATTAAGCACATCACAGTAACCGCTGATCATAGCAAACCC
TCTGAAGGCGGAATATCAGCAAATTCCTGGAGTTATTTCAACCTATTGACTTTTAATTTG
ATTTGA
>AA QryID: NC_000913_(+)_4175152_4175268 HitID: NC_016514_(+)_207937_208032
rank=1 bitscore=47.0 evalue=0.001 perc_ident=87.1 perc_conserv=90.32 perc_gaps=0.0
query_cvg=81.58 subj_cvg=100.0 alpha=53.6 beta=2 prob=0.9979869883563235
TTGCCGATAACATTTGACGCAATGCGCACTAAAAGGGCATCATTTGATGCCCTTTTTGCA
CGCTTTCGTACCAGAACCTGGCTCATCAGTGATTTTCTTTGTCATAATCATTGCTGA
>AA QryID: NC_000913_(+)_2541687_2541779 HitID: NC_010102_(-)_3570393_3570497
rank=1 bitscore=47.0 evalue=0.001 perc_ident=83.33 perc_conserv=86.67 perc_gaps=0.0
query_cvg=100.0 subj_cvg=88.24 alpha=46.56 beta=2 prob=0.9979833010687846
TTGTTGCAGTCAGTTTGGACACGAACAGCGCGGAGAAACCGGAGCGTACACATAGTACGT
GAGGATTTCGAGCACTGCCCGGGGCCAAAATGA
>AA QryID: NC_000913_(-)_1986575_1986715 HitID: NC_011415_(-)_2203660_2203800
rank=1 bitscore=50.1 evalue=3.0E-4 perc_ident=63.04 perc_conserv=63.04 perc_gaps=0.0
query_cvg=100.0 subj_cvg=100.0 alpha=63.04 beta=12 prob=0.9979771192284685
TTGTTGCTGTGGCGTCACTATAATTTGTTATTTTATTATTTGCAAAAGGTAAAATATAAA
CTTCAATTATTAAAAATACGAATGGGAATAAAACGCATTACCGCTATTTCTTTAATCAGC
GAAGATCCCCTCGCTGATTAA
>AA QryID: NC_000913_(-)_2727283_2727375 HitID: NC_006511_(-)_2617696_2617788
rank=1 bitscore=34.7 evalue=7.0 perc_ident=73.33 perc_conserv=76.67 perc_gaps=0.0
query_cvg=100.0 subj_cvg=100.0 alpha=73.33 beta=1 prob=0.9979721008041866
GTGAAAGTCGCCGACCTCAATATCTCAAAACAGACTGTTAAGTCTTGTTTGATATATTTG
CTCTTTAAAAATCTGGATCAAGCTGAAAATTGA
>AA QryID: NC_000913_(+)_2276492_2276590 HitID: NC_009792_(-)_574175_574273
rank=1 bitscore=36.2 evalue=2.7 perc_ident=70.97 perc_conserv=74.19 perc_gaps=0.0
query_cvg=96.88 subj_cvg=96.88 alpha=68.75 beta=1 prob=0.9979704683016408
TTGATTTTACATAAAATAAACATATATCGGGGGAATGATAGATTTGTGGTTGACATGCAT
CAACAAAGGAAGCCTTTTAGCTTCCTCGTTGTGCAATAG
>AA QryID: NC_000913_(+)_3939387_3939494 HitID: NC_016832_(-)_3723697_3723816
rank=1 bitscore=43.9 evalue=0.013 perc_ident=74.29 perc_conserv=82.86 perc_gaps=0.0
query_cvg=100.0 subj_cvg=89.74 alpha=41.27 beta=2 prob=0.9979642287845393
ATGCCACCAGGAAGTGTGATTACGGTTGCAAAAACGGCAAATTGCTTGTTTTATGGCACA
TTAACGGGGCTTTTGCTGAAAAAATGCGCGGTCAGAAAATTATTTTAA
>AA QryID: NC_000913_(+)_3382388_3382549 HitID: NC_010102_(+)_3486487_3486609
rank=1 bitscore=40.4 evalue=0.24 perc_ident=61.7 perc_conserv=65.96 perc_gaps=14.89
query_cvg=88.68 subj_cvg=100.0 alpha=29.47 beta=1 prob=0.9979545024368213
ATGTGTAGTCACGCAAGTTTAGCGTTTATGCATTTAATTGCCGTAATCAGGAACCTAACT
TACGTAATTAACAGTCGCTTATGCATTAGCGCAACATTCCAACAGGTGGTGACAATATAC
CCTACCGTTCAGCCAAAACAACATCAATTTGATAACAATTAA
>AA QryID: NC_000913_(-)_259393_259545 HitID: NC_009792_(+)_2736596_2736748
rank=1 bitscore=47.8 evalue=0.002 perc_ident=62.79 perc_conserv=74.42 perc_gaps=0.0
query_cvg=86.0 subj_cvg=86.0 alpha=54.0 beta=11 prob=0.9979170292166802
TTGCCAACCATCTGCGAGCAAAGCATGGCGTTTTGTTGCGCGGGATCAGCAAGCCTAGCG
GCAGTTGTTTACGCTTTTATTACAGATTTAATAAATTACCACATTTTAAGAATATTATTA
ATCTGTAATATATCTTTAACAATCTCAGGTTAA
>AA QryID: NC_000913_(+)_2875893_2876120 HitID: NC_018661_(-)_1154515_1154736
rank=1 bitscore=64.3 evalue=2.0E-8 perc_ident=54.29 perc_conserv=57.14 perc_gaps=0.0
query_cvg=93.33 subj_cvg=95.89 alpha=11.61 beta=4 prob=0.9977991908669975
ATGTCTGAACTCCCGGTTTATCCCCGCTGGCGCGGGGAACTCCCGGGGGATAATGTTTAC
GGTCATGCGCCCCCCGGTTTATCCCCGCTGGCGCGGGGAACTCTGGGCGGCTTGCCTTGC
AGCCAGCTCCAGCAGCGGTTTATCCCCGCTGGCGCGGGGAACTCAAGCTGGCTGGCAATC
TCTTTCGGGGTGAGTCCGGTTTATCCCCGCTGGCGCGGGGAACTCTAG
>AA QryID: NC_000913_(-)_915483_915638 HitID: NC_017651_(-)_937022_937177
rank=1 bitscore=48.5 evalue=0.001 perc_ident=58.82 perc_conserv=58.82 perc_gaps=0.0
query_cvg=100.0 subj_cvg=100.0 alpha=58.82 beta=6 prob=0.9977537476222578
TTGCTAATACGTGATGACATCACATTTAAGCATGGCAAATGTCTGAAAAATGAGCGAATA
ATCGCGTTATACTCCCTCTTGCTGATAAGTCTGCAAAAACAACAAAATCAAATAATTAGC
ATGATAAATTATTTTTTATTACGCCTAAAGGATTAA

```
>AA QryID: NC_000913_(+)_3272950_3273075 HitID: NC_011353_(+)_4117246_4117371
rank=1 bitscore=31.6 evalue=60.0 perc_ident=65.85 perc_conserv=68.29 perc_gaps=0.0
query_cvg=100.0 subj_cvg=100.0 alpha=65.85 beta=2 prob=0.9977269495434506
ATGCTGATTTTTATTATTATGGGGAAGGTGTTATTTATGAGTTTCATTTATGCCGTAACG
ACAATGAACTCGGGAATTAGTATAAGCAGCGCGAGAATAATAATCATTGTGCAAATGCTA
ATTTAA
>AA QryID: NC_000913_(+)_3048950_3049063 HitID: NC_017641_(+)_3484449_3484562
rank=1 bitscore=30.4 evalue=128.0 perc_ident=67.57 perc_conserv=67.57 perc_gaps=0.0
query_cvg=100.0 subj_cvg=100.0 alpha=67.57 beta=3 prob=0.9975522218261237
TTGAATAAGACAATATTAAGAGCTAAAAAAATGTCAAAAAACACTAAATCAAAAAATAAT
GGCATTAGAAAATATAATGCGAAAACGGAGGTGAAATTAGTTTATTTCAAATGA
>AA QryID: NC_000913_(-)_2902161_2902307 HitID: NC_012125_(-)_3010730_3010876
rank=1 bitscore=47.4 evalue=0.002 perc_ident=58.33 perc_conserv=64.58 perc_gaps=0.0
query_cvg=100.0 subj_cvg=100.0 alpha=-2.09 beta=1 prob=0.9970088898909029
GTGTTCCCCGCGCCAGCGGGGATAAACCGACGTGGTCATGGGTGCTGCTGTTGCAGAGCC
AGTGTTCCCCGCGCCAGCGGGGATAAACCGAGCAGATACACGGCTTTGTATTCCGTGCGC
CCGTGTTCCCCGCGCCAGCGGGGATAA
>AA QryID: NC_000913_(-)_1268699_1268818 HitID: NC_017652_(-)_1453633_1453752
rank=1 bitscore=40.4 evalue=0.13 perc_ident=56.41 perc_conserv=61.54 perc_gaps=0.0
query_cvg=100.0 subj_cvg=100.0 alpha=56.41 beta=2 prob=0.9939704934403957
TTGGCAACCCGAGCTGGAAGCCTCATCCAGAGCACTCATGAAATGACTGTAACAGCTGTT
GAAAAACCGGAATGCACTCTTCATTGCAAACATTTCAGGGATGAAGTGAAGATGAATTGA
>AA QryID: NC_000913_(-)_1269234_1269353 HitID: NC_017652_(-)_1453633_1453752
rank=1 bitscore=42.0 evalue=0.044 perc_ident=56.41 perc_conserv=66.67 perc_gaps=0.0
query_cvg=100.0 subj_cvg=100.0 alpha=32.44 beta=2 prob=0.9932317750369725
TTGGCAACCCGAGCTGGAAGCATCATCCAGAGCACTCATGAAACGACTGCAACAGCTGTT
GAAAAGCCGGAATGTACTCTTTATTGCAAACATTTCAGGGATGAAGTGAAGATGAATTGA
>AA QryID: NC_000913_(+)_1752529_1752651 HitID: NC_017644_(+)_1730710_1730847
rank=1 bitscore=40.8 evalue=0.098 perc_ident=63.89 perc_conserv=63.89 perc_gaps=0.0
query_cvg=90.0 subj_cvg=80.0 alpha=54.31 beta=8 prob=0.992836876182098
GTGCTGACAATTCAGCAGCCTGATATTAAGCATTCAACTAATACGCTGCTGACTCTGTCC
CCAACACCAGATATTTTCCCTAACCCGATAGTGTATGAGAAAAGATTAACGCCGATAAAC
TAA
>AA QryID: NC_000913_(-)_692641_692733 HitID: NC_013716_(-)_733466_733573
rank=1 bitscore=32.0 evalue=52.0 perc_ident=67.86 perc_conserv=67.86 perc_gaps=0.0
query_cvg=93.33 subj_cvg=80.0 alpha=58.81 beta=1 prob=0.992616612934835
GTGGGCCTTGTTTTTTCTCTCTTTATCCCCATCTTTATTGCAATGCTTGCGCCTTTTTCC
TGTGGCGTGAATAATTTCCTTATAGGCCGGTGA
>AA QryID: NC_000913_(+)_1887790_1887921 HitID: NC_009792_(-)_1136526_1136660
rank=1 bitscore=45.1 evalue=0.005 perc_ident=53.49 perc_conserv=55.81 perc_gaps=0.0
query_cvg=100.0 subj_cvg=97.73 alpha=52.88 beta=8 prob=0.9916073150011555
GTGTTCGTCGTCATCGCAACCCCAAATGATATACATGCCGTTAACATAATATTAACTCAT
CATACCAGCTTGATAATTACCCAACGAAAAGGTTGCGAAGCGCGTCACTATTTATTTTTA
TCTTTACCGTAA
>AA QryID: NC_000913_(-)_1103016_1103123 HitID: NC_009792_(+)_1882829_1882954
rank=1 bitscore=41.2 evalue=0.083 perc_ident=62.86 perc_conserv=68.57 perc_gaps=0.0
query_cvg=100.0 subj_cvg=85.37 alpha=24.53 beta=3 prob=0.9892171056886454
TTGGTTGTTAACGCAACCTGTATTTTGTTAACGCTGCGTTACGATGGAAAGTATGTCTGC
GGAAATATTTTTAATAACTCACCCGCGCGTTGTATTTTTCTTTTTTAG
>AA QryID: NC_000913_(-)_3550856_3550975 HitID: NC_017651_(-)_3850488_3850634
rank=1 bitscore=42.4 evalue=0.033 perc_ident=60.47 perc_conserv=65.12 perc_gaps=9.3
query_cvg=100.0 subj_cvg=89.58 alpha=21.04 beta=5 prob=0.9889101124643196
GTGTCACAATTCCAAATCTTTATTAACAACTCACCTAAAACGACGCTGATCCAGCGTGAA
TACTGGTTTCCCTTATGTTCATCAGATTCATTTAAGCAAGGGTTTCTTCTTCATTCCTGA
>AA QryID: NC_000913_(-)_984972_985097 HitID: NC_016514_(-)_1591882_1592007
rank=1 bitscore=42.7 evalue=0.032 perc_ident=50.0 perc_conserv=67.5 perc_gaps=0.0
query_cvg=97.56 subj_cvg=97.56 alpha=17.93 beta=4 prob=0.9869255192432593
ATGCCGAAAAAACAGGACTTTGGTCCTGTTTTTTTTATACCTTCCAGAGCAATCTCACGT
CTTGCAAAAACAGCCTGCGTTTTCATCAGTAATAGTTGGAATTTTGTAAATCTCCCGTTA
CCCTGA
>AA QryID: NC_000913_(+)_709969_710124 HitID: NC_016856_(+)_757442_757591
```

rank=1 bitscore=38.5 evalue=0.93 perc_ident=52.94 perc_conserv=62.75 perc_gaps=3.92
query_cvg=100.0 subj_cvg=100.0 alpha=28.23 beta=2 prob=0.98613675825633
ATGAAAACCACAAGTCCCTGGCAAATATTGTTAATAAAAACGTGGCACAGCTTACATTTA
CAACGGCAAGGCCACTTGAACACCGGGACATTGTACAGGTACAACAGCAAAAGTTACAAA
TTTGTAGCAATTATTTTGATTGGCATTATCTATTAA
>AA QryID: NC_000913_(-)_4010899_4011045 HitID: NC_015968_(-)_3517198_3517317
rank=1 bitscore=44.7 evalue=0.013 perc_ident=58.97 perc_conserv=71.79 perc_gaps=0.0
query_cvg=81.25 subj_cvg=100.0 alpha=53.45 beta=17 prob=0.9279256744739988
ATGGATTTTGAATTTAGGGAAGGCGGCAAGTTTATTCATCCCCGGGAGCTTACTGAAGTA
AGTGACCGGGGTGGAGAAACGCAGCCAACGCACCATAAATTTAAAAGACGAAGGATTTTA
GCCGTCCAGATGTTTACACATCCATAA
>AA QryID: NC_000913_(-)_2520540_2520698 HitID: NC_018661_(+)_1522679_1522837
rank=1 bitscore=69.7 evalue=4.0E-10 perc_ident=100.0 perc_conserv=100.0 perc_gaps=0.0
query_cvg=100.0 subj_cvg=100.0 alpha=72.54 beta=59 prob=0.7043264849051207
GTGAAAGATATTTTATTTTCTTCCGGCGTTGGTTTTGGTATTGGTGCTTTGTTTACAATC
GTGCGCTTGCCTATTCCTGTACCAAATGTATTACCGGGTATATTATCCATTGTGTTTATG
TATGTCGGATATCTGGTGGTGAAATACTTTATGCCATGA

- ***Mycobacterium tuberculosis* H37Rv uid57777**

vsORFs

>AA QryID: NC_000962_(+)_3597952_3598041 HitID: NC_019965_(+)_3629272_3629361
rank=1 bitscore=96.9 evalue=1.0E-18 perc_ident=100.0 perc_conserv=100.0 perc_gaps=0.0
query_cvg=100.0 subj_cvg=100.0 alpha=56.29 beta=9 prob=0.9999578085549663
ATGACGGAGGTTCCGATGGCCAAGCGTGGCCGTAAGAAGCGTGACCGCAAGTACAGCAAG
GCCAACCACGGCAAGCGGCCCAATTCCTAA

sORFs

>AA QryID: NC_000962_(+)_3331854_3332048 HitID: NC_019952_(+)_3427771_3427965
rank=1 bitscore=93.2 evalue=3.0E-17 perc_ident=81.25 perc_conserv=81.25 perc_gaps=0.0
query_cvg=100.0 subj_cvg=100.0 alpha=43.75 beta=98 prob=0.9999559694862902
ATGGCCGCTGTGTGCGATATCTGCGGGAAAGGCCCCGGCTTCGGCAAGTCGGTGTCGCAC
TCCCACCGCCGCACCAGCCGCCGGTGGGATCCGAACATCCAGACTGTGCACGCCGTGACC
CGTCCCGGCGGCAACAAGAAGCGACTCAACGTTTGCACATCCTGCATCAAGGCGGGCAAG
ATCACCCGCGGCTGA
>AA QryID: NC_000962_(-)_2124830_2125048 HitID: NC_020245_(-)_2095945_2096163
rank=1 bitscore=66.6 evalue=4.0E-9 perc_ident=62.5 perc_conserv=62.5 perc_gaps=0.0
query_cvg=100.0 subj_cvg=100.0 alpha=62.5 beta=49 prob=0.9959664191419434
TTGCTGATGTACGTGTGCTTGTGCGTCGGCGTCACCAACCAAACCGTGTGTGACGCCGTG
GCGCGAGGTGCGTCGACCTCCAAAGAAGTGGCCGCTGTGTGCGGGGCTGGCGGCGACTGC
GGACGCTGCCGGCGCACACTGCGAGCTATCATCGCAGCCGCCCGACTCAACCCAACCCAA
CTCGACCCCGCAGGTCGCACGAGCGACGCCGTTTGCTGA
>AA QryID: NC_000962_(+)_1371529_1371702 HitID: NC_002755_(+)_1371017_1371190
rank=1 bitscore=99.8 evalue=4.0E-19 perc_ident=100.0 perc_conserv=100.0 perc_gaps=0.0
query_cvg=100.0 subj_cvg=100.0 alpha=100.0 beta=70 prob=0.9950166183475792
TTGTACTCCGGCGCTATGAAGTCGATCTCCGTCGGTGAGCTGCGCCAGAATCCCGCTCCC
ATGATCGCCGACCTCGAACGGGGTGAGCCATACGCGCTGACCCGCCACAACCACCGGATC
GGAACGATCATTCCTGCCGTCTCGTCGGCAACACTCATTCCCCGGAAAGCCTAG
>AA QryID: NC_000962_(-)_2260021_2260308 HitID: NC_020245_(-)_2208322_2208561
rank=1 bitscore=140.0 evalue=3.0E-31 perc_ident=97.47 perc_conserv=98.73 perc_gaps=0.0
query_cvg=83.16 subj_cvg=100.0 alpha=56.74 beta=18 prob=0.9919473709845524
GTGCATCGGCAATCCATCCAAGGTCACCGACACAAGGATGGTGCACCAGTGAGCTTCGCG
TGCTCCCATCAAGATCCACGGCGGCGGTGCGCACCACTTGGGGCCGGGCGTTGCCTGCTC
GGCTCGGGTTCGGCGCTAGGCTGGATCATCGACCGCGTCACCACCGACAAAGCGTCCGGA
ATCGCCCAGGATCCCAACGACTGGTGCGGAGAGCACGAGATCCCACCTACATCGTCGATC
GCATCAGAAAGGTCGCACCGGGGACTTGCGCGCGGCCAAAGCGGCTAA
>AA QryID: NC_000962_(-)_2453458_2453736 HitID: NC_016768_(+)_1962686_1962964

rank=1 bitscore=103.0 evalue=3.0E-20 perc_ident=71.74 perc_conserv=71.74 perc_gaps=0.0
query_cvg=100.0 subj_cvg=100.0 alpha=71.74 beta=38 prob=0.9870995756978271
ATGAGCCAAGCCGAGAGGAACGACCCCATGGCACGCAGCGGACCAGACACTAGCGGCGGC
ACAGCCCCCGGGCCGGGGGCGCCGGTGGTAATCGACTGTGACGATTGCGCGGCGCGCGGA
TCTGGATGCCGCGACTGTGTGGTCAGCGTGCTCATCGGGGTACCGGAAAGCCTGTCGCAT
GACGAGCGTGCTGCGTTGGAAGTGCTCGCCGATGTCGGATTGGCCCCTCGGTTGCGCCTG
GTGCCGGTCCGCCGCCAGCGCGGATCCGGTGTCGCGTAA
>AA QryID: NC_000962_(-)_3700356_3700526 HitID: NC_019952_(-)_3818133_3818303
rank=1 bitscore=86.7 evalue=3.0E-15 perc_ident=100.0 perc_conserv=100.0 perc_gaps=0.0
query_cvg=100.0 subj_cvg=100.0 alpha=100.0 beta=12 prob=0.985057124229441
ATGAACGGCGACAGCGCCTCCACCATCGACATCGACAAGGCTGTTACCCGCACACCCGTT
CGCCGGATCGTGCGTTCGGCGCTGGATCGGCTGTGGTTGCGGCAGTCCCAGCCTAGGCGC
TATCGGTTCCTCGAGGACTCGTGCATGGCACGCGCGCTGAATCGACTCTGA
>AA QryID: NC_000962_(+)_1014394_1014555 HitID: NC_017026_(+)_1015002_1015163
rank=1 bitscore=79.3 evalue=5.0E-13 perc_ident=100.0 perc_conserv=100.0 perc_gaps=0.0
query_cvg=100.0 subj_cvg=100.0 alpha=100.0 beta=9 prob=0.9850494596656049
TTGGTGGTGCATATCGTCGGTCTGTCGATCGAGACCACAGCACCGACCGACTCCGCGATT
ACTCCCATCATGGTCCGGGAAATCAACATCGGTGAGATCCCCCTAGGCCTCAGGCTGGGC
AGCGACACCACACTGCTCGACGCCGCTCTCGCGGGTGGGTAA
>AA QryID: NC_000962_(-)_3435615_3435713 HitID: NC_017528_(-)_3429907_3430005
rank=1 bitscore=52.0 evalue=5.0E-5 perc_ident=100.0 perc_conserv=100.0 perc_gaps=0.0
query_cvg=100.0 subj_cvg=100.0 alpha=100.0 beta=3 prob=0.9850402818158946
TTGGTGTTGGTCGAGGTGCCACCGGATAATCCTGAGGTCGTAGCGGGCTGGGTATCGGCG
CTGGCCGACAAATGGATGTTCGCATCGCGGGTTGGGTAA
>AA QryID: NC_000962_(+)_1219089_1219217 HitID: NC_017528_(+)_1219966_1220094
rank=1 bitscore=60.1 evalue=2.0E-7 perc_ident=100.0 perc_conserv=100.0 perc_gaps=0.0
query_cvg=100.0 subj_cvg=100.0 alpha=100.0 beta=4 prob=0.9850392242733381
GTGTCGATCGTTGCCGCTGAATTGGCGCCGATGACCGCAACCGGTATCGCCGCTACGCCG
GCCCGAGGCGGGTACACCACGGTTTTCGAGGGATGGCAATATCCGGGAGTGCGCCGGCTG
GCGGCCTAA
>AA QryID: NC_000962_(-)_2538550_2538645 HitID: NC_002755_(-)_2535754_2535849
rank=1 bitscore=53.9 evalue=1.0E-5 perc_ident=100.0 perc_conserv=100.0 perc_gaps=0.0
query_cvg=100.0 subj_cvg=100.0 alpha=100.0 beta=3 prob=0.9850388164958612
GTGGGGAGGTTGCTGCCGACGGCCGAACTCATTTCTCAACACGGCTCCAAGCCGGTAGGC
ATCGTCGCCACCGACGACATCGAGGCCTTGGCCTAG
>AA QryID: NC_000962_(+)_4080930_4081064 HitID: NC_002755_(+)_4073171_4073305
rank=1 bitscore=68.6 evalue=7.0E-10 perc_ident=100.0 perc_conserv=100.0 perc_gaps=0.0
query_cvg=100.0 subj_cvg=100.0 alpha=100.0 beta=5 prob=0.985037825571317
GTGACTGGACGCAAGGCCGGTGGGGCCCCCTACACCGTCACCATCCCGGAGTTCGGAGCC
GCAGCTTTGCGCGAGCAGCGGGCACTGGTCATCCCGTTCGACCCGGTGTTTCCGGCCCGG
CGCGGCACCCGCTAG
>AA QryID: NC_000962_(+)_1976424_1976519 HitID: NC_017528_(+)_1975805_1975900
rank=1 bitscore=48.5 evalue=5.0E-4 perc_ident=100.0 perc_conserv=100.0 perc_gaps=0.0
query_cvg=100.0 subj_cvg=100.0 alpha=100.0 beta=2 prob=0.9850377691375074
GTGTGGGTCGCCAACGCCGTGCTGCCAGCCTCCGGCAAGCTCGACAGCATCACCGCGGAG
CCGGTTGGCCGCGCGCTGCGGGGACGGCGCGCTTGA
>AA QryID: NC_000962_(+)_380442_380540 HitID: NC_002755_(+)_380503_380601
rank=1 bitscore=43.5 evalue=0.018 perc_ident=100.0 perc_conserv=100.0 perc_gaps=0.0
query_cvg=100.0 subj_cvg=100.0 alpha=100.0 beta=1 prob=0.9850363736961727
GTGACACCACCGCATCGACCGCATACCAATGAGGTTGTCACCGAACCGTATACGGCCCAC
CCGCCGCTATGGTTAACGCTGGCCACCGACCCCTATTGA
>AA QryID: NC_000962_(-)_1623124_1623237 HitID: NC_018143_(-)_1623130_1623243
rank=1 bitscore=52.0 evalue=5.0E-5 perc_ident=100.0 perc_conserv=100.0 perc_gaps=0.0
query_cvg=100.0 subj_cvg=100.0 alpha=100.0 beta=2 prob=0.9850349326001993
ATGTTCCTCGGCCAGGCAACGATCTGGGCAGGTTGGGCGATCCTCTGTGGCAGGCTCCCG
GTAGCCACCGGCCTCGCCGTATTCGTCGGCATTTGGTTCGTCGCGTCTCCGTAA
>AA QryID: NC_000962_(-)_3822122_3822217 HitID: NC_002755_(-)_3812999_3813094
rank=1 bitscore=45.8 evalue=0.003 perc_ident=100.0 perc_conserv=100.0 perc_gaps=0.0
query_cvg=100.0 subj_cvg=100.0 alpha=100.0 beta=1 prob=0.9850344883693242
ATGCGCCGCACCTTCCTCATCTGCCCCACGCGTACCGACCCGGACCGACTACGCGAGGCG
GCCATATCGCTAGCCCTGTGGCGCCGGGACGAATGA
>AA QryID: NC_000962_(-)_3232513_3232608 HitID: NC_002755_(-)_3226665_3226760

rank=1 bitscore=46.6 evalue=0.002 perc_ident=100.0 perc_conserv=100.0 perc_gaps=0.0
query_cvg=100.0 subj_cvg=100.0 alpha=100.0 beta=1 prob=0.9850338190996897
ATGGCGGCACCATCGCCGCAACAACTAGTGCGCATGGTTCTGGATCAGGTCGTCAGTCGT
AAGGCTGGGGCCGTGCCAACGCTCGACTGGAGGTGA
>AA QryID: NC_000962_(+)_1957266_1957385 HitID: NC_002755_(+)_1948124_1948243
rank=1 bitscore=47.4 evalue=0.001 perc_ident=100.0 perc_conserv=100.0 perc_gaps=0.0
query_cvg=100.0 subj_cvg=100.0 alpha=100.0 beta=1 prob=0.9850331428055461
GTGGCGGCCGATCTGAACAGCTTCGTGGGGGGATCCGCTTCGTCAACCAACGCGGAAAGC
ATGGCTTTGGCCTTCCGCGGTCGCGTCCACATGAGTGTCAATATAGCTGGACTAACATGA
>AA QryID: NC_000962_(-)_2179566_2179658 HitID: NC_002755_(-)_2176921_2177013
rank=1 bitscore=47.4 evalue=0.001 perc_ident=100.0 perc_conserv=100.0 perc_gaps=0.0
query_cvg=100.0 subj_cvg=100.0 alpha=100.0 beta=1 prob=0.9850331428055461
ATGCGGTTTGGCTCCCTTGCGCTGGTCGCCTACGACTCGGCCATCAAGCATTCATGGCCA
CGCCCGTCGTCGGTGCGCCGGCTACGGATGTGA
>AA QryID: NC_000962_(-)_4360028_4360123 HitID: NC_019965_(-)_4370554_4370649
rank=1 bitscore=55.8 evalue=3.0E-6 perc_ident=100.0 perc_conserv=100.0 perc_gaps=0.0
query_cvg=100.0 subj_cvg=100.0 alpha=100.0 beta=2 prob=0.9850317056806246
GTGAGTACGCATTGTCAATGTTTTGACATGGATCGGCCGGGTTCGGAGGGCGCCATAGTC
CTGGTCGCCAATATTGCCGCAGCTAGCTGGTCTTAG
>AA QryID: NC_000962_(-)_2270447_2270563 HitID: NC_020245_(-)_2223745_2223861
rank=1 bitscore=58.2 evalue=6.0E-7 perc_ident=100.0 perc_conserv=100.0 perc_gaps=0.0
query_cvg=100.0 subj_cvg=100.0 alpha=100.0 beta=2 prob=0.9850295861012299
ATGCTCGGCGACGAACTCGCAGCTCAGCGTGGCGTCCTTCCCTCGAAAGAAGGTGCGCAC
GAAACCCGGGAGGCTTCACTATGTGAAGGTCACCTCAACCTCGAACTGGCCGGCTGA
>AA QryID: NC_000962_(-)_522173_522283 HitID: NC_002755_(-)_523622_523732
rank=1 bitscore=52.0 evalue=4.0E-5 perc_ident=100.0 perc_conserv=100.0 perc_gaps=0.0
query_cvg=100.0 subj_cvg=100.0 alpha=100.0 beta=1 prob=0.985029115633671 3
TTGTCGAGCCGGTTCCGCACCCGGATGCGCTTGCCGGCGGTGACGGGTCCGTATATGGTG
CTCGGCGCAGTGAATGTGGTTCAGGCCCAGTGGGTCAGACGCACCGCGTAG
>AA QryID: NC_000962_(-)_4319855_4319956 HitID: NC_018143_(-)_4320033_4320134
rank=1 bitscore=52.4 evalue=3.0E-5 perc_ident=100.0 perc_conserv=100.0 perc_gaps=0.0
query_cvg=100.0 subj_cvg=100.0 alpha=100.0 beta=1 prob=0.9850287541092613
ATGGTCGAGATGCTGGGTGTGTGTGTAGTCGCGACGGATCTCGACGGGCATCGGATTGTG
TTGCGCGACTCTGAAAAGACGCTGAAAATGAGAAACCGATAG
>AA QryID: NC_000962_(-)_11555_11692 HitID: NC_017528_(-)_11560_11697
rank=1 bitscore=79.3 evalue=5.0E-13 perc_ident=100.0 perc_conserv=100.0 perc_gaps=0.0
query_cvg=100.0 subj_cvg=100.0 alpha=100.0 beta=5 prob=0.9850287315035453
GTGCTGGTCGCTTACATAGACGAGTCCGGCAATACCGGAGACCCCGCCAACGGCGGATCC
ATGACTTTCGCTCTAGGGTGCGTGCTCGTGGACGCCGACAACCGGCCGACCGCGTTCGAT
GGGTTGCTGAGCTTCTAG
>AA QryID: NC_000962_(-)_4359823_4359951 HitID: NC_020245_(-)_4252679_4252807
rank=1 bitscore=59.7 evalue=2.0E-7 perc_ident=100.0 perc_conserv=100.0 perc_gaps=0.0
query_cvg=100.0 subj_cvg=100.0 alpha=100.0 beta=2 prob=0.985028228428318
GTGACCACCCGGCTGATCGCCCAAGCGGTGCATCATGCGCGCGGATTCAACGGGTTACTG
CGAATACCGGCGCGGGGTGGATCCAGCGGCCGAGCCGGCGTGAAATGCCGGAGGCCAACC
GGACGGTGA
>AA QryID: NC_000962_(+)_577506_577613 HitID: NC_017026_(+)_577884_577991
rank=1 bitscore=60.5 evalue=1.0E-7 perc_ident=100.0 perc_conserv=100.0 perc_gaps=0.0
query_cvg=100.0 subj_cvg=100.0 alpha=100.0 beta=2 prob=0.985027493795716
GTGGAGGTCAAGACGCCCGCCGGTGATGGGCTGGTGGCGCTCACCCCGTTCCGGACTCAG
AAATTCGCGATCACAATTTGCGCGTTCAAGTCATTGGCATGCATGTGA
>AA QryID: NC_000962_(+)_2538422_2538553 HitID: NC_002755_(+)_2535626_2535757
rank=1 bitscore=60.8 evalue=1.0E-7 perc_ident=100.0 perc_conserv=100.0 perc_gaps=0.0
query_cvg=100.0 subj_cvg=100.0 alpha=100.0 beta=2 prob=0.9850272163995435
ATGTGGGCGCCGGTGATCTGGATAGCGACATTGCCGCAACGCCCGGTTGGTCAGCAAATG
GTGCCCATGCTGGCGACCAACGGGACCTCCGGCGCGGTAAGGCAGCCGGGCTCCAGTAAT
CCCAGCGGCTAG
>AA QryID: NC_000962_(+)_3642982_3643116 HitID: NC_002755_(+)_3637882_3638016
rank=1 bitscore=68.2 evalue=1.0E-9 perc_ident=100.0 perc_conserv=100.0 perc_gaps=0.0
query_cvg=100.0 subj_cvg=100.0 alpha=100.0 beta=3 prob=0.9850265815720285
GTGGCTCCTCGGCTCGAAATTCTCCAAGAAGGACGACAGGACTTAGGCGCCGTGATAGAT
GCCGCTGTGGGCGGCGCACTGTCGGTGATGCTCGGCAACATCCCATTGGTGGTTCCGAAC

GCCAACCAGCTGTAA
>AA QryID: NC_000962_(+)_876424_876570 HitID: NC_017026_(+)_876948_877094
rank=1 bitscore=55.1 evalue=1.0E-5 perc_ident=100.0 perc_conserv=100.0 perc_gaps=0.0
query_cvg=100.0 subj_cvg=100.0 alpha=100.0 beta=1 prob=0.9850262652099606
TTGGCTCCGAACATGGCCAGCATCAGCGCCAGCGAGCATACCGCCGCTGCCATGCGGGTG
TCGGGCAGCAACAGGCCCACGGCGACCAGGAGCCTCCAGCGCACCGGTGATGGTGACCAG
CAGGCCGGGCGCAAGCAGCCCGGGTGA
>AA QryID: NC_000962_(-)_3719764_3719871 HitID: NC_002755_(-)_3717373_3717480
rank=1 bitscore=55.1 evalue=5.0E-6 perc_ident=100.0 perc_conserv=100.0 perc_gaps=0.0
query_cvg=100.0 subj_cvg=100.0 alpha=100.0 beta=1 prob=0.9850262652099606
TTGCGGTACAAGCCGCGCGGCGCCGGTAGCCCTCGCCAGAGTGACCGGGTCGCCGGGGCA
ACCGTCAACGTTGACCGAGTACTCACGGTGCGAGCCTGGGCATTGTGA
>AA QryID: NC_000962_(-)_2497225_2497416 HitID: NC_017026_(-)_2490668_2490859
rank=1 bitscore=97.4 evalue=2.0E-18 perc_ident=100.0 perc_conserv=100.0 perc_gaps=0.0
query_cvg=100.0 subj_cvg=100.0 alpha=100.0 beta=7 prob=0.9850243840114443
TTGCACGGCATCGGGTTGCCGTCGCCGGGCGGGCGGGCCGAACTCTCGGAGATCTGCGGC
CACATCGCGTCGCTGCCGGCGACCGCCGTGGGAGATGGGTTCGTCGAAGGGGTGGACGGC
ATCGGGGATCGCCCTCGGGCGATGGCCAAGGTGCACCATGCCGGCGTGGACGGGGTGCCA
GCGCCAACCTGA
>AA QryID: NC_000962_(+)_3893958_3894080 HitID: NC_002755_(+)_3887791_3887913
rank=1 bitscore=58.5 evalue=5.0E-7 perc_ident=100.0 perc_conserv=100.0 perc_gaps=0.0
query_cvg=100.0 subj_cvg=100.0 alpha=100.0 beta=1 prob=0.9850230078346842
GTGTTGGGGTCGATTTGGCCGGACCAGTCGTCACCAACGCTTGGCGTGCGCGCCAGGCGG
GCGATCAGATCGCTTGACTACCAATCAATCTTGAGCTCCCGGGCCGATGCTCGGGCTAAA
TGA
>AA QryID: NC_000962_(-)_901365_901472 HitID: NC_002755_(-)_901274_901381
rank=1 bitscore=59.7 evalue=2.0E-7 perc_ident=100.0 perc_conserv=100.0 perc_gaps=0.0
query_cvg=100.0 subj_cvg=100.0 alpha=100.0 beta=1 prob=0.985021824504452
GTGTCTTGCAAGAGTTATCGCCTTGACCGCACAATTCTGTATGGACCCCGCGCCAGCGCG
GTCAACCTGCGCCGACCCGCGATCTGTGAGATTCTTTCGGTGACCTAA
>AA QryID: NC_000962_(-)_4190718_4190828 HitID: NC_002755_(-)_4183031_4183141
rank=1 bitscore=62.0 evalue=4.0E-8 perc_ident=100.0 perc_conserv=100.0 perc_gaps=0.0
query_cvg=100.0 subj_cvg=100.0 alpha=100.0 beta=1 prob=0.9850195060663742
GTGCTGCGGGTCGCGCTGGGTTTGACCAAAGCCAGCCCAGTTTATCTGCCTGGTGTAAAG
GGACGTATCTTGCTGGCTGGGCAACTCGAGCATCGTAGCTCGTGGAAATGA
>AA QryID: NC_000962_(+)_2637491_2637601 HitID: NC_002755_(+)_2634822_2634932
rank=1 bitscore=62.4 evalue=4.0E-8 perc_ident=100.0 perc_conserv=100.0 perc_gaps=0.0
query_cvg=100.0 subj_cvg=100.0 alpha=100.0 beta=1 prob=0.9850190959977085
ATGTGCCCGACGCTCGATGCGCACCAATTCGAACCAACTCAGGTCTTACGCTGCCTGGAC
GCCGAACTAGCTCGATCCAGCGCCGACCCGCACCCCACTACCGGCATCTGA
>AA QryID: NC_000962_(+)_1163690_1163827 HitID: NC_002755_(+)_1163734_1163871
rank=1 bitscore=63.9 evalue=2.0E-8 perc_ident=100.0 perc_conserv=100.0 perc_gaps=0.0
query_cvg=100.0 subj_cvg=100.0 alpha=100.0 beta=1 prob=0.9850175398346066
TTGGTGCACTGGATCGGGTCCAAGCGCTACATTCGCCGTCAAGCCTCCACAGCCCGATTG
GCAGAGGCAGCGGACAATCCGCGCTCACGGGTGCTGGCGTTTGCTAGTGCCGGTAATCTT
CGAAAGAGTCGCTTCTAA
>AA QryID: NC_000962_(+)_1170755_1170907 HitID: NC_002755_(+)_1170801_1170953
rank=1 bitscore=84.3 evalue=2.0E-14 perc_ident=100.0 perc_conserv=100.0 perc_gaps=0.0
query_cvg=100.0 subj_cvg=100.0 alpha=100.0 beta=4 prob=0.9850172772503237
TTGCTGTTGTCAACTCTTGCGAGTTACGTGAGTGCGGCCGGAGCACACGCTCGTATCGTC
GTCACAGTCGAAGGGCGCGATCTTGAGTTCGACGTATCGACCTTCGCCCTTGTGGGCCCG
CAGCAGCTGCCCGAAGTCGAGCCGTCGCAGTAG
>AA QryID: NC_000962_(-)_2582477_2582644 HitID: NC_002755_(-)_2578323_2578490
rank=1 bitscore=85.5 evalue=7.0E-15 perc_ident=100.0 perc_conserv=100.0 perc_gaps=0.0
query_cvg=100.0 subj_cvg=100.0 alpha=100.0 beta=4 prob=0.9850160070884078
ATGACGGCGACATCTGTGGTTCGGCTGGGCACCAAGGCCGCAGCCGAATACCTCGGCGGC
CTACCTGAGTCGACGCTGAGGTACTGGCGTTACTGGGCACCAGTCCGCGCAGCTATCGCC
TTGGCCGACACACGTTTTGCGATATCGCCGACCTGGATGCGTGGGTAG
>AA QryID: NC_000962_(+)_747979_748098 HitID: NC_020245_(+)_749721_749840
rank=1 bitscore=67.8 evalue=9.0E-10 perc_ident=100.0 perc_conserv=100.0 perc_gaps=0.0
query_cvg=100.0 subj_cvg=100.0 alpha=100.0 beta=1 prob=0.9850133546535038
GTGCGGCGTCGCGGGATTCACACTCGGCGATACGTGTCGCCGTTTTGGCTGACCGGACCG

GGCCAGGCTATTGTGGTTGCCGATCCACCGAAGACCGTCGGTCACCGAGCAATCGGTTGA
>AA QryID: NC_000962_(+)_1728630_1728761 HitID: NC_002755_(+)_1728784_1728915
rank=1 bitscore=68.6 evalue=5.0E-10 perc_ident=100.0 perc_conserv=100.0 perc_gaps=0.0
query_cvg=100.0 subj_cvg=100.0 alpha=100.0 beta=1 prob=0.9850124706921974
ATGATTGATTCATCAGTCAATATTAAAGTCGCAGCTCACAACTATAATACGCCGGTGCAG
CGGACAATTGCGGAAGCGCCGGACGCCTCGCGGTCCGATGTCGCCTTTCCCTGCCTCGTC
GTCAATATCTGA
>AA QryID: NC_000962_(+)_130581_130706 HitID: NC_002755_(+)_130572_130697
rank=1 bitscore=72.8 evalue=2.0E-11 perc_ident=100.0 perc_conserv=100.0 perc_gaps=0.0
query_cvg=100.0 subj_cvg=100.0 alpha=100.0 beta=1 prob=0.9850076825277734
ATGGCGTATCAGCGCCATGTCTACCGACTTGCGCATACTTCGCCGGGTGAGGTCGCCGGT
GAAGGCAGTCCGGACCCCTTTGGTCTGCGAGCGATGAATGCAGACGCCGTGTCGGATCTA
GCTTGA
>AA QryID: NC_000962_(+)_4019074_4019208 HitID: NC_020245_(+)_3927887_3928021
rank=1 bitscore=72.8 evalue=4.0E-11 perc_ident=100.0 perc_conserv=100.0 perc_gaps=0.0
query_cvg=100.0 subj_cvg=100.0 alpha=100.0 beta=1 prob=0.9850076825277734
ATGGCCGACGGTCCCCTTGCCATCACGGCGAAATCGGTTTACATACATGGCTATTCGGTA
GATACGGCAGAGATTCCAACAGCTGTGCGTGGCCACCCGAATGCCGCGGGAACCGCGATC
AAGGACCGCCGCTGA
>AA QryID: NC_000962_(-)_3202034_3202177 HitID: NC_020245_(-)_3125615_3125758
rank=1 bitscore=86.3 evalue=4.0E-15 perc_ident=100.0 perc_conserv=100.0 perc_gaps=0.0
query_cvg=100.0 subj_cvg=100.0 alpha=100.0 beta=3 prob=0.9850076034906874
ATGGACTGGCTTGTTCGACGACCGGGGCCGCTGTCAGCGATCAGCGACCTGAGGCTGGCG
ATTCAAGACCAGTTCGTGACCGCATCACGTACCACTGCCTATTCGGGTGGGGCGCGTAAC
CACGCCGTGATCCCGGCCGGCTAG
>AA QryID: NC_000962_(-)_3067972_3068067 HitID: NC_019951_(-)_3189590_3189685
rank=1 bitscore=51.6 evalue=6.0E-5 perc_ident=96.77 perc_conserv=96.77 perc_gaps=0.0
query_cvg=100.0 subj_cvg=100.0 alpha=96.77 beta=5 prob=0.9850073745231906
GTGGCACTGGCCAGTCACCTCGACATCCCCCTGCTGACCGCGGACCCGCGTCTGCGCCGG
GAGCCCGGGCTGACCTGCCACACCGAGTTCGTCTAG
>AA QryID: NC_000962_(-)_3558629_3558853 HitID: NC_017528_(-)_3553028_3553252
rank=1 bitscore=109.0 evalue=4.0E-22 perc_ident=100.0 perc_conserv=100.0 perc_gaps=0.0
query_cvg=100.0 subj_cvg=100.0 alpha=100.0 beta=6 prob=0.9850046280356461
GTGCTCAGGAACGGGAGCAGCACCGTCGTCAGGCGAGCGCCAACCGCGAACCGTGACGCG
GCCGGTGGCGGCATCAAGATGCCGCCACCGGCCGCGCCGGATGCCCCTGTACAACGGCCC
GACTACTCCGAAACCGACTGGGACGCGCTACGGGCCGCTTATCCACGTGACGCTGAACGG
GTGAAGCGGGCCATGTTCATTGTGTCAGCGGCAACCGAAAACTGA
>AA QryID: NC_000962_(-)_4320254_4320433 HitID: NC_002755_(-)_4312578_4312757
rank=1 bitscore=100.0 evalue=2.0E-19 perc_ident=100.0 perc_conserv=100.0 perc_gaps=0.0
query_cvg=100.0 subj_cvg=100.0 alpha=100.0 beta=4 prob=0.9849990694170873
GTGTGGCGAAGGTCTATGGGGTGGGTGGGCAAGAAGAAGTCGACGGCTGGGCAGTTGGCG
GGGACGGCTAACGAATTGACCAAGGAGGTGCTGGAGCGGGCCGTGCACCGCGAGTCGCCG
GTGATCCGGCCGGACGTGGTAGTGGGAATTCCCGCAGTGGATCGACGCCCAAAACAGTGA
>AA QryID: NC_000962_(-)_986734_986880 HitID: NC_002755_(-)_986746_986892
rank=1 bitscore=84.0 evalue=2.0E-14 perc_ident=100.0 perc_conserv=100.0 perc_gaps=0.0
query_cvg=100.0 subj_cvg=100.0 alpha=100.0 beta=1 prob=0.9849936210961682
TTGAACGTCATCGCAATTCCCGCAGTGGGTGAGTACCTGACGCTCCTGTGGTTTTCCTCA
GGTGAGGGCATCAAAGTTAGCGGGACGGTGGTGTGTCTCGACGCTCTGCACGACCTCGGC
GTTGTTCGTCCGGGAATATTGGGTTAG
>AA QryID: NC_000962_(+)_3557099_3557284 HitID: NC_017528_(+)_3551494_3551679
rank=1 bitscore=105.0 evalue=8.0E-21 perc_ident=100.0 perc_conserv=100.0 perc_gaps=0.0
query_cvg=100.0 subj_cvg=100.0 alpha=100.0 beta=4 prob=0.9849924919979305
ATGGATCATCCTACTGAGCGGGAATGGGCCAGCATCGCCGAACATACACGCGCCTCCAAC
TTCACCGGCGACCTGTTACGAATGCCGCCTTACCCGCTGATCCTCACCCTCCGAACGCTG
GTGGGGTCTGCCGAGGTGGTCACTGCATCACATACCCTCTTCCTGTCGGCGGCAACTGAA
TACTGA
>AA QryID: NC_000962_(-)_1634821_1634979 HitID: NC_002755_(-)_1634936_1635094
rank=1 bitscore=88.6 evalue=7.0E-16 perc_ident=100.0 perc_conserv=100.0 perc_gaps=0.0
query_cvg=100.0 subj_cvg=100.0 alpha=100.0 beta=1 prob=0.9849872551596914
ATGGTAGACCGGGTAACTGTGGCAGCAGAGCCGCAGGGTCGATTCACACGACTTCGCCGG
CATATCGGCACCGCTCGCCAGCCCGAACCGCGGTCAGGACAATTTTCACACCAGCCCGAT
GAAAATCGCTATCTGGGATTGATCAGTGTGACGCCCTAA

>AA QryID: NC_000962_(+)_4057488_4057649 HitID: NC_002755_(+)_4049729_4049890
rank=1 bitscore=90.1 evalue=3.0E-16 perc_ident=100.0 perc_conserv=100.0 perc_gaps=0.0
query_cvg=100.0 subj_cvg=100.0 alpha=100.0 beta=1 prob=0.9849850996312893
TTGGTTGACCTGCTGCTTTTGTCCGCGAATTCACCGGAATTTGAACTGGACCTGGCCGGC
AATCGTGGGGCAGTCACTGTGAGCTGTAGCCATGCCAGCTGCACAGGAAGTGCGATCCGG
ACGTCAAGGGAGGCCCGACTGGTCCGGCCGGCCGATCAATGA
>AA QryID: NC_000962_(+)_838129_838371 HitID: NC_002755_(+)_840318_840560
rank=1 bitscore=93.2 evalue=4.0E-17 perc_ident=100.0 perc_conserv=100.0 perc_gaps=0.0
query_cvg=100.0 subj_cvg=100.0 alpha=100.0 beta=1 prob=0.98498051604753
ATGGGGGCCGCCGCGCCAGGTATCTCGGCGAAGATCCCCGGCGCTCGAGCGCTTTGTCAG
AGGCCCGTCGCGGGTCGTCGTGACGACGGCTATCCGGGCGGTGCGGGTTTCGCGGCGCGC
CCTGTGCCCGGCACCGCCGCCCGTTTGTCGGCAACGCCGCCGCGACCCGTGAGCCGTCCA
GCAGCTGGCGCCTGCGAAACGTGTGGAAGCGCTGCATGCGGTGCCGGATCGCGATATCGT
TGA
>AA QryID: NC_000962_(+)_1617565_1617729 HitID: NC_018143_(+)_1617571_1617735
rank=1 bitscore=97.8 evalue=2.0E-18 perc_ident=100.0 perc_conserv=100.0 perc_gaps=0.0
query_cvg=100.0 subj_cvg=100.0 alpha=100.0 beta=1 prob=0.9849733826933016
ATGTTTGGCCACGAACGGAATCGCGACAGCGCCACGGCGTCGAGCCTCGCCATGCACCTA
GCCGGCGCCTTTGAACTCGTGAGCGGACCGAAGTGGACCGCCTGTCGCTTCGAGGCGGGC
ACAGTGCGTATTCCCTCGCAAGGGAAGCGCCGGTGGCAGGCGTGA
>AA QryID: NC_000962_(+)_3457552_3457764 HitID: NC_017528_(+)_3451857_3452069
rank=1 bitscore=113.0 evalue=4.0E-23 perc_ident=100.0 perc_conserv=100.0 perc_gaps=0.0
query_cvg=100.0 subj_cvg=100.0 alpha=100.0 beta=2 prob=0.9849591479669438
TTGGATCACCCGCCGCGTGCTGCGGCTGGTGTCCAGCCAGGCGATTGCCCGCGCTCGCAC
CCGCTCGGCGCGCGGGTTGTCGGCGACCCCGAAGATGACCTCGGCGACGATGTCGAGCGC
GATCGGGCCGGCGCGGTCTCGGAAACGGACCTCCTCGCCCATTGGCCAGGTGGCGAGCGC
CTTTCGGTCACGCGTTCCATGGCCCGCTCGTAG
>AA QryID: NC_000962_(-)_2870482_2870691 HitID: NC_002755_(-)_2866627_2866836
rank=1 bitscore=110.0 evalue=2.0E-22 perc_ident=100.0 perc_conserv=100.0 perc_gaps=0.0
query_cvg=100.0 subj_cvg=100.0 alpha=100.0 beta=1 prob=0.984952388277586l
TTGGACCGTCGCCGTCGAGGCGGCGTTGCCGCATGCTTGCTCGTAACGGGAGTTTCATGT
CGGTCACCGCGCGCCGGGCTCGAACGTGAGCGATCTTGCATGGGAACCATGGCGAACTCA
GCGGCTGATTACGACGCGCGTAAAGGGGCGGACACTCGCTTCGCCCCTGTACAGAAGTCA
GCACTGTCATCGCTTTGGCCAGTCGCCTAG
>AA QryID: NC_000962_(+)_1928856_1929071 HitID: NC_002755_(+)_1919658_1919873
rank=1 bitscore=125.0 evalue=9.0E-27 perc_ident=100.0 perc_conserv=100.0 perc_gaps=0.0
query_cvg=100.0 subj_cvg=100.0 alpha=100.0 beta=1 prob=0.984921959268686
ATGTCGTCGACGGTGGGTACGGAGCCGAAGGCGTTGCCGTCGTCGACGACGCTGGCGAAT
AGGTTTGAGGTCCAGCCCGAAGCCGCGGGCTTGAGGCTGATGAGGAAAACCGGCGCGTTG
CGCTCGTTGAACTGCTGGATCACATTGGGGCGTCATCGAGGTCGATCGACGGATACATCA
GGGAATGCATGGCCGCACCGTATCGACTCGGTCTGA
>AA QryID: NC_000962_(-)_157681_157812 HitID: NC_020245_(-)_157911_158036
rank=1 bitscore=53.1 evalue=2.0E-5 perc_ident=97.56 perc_conserv=100.0 perc_gaps=0.0
query_cvg=95.35 subj_cvg=100.0 alpha=95.29 beta=1 prob=0.9846952497616841
TTGTGCGTGCGGTCGTTGCGCCGCTCGGGAGCCAGCCGTGACCCGGGTGTGTCAATCGCC
CAAGGTGTCCGGGCCGACGCTGAATCGTTAGCCAACCGCGATCTCGCGCTGCGGCCACGA
CATTCGAACTGA
>AA QryID: NC_000962_(-)_1272036_1272191 HitID: NC_002755_(-)_1271525_1271680
rank=1 bitscore=87.8 evalue=1.0E-15 perc_ident=100.0 perc_conserv=100.0 perc_gaps=0.0
query_cvg=100.0 subj_cvg=100.0 alpha=78.23 beta=33 prob=0.984500985516026
ATGGCGGCTTTTAGGCTCGCTGTCATGTGGATCGACGACTCGAGTGCCGACGTCATCAAG
GCTGACTTCGAGGCTCTCTACCACGGCGACATGCTGGTAGAGGGCGACACCTCCGAGCAG
CTCGAAGACTTGCACCCGCTGCCCACCGCGAGCTGA
>AA QryID: NC_000962_(+)_4319825_4320040 HitID: NC_018078_(+)_4307640_4307855
rank=1 bitscore=104.0 evalue=2.0E-20 perc_ident=90.14 perc_conserv=90.14 perc_gaps=0.0
query_cvg=100.0 subj_cvg=100.0 alpha=90.14 beta=5 prob=0.9844908743647276
ATGGTCTTCGACAAGCCCACCGTGAGTTGTCTATCGGTTTCTCATTTTCAGCGTCTTTTC
AGAGTCGCGCAACACAATCCGATGCCCGTCGAGATCCGTCGCGACTACACACACACCCAG
CATCTCGACCATCGCGACTCCGGCCGACGACGGCTAACGAGCAGCTTCGCCCCACCCGCC
CCCGCAGCAACAACACAACGGCACGGCAGCAGCTGA
>AA QryID: NC_000962_(-)_4317685_4317897 HitID: NC_017528_(-)_4312672_4312908

rank=1 bitscore=121.0 evalue=1.0E-25 perc_ident=98.57 perc_conserv=100.0 perc_gaps=0.0
query_cvg=100.0 subj_cvg=89.74 alpha=76.1 beta=8 prob=0.98437176689874
TTGGCTATCAGGGTGGACCTTGACGGGCGCAAAGACGCCTCGGGCCGGCCGATCCAAGAC
AGCCCGGGGCGTCTCGTCGATGCCGGGATTCATGGCGCCGTCACCGTGGAGGCCGCCGAG
CTCGCGCTCAAGGAATTTTGTCTGGGCGCATGCGTAGCCGGAATTCGTGCCATTCCTGGA
CTATCCGATCGAGCTGCGCAAGTTAATCAATAA
>AA QryID: NC_000962_(+)_1282030_1282218 HitID: NC_019951_(+)_1337863_1338051
rank=1 bitscore=51.2 evalue=1.0E-4 perc_ident=74.19 perc_conserv=74.19 perc_gaps=0.0
query_cvg=100.0 subj_cvg=100.0 alpha=74.19 beta=29 prob=0.9842336994366719
ATGGGTGAATCGAAGTCCCCGCAAGAGTCCAGCTCAGAGGGTGAGACCAAGCGCAAGTTC
CGGGAAGCCCTCGACCGCAAGATGGCACAGTCGTCGAGCGGATCCGATCATAAGGATGGC
GGCGGCAAGCAGTCGCGGGCGCACGGTCCGGTGGCGAGCCGTCGGGAATTCCGCCGCAAG
AGCGGCTAG
>AA QryID: NC_000962_(+)_2632565_2632792 HitID: NC_012943_(-)_1783799_1784026
rank=1 bitscore=82.4 evalue=7.0E-14 perc_ident=82.67 perc_conserv=82.67 perc_gaps=0.0
query_cvg=100.0 subj_cvg=100.0 alpha=82.67 beta=6 prob=0.9834778377146944
TTGCTTGCATTCTGGCTCCGGGGCATAGCGACAAGTGTTGCCCTGGCTGTTGACGTGCTC
TTCGGGCAAGCGGACTTCACGCTTTCAAGCGTGCACTCGGCCGAACTTGCCAGCGCGAAC
TCCACCAGCGGACACCTTCAGATCGCGATGGTTGTGCTGGCGCTGCTGATCGCCGGGCTC
ACGGCCGGAGGGGCTTTCCGCATGGCCAGCGGACTGGGCCACGCCTAA
>AA QryID: NC_000962_(-)_3288026_3288196 HitID: NC_020245_(-)_3211664_3211834
rank=1 bitscore=72.8 evalue=5.0E-11 perc_ident=82.14 perc_conserv=82.14 perc_gaps=0.0
query_cvg=100.0 subj_cvg=100.0 alpha=82.14 beta=2 prob=0.9829573800223941
GTGACGGTGGCCGGCGACGGAGATGGGCTGGTGCTTGGCCTGAGCGCCATGTGCGATGTG
GCGTCGTTCATGGGCGCCGTCGCCCAACGTGAACTCAGGGCGGAAAATCGGCGCCGGATT
TCGCCCTCAGTGCACGCTCGGCACCGGTTTAACTTCACGCGGTCAACGTGA
>AA QryID: NC_000962_(-)_3848844_3848999 HitID: NC_018078_(-)_3839772_3839927
rank=1 bitscore=57.8 evalue=1.0E-6 perc_ident=78.43 perc_conserv=78.43 perc_gaps=0.0
query_cvg=100.0 subj_cvg=100.0 alpha=78.43 beta=7 prob=0.9827902705189049
TTGGCGGCGCTGTCGGCGCGGCGGGGCCCCAAGCCGGGCAAGGCCGGCGCCAATGCCGCT
GACGCCGAGATCGCGTGGCTGCGCGCCGAGCTCGACACCGCACGCGAGGTGATCCGTGTG
CAGGGGGAACTGTCCGCGCTCTTGGAGAGGCTCTGA
>AA QryID: NC_000962_(-)_639972_640106 HitID: NC_002755_(-)_641424_641558
rank=1 bitscore=74.7 evalue=1.0E-11 perc_ident=100.0 perc_conserv=100.0 perc_gaps=0.0
query_cvg=100.0 subj_cvg=100.0 alpha=83.53 beta=1 prob=0.9827827848916948
ATGATCGAGTACGCGGTGACCCTCCACAGAAAGCCGCCCGGGCGCCAACTCACGACCACA
TGTGACCAAGGCCCCGGGCACCATACCGTCCGGCTGACGCCCAGCGGCCATGATTACTGT
GGCAGGCATGGGTGA
>AA QryID: NC_000962_(+)_2959898_2960095 HitID: NC_017026_(+)_2953623_2953820
rank=1 bitscore=95.9 evalue=6.0E-18 perc_ident=83.08 perc_conserv=83.08 perc_gaps=0.0
query_cvg=100.0 subj_cvg=100.0 alpha=83.08 beta=1 prob=0.9825656474176786
GTGAATCAGCACAAAGAAGCCTGTGCAGTAGCGCGACGCTCGGCGTACCGGCACGAGTCC
GACGGCCCGCATGTCCATGCGGCCGCCGGCACCAGCGCCGAGGCCCCCGCAGGATACCGG
GATCTGCAGCTCCTCGTGCGGAAACAGTTGCCGCAGTTCGGGTTCGGGCAGTTCGGCGAG
CGTGATGTTCGCGCCTAA
>AA QryID: NC_000962_(+)_828947_829159 HitID: NC_009525_(+)_830253_830465
rank=1 bitscore=80.5 evalue=3.0E-13 perc_ident=80.0 perc_conserv=80.0 perc_gaps=0.0
query_cvg=100.0 subj_cvg=100.0 alpha=80.0 beta=5 prob=0.982463381818286
GTGGTCGGGCGTCGACCACCTGCTCGGAGTGGGCTGCATGATGCCGCGCATCTTCAGTCG
TCGATCACCGTGGTGCTGGCCAAACACGAGTTCTCCGCTGCCACGGTGGCCGACGGGTAC
AGCCGCAGCGGGGCCGGGTTCGGGGTCGCGGCGGCGGCCTCCGGTGGCGGCACTTTCCTC
GGTCAGAAATGCGCCGCAGCAACGGCAAGCTGA
>AA QryID: NC_000962_(+)_1113861_1114112 HitID: NC_018143_(+)_1113865_1114116
rank=1 bitscore=164.0 evalue=1.0E-38 perc_ident=100.0 perc_conserv=100.0 perc_gaps=0.0
query_cvg=100.0 subj_cvg=100.0 alpha=73.73 beta=17 prob=0.9822523006405806
ATGAGCACGAAATACTACCTGCAGAAGGTCCCTGTCGAAGCCGTCCAGCCGGGCTTTTCG
CTGGCCATTCCACACGATGGCGACTATCGCCTTTTCCAGGTCGACTGCACGCAAATGTGC
CAGCGAAGTGGCCAGCCGGTGATGATCAGACTCATGTCGGAGTCCGTCGATGGTGGCCAG
CCGTGGGTCTTGGAATATGAAGCGGGCACGGCGGTAATCCGGCTTCTCGGTGTTTGCCAG
GCCGCTTCGTAG
>AA QryID: NC_000962_(-)_31824_31967 HitID: NC_012943_(-)_31926_32069

rank=1 bitscore=74.7 evalue=1.0E-11 perc_ident=100.0 perc_conserv=100.0 perc_gaps=0.0
query_cvg=100.0 subj_cvg=100.0 alpha=75.9 beta=16 prob=0.9821749032040168
ATGGCTGATTCTGCGCTGCAGCAGCAACTCGACGAGGTGCGGGCTCTGCTCACTCGAGCG
CGAGAATTGTTCGGCCCCAATCCGATCGAGCCGCCCACCGACATCGCACCCGATCCCGAC
AGCACCAAGACCTGGCTCATCTAG
>AA QryID: NC_000962_(-)_789524_789799 HitID: NC_002755_(-)_791498_791773
rank=1 bitscore=101.0 evalue=2.0E-19 perc_ident=82.42 perc_conserv=82.42 perc_gaps=0.0
query_cvg=100.0 subj_cvg=100.0 alpha=82.42 beta=1 prob=0.9821428103536816
ATGGCTGCGTGGCGCGACACAAGCTGGCTGACCACGATCAGCCGCCGTCATGTCCCCATG
CCGGTCACCGAGCTCCCACGAACAACCGAGCTCCCGACCGGCATATTCGCCGGCTCCGGT
TGTCCCACCCTGGTTGCTGCTAGGCCGTCGCAGGCCTGGTCGCCACGCGCGAGGCGATCA
CCGCAGCCAGCGGCGCCGCGGGACAAACAGGTCAATCCGAGCACGGAGCCATCCTCGATG
CTACCGACAACCGAAGCGCGGCATTCGCGGTCTTGA
>AA QryID: NC_000962_(+)_3429568_3429750 HitID: NC_020245_(+)_3353350_3353532
rank=1 bitscore=68.9 evalue=8.0E-10 perc_ident=76.67 perc_conserv=78.33 perc_gaps=0.0
query_cvg=100.0 subj_cvg=100.0 alpha=78.33 beta=5 prob=0.9821351960398654
GTGGGTCCAACCCGCGATTCGCGGCAGCCGGCGGAGAGGGCGACCAAGAGACGTTATCGG
TTCGCTCGGGGACTCATGGCCGGTCTGCTGGGCACGATGGCTCTCACGAGCGGCGGTGGT
GTCGCTCGCGAGGATCCATTGGAACCTGATCCGCTAGCCCCGATCATCGACGATTCCAGG
TAA
>AA QryID: NC_000962_(+)_2968947_2969078 HitID: NC_002755_(+)_2965091_2965222
rank=1 bitscore=48.9 evalue=4.0E-4 perc_ident=100.0 perc_conserv=100.0 perc_gaps=0.0
query_cvg=100.0 subj_cvg=100.0 alpha=74.58 beta=15 prob=0.982108647464115
GTGCCGTTTACCACGCGTGATGTCCAGGCATCCCTGATGCGAATCCCGGCCCCAGCGTCC
AACACCACCGGGCTGACCGAGACCAGTCGCCTGACGGTCGACAAGGTGACAACATCCCCC
GCACCAGCCTGA
>AA QryID: NC_000962_(-)_2364797_2365093 HitID: NC_018143_(-)_2364805_2365101
rank=1 bitscore=107.0 evalue=3.0E-21 perc_ident=77.55 perc_conserv=77.55 perc_gaps=0.0
query_cvg=100.0 subj_cvg=100.0 alpha=77.55 beta=11 prob=0.9817825394954935
GTGTCGACTGCTCGAGGTCGCCGAGTCGGGCTACCGCGAATGACGTTCACGACCGTGGTG
CTCAACCCACCCCTGGCCACCCTGGAGGTGGCCGTGCTCGACGCCGACCGGCTACGGCGC
GCGTTTCGGCGCATCGCGGGTGCCGCGTTGGGAAAACGGCTGCGGGAACTGGATCGCAAA
GATGCCAAGGGACACAAAGGGGTTCCACGGGCGCCAAAGACTCCCAGTCCGACGGCGAAT
CGCCGGATTAGTGACGGGCCCGCGCGCCGATTGACGAGATGCTGTCGACAATCTTGA
>AA QryID: NC_000962_(+)_1192235_1192375 HitID: NC_018143_(+)_1192240_1192380
rank=1 bitscore=85.5 evalue=6.0E-15 perc_ident=100.0 perc_conserv=100.0 perc_gaps=0.0
query_cvg=100.0 subj_cvg=100.0 alpha=80.71 beta=3 prob=0.9815179289176543
ATGCAGAGTGTTCGACAAATCTTTCCCCAAGACATTTTTATTGGTCGCACGATGGGCGTC
GTCGTCGAGCGGTATGGCAGCACCGATTTGTCTTCCAGGGGAATGTTCGTACCGTTTCAT
GACGTCGACTGTGTCCAATAG
>AA QryID: NC_000962_(+)_1757438_1757671 HitID: NC_018143_(+)_1757444_1757677
rank=1 bitscore=132.0 evalue=5.0E-29 perc_ident=100.0 perc_conserv=100.0 perc_gaps=0.0
query_cvg=100.0 subj_cvg=100.0 alpha=83.53 beta=3 prob=0.9814858336342136
ATGGTCGCGACCACTACGCACTTCCCGAAGCAAAAAGCGCCCTGCGGGCACATGGTTGAC
GGCGATCACCACATCGAGCGCGACGACGAAGGCCTTGCCTACGACGACCTCAAGTTTTCC
TGCGGCTGCCGCGAAATCCGGCATTTCTACCACGACGGATCCATGCGGGTACGCACGATT
CGACACGACGGCAAGGTGTTGAAGGACGAGCACAGCGGCGATCACGAAGCGTGA
>AA QryID: NC_000962_(-)_2582675_2582830 HitID: NC_002755_(-)_2578521_2578676
rank=1 bitscore=63.9 evalue=2.0E-8 perc_ident=78.43 perc_conserv=78.43 perc_gaps=0.0
query_cvg=100.0 subj_cvg=100.0 alpha=78.43 beta=1 prob=0.9814086494137797
GTGACCGCCGTTCCTGCTACGGCAAACATCGGGATCTTGGTGTCGACTTCTCGGCGTTCT
TCGGTTGTTAGATGCCATCGGGCTAATGGTTACTGTGCGGTCAATGGAAAGGATTTTGCG
GTCGGCCCTGGGACTGCCAAAGGGATTGGGACATAA
>AA QryID: NC_000962_(+)_3547037_3547330 HitID: NC_017026_(+)_3541128_3541421
rank=1 bitscore=165.0 evalue=9.0E-39 perc_ident=100.0 perc_conserv=100.0 perc_gaps=0.0
query_cvg=100.0 subj_cvg=100.0 alpha=75.61 beta=15 prob=0.981392010458906
GTGACGGCGGCCGAGGACAAAGAAGAGCGGCTTCGGCTGTCCGGAACCCGGATCGAACTC
GAGGAGCTACTTCAGCTTCCGGTCGATGTTGCGTACGAGGGCCTGTTGACGGACGACGTT
TCCGAATCCGTTCGCAAAAAGCTCATTACGCTACGAGCCGGTCCCTCAAGAACCGCCTGC
TCGAATCTGCGCAACCCCGCTGGCGTTGGGGCGGACGACGGTGCTCGGCGTGATGTGGTG
CACCAAAGGGACATTGCCGACGGAACTGGCGTTGAGCCAGCAACACACCGTTGA
>AA QryID: NC_000962_(-)_934293_934481 HitID: NC_020245_(-)_935120_935308

rank=1 bitscore=81.3 evalue=2.0E-13 perc_ident=79.03 perc_conserv=79.03 perc_gaps=0.0
query_cvg=100.0 subj_cvg=100.0 alpha=79.03 beta=2 prob=0.9813489540784104
ATGATCGGCATCAGCTACTTCGTTATGACCCAACTCGAGGCGGCGGTCGAGCGACGCTCA
AGGCGATCTTCCCGTTGTGGCGACCGCCGACCTCTCGACGGCGCCAACCACCATGGCTTG
TTGGGTTCGTCGTTCCTCGCGCCTGCTCGGCCTGACCTCCAAGCGCAGCGACAAGCTTTG
GCGCAGTAG
>AA QryID: NC_000962_(+)_2232399_2232671 HitID: NC_017026_(+)_2227689_2227961
rank=1 bitscore=121.0 evalue=1.0E-25 perc_ident=80.0 perc_conserv=80.0 perc_gaps=0.0
query_cvg=100.0 subj_cvg=100.0 alpha=80.0 beta=5 prob=0.9811714578418584
ATGCTCGACGATCCGCGTTCCGCGGGTAGCGTCGTTGCGCCGTACGACGATGGCGAGCTG
CTGCGTCTCGCCGAACTACGCGCTAGCAGCGGGCTAAAACTACCTGATTGCTGCGTGCCG
GATGTGGCAATTCATCACCAGGCAAGCCTCGCAACCTTTGACGACACGCTCGCTGCCGCA
GCACGCACAAGGAGCGTGCCCGCTAGCACAAACGGCGCAGCTAACCCAATACGACCAGCT
TCACTTGACATAATGTCGCTTATCGGCTTATAA
>AA QryID: NC_000962_(+)_3580277_3580495 HitID: NC_012207_(+)_3525409_3525627
rank=1 bitscore=87.4 evalue=2.0E-15 perc_ident=100.0 perc_conserv=100.0 perc_gaps=0.0
query_cvg=100.0 subj_cvg=100.0 alpha=73.28 beta=18 prob=0.9807474435740465
GTGGCTACCATGGCGGCGGTCGTAGGCGGCGGTCCACAGGACGAAATACCCGAAGCCGAT
GCGGTGGAGCAAGGGCGTGCTGTCGATTTCGACGACGAAGCCGGGTTGGACACCGCCTAC
CTCAGCGGCGGCGCCGGCGACCGAGACGCCAGCGAAGCCGACGTCGTCGACCAAGCCTTC
GTCGTTCCGGTCGCCGACGACGAAGAAATCGACCGGTAG
>AA QryID: NC_000962_(-)_33900_34157 HitID: NC_002755_(-)_33844_34101
rank=1 bitscore=123.0 evalue=3.0E-26 perc_ident=100.0 perc_conserv=100.0 perc_gaps=0.0
query_cvg=100.0 subj_cvg=100.0 alpha=82.51 beta=1 prob=0.980713968054949
TTGGTGGACCTGAAAGCGCATCCACCACCGAAGGTGGCGGTGGGAACAGAGTCACGGGGT
GCAAAGTCGAATCGACTCAGCGCTGCCGCCGATCGGCACTGCGACAACGCGCCAACACGG
TCACCCAATACCGCACCTTTCGACGTTATCGCCGGCCCACCAGCACCGGCACCACAATGC
ACCAAACGCAACATCGCACGCGCATTGGGTCGCATAGCGCCGCTAAGAGTAGGACGTGCC
GCCGTGAATTATCAATAG
>AA QryID: NC_000962_(+)_132875_132997 HitID: NC_020245_(+)_132952_133074
rank=1 bitscore=62.4 evalue=4.0E-8 perc_ident=100.0 perc_conserv=100.0 perc_gaps=0.0
query_cvg=100.0 subj_cvg=100.0 alpha=78.75 beta=1 prob=0.9807111780953393
GTGTTGCATCCCGCCCAACGGCGCAGGCAACAATGGTGCGATGAGTGGCGCCAGCTCATC
GGAGTCGCCCACCTGCTATCGCCATCCCGGGCGCCGGACCTACGTCCGCTGCACCCGATG
TGA
>AA QryID: NC_000962_(+)_1622756_1623007 HitID: NC_002755_(+)_1622638_1622889
rank=1 bitscore=162.0 evalue=4.0E-38 perc_ident=100.0 perc_conserv=100.0 perc_gaps=0.0
query_cvg=100.0 subj_cvg=100.0 alpha=82.35 beta=6 prob=0.9806185709906448
TTGAGTTCTGCGATGCAGAGGTGGCAGATCGTCAATGCGGGCGAGAATTTGTTCCGGCCT
CTGTTGATGCGGGTGACATCGGAAGGTGTGGGTAAAGGGATCAGCCCGAGATCATGCAAT
CACTGTCCTGACAACCAGATTCAGCACGGCCTGGTAATCGACAGGATTCTGGGACTATCA
GACTCCAGCATCACGGTTCTCACCCGGGCCCAGGTCGAGGCGATGGTCGCGGCGCTGCCG
CGAAGCTACTGA
>AA QryID: NC_000962_(+)_2736988_2737116 HitID: NC_002755_(+)_2733838_2733966
rank=1 bitscore=76.6 evalue=2.0E-12 perc_ident=100.0 perc_conserv=100.0 perc_gaps=0.0
query_cvg=100.0 subj_cvg=100.0 alpha=79.46 beta=1 prob=0.9806031384694648
TTGGCCGCCGAGCAGTCCCATGACGATCCGCTAGTTCACCTCGGATACCCGCTCGGCCGC
AATGCGCAGCTAGCGGCCATGTTGATCGAAATCATTTGGGGTACACCGCATCTCGGAGCA
ATATGGTAG
>AA QryID: NC_000962_(-)_800104_800394 HitID: NC_018078_(-)_800947_801237
rank=1 bitscore=167.0 evalue=1.0E-39 perc_ident=100.0 perc_conserv=100.0 perc_gaps=0.0
query_cvg=100.0 subj_cvg=100.0 alpha=81.48 beta=7 prob=0.980428429840238
TTGCGCCGAATACTGCGCCGCTGTTTACCTGTCGTAGGTCCACCGGCCCCGCGCTCGGGC
GTGTCGCCCGCACACAGCCTTGTCGCGATCAATTTCAAGGCCGAAATCGTCGCGCTTCAA
GGGTGGGACCGGACGCGCCCGTGTGGGCGCTGGTCGTATGCCCGGCCAGACGCAAACCTG
GCGCAGGGCAACCTGAACAGTATGCCCCAGATCACCGCTTCGGCCAAATCGCCGCTGGCT
CGGGTGCAGCGGCGGGCGGCCGGTTGCTATCGGCACCTGACCCGGCACTGA
>AA QryID: NC_000962_(+)_2829954_2830139 HitID: NC_009525_(+)_2841922_2842107
rank=1 bitscore=117.0 evalue=3.0E-24 perc_ident=100.0 perc_conserv=100.0 perc_gaps=0.0
query_cvg=100.0 subj_cvg=100.0 alpha=80.19 beta=4 prob=0.9802612145513232
ATGCTGGCTGCGTTTAGGTCTCACGATGCCGTGTTAAGAGAGTTCGAAAAGCTCGGCCGC
TATCATCAGTCAACCGGGCACGGCTGCCTCTGCGGCAAACGAAACTGTGCAACGCTGTCC

ATCATCGATAGCAACCAGATATATGGCCACATTGACCGAATGAATCGCCGCGACGAGCTT
GGCTAA
>AA QryID: NC_000962_(-)_1526236_1526376 HitID: NC_017026_(-)_1527162_1527302
rank=1 bitscore=82.8 evalue=5.0E-14 perc_ident=100.0 perc_conserv=100.0 perc_gaps=0.0
query_cvg=100.0 subj_cvg=100.0 alpha=77.54 beta=5 prob=0.9801846944726926
GTGACGGACTTTTACGCATTATCGCTGACGATCTTTGCCTCCCAGGACTCCAGAATCTAC
TGCGACGGTTCGGGTCAAGACGACTTCGGTGGTGTGCTTCGCGTTGCCACCGCGGTCGGC
GAGGTGCCCACCGGGCTGTGA
>AA QryID: NC_000962_(+)_68418_68579 HitID: NC_020245_(+)_68410_68571
rank=1 bitscore=47.4 evalue=0.002 perc_ident=71.7 perc_conserv=71.7 perc_gaps=0.0
query_cvg=100.0 subj_cvg=100.0 alpha=71.7 beta=9 prob=0.9801743480809474
GTGATCATGGAACTTTCCGTGTCTGTTATCGCGGGGTTGGTCATCGCACTGCTGGCGGCC
ATCACCCCTGCTGCGGGCGAACGCCCGGAAAGCCGCCGCCAGGCGCTCGCAAATGCCGCC
GAGGCCGGGGAGCATCCGGCCACATCACCGTTGCGACGGTAG
>AA QryID: NC_000962_(+)_2368732_2368860 HitID: NC_002755_(+)_2369640_2369768
rank=1 bitscore=67.4 evalue=1.0E-9 perc_ident=100.0 perc_conserv=100.0 perc_gaps=0.0
query_cvg=100.0 subj_cvg=100.0 alpha=77.61 beta=1 prob=0.9797469193943291
TTGATTACGTGGATCACCATGTGGGTGATCGCGGTCGACAGCTTTGGTACCGAGCACATC
GCCACAACGCGCGGTACGAATCTAGTACACAAATCCGCACCAGCCGCCATGCGACTTCGC
AGGTCATAG
>AA QryID: NC_000962_(+)_4316824_4316928 HitID: NC_002755_(+)_4309147_4309251
rank=1 bitscore=61.2 evalue=9.0E-8 perc_ident=100.0 perc_conserv=100.0 perc_gaps=0.0
query_cvg=100.0 subj_cvg=100.0 alpha=77.2 beta=1 prob=0.9797290314963294
GTGGATAACAGCGCCCGTCAATGCGTTCTCATCGACAGCCTGGCAGGTACCCGAGCGAAA
TGGATTGTCGCACTAAGCATCCACATCTGCCCCGGCTGCACCTAG
>AA QryID: NC_000962_(+)_927620_927808 HitID: NC_017528_(+)_928280_928468
rank=1 bitscore=80.5 evalue=3.0E-13 perc_ident=74.19 perc_conserv=74.19 perc_gaps=0.0
query_cvg=100.0 subj_cvg=100.0 alpha=74.19 beta=6 prob=0.9795642261051574
TTGGACCACGTGATCGACGACCGTCACAAGTCGACACGCCGAACGTGCAACCACGGCGGC
ATCACCTGGCGTGTCGCCGCCACCAGCGCACGCTCGGCACGGAGTTTAGCAACTACTCAT
CCAGAAGCCGGCCACTACGGCCTGGCCACCTGGTTTACCCGCATGGACGCGATGACCGCA
CCGACCTGA
>AA QryID: NC_000962_(-)_2582827_2582991 HitID: NC_002755_(-)_2578673_2578837
rank=1 bitscore=73.2 evalue=3.0E-11 perc_ident=75.93 perc_conserv=75.93 perc_gaps=0.0
query_cvg=100.0 subj_cvg=100.0 alpha=75.93 beta=1 prob=0.9795523173986971
GTGCGGGAGATCACCGGTGTGCCAGTGTCGACCCTGCACGGCTGGGCTGCAAAGCGAGAG
CGCGGAATTGACGCACCGGGACCCCATTATGTGCGCCTCGGCGGGCGGGATCGGCGCTGG
ACGCGGCGCGACATGTACGACTGGCTCGAATCAGCCCGAGTGTGA
>AA QryID: NC_000962_(-)_1530973_1531209 HitID: NC_017026_(-)_1531901_1532137
rank=1 bitscore=136.0 evalue=3.0E-30 perc_ident=100.0 perc_conserv=100.0 perc_gaps=0.0
query_cvg=100.0 subj_cvg=100.0 alpha=76.64 beta=12 prob=0.9793413394848101
ATGATGGCTCTGATGAATGAGATTGATCTGTATGAGCACAAGACACCGCTGCCGATCTCG
CCGACGAGCGAAGGCACCCCGATGACCGAAACGCATTTCCGCCAGACGCTCTACGAGTGC
GCGGTTAAGTTGCGCGAGCTGGCCTACACCTTGCCGCAGGGAGTGGGCGAGCACGCTCTC
TTGCGAATGTCCGAGCAGATGATCGTAACCGCCGGTCAGGTCGCGCCGAGAGTCTGA
>AA QryID: NC_000962_(-)_4093021_4093251 HitID: NC_002755_(-)_4085263_4085493
rank=1 bitscore=118.0 evalue=1.0E-24 perc_ident=100.0 perc_conserv=100.0 perc_gaps=0.0
query_cvg=100.0 subj_cvg=100.0 alpha=77.17 beta=8 prob=0.979279652824607З
TTGCTTACGTTGGACATTGCGACCAGGGTGTGGTCGACGTCCCGGGGAACCCGACCTCCT
AGCAGGCCCGGGGCCTTGCGGCGACTGTCTGAACGGGCAATCGTCACCACCCTGCTGATG
CCGGCGCCACTGGATGTCGACGACGAGATCGGCGGGGTCGTCCGACGGGCGTTGCCGATT
CCCTACGTCGGCGGCTCGCGCATTCAGCCCAGTTCAGTTCAGAGCGCATAG
>AA QryID: NC_000962_(-)_2619023_2619193 HitID: NC_020245_(-)_2565766_2565936
rank=1 bitscore=102.0 evalue=7.0E-20 perc_ident=100.0 perc_conserv=100.0 perc_gaps=0.0
query_cvg=100.0 subj_cvg=100.0 alpha=78.53 beta=2 prob=0.9791721226413832
GTGTCCGACGAGAGTCGGGGCGGGTTCGGCTACGTCGACGTATGGGCCCGAACAGCTCTG
TGCGCCGACACCAAGCTGGTGCCGAGCTGGCCGCGGTTGGTGAGTGCATGCCACTCGCCT
GATGATGCCGGGACTCCGCCGACCTCGACACTTCAGTGTGAGGCGCAGTAG
>AA QryID: NC_000962_(-)_2152953_2153183 HitID: NC_012943_(+)_2269677_2269907
rank=1 bitscore=126.0 evalue=4.0E-27 perc_ident=100.0 perc_conserv=100.0 perc_gaps=0.0
query_cvg=100.0 subj_cvg=100.0 alpha=78.66 beta=5 prob=0.9790961848812957
GTGACAGCGGTGGTTGCTCGACACGGAGGTGGCCAACGGCTTCCGGACATCCTCCGATAC

ATGCGGTCTGGGCGGTGTGCTGGCGGACAAGGCTGTGACTCGGTGGAAGCGTTGGCGCCG
TCCGCGATTTACCGCATCATTGCCGGACACTGCGTCGGTGGCGCGCCGTCCGTGCGCTCG
CAGGCGCATATGGGGATCGAACAAGCCCATCGGCGGCGGGAGAACGAGTGA
>AA QryID: NC_000962_(-)_2582315_2582440 HitID: NC_020245_(-)_2529056_2529181
rank=1 bitscore=68.6 evalue=4.0E-10 perc_ident=100.0 perc_conserv=100.0 perc_gaps=0.0
query_cvg=100.0 subj_cvg=100.0 alpha=76.21 beta=2 prob=0.9789905882893022
GTGAGTAACGGATTCGTACCACGGATTGTTGCGGTACTGACGCTGACCCTGGTTCTCGGC
TACTGCGGACTGCGCTTCGGCGAGGCAGCGGCCCTGCGTCGCAAGAACGTGGGGACCGGG
AGTTGA
>AA QryID: NC_000962_(-)_1394008_1394160 HitID: NC_002755_(-)_1393495_1393647
rank=1 bitscore=79.3 evalue=4.0E-13 perc_ident=100.0 perc_conserv=100.0 perc_gaps=0.0
query_cvg=100.0 subj_cvg=100.0 alpha=77.0 beta=1 prob=0.9787780788507645
TTGCTAGCGGTGGCATTGGGTCACGGGCGTCACGGCATGCACCAGGGCAACCCGTATCGT
CACCAATGTCCACGCCGCATTATTCCGCTTCGAGCGGTGAACCGCGCCGCTACCGAAACA
CATGCTCGGATCGTTGAACCGGCATTAGCCTGA
>AA QryID: NC_000962_(+)_2632280_2632393 HitID: NC_002755_(+)_2628125_2628238
rank=1 bitscore=65.9 evalue=3.0E-9 perc_ident=100.0 perc_conserv=100.0 perc_gaps=0.0
query_cvg=100.0 subj_cvg=100.0 alpha=76.09 beta=1 prob=0.9787195462252731
TTGTCGCTGGGTATCGGGCACTCGCCGACAGTAAAAAATTATTCGAATCCCGCATTCCTG
ACAAAACTTGATATGACCGATCTCACCGGCCGGCTTCGGCGCTTAAGTCACTAG
>AA QryID: NC_000962_(-)_4008464_4008634 HitID: NC_002755_(-)_4000604_4000774
rank=1 bitscore=89.7 evalue=4.0E-16 perc_ident=100.0 perc_conserv=100.0 perc_gaps=0.0
query_cvg=100.0 subj_cvg=100.0 alpha=77.35 beta=1 prob=0.978560037629802
GTGAGCCACGTCCGGTATGTCGCGTGGTTGGGGCAGCTGTCCTCGCGGATGGCGAATATG
CAGACGCTTACGTCAAGGAGCAGCATTGTCGCCGATCAGTTCGGCGAGGGCTTCCTTGTC
GTCCAAGTCGACACCGGCCTGCAAGCCTCCGGTGCCGTGGGTCGGCAATAG
>AA QryID: NC_000962_(-)_924393_924563 HitID: NC_002755_(-)_924302_924472
rank=1 bitscore=84.7 evalue=1.0E-14 perc_ident=100.0 perc_conserv=100.0 perc_gaps=0.0
query_cvg=100.0 subj_cvg=100.0 alpha=77.02 beta=1 prob=0.9785451429574248
ATGGGTTTGGCGCCTGTGATGCTGGAAATGGCTGAACAATCAGGTCTTTCACGAGCGATC
GAGGAGCAGATGGACCCGCCGTTCGACCCGGGTGGAATCCGCAGCGATCAACCCGGCCGG
CAATCTCGATCATCGCCGCGATGGCCTACCGGGGCGGGACGGCATCGGTGA
>AA QryID: NC_000962_(+)_230481_230630 HitID: NC_002755_(+)_230593_230742
rank=1 bitscore=79.3 evalue=4.0E-13 perc_ident=100.0 perc_conserv=100.0 perc_gaps=0.0
query_cvg=100.0 subj_cvg=100.0 alpha=76.63 beta=1 prob=0.9785040743572198
ATGGGTGGCGAGTTGACCGGGCGCCGGATCCCGACGGTTGTGGTTGATCTGCCGAATCAA
CGGCTTCTGGCCACGAACATGTGTCCGCGACTGGCGTCTGCGATACCAACCCAATCGGTT
ACTATAGAAACTGTTCCCGCCGACAACTAA
>AA QryID: NC_000962_(+)_3798935_3799063 HitID: NC_002755_(+)_3789810_3789938
rank=1 bitscore=71.6 evalue=5.0E-11 perc_ident=100.0 perc_conserv=100.0 perc_gaps=0.0
query_cvg=100.0 subj_cvg=100.0 alpha=75.45 beta=2 prob=0.9782905225686925
TTGGGCGGCGAATGCTACGCAATGGTGGTGCGCTTCCCAAGGGAGCACAACGCGAAGCTA
AAGCAGTTGCACGCCGAGACCGAGCCGAAAGGTCGCCCTGCGGGGAAGGCGGCCACGGGA
GAATTGTGA
>AA QryID: NC_000962_(+)_1163937_1164185 HitID: NC_017026_(+)_1164650_1164898
rank=1 bitscore=153.0 evalue=4.0E-35 perc_ident=100.0 perc_conserv=100.0 perc_gaps=0.0
query_cvg=100.0 subj_cvg=100.0 alpha=79.57 beta=4 prob=0.9782901925956446
GTGCCCGATTCCTGGCCGGTCGTTTGCGTTGACGACTGGTCTGTTGCCGGCCTGGAGACC
CAGGGGCAACACCCGCACGATTGGCTCAAACATTCTTCGCAGAAGCGGACGTGGCTCTTC
AAGCCGGCGCGACCGGAGCGCGATCGTTTACTCGGCGAAGACGTGGCAGAAAAGCTCGCC
AGCGAGTTGGCGCGGCTACGCGATGTCTCCACAACAAGAGGGGAAGCTCACCCGTCGTGC
AAATGCTGA
>AA QryID: NC_000962_(-)_704475_704672 HitID: NC_019965_(-)_702792_702989
rank=1 bitscore=127.0 evalue=2.0E-27 perc_ident=100.0 perc_conserv=100.0 perc_gaps=0.0
query_cvg=100.0 subj_cvg=100.0 alpha=76.58 beta=7 prob=0.9781302659359112
GTGACCGACGAGAAGTGCGTGAGATGCGGGGGCGACCAGCTCGTCGAAGGGGCCGTCGTC
TGGAACGCGCCGCTGCGTTTCAAGCGCGAAGGTGCCGGCCACTTCAATCGCGGCACCCAG
GTCAACGCCGTTGCCTGCGAGACTTGTGGGCATATCGACCTCTATCTCGAAAGCCGTGCC
CGCGGCTCGACGAAGTGA
>AA QryID: NC_000962_(+)_11225_11416 HitID: NC_017528_(+)_11230_11421
rank=1 bitscore=92.0 evalue=7.0E-17 perc_ident=74.6 perc_conserv=74.6 perc_gaps=0.0
query_cvg=100.0 subj_cvg=100.0 alpha=74.6 beta=2 prob=0.9781004193800039

TTGATCACAATCGCGAGTGAAGAGGGCGTTGATGGCCACTCCGACGGCCTCGACACCCGA
CCCGTACAGGTGGCGGTAGCGGTCCAAGGTCAACCCGGCGGAGTCGTGTTCGAGCATGTT
CTGAAGTGCCTTGAATTCGCCCCGGCCTGGATCGCCAACGACGCCGGGTGTGCCGAGCTC
ATGCAGTTTTGA
>AA QryID: NC_000962_(-)_1928837_1928998 HitID: NC_018143_(-)_1928844_1929005
rank=1 bitscore=99.8 evalue=4.0E-19 perc_ident=100.0 perc_conserv=100.0 perc_gaps=0.0
query_cvg=100.0 subj_cvg=100.0 alpha=76.93 beta=2 prob=0.9780898644829619
GTGATCCAGCAGTTCAACGAGCGCAACGCGCCGGTTTTCCTCATCAGCCTCAAGCCCGCG
GCTTCGGGCTGGACCTCAAACCTATTCGCCAGCGTCGTCGACGACGGCAACGCCTTCGGC
TCCGTACCCACCGTCGACGACATCCGCGGACTACTCGCTTGA
>AA QryID: NC_000962_(-)_3457359_3457466 HitID: NC_002755_(-)_3453150_3453257
rank=1 bitscore=44.3 evalue=0.009 perc_ident=100.0 perc_conserv=100.0 perc_gaps=0.0
query_cvg=100.0 subj_cvg=100.0 alpha=73.4 beta=1 prob=0.9777101186609026
GTGCAAGAGGAGATCGACGAGCGCCGCCGCATGAACGGGCAACTCAAACACACTCAACGG
CAACGGATTACCGGTCGCTGCGCCGGAGGGCCCGATCCCGCTCGATGA
>AA QryID: NC_000962_(+)_1680727_1680990 HitID: NC_002755_(+)_1680883_1681146
rank=1 bitscore=156.0 evalue=3.0E-36 perc_ident=100.0 perc_conserv=100.0 perc_gaps=0.0
query_cvg=100.0 subj_cvg=100.0 alpha=78.3 beta=5 prob=0.9775803843349018
TTGTCGGATCGGGAGCCTTGTGACCGGTTGGCCCTTGATCTGCGTTGGGAGGCCGCGGCG
GGGTTGACGGTGCACGCGCCGTCGTTGCATCCCACGGTGTTGGTCGGGATGCGTAACCGG
CTGCGGGCTTCGGATCCGACGTGTTGGTGGATGCACCATTTTCAAATGCCGTCGCGGGTC
GAAACTTGGGTGCCGTCGAAGAATAACCCCACCAAAGGCCCTACATCAGCGCCGTCCTAC
GTTCGTGTGTCGGACAATCCTTAG
>AA QryID: NC_000962_(-)_3359320_3359538 HitID: NC_009525_(-)_3371489_3371707
rank=1 bitscore=93.2 evalue=4.0E-17 perc_ident=68.06 perc_conserv=68.06 perc_gaps=0.0
query_cvg=100.0 subj_cvg=100.0 alpha=72.22 beta=5 prob=0.9775565445349342
GTGTGCCACGGGGCAGGTCCGCCCCGTTGTGTCACCGGCGTCGCTGCCTTCATCGGGGCT
TCATCTGGCGCCGTTAGCGTCGCCTTCGTAACCACGACGACGACAAGAAGGGATGAAGCG
ATGAGGGTGCTCGTCACCAAACCCGATGGTACTCAGGTGGAAGTGCACCTGGATCAGGGC
TTTCGCTTCCTGGGCACGGAGACCGTGGATAACGACTGA
>AA QryID: NC_000962_(+)_3965423_3965620 HitID: NC_018143_(+)_3965602_3965799
rank=1 bitscore=100.0 evalue=2.0E-19 perc_ident=100.0 perc_conserv=100.0 perc_gaps=0.0
query_cvg=100.0 subj_cvg=100.0 alpha=75.18 beta=4 prob=0.9773922077132042
ATGGCTTTTCGGGAGGTCAGTATGAACAAGATCAGGGAAGTGCTGCGGGTCTGGCTGGGG
GTGGCCGGGTTGCCGGCCCCGGGGTGCCGCACGATCGCCGCGCATTGCGGTATGGACCGC
AAGACGGTGCGGCGCTACGTGGAGGCCCGCGCAGGCACCCGGTCTGCCCCGCGACGACGA
TGTCAGCGCTATCGATGA
>AA QryID: NC_000962_(-)_3494317_3494442 HitID: NC_017026_(-)_3488376_3488501
rank=1 bitscore=63.2 evalue=2.0E-8 perc_ident=100.0 perc_conserv=100.0 perc_gaps=0.0
query_cvg=100.0 subj_cvg=100.0 alpha=72.88 beta=4 prob=0.9773780336245029
ATGGGAAGTGGGTTGTCGATGAGATCTCGCGCGGAGGTCACCAGCAGGTACGCCAAGGCG
TATGTGCAGGCTTTGAAGAAGAGCCGGGGCCGGATTTTCGACCAGGTGGTTGACCTGACG
GGCTAG
>AA QryID: NC_000962_(+)_4056464_4056676 HitID: NC_002755_(+)_4048705_4048917
rank=1 bitscore=119.0 evalue=4.0E-25 perc_ident=100.0 perc_conserv=100.0 perc_gaps=0.0
query_cvg=100.0 subj_cvg=100.0 alpha=77.49 beta=1 prob=0.9773055093646156
TTGGCCGATGTCAACAGTTTTTTTCATGCTAAGCAGATCGTCAGTTTTGAGTTCGTGAAG
ACGGCATGTTCACTTGTTGTCGACTACATCGTCTGCGCACATTTGCCCTCCTGCAACTGC
GCTGCGACAATGCGCCAACCGCCGTGTAGGCGGCGCGATCCCAAGGCAGTGTCTCCGACG
TCGATGCCTGCGCTTCGCCTTCGATCGGTATGA
>AA QryID: NC_000962_(+)_1992739_1992972 HitID: NC_009525_(+)_2002424_2002657
rank=1 bitscore=124.0 evalue=2.0E-26 perc_ident=100.0 perc_conserv=100.0 perc_gaps=0.0
query_cvg=100.0 subj_cvg=100.0 alpha=76.11 beta=5 prob=0.9772895056342765
GTGGTCACACGCTGCGTGCTTCACATTGGTAGCTTGGGCACGTCGGCAACCGTCACAGCT
GTCACACGGGTCCCTGTGGGGTTGGTCGGCCACCGGCGACAACGTTTCCTGCGCGCCTTG
ATCTGTCGCCGCTGGGCAGGCATCGCCGCGACGGCCGTATCAGGCTTGGTCGGTGTGAGC
CGCCAAATCGGTATTGACGAATTCGTCATCGAACTCCCGGCCAAGACCACTTAG
>AA QryID: NC_000962_(-)_4376074_4376238 HitID: NC_020245_(-)_4268927_4269091
rank=1 bitscore=102.0 evalue=6.0E-20 perc_ident=100.0 perc_conserv=100.0 perc_gaps=0.0
query_cvg=100.0 subj_cvg=100.0 alpha=76.12 beta=1 prob=0.9770690867438105
ATGGCTACTTTCTGCCTGACCATGTCGCTGTACGGGTTCAGTGACACCCGCGCGTATCCT
CCAGGCGTCGCAGATTTCACATCCGCCCCACACGTACCGCTGACATGCTGGAACACATGG

GGCGGCGTGGACCAGTTCGACGTCGGCATCGGTGCAGACGACTGA
>AA QryID: NC_000962_(-)_1933963_1934202 HitID: NC_018143_(-)_1933970_1934209
rank=1 bitscore=142.0 evalue=5.0E-32 perc_ident=100.0 perc_conserv=100.0 perc_gaps=0.0
query_cvg=100.0 subj_cvg=100.0 alpha=78.28 beta=1 prob=0.976843910411771
ATGTCCCCATGGTGTATCGATTACGTACCCTCTTACTTCGTCGGGGTCGCATCGGCATTC
GCCTTGCCCGCCTGTGCATTCACCACGATCGACCCGGTTGCTGACATGTATCGGGACGGT
GTGTCGCCCGAGCCAGGACGCCAGACGTTGCCAACAGTAACGATTATACCCCTATGGGGT
ATTAACACGCGCGTCACACGGCCGAAACCTTTTTCGCAGAAGATCTCTTATGGAGATTAA
>AA QryID: NC_000962_(+)_2334728_2334877 HitID: NC_017026_(+)_2328053_2328202
rank=1 bitscore=80.9 evalue=2.0E-13 perc_ident=100.0 perc_conserv=100.0 perc_gaps=0.0
query_cvg=100.0 subj_cvg=100.0 alpha=73.19 beta=4 prob=0.9767926501029405
ATGCGGGCGAACGGTGTGTTCGGCCTGTTGGCGGCCGCCGCGTGCGGTGTTCCCATCCCC
GTTATCGACAACCGCGCCGAGGAGATGACGGGCCGGCACGCCACAACGGCAACGAGTTTC
AGCATCACGGACCAGTCGTGCGCATCATGA
>AA QryID: NC_000962_(+)_177388_177513 HitID: NC_002755_(+)_177559_177684
rank=1 bitscore=69.7 evalue=2.0E-10 perc_ident=100.0 perc_conserv=100.0 perc_gaps=0.0
query_cvg=100.0 subj_cvg=100.0 alpha=72.32 beta=4 prob=0.9766634718610667
GTGCCGGCCACCATTGGCCTTCCCGATGGATTGGTGCCAGCAGCAACCGGCATCATCGAA
AACCGGCTCTTCATGATCGAGGGCCGGCAGCGGCTCGAGCAGCGGCAGGCCGGGGTGATC
ACGTAG
>AA QryID: NC_000962_(+)_1190529_1190651 HitID: NC_002755_(+)_1190575_1190697
rank=1 bitscore=78.6 evalue=5.0E-13 perc_ident=100.0 perc_conserv=100.0 perc_gaps=0.0
query_cvg=100.0 subj_cvg=100.0 alpha=73.99 beta=1 prob=0.9765679741285933
GTGAATTCTTTGAGCGGAATTGCTCGTGCGATCCGGCTACGTTTTCGAGGTGAGATCTGG
GTGGGCGGCGATGCCCCGTGCTTCGATGATCAATTTGGGGATCTGAAATGTCAAATGTGT
TGA
>AA QryID: NC_000962_(+)_3489169_3489309 HitID: NC_020245_(+)_3410176_3410316
rank=1 bitscore=81.3 evalue=1.0E-13 perc_ident=100.0 perc_conserv=100.0 perc_gaps=0.0
query_cvg=100.0 subj_cvg=100.0 alpha=72.85 beta=3 prob=0.9762244096885799
GTGCACCACATTCTGGCGATGTTGGTGAGGTTTGTTCCTGCGCCCGTCCGTGGCGCGTTC
GGGATCGTTGGGGTTGGCCGGTTGCCCACCTCGGCGGAAGCGGACGGTGAGCGCGGCCGA
GTCGTCGACATTTGGCGGTAG
>AA QryID: NC_000962_(+)_985992_986147 HitID: NC_002755_(+)_986004_986159
rank=1 bitscore=85.5 evalue=6.0E-15 perc_ident=100.0 perc_conserv=100.0 perc_gaps=0.0
query_cvg=100.0 subj_cvg=100.0 alpha=73.45 beta=2 prob=0.9761695943467141
GTGCTCGGCCAATCCGGGGCCCGGACAGGCTCACGGATATTGACTCCCGGCGCGATGGAA
AATCATCGCGGTGCCGTCATTCAATCGCCGGACACGTGGCCACCGCCCAGCGGTGTGGCC
AGCAAGCCGAATCTCAACCGCAGGTGTGTTCAATGA
>AA QryID: NC_000962_(+)_503146_503298 HitID: NC_017026_(+)_503468_503620
rank=1 bitscore=78.2 evalue=1.0E-12 perc_ident=100.0 perc_conserv=100.0 perc_gaps=0.0
query_cvg=100.0 subj_cvg=100.0 alpha=71.1 beta=6 prob=0.9760045485852692
GTGCGGGCGGCCCGCTACCGGTCCGATTGCCCCGGATGTGGCAGCTTGCGCATACAGTGC
AGCCTTATACACGCCGACCTGTTGGCTGCCGCCGACTACAACGTTGTGGGATTGGCGGCG
GCGGTGCTATCGGTGTGGGCCTACTTGGCGTAG
>AA QryID: NC_000962_(-)_1961887_1962129 HitID: NC_018078_(+)_2460398_2460640
rank=1 bitscore=139.0 evalue=4.0E-31 perc_ident=100.0 perc_conserv=100.0 perc_gaps=0.0
query_cvg=100.0 subj_cvg=100.0 alpha=72.76 beta=10 prob=0.9757723372271181
ATGCGAGACACGACTTTCGGCCCTGTCGTCACCCGCCTGTGCGGGTGGACGTATGCCCTT
TCCGTCGTCCTGCTGTGGGTGACCTGCACCGCCTATGCTGTCTTGATCGCGGTGAGCACG
ACACGAATCGTCATCTTCCGCAAAGAATTCGCCGACGACCTAGCTGATCCGCGCCGGGGA
TTTGGGATGTTCACCTTTGTCGCTGCGTCCGACGTGCTCGGCACGCGATTAGTTGGTCAG
TAG
>AA QryID: NC_000962_(-)_3310445_3310651 HitID: NC_017528_(-)_3304651_3304857
rank=1 bitscore=95.1 evalue=1.0E-17 perc_ident=100.0 perc_conserv=100.0 perc_gaps=0.0
query_cvg=100.0 subj_cvg=100.0 alpha=71.32 beta=7 prob=0.9757173521294413
GTGTCGCAGTTCAAAACGCTGAAGTACCGCAACGACTCTCCTGAACGGTTCGATTCGATC
GAACACGCCCGCACCTGGCCAGGCCACATGGGCGCGGCCATGGCCAACGCCTACTCGGCC
AACCCGAATCCATTCGGCGTCTCACCGCAACCCCCGAAACCGGCGACCGCGGCATGGATC
AACCCGCCCACCCCAGATCCGAAATAG
>AA QryID: NC_000962_(+)_3318044_3318286 HitID: NC_002755_(+)_3312364_3312606
rank=1 bitscore=88.2 evalue=1.0E-15 perc_ident=66.25 perc_conserv=66.25 perc_gaps=0.0
query_cvg=100.0 subj_cvg=100.0 alpha=66.25 beta=12 prob=0.9756692576254595

TTGTCGGCCACCGCCGGCAAACCAACATTCAGCCACAACGCGACAGAGAGGTACCCAATG
TTCACTGCCCGTATCCGCGCCCTCGCCGGCATGTCTCTGCTAGCCTCGGCGATCGGACTG
GCGGCCTTCGGAGCCGCTACCGGCACCGCCAATGCCGCCCCGACCCACCAACCCGAGTGG
GGCACCTACACCTGCTACGACTACGCAACCCAGACGTTCTACGAGTGCTTTGACCCCAGC
TAG
>AA QryID: NC_000962_(-)_3239515_3239706 HitID: NC_009525_(-)_3251507_3251698
rank=1 bitscore=117.0 evalue=2.0E-24 perc_ident=100.0 perc_conserv=100.0 perc_gaps=0.0
query_cvg=100.0 subj_cvg=100.0 alpha=74.21 beta=3 prob=0.9756270262970188
TTGCGGGGGACTGCGCCCGGCTTCGCCGCGCTTGCGATCATCACTGGTTCGATGGTGATG
ACCTGGGGGCACCTCCCGCTTGCGGGGGACTGCGCCCGGCTTCGCCGCGCTTGCGATCAT
CACTGGTTCGATGGTGATGACCCGCTGCGCCCGGCTTCGCCGCGCTTGCGATCATCACTG
GTTCGATGGTGA
>AA QryID: NC_000962_(-)_3755328_3755615 HitID: NC_018078_(-)_3752302_3752538
rank=1 bitscore=142.0 evalue=7.0E-32 perc_ident=98.72 perc_conserv=100.0 perc_gaps=0.0
query_cvg=82.11 subj_cvg=100.0 alpha=61.47 beta=7 prob=0.9754890768456959
ATGAGCCGACGGCGGCGGCGTGCCCGTCGTGCGGGGGTGGTCTCGATATCAGTGAAGGAA
TACGCGGTTACCTCACCGAAAGATCTACCTTATGGCGAAGACCGCATCATGGTGCGCTGG
AACAAAATTCGCTGGCGATGCCGAGAAGACTACTGCAAGCTGGGGCCGTTCACCGAGGCC
ATCACCCAGGTACCTGCCCGCGTCCGCAGCACGCTGCGGCTGCGTCGGCAGATGGCCAAG
GCGATCGGGGATGCGGCCCGCTCGGTGGGCCGAGGTCGCCCAGGCTGA
>AA QryID: NC_000962_(+)_1735608_1735775 HitID: NC_002755_(+)_1735761_1735928
rank=1 bitscore=101.0 evalue=8.0E-20 perc_ident=100.0 perc_conserv=100.0 perc_gaps=0.0
query_cvg=100.0 subj_cvg=100.0 alpha=72.99 beta=3 prob=0.9754708002966239
TTGAGCCCAGGTGGCTTCCGGCTTACCGGACACGTCGTGTGGTGCCGAAGCTCTGACGAG
AGGGGTGCGGATTTCCGGCAGTTGCCGGCATCTCTGTACTCCTGTGACGCGCTTTATCGT
GCGGACAACCGTACGTGTCGTGGCCGTGAGGAGGTGAGGGACGCATGA
>AA QryID: NC_000962_(-)_2718844_2719047 HitID: NC_017528_(-)_2718640_2718843
rank=1 bitscore=113.0 evalue=4.0E-23 perc_ident=98.51 perc_conserv=100.0 perc_gaps=0.0
query_cvg=100.0 subj_cvg=100.0 alpha=73.5 beta=3 prob=0.9753875249222875
ATGCCGCTCGCCGAGGGAGTCACCGGCGAGGGTCGCGACACCCAGTCCCGCCCCGTCGGC
GACGACCTCGACCTCACCCGGAACGAGACCAAGATGATCACGCAAAGGCTTAGGAATAAC
CAATCGTGCTGCCTTATCAATGGCTGCTCGCTTAGGCTGGCTGGCAGTTGCCGCGAACGG
AACTGCGGCAAGATAATCCAGTAA
>AA QryID: NC_000962_(+)_3964168_3964296 HitID: NC_020245_(+)_3872981_3873109
rank=1 bitscore=67.8 evalue=8.0E-10 perc_ident=100.0 perc_conserv=100.0 perc_gaps=0.0
query_cvg=100.0 subj_cvg=100.0 alpha=70.13 beta=4 prob=0.975213501978182
GTGCGCGAGTCGATCGAGGCGCACCAGAGCATCGTGTTCGCCGCCCTGGCGGTCAGAACT
GGATTGAACACCAAACAGGTTGGAGCATCAAGAAATTCGTTCAAACACTACGTCGCTACC
GCACCGTGA
>AA QryID: NC_000962_(-)_2792550_2792720 HitID: NC_002755_(-)_2788038_2788208
rank=1 bitscore=99.8 evalue=4.0E-19 perc_ident=100.0 perc_conserv=100.0 perc_gaps=0.0
query_cvg=100.0 subj_cvg=100.0 alpha=71.94 beta=4 prob=0.9751001632685012
TTGGCCGTTCCAACTCGGCTGCCCAACACGAGGTTTCGTCGGTCGTGGCCGCGGCATCGG
TGCTGCGGGTGGATCATGGCCCGCATCGGCTTTCACCACCGCGTCATCGCGACTAGCGTT
GTGTTGGCACTGTGGTGCGTGGCGGTGGCGGGTTTGAAGCAGGAGGTCTGA
>AA QryID: NC_000962_(-)_3500908_3501183 HitID: NC_002755_(-)_3496705_3496980
rank=1 bitscore=165.0 evalue=9.0E-39 perc_ident=100.0 perc_conserv=100.0 perc_gaps=0.0
query_cvg=100.0 subj_cvg=100.0 alpha=76.69 beta=1 prob=0.9749984937718551
GTGCGGCAACTCGTGCCGCGGCCAGTGTACAACTGGGCGGGCAGGTTGTATGGCAGTCGA
CTGACGGCGGATCGGAGATTGATCGACGCCGTGCCGGCCGCATCGAGATGCGCTCTCACA
GCGCGGCTATGCCCAAATATTCTGTGGGTCAAGCGATATGCAGCCGATGGACGGCCGCTG
GTTCGGCAGCTGTCGGCAACTGTAAGCCATTTCTGGGACTTTGCTGTGAAAAGCTGGGCG
ATGGTTGTGGACCTGGACGAGCCACCCGTGCGATAG
>AA QryID: NC_000962_(-)_1147980_1148156 HitID: NC_002755_(-)_1148024_1148200
rank=1 bitscore=91.3 evalue=2.0E-16 perc_ident=100.0 perc_conserv=100.0 perc_gaps=0.0
query_cvg=100.0 subj_cvg=100.0 alpha=72.47 beta=1 prob=0.974976284694834
ATGGCCGCCGGTCCATGGACGGCCAGGTGCAGGGTGAGCACGTATCGAAGCTCAATTCCA
TGCAGTTGTCTGGTCATGGCCGAACATTCCCATGCGGGTCTGACATCGGCCAAGGTTGTC
GCTACGAGGCGGGCAGAAGAGTGTCCCCCCGCGCAGATACGCGTGTGGCAGATGTGA
>AA QryID: NC_000962_(+)_1113634_1113864 HitID: NC_016804_(+)_1114402_1114632
rank=1 bitscore=132.0 evalue=5.0E-29 perc_ident=100.0 perc_conserv=100.0 perc_gaps=0.0
query_cvg=100.0 subj_cvg=100.0 alpha=73.3 beta=5 prob=0.9749705368526611

GTGGTTGAGACGGCGAGCACAGCCCTCGGGGCAATCCTGGCAGGTCGCAATGCGGTGGTG
CCGCCACGGTGTCCACGTCGAGGCGCCGGCCTTGTGGTACCGGTAAAGTGCTGTGGCGAC
CGCGATCTGGCGCGAAGCCTGATGAAGATCGAATATTCGGCTGAATATTCGCTAAGACAT
GTGTGGCGGCGTCCGATCCTGTCACAACCTGCCCCTAGGGTCGGTGCATGA
>AA QryID: NC_000962_(-)_665434_665601 HitID: NC_020245_(-)_666657_666824
rank=1 bitscore=104.0 evalue=2.0E-20 perc_ident=100.0 perc_conserv=100.0 perc_gaps=0.0
query_cvg=100.0 subj_cvg=100.0 alpha=71.47 beta=5 prob=0.9748988815526279
ATGGCCGCCGACCCGCAATGTACACGCTGCAAGCAAACCATCGAACCCGGATGGCTATAC
ATCACCGCCCATCGCCGCGGTCAAGCCGGGATCGTCGATGACGGCGCAGTACTGATTCAC
GTGCCCGGTGAATGCCCGCACCCCGGGGAGCACGTTCCGCGAAGCTAG
>AA QryID: NC_000962_(+)_811055_811186 HitID: NC_002755_(+)_813244_813375
rank=1 bitscore=46.6 evalue=0.002 perc_ident=67.44 perc_conserv=67.44 perc_gaps=0.0
query_cvg=100.0 subj_cvg=100.0 alpha=67.44 beta=1 prob=0.9748927010965236
TTGACGCCGGATCAGCTATCCGATGATTCAGGCGGAGATCTCGACGATCGTGGGCGCTAC
CGCCAATCCGGTATCCGGGTAGATCATGATCGACATGGGTTGATCTGCCCTGGTGGGGCG
GACTCACATTAG
>AA QryID: NC_000962_(-)_1056786_1057070 HitID: NC_016768_(+)_3351640_3351924
rank=1 bitscore=131.0 evalue=1.0E-28 perc_ident=86.17 perc_conserv=86.17 perc_gaps=0.0
query_cvg=100.0 subj_cvg=100.0 alpha=69.0 beta=12 prob=0.9748900646510263
ATGCCGCGAAGCTGGTGGCCAACAGCACCCCGTATCTGGGCGTATTCGGGCAACGGCACG
CCCTCGGCGGAGTTGAGCGGCAATAGGCGACAGGCTTTGCAAAGCAAGTACCAAGCGGCG
CAACGGCGGTTCAACTTCCCGGATGGCGAAACCCACACCTGGGCGTACTGGCGTGCGCCA
TTGCAGGCCATGCTGCCTGATCTGCAGCGGGTGCTGGGCGACGGTCAGCGGCCTCGGCGG
GGGCCGAGGCCGCGAGAGCGCAGGCCCGCATCGCCGAGCCTGTAG
>AA QryID: NC_000962_(-)_3135603_3135719 HitID: NC_002755_(-)_3129817_3129933
rank=1 bitscore=52.8 evalue=3.0E-5 perc_ident=100.0 perc_conserv=100.0 perc_gaps=0.0
query_cvg=100.0 subj_cvg=100.0 alpha=69.85 beta=1 prob=0.9748283131294188
TTGATTTCAGGGGTGACGTTCTTGGCGACTCTCCGGCGCGTCGGCCGGTCGTCCGCGAAG
CGCGTTTTGTCTCTCGCCGTTGCACCGCATCGCCGCCAGCCCGTACAGGGCACTTGA
>AA QryID: NC_000962_(-)_2614068_2614352 HitID: NC_002755_(-)_2609913_2610197
rank=1 bitscore=177.0 evalue=1.0E-42 perc_ident=100.0 perc_conserv=100.0 perc_gaps=0.0
query_cvg=100.0 subj_cvg=100.0 alpha=75.36 beta=1 prob=0.9747813112897978
ATGTGCGTGAGCCGCGGCGGAATGCTCGAGCGTGCAAAGTTTCCGCGGCGCTTGTTTTGT
CGTAATGAATACATCTTGTTTTCCAAGGTCAGCAGCCTCCATGCAACCCGGTGCGATTCC
GCTATCGATGCGAAACCGGGTTTGCGACGGTTGATCAGAGCCCATCTCGACACTATTCAC
CGTGTGACACCGTGTTTGAATCGTCGCAGTGGCCTACCCGCAGCGCGGGGTATCAAGTAC
ATTGAAATGGCCCAAGCTGATGAGCGGCCCGTCGTGGGAGGATGA
>AA QryID: NC_000962_(+)_183363_183569 HitID: NC_019965_(+)_183197_183403
rank=1 bitscore=105.0 evalue=6.0E-21 perc_ident=97.06 perc_conserv=98.53 perc_gaps=0.0
query_cvg=100.0 subj_cvg=100.0 alpha=71.36 beta=3 prob=0.9744152750092361
TTGTGGAGCTTCCACGACAACCGCATTGCGATGCGGTTCCAGTACGAATCCCGTGACCGC
AACGGCCAGTGGTATCGCAGCTACGGCACCGAACTGTGGCGAAGCCAGCATCAACGACGT
GCCGATCGCCGAATCCGAGCGTCGCTACCTCGGTGCGCGCTCGGCATCCGAGTATGGCCA
GGAAATACCGCTCTGGTAGCCCGGTAG
>AA QryID: NC_000962_(-)_2125042_2125233 HitID: NC_019951_(-)_2209724_2209915
rank=1 bitscore=104.0 evalue=1.0E-20 perc_ident=100.0 perc_conserv=100.0 perc_gaps=0.0
query_cvg=100.0 subj_cvg=100.0 alpha=71.87 beta=2 prob=0.9743799634516117
GTGCCGCGTCTCAATCTAGTGCAGCGAGTACGGGTCGGCGCAGGGTTTGTTGCGGCGTCG
GCGCGTCTCGCCGGCGCAAAGTCCAAGCCGCGGGTCATTGTTAGGTTAGACTCCACTAAT
CAGCTTAGGCTAGACTCCACTAATATGCGGCTTGATCGTTCGCCGACGCAGCATCTGCTG
GGGGTTTGCTGA
>AA QryID: NC_000962_(-)_2959847_2960104 HitID: NC_019950_(-)_2997680_2997937
rank=1 bitscore=132.0 evalue=5.0E-29 perc_ident=89.41 perc_conserv=89.41 perc_gaps=0.0
query_cvg=100.0 subj_cvg=100.0 alpha=68.4 beta=11 prob=0.9740984081082045
TTGTTGCCGTTAGGCGCGAACATCACGCTCGCCGAACTGCCCGAACCCGAACTGCGGCAA
CTGTTTCCGCACGAGGAGCTGCAGATCCCGGTATCCTGCGGGGGCCTCGGCGCTGGTGCC
GGCGGCCGCATGGACATGCGGGCCGTCGGACTCGTGCCGGTACGCCGAGCGTCGCGCTAC
TGCACAGGCTTCTTTGTGCTGATTCACAGCTACCTCACATTAATGGGCCGCGGCGCGAGG
TTACGGTTGTCTCGTTGA
>AA QryID: NC_000962_(+)_2680458_2680667 HitID: NC_017528_(+)_2680221_2680430
rank=1 bitscore=133.0 evalue=2.0E-29 perc_ident=100.0 perc_conserv=100.0 perc_gaps=0.0
query_cvg=100.0 subj_cvg=100.0 alpha=67.39 beta=15 prob=0.9740548137510097

ATGTTCGTAATCCGGCTCGCCGACGGCGAAGAAGTCCACGGCGAGTGCGACGAGCTGACG
ATTAACCCAGCAACCGGCGTCCTCACGGTCTGCCGGGTCGACGGGTTCGAGGAAACCACC
ACGCACTACTCGCCGTCGGCGTGGCGGTCGGTGACACACCGCAAGCGGGGGGTCGGCGTT
AGACCATCCCTGGTCTCAACTGCTCAATAA
>AA QryID: NC_000962_(+)_1473464_1473613 HitID: NC_020245_(+)_1470311_1470460
rank=1 bitscore=47.8 evalue=0.001 perc_ident=65.31 perc_conserv=65.31 perc_gaps=0.0
query_cvg=100.0 subj_cvg=100.0 alpha=65.31 beta=2 prob=0.973803015510048
ATGACAACAAAGTTGGCCACCAACACACTGTTGGGTCCTGAGGCAACACTCGGACTTGTT
CCAGGTGTTGTCCCACCGCCTTGGTGGTGGGGTGTGGTGTTTGAGAACTGGATAGTGGTT
GCGAGCATCAATGGATACGCTGCCGGCTAG
>AA QryID: NC_000962_(+)_2684487_2684588 HitID: NC_002755_(+)_2681818_2681919
rank=1 bitscore=50.8 evalue=1.0E-4 perc_ident=100.0 perc_conserv=100.0 perc_gaps=0.0
query_cvg=100.0 subj_cvg=100.0 alpha=67.51 beta=1 prob=0.9735736306312341
GTGACCGCCGCCAAGAACCCGCGCCCCGATCTGCGAATCGCGCTGGTGGCTCGGCGGCAC
ATCGACCTCAAGCGGGTCTGCAGCTGTGGCTGTCGGCCTTGA
>AA QryID: NC_000962_(+)_2608348_2608518 HitID: NC_020245_(+)_2555090_2555260
rank=1 bitscore=109.0 evalue=4.0E-22 perc_ident=100.0 perc_conserv=100.0 perc_gaps=0.0
query_cvg=100.0 subj_cvg=100.0 alpha=70.31 beta=2 prob=0.973382163944395б
GTGGCGATCTGTGACCAGACACTAGGCTGCGCCAGCGACGGCGTCAGCCGCTTCGGTCGA
TTCGAGCCGAATGGTCGACGGCTGCGGAACCGACCGCAAAACTGGGGCAAAAGGTTCACC
GCGGGTGTAAGCCAGCTAGGTGAACCGATCCCGCTGGCCCATGGCCTATAG
>AA QryID: NC_000962_(+)_3965625_3965822 HitID: NC_020245_(+)_3874438_3874635
rank=1 bitscore=113.0 evalue=3.0E-23 perc_ident=100.0 perc_conserv=100.0 perc_gaps=0.0
query_cvg=100.0 subj_cvg=100.0 alpha=70.36 beta=2 prob=0.9732888786038798
TTGATCGGGGCGGTCGCCGACGCGGTGCGTCCGGCCCGGCCGGATGGTCATGGTGCGGCG
TGGGAGCAACTGCTGGGGGTAGCGAACTGTTCACTACCCCCTGGCAATCAGGTGGTCCCG
TCTCCCTGGCAAGCGACAGCTCGGGCAATCCACCTGGTAATCAAACAATTTCGGGCGGCT
GGCGAGTTGTCGCGCTGA
>AA QryID: NC_000962_(+)_986674_986823 HitID: NC_018143_(+)_986677_986826
rank=1 bitscore=94.7 evalue=1.0E-17 perc_ident=100.0 perc_conserv=100.0 perc_gaps=0.0
query_cvg=100.0 subj_cvg=100.0 alpha=67.12 beta=7 prob=0.97327703110508
GTGCGGCTCGCCGGCAACGTTGGCAACATCCCGATTCCCATTGATTGCACGTTGCGCGGC
CTAACCCAATATTCCCGGACGAACAACGCCGAGGTCGTGCAGAGCGTCGAGACACACCAC
CGTCCCGCTAACTTTGATGCCCTCACCTGA
>AA QryID: NC_000962_(-)_1261722_1261841 HitID: NC_002755_(-)_1261269_1261388
rank=1 bitscore=51.6 evalue=7.0E-5 perc_ident=100.0 perc_conserv=100.0 perc_gaps=0.0
query_cvg=100.0 subj_cvg=100.0 alpha=66.67 beta=1 prob=0.9731504761947376
ATGGTCCGGGCCTATCCCGAATCTCACCCACATGACGATCGCGGCGGGCCCGATGAGGAC
ATCCGGCGCCGAGCGATTTTGCCGCCGGCCGGGCCATGCCGGCCGATGTCACCGGAGTAG
>AA QryID: NC_000962_(-)_1178604_1178816 HitID: NC_018078_(+)_3232905_3233117
rank=1 bitscore=129.0 evalue=4.0E-28 perc_ident=100.0 perc_conserv=100.0 perc_gaps=0.0
query_cvg=100.0 subj_cvg=100.0 alpha=68.83 beta=7 prob=0.9731069395363392
GTGCGGCCTTGCGGATCAGCCAGGGGCCAACGGCCAGCCAGCTGCGGTATGCGCGAGTGC
CTAGCCATGGTCGGCCAAGTCTTCACCGCCGGCGACATCGACTACCGGATGTTTCAAACG
ATCGTGTGTCGCAGCGATCTGACCGTCGACGGTGAGGTGTTGGCGCCGTGGCCGCCGAGC
TCGCTCTCAGGGCGGCTCGCTGGCCGTCGATGA
>AA QryID: NC_000962_(-)_2732784_2732912 HitID: NC_020245_(-)_2667600_2667728
rank=1 bitscore=78.2 evalue=6.0E-13 perc_ident=100.0 perc_conserv=100.0 perc_gaps=0.0
query_cvg=100.0 subj_cvg=100.0 alpha=66.96 beta=4 prob=0.9731013428583652
GTGCGCGACGAGGACGATGACGAGGACCCGTACGCGGCGCATTCCTCGATTGGTCGCTGG
TGGCTTTTCGGCAAACGCGATGATCGTGGGCTGGGCACGTTAGTCTGCTGCGTAATCGGC
GCGTCGTAG
>AA QryID: NC_000962_(+)_99379_99573 HitID: NC_019965_(+)_98931_99125
rank=1 bitscore=75.5 evalue=8.0E-12 perc_ident=100.0 perc_conserv=100.0 perc_gaps=0.0
query_cvg=100.0 subj_cvg=100.0 alpha=63.99 beta=9 prob=0.9727966489849533
ATGTCGCCGGACCGACGAAGGACCGACGCTGTGTCAACACTGCCAACCTGGGTCAGCCAG
AGCTCTACCGACCGCGGCGTGGTCGCGCCAATCACAGCGCGTGCCCGCGACGCACTGCAG
GCCGTGCTGCGCGCCAGGCGCCGCGGCCAGCGCTCTGACTTGCGCCTTATGCGCAGAGGC
GTGGAGCGTTGTTGA
>AA QryID: NC_000962_(+)_2228676_2228906 HitID: NC_017026_(+)_2223965_2224195
rank=1 bitscore=136.0 evalue=4.0E-30 perc_ident=100.0 perc_conserv=100.0 perc_gaps=0.0
query_cvg=100.0 subj_cvg=100.0 alpha=54.03 beta=20 prob=0.9727897807661962

ATGACCGACTCTGTGGTCGTCCGCGTCAAGCCCGGCAGTCACAAAGGACCCCTGGTCGAG
GTCGGTCCCAACGGTGAGCTGATTATCTACGTCCGCGAGCCGGCGATTGATGGCAAGGCC
AACGATGCGGTCACCCGGCTGCTCGCAGCTCACCTTCAATTGCCAAAGAGCCGAGTCAAA
TTGGTGTCCGGAGCGACGTCGCGGTTCAAGCGTTTCCGTCTGAGTCGTTAA
>AA QryID: NC_000962_(-)_3239570_3239794 HitID: NC_009525_(-)_3251507_3251698
rank=1 bitscore=105.0 evalue=1.0E-20 perc_ident=85.92 perc_conserv=87.32
perc_gaps=11.27 query_cvg=95.95 subj_cvg=100.0 alpha=70.69 beta=3
prob=0.9727599766187491
TTGCCGCGCTTGCGGGGGACTGCGCCCGGCTTCGCCGCGCTTGCGATCATCACTGGTTCG
ATGGTGATGACCTGGGGGCACCTCCCGCTTGCGGGGGACTGCGCCCGGCTTCGCCGCGCT
TGCGATCATCACTGGTTCGATGGTGATGACCTGGGGGCACCTCCCGCTTGCGGGGGACTG
CGCCCGGCTTCGCCGCGCTTGCGATCATCACTGGTTCGATGGTGA
>AA QryID: NC_000962_(-)_1616365_1616601 HitID: NC_017528_(-)_1617491_1617727
rank=1 bitscore=67.8 evalue=2.0E-9 perc_ident=62.82 perc_conserv=62.82 perc_gaps=0.0
query_cvg=100.0 subj_cvg=100.0 alpha=62.82 beta=4 prob=0.972732306734732
GTGGGGCGAATGGCGCCGGATCGACATGCGCTAGGGCTTGGTCTGCTGGTCGGGGCTCTC
GAACGCGGGATGGCCGCCGGGGTGATTCAGCGCGTACCGTTGCCACCACTTTCGCATCTG
CTGCTGGCCGCGCTAACCGAATCCGCGCTGCAGATCGCGGACGCGACGGACAAAGACCGG
ACCAGAGTCGAGGTCGAACGCGCATTTATGGCCCTACTCGAAGGTCTACGGGTGTAG
>AA QryID: NC_000962_(-)_3487793_3488026 HitID: NC_002755_(-)_3483590_3483823
rank=1 bitscore=131.0 evalue=1.0E-28 perc_ident=100.0 perc_conserv=100.0 perc_gaps=0.0
query_cvg=100.0 subj_cvg=100.0 alpha=68.42 beta=3 prob=0.9722634911481204
GTGCCTTCATCACTCGCAAAGAGATGCTCGACCCCGGCTCCGCCGCGCCGAGGCGGGCAC
ACCAAGATCCTGCACCGCCTGATGAGCCGCCAACCGATCATGGGCCCAACCCCAATTAGG
GGCCGTCGGTTGCGGGTAGTGCGTGAGGAGTTTGCGTGGTTGCGGTCCCGGCTGCCGACG
TTGTGGACGAACTCGTATTTTGTGGCCACCGTTGGCGGTTTCGGCTTGAGTTGA
>AA QryID: NC_000962_(-)_3378495_3378671 HitID: NC_002755_(-)_3374231_3374407
rank=1 bitscore=73.9 evalue=2.0E-11 perc_ident=77.59 perc_conserv=77.59 perc_gaps=0.0
query_cvg=100.0 subj_cvg=100.0 alpha=60.5 beta=8 prob=0.9720503764602819
GTGGGTTTGATGGGCCCCCATCCGAATGCCGTTGCGCTGTTGGTGGATCCGGTAGCCGAT
GTGGTCGGCGAGGAGCTTGGTGTGTGGCTGGCGGCCTCGCTGCCGGCGGTGGTGCGTGGC
CAATTGCGCAGGCGCGGGTGTGAGGAATCTGCGGCGTCGTCGTCTACGCCTGGGTAA
>AA QryID: NC_000962_(-)_1338605_1338832 HitID: NC_002755_(-)_1338092_1338319
rank=1 bitscore=117.0 evalue=1.0E-24 perc_ident=93.33 perc_conserv=93.33 perc_gaps=0.0
query_cvg=100.0 subj_cvg=100.0 alpha=65.96 beta=4 prob=0.9720463080085637
ATGAGCCAAAGTGCAATCTGCACCTACCGCCGCCGCCGAAGGGTAAATCTTCCCAATCAG
CGCCCACTTGATGTGCAAAATGCACCCGCCGCACAGTTCACCATGTGGTTCGACGCGACA
ACGACGACTAGCTGGGTTACTATGCCCGGTCGGTGGCGGATTTCGCAAATTGCGGTGTGT
CGCAAGTTGTTCAACACGGTCGACTCATCCAGCAGACCCACACCGTGA
>AA QryID: NC_000962_(+)_3421221_3421430 HitID: NC_020245_(+)_3344959_3345144
rank=1 bitscore=71.2 evalue=1.0E-10 perc_ident=77.05 perc_conserv=77.05 perc_gaps=0.0
query_cvg=88.41 subj_cvg=100.0 alpha=72.58 beta=1 prob=0.9718709484145992
ATGACGGTGTGGAAACCGCGGAATATGGGTGTGCTCCAGCGACGAAAGGCTCAATCGATG
AGCGCGACTAAAAGCAAGGGTTTGCGGGCGTTTCAGACACTGGTCGCGGCGCTGGCTGCG
GTAGTTGCAGTACTAGCAGCGGGCTGCGCTACCCAGCGCGTTCCCACGGTTCTGCCGGAA
TCGGAGTTAATTCCTCAAAGCCTCGGTTAG
>AA QryID: NC_000962_(+)_139956_140144 HitID: NC_017026_(+)_140040_140228
rank=1 bitscore=72.0 evalue=9.0E-11 perc_ident=80.65 perc_conserv=80.65 perc_gaps=0.0
query_cvg=100.0 subj_cvg=100.0 alpha=54.31 beta=21 prob=0.9718174923937907
ATGACCACGATGATCATGACTTTTGTTGTTCCACAACGTGTTACCCGTGCGACGAAAGGG
CGGGCACGGTCGCTGCTGCGGGTGAGTCGGCGTCTGACGGACACGTTTCGCGCACCGCTC
GCCTGGACCCCGCAGGAGCGGGCCGACCGGTATGTGGCACGTATGCCGATCGCGGTGATT
GCGGACTGA
>AA QryID: NC_000962_(+)_2206950_2207207 HitID: NC_009525_(+)_2216849_2217106
rank=1 bitscore=157.0 evalue=2.0E-36 perc_ident=100.0 perc_conserv=100.0 perc_gaps=0.0
query_cvg=100.0 subj_cvg=100.0 alpha=66.76 beta=3 prob=0.9717650900123531
GTGCTGCTGCCAAGACCCCAGGAAGTCTCGGACGAGAGACTCGCTAGCCTCCGTGGTATC
GGGCATCCCTATCACCCCTGCTCGATCCTCAATATCGGACTAACAAAATACATCATCGCG
CCTGTATACGCGATTACATTGCAATTTATCCTTATCACCCTTCTTAGAGTGCATATCAGT
AATAGACATATCGCGCTCCTCGCGCCCCAGGAGGCGGTCGACGAATTCGCCGTGCGCAAC
GACATGAGCCGTCGCTGA
>AA QryID: NC_000962_(-)_2108948_2109064 HitID: NC_020245_(-)_2080062_2080178

rank=1 bitscore=65.5 evalue=4.0E-9 perc_ident=100.0 perc_conserv=100.0 perc_gaps=0.0
query_cvg=100.0 subj_cvg=100.0 alpha=62.88 beta=2 prob=0.9717245579810239
TTGTGGCGTGATCAAACGTGTGTGGCGCCCCACGGTGTTTTCGTGGGCGCTTTTCTGCTG
TTCAGCGGTGCTTTCAGGTGGAGTTCAACGGTGTCGGCGTTTCGCACGGTGCACTGA
>AA QryID: NC_000962_(-)_3568835_3568957 HitID: NC_017528_(-)_3563244_3563366
rank=1 bitscore=30.4 evalue=142.0 perc_ident=65.0 perc_conserv=65.0 perc_gaps=0.0
query_cvg=100.0 subj_cvg=100.0 alpha=65.0 beta=2 prob=0.9717075299857056
GTGATTGACAACAACGCATTTGGTGTCGGCGCGGCTGCCGCGGTGCCAACCGCGGGAACC
CCCCATAGCAAGAACCGTGTCGCCGCCGCGACTGACGAGTCCGTGGAGCGGGGCTCTACG
TAA
>AA QryID: NC_000962_(-)_3546877_3547056 HitID: NC_020245_(-)_3471364_3471543
rank=1 bitscore=77.4 evalue=2.0E-12 perc_ident=100.0 perc_conserv=100.0 perc_gaps=0.0
query_cvg=100.0 subj_cvg=100.0 alpha=59.38 beta=2 prob=0.9710161044566703
TTGTCCTCGGCCGCCGTCACCAGGCCGCCGGGTCAGCGGAAGCGGACGTTCAACGTCGCC
TGGCCAAAGGTCTTCCGGCCGCCAGTCATCGCGGCGACGGTGACGACACCCGTGGGAGTT
GCCGGGTCCAGCGACTTGATTCGGCCGCTGTATTCGATGTCTGCTCCCTCGGTGGCCTAG
>AA QryID: NC_000962_(-)_1174927_1175199 HitID: NC_020245_(-)_1175746_1176018
rank=1 bitscore=116.0 evalue=4.0E-24 perc_ident=80.0 perc_conserv=80.0 perc_gaps=0.0
query_cvg=100.0 subj_cvg=100.0 alpha=59.94 beta=2 prob=0.9709860317939165
TTGCGTGGCACCACTTCGACCGGGCCACATTTTAGATCGGACGCGACGAGATCACGATCC
CGGGATCGGATCAGACAATCGGAATCTAACTCACCCGAGCGCTCGATCGCCGATACATTC
CGGCTCCGCTACGAAGTCGGTTACGAACTGGCGCGTGACGCGCTGCGAGAGTGGCTGCGC
CGAGGCGGCAAACCCGCCCGGCTGATCGAGATCGCCACCCGCCTACCGCGGGAGAAGTCC
CCGGTCTTACAAACATTGGCGATGCTGGCGTGA
>AA QryID: NC_000962_(-)_930553_930684 HitID: NC_020245_(-)_931380_931511
rank=1 bitscore=71.6 evalue=7.0E-11 perc_ident=100.0 perc_conserv=100.0 perc_gaps=0.0
query_cvg=100.0 subj_cvg=100.0 alpha=58.91 beta=1 prob=0.9709604875095961
TTGCGCGAGGCGCACGAACTTGTCGAATCTGTCGAACGTGCGGGGCCAACGCCCGCCCAA
ACGACCGCCGAGCCGTGCCGGATCTCCGGCGGCCATCGGATGTTGACGAGTGTCAACTTC
GTCTCCCGGTAG
>AA QryID: NC_000962_(+)_2786232_2786519 HitID: NC_018078_(-)_1628636_1628893
rank=1 bitscore=121.0 evalue=1.0E-25 perc_ident=85.88 perc_conserv=85.88 perc_gaps=0.0
query_cvg=89.47 subj_cvg=100.0 alpha=57.55 beta=8 prob=0.9708800125060484
ATGGGGCAGGGTGACGGCGATAACATCCCAATGACCGTGATTCTTCGAACCATGGCGACG
TACGGGCCACGACAACCTGCCATCGAAGGGGCGACGACAATGAAGACAAGGAACCCACGG
ACGCTGCTAACCTGGCTGCTCGGCGCGATAGTTACTGGGTTGTACGTGGTTTTCGCTACG
GGCTGCCAATTGCAAGCGCCCGCGCCTCCCACTCCGGAAATAGGTTGGTCGGGCCCGCAG
GCTCCACTGCCGGCGCCGGATGCGGCGCCAACGCACCTCGGCGTCTAG
>AA QryID: NC_000962_(+)_1946860_1946982 HitID: NC_002755_(+)_1937662_1937784
rank=1 bitscore=50.8 evalue=1.0E-4 perc_ident=100.0 perc_conserv=100.0 perc_gaps=0.0
query_cvg=100.0 subj_cvg=100.0 alpha=54.31 beta=4 prob=0.9707893394790843
GTGCTGGACACCGCCTACCGAGACCACCTCGAGCGGTTCGTTCGCAAACCACCCGAGCCA
CCCGCGCTACCGGCCTTCAGCGCGATCAACCCACCACCAAAGGAGGACCAGCCGACTCAA
TGA
>AA QryID: NC_000962_(-)_4320165_4320257 HitID: NC_017026_(-)_4288684_4288776
rank=1 bitscore=48.5 evalue=6.0E-4 perc_ident=100.0 perc_conserv=100.0 perc_gaps=0.0
query_cvg=100.0 subj_cvg=100.0 alpha=49.12 beta=4 prob=0.9705726791354996
GTGACCACGGTCAACCCGCCGACCGAAGCGATCAGCGGCCGCGTCGAGCACCTCCGGGGA
TCGACCCTCGGCTTTCGCAATCTCACCAACTAA
>AA QryID: NC_000962_(+)_183388_183561 HitID: NC_020245_(+)_183645_183791
rank=1 bitscore=76.6 evalue=3.0E-12 perc_ident=97.92 perc_conserv=100.0 perc_gaps=0.0
query_cvg=84.21 subj_cvg=100.0 alpha=53.79 beta=4 prob=0.970464585310799
TTGCGATGCGGTTCCAGTACGAATCCCGTGACCGCAACGGCCAGTGGTATCGCAGCTACG
GCACCGAACTGTGGCGAAGCCAGCATCAACGACGTGCCGATCGCCGAATCCGAGCGTCGC
TACCTCGGTGCGCGCTCGGCATCCGAGTATGGCCAGGAAATACCGCTCTGGTAG
>AA QryID: NC_000962_(-)_565482_565619 HitID: NC_002755_(-)_566930_567067
rank=1 bitscore=38.1 evalue=1.1 perc_ident=57.78 perc_conserv=57.78 perc_gaps=0.0
query_cvg=100.0 subj_cvg=100.0 alpha=57.78 beta=1 prob=0.9646192712978644
ATGTCAGGCCCAGCTGATCCGGTCACTACATGTCTCCTTTGCTGCACGTCATCGACGGAG
TCGCTGTTGGCCAGCGCGGCCGATCGGCTGGATCAGCCTGCGGTCGACCTCACCCAGATG
ACTGTCCTTCAGGTTTGA

- ***Mycoplasma hyopneumoniae* 7448 uid58039s**

No vsORFs

sORFs

>AA QryID: NC_007332_(-)_538789_539031 HitID: NC_017509_(+)_256001_256243
rank=1 bitscore=103.0 evalue=3.0E-20 perc_ident=98.75 perc_conserv=98.75 perc_gaps=0.0
query_cvg=100.0 subj_cvg=100.0 alpha=98.75 beta=1 prob=0.9999032783641713
ATGATAACACTTAATCAAGAAAAATTTGATATTTTATTTAAAAAAAAATTAATTGAAAAT
TTAATTGAAAATCATAAAGAAAAATTAATTGAATTTGGCAAAAGTCAAAATATTGATTTT
GTTACTGATTTAGAAACTTTATCAATTGCCAATTTAAATGATCTTTATTCAGAACTAGAA
CTTGTTAAAGAGTATCTTAAAATCGAATTAAAATCTAGCATTTTATTTCAACTGAAAAAT
TAA
>AA QryID: NC_007332_(+)_724272_724532 HitID: NC_006360_(+)_708007_708267
rank=1 bitscore=135.0 evalue=6.0E-30 perc_ident=96.51 perc_conserv=97.67 perc_gaps=0.0
query_cvg=100.0 subj_cvg=100.0 alpha=96.51 beta=1 prob=0.9999014273086297
ATGATAAATTTAATAGAAAATATAGTTGTAAAAGCAGAGCAAAATAACAAAATCCCAAAA
AAACAGATAGATTATTTTATTAAAACTACTACCACATTAGAGAGTTTTCTAAAAGATAAG
GAAATAAAAAAATATATTACAACTGACTACTCTACTAGTTTTATGCATTCAAGTAGTCCA
GAAAATTTTTTGTTAAAAAACCAAATGCCCGTTATGGTTGTATTTGCTAATTTCAATGTT
GGACACAATATTATCATATAA
>AA QryID: NC_007332_(+)_429766_429966 HitID: NC_017509_(+)_444412_444612
rank=1 bitscore=88.2 evalue=1.0E-15 perc_ident=98.48 perc_conserv=98.48 perc_gaps=0.0
query_cvg=100.0 subj_cvg=100.0 alpha=84.23 beta=1 prob=0.9998978515414526
TTGGATAGGAATTCTCAATTTTTTGCGATTTTTACTCTTCAATTTCAGGTAATAATTTGT
TCTCAAAACTGCGATTTCGATCTCTTGGTATTTTTAGATGAATATTATTACTATTATAAT
TCCCAGTTTTGCCCGAAAACCCGTTTGGCTTATTCAGTCTATGCACACCATTTTTGCTTC
GGTTACTTTTTCATAGCCTAA
>AA QryID: NC_007332_(-)_421384_421575 HitID: NC_017509_(-)_436033_436224
rank=1 bitscore=87.0 evalue=3.0E-15 perc_ident=98.41 perc_conserv=98.41 perc_gaps=0.0
query_cvg=100.0 subj_cvg=100.0 alpha=73.95 beta=1 prob=0.9998968400719386
ATGACAGGTGATAAATCTCAACTTGAAAAAATAAATTCAATTTTAAAAATAGTCCCTAAT
TTACCAAACCAAATTCAATTTAATCGGCTTTATTATATAACTAATATCTATTTAAATATA
GATCCAAAAAAGAATTTGCCGAAACTTATTAGTTTAATTTCAAAATATTATAATAAAGAA
AAGAAAATTTAG
>AA QryID: NC_007332_(+)_742631_742747 HitID: NC_017509_(+)_743485_743601
rank=1 bitscore=46.2 evalue=0.003 perc_ident=97.37 perc_conserv=100.0 perc_gaps=0.0
query_cvg=100.0 subj_cvg=100.0 alpha=97.37 beta=1 prob=0.9998899200251483
TTGGATTTTACAGAAGAATTTAGCCAAATTTATAAAAAAGAAAAATGCAAAATAGACACA
AAGAGTCAATTTTGGATGTATATAAAACAATTTAATAAGAAATTGAAATATAATTAA
>AA QryID: NC_007332_(-)_528983_529249 HitID: NC_017509_(+)_265786_266052
rank=1 bitscore=84.7 evalue=2.0E-14 perc_ident=85.23 perc_conserv=85.23 perc_gaps=0.0
query_cvg=100.0 subj_cvg=100.0 alpha=85.23 beta=1 prob=0.9998819923012476
TTGTTTTTAATTAGTACTAAATATTTTTTTAGTATTTTTTTCTTATTTCTTTTTTTATTA
ATTTATAAAAAAGTAAAGACTTCATTTAGCGTTATTCTAAAAAATCTTGTAAAATCTCCT
CAAAAATTTAACAAAATATCAATTCTTATTATTGAAAGGAAAAATTTAAAATGCAAAATT
TCATCTCTCTTTTTCAACAAACAGATCCAACACAAAAAAATCTTGAAGAAGTTCAGAAAT
CAGTTAAACATGTTTCAGATTCAGTAA
>AA QryID: NC_007332_(-)_857471_857695 HitID: NC_006360_(-)_831838_832062
rank=1 bitscore=89.0 evalue=8.0E-16 perc_ident=74.32 perc_conserv=74.32 perc_gaps=0.0
query_cvg=100.0 subj_cvg=100.0 alpha=56.04 beta=3 prob=0.9921784846898027
TTGGAAAGACCGATTAAAACCCCAAAACTGAAAAAAACACGTAAAAAGAAGTATTATTTT
CGTCTTTTTATAAAGGAAATGAAAAGAGTGAAATGGCCTAGCGGCCGTGTTACCTTTAAA
AGTTTTGGTCAATCCTTGCTTTTTTCCACAATTTTTATGTTTGTTTTTTTTGCAGTTACG
GTTATTGCGGCACTTATTTGGAATCAGGCTGGCGTCGGAATTTAA
>AA QryID: NC_007332_(+)_243003_243188 HitID: NC_017509_(+)_239433_239612
rank=1 bitscore=56.2 evalue=6.0E-6 perc_ident=79.66 perc_conserv=79.66 perc_gaps=0.0
query_cvg=96.72 subj_cvg=100.0 alpha=78.36 beta=1 prob=0.9506326380420556
GTGAAGATGCAAGCAAAAAACAATTTATCTCAATTTGTGAATATGATTTTTTCTTATAAA
ATCAATTTTTTTGCATTTTTATTTTTGAATATTTTTTCTAGATCTTTCTCAATTAATAAA

ATTAAGCAAATTTTGAATAATAAATCAATTAACTGTTTGCCGTTTTCGAATCTAATAATT
ATATAA
>AA QryID: NC_007332_(-)_531131_531319 HitID: NC_017509_(+)_263716_263904
rank=1 bitscore=51.6 evalue=1.0E-4 perc_ident=77.42 perc_conserv=77.42 perc_gaps=0.0
query_cvg=100.0 subj_cvg=100.0 alpha=77.42 beta=1 prob=0.9151943030855665
ATGAAAGAAAAAAAGCAAAATGAGAATATTCTATTAGGGAATTTATTAAATTTGCAAAAT
TCTAGTGTTGAGAATTTAAACCAATTAAAATTTCAATTACAAACAGATTTAGATTTGCAT
ATTCTAACTGTAAAACAACAAATTGATATTGTAAATCAACTAATTAAAAACAAACAAAAA
AAACAGTAA
>AA QryID: NC_007332_(-)_264550_264708 HitID: NC_006360_(+)_186644_186802
rank=1 bitscore=46.2 evalue=0.004 perc_ident=76.92 perc_conserv=76.92 perc_gaps=0.0
query_cvg=100.0 subj_cvg=100.0 alpha=76.92 beta=1 prob=0.8670494141551359
ATGAATAAATTAAATTTTTTTTCAAAAAATCGTTTAAAATGTATAAAAAATATTATTAAT
TATTTTTTTAACCTTTTTATTTATCCTTTATTTTATTATTTTAAATCAAAAAAAGTCTAT
AAATTCATAGGGTTTTTAGGTAAAAAATCAATTTTTTAA
>AA QryID: NC_007332_(-)_689110_689274 HitID: NC_006360_(-)_651629_651787
rank=1 bitscore=51.2 evalue=1.0E-4 perc_ident=69.23 perc_conserv=69.23 perc_gaps=0.0
query_cvg=96.3 subj_cvg=100.0 alpha=67.95 beta=1 prob=0.7014466750718507
TTGTATATGCCCAAAGATAAAAATATTATGATAATTAAGAATATTTCACTAAAATATTCA
ATAAATAAAAGGTTTTTTCGGAAAAAGCAACAAAAATTTTTAACTCAAAAATTGAATTAT
TTATATTCAATTTTAAAATCTGGTTGGATTTTTTTGCGCTATTAG

- ***Pseudomonas aeruginosa* PAO1**

vsORFs

>AA QryID: NC_002516_(-)_3108663_3108752 HitID: NC_018080_(+)_2286700_2286789
rank=1 bitscore=95.2 evalue=3.0E-18 perc_ident=100.0 perc_conserv=100.0 perc_gaps=0.0
query_cvg=100.0 subj_cvg=100.0 alpha=44.3 beta=1 prob=0.9999805699936329
ATGCTGCGTTACGTCGTGGGTTTTCTCGCGTTCACCGTGCTGGCGGCCTATCTGTTGCTG
GGGGTTTCCCAGCACGCCTTCCTGCCGTGA
>AA QryID: NC_002516_(-)_3475171_3475257 HitID: NC_012560_(-)_3485013_3485099
rank=1 bitscore=63.0 evalue=2.0E-8 perc_ident=78.57 perc_conserv=82.14 perc_gaps=0.0
query_cvg=100.0 subj_cvg=100.0 alpha=22.25 beta=4 prob=0.9998849513624719
ATGGACGACTACCAGGAAGAACTGCTGGAGCAGCATGCCGCCGAATGCGACCTGCCGGAG
CCGGCGGACGACGCCACCGAGATGTGA

sORFs

>AA QryID: NC_002516_(+)_3116075_3116305 HitID: NC_017548_(-)_2403183_2403413
rank=1 bitscore=138.0 evalue=1.0E-30 perc_ident=100.0 perc_conserv=100.0 perc_gaps=0.0
query_cvg=100.0 subj_cvg=100.0 alpha=75.62 beta=133 prob=0.999931852177036
ATGGACACCAGCCCGCACACCTTTTCCAACCTGTTCCGCCAGTTGGGGCTCCCCGCTGGG
CGGGACGACATCGTCGCCTTTCTCGCCCGGCACCACCTCGAGGAGGGCACGGCGCTGCCC
GATGCGCCATTCTGGAACCCTGCCCAGGCCAGCTTCCTGCGCGAGGCCCTGGCCGAGGAC
TCCGACTGGGCCGAACAGGTCGATGAACTGGCCTTGCAACTGACCCGCTGA
>AA QryID: NC_002516_(-)_5790181_5790441 HitID: NC_017548_(-)_5843638_5843898
rank=1 bitscore=139.0 evalue=5.0E-31 perc_ident=100.0 perc_conserv=100.0 perc_gaps=0.0
query_cvg=100.0 subj_cvg=100.0 alpha=75.99 beta=120 prob=0.9999318521770011
ATGGCCAGGGCGAAGAGCAATACGCCGATCCTCCAGCTGGACGCCGCGCAGACTCAGGAC
GCGGTACTGGCGATCAAGCGCTTCATGGAGGACCGCTTCGAACTGGAGCTGGGTTCCTTC
GAAGCCGAGGAACTGCTCGATTTCTTCGCCCGCGAGTTCGCCCCGTACTTCTACAACAAG
GCCATCCTCGACGTGCAGTCGTACCTGAAGGAACGGTTCGAAAGCATCGAGAGCGATGTC
TGGGCGCTGGAAAAGAGTTGA
>AA QryID: NC_002516_(-)_2330591_2330833 HitID: NC_018080_(+)_3257097_3257336
rank=1 bitscore=131.0 evalue=1.0E-28 perc_ident=92.5 perc_conserv=95.0 perc_gaps=1.25
query_cvg=100.0 subj_cvg=100.0 alpha=66.24 beta=105 prob=0.999931852169476
ATGAACCGAGCCAAGCTCTACCTCTGCTGCGTCGCACTACTGCTGGTGCCGGCCATCGCC
GGTGCGGCGACTGCCGACTACGTCTACGGCATGCCGCTGGACGTGGCCAGGGTGCTGAGC
GTCGACGAGGCGCCCAGCGATACCTGCCAGGTGGTGCGCGCCACCCTGGTGTACCTGGAC
TCCCACGGCGAGCGCCAGGTGTTGCACTACCTGAAGCAGGCGCGTGCGTGCAGCGACGAA

TGA
>AA QryID: NC_002516_(-)_433933_434103 HitID: NC_011770_(-)_427674_427844
rank=1 bitscore=89.7 evalue=4.0E-16 perc_ident=96.43 perc_conserv=96.43 perc_gaps=0.0
query_cvg=100.0 subj_cvg=100.0 alpha=78.12 beta=134 prob=0.999931841651695
GTGGAGGGCAAGGCCAACGCCCATCTGCTGACCTTCCTCGGCAAGGCCTTCGGCGTGGCC
AAGAGCCTGGTGAGTCTCGAAAGCGGCGAGCTGAACCGGCAGAAACGGGTGCGCATCCGC
CGTCCGACCCGATTGCCGGAGGAACTGGGGATCGCCGCTCGACCCGCTTGA
>AA QryID: NC_002516_(-)_5204648_5204830 HitID: NC_018080_(-)_5306068_5306250
rank=1 bitscore=108.0 evalue=6.0E-22 perc_ident=100.0 perc_conserv=100.0 perc_gaps=0.0
query_cvg=100.0 subj_cvg=100.0 alpha=65.0 beta=72 prob=0.9999297654096436
ATGCTCGAAAGCCAGGCCTATAGCTGCCCTTACTGCGGGGAACCGGCCGAGGCGCTGCTC
GACCTGAGCGCCGGCGACCAGCAGTACTACGAGGACTGCCCGGTGTGCTGCCGGCCGATC
CTGTTCGACCTGCGCACCGATGGGCAGGACTGGTCACTGAACGTCCGCCGGGAGGATGAC
TGA
>AA QryID: NC_002516_(-)_1204785_1205006 HitID: NC_017548_(+)_4353149_4353370
rank=1 bitscore=148.0 evalue=9.0E-34 perc_ident=100.0 perc_conserv=100.0 perc_gaps=0.0
query_cvg=100.0 subj_cvg=100.0 alpha=43.6 beta=63 prob=0.9998604836496923
ATGCCTGGCCATCCCGATCCACTGCACGGCGTCACCCTGGAAAAACTGCTCACCGAACTG
GTCGCCCACCACGGCTGGGCGGAGCTTGGCAAACGGGTCGACATTCGTTGTTTCAGGAAC
GACCCGAGCATCAAGTCCAGCCTCGCCTTCCTGCGCAAGACGCCCTGGGCCCGGGAAAAG
GTCGAGGCGCTCTACGTCCAGCTCAAGCGCAACCAAGGCTGA
>AA QryID: NC_002516_(-)_5836470_5836685 HitID: NC_017548_(-)_5891309_5891524
rank=1 bitscore=119.0 evalue=6.0E-25 perc_ident=100.0 perc_conserv=100.0 perc_gaps=0.0
query_cvg=100.0 subj_cvg=100.0 alpha=80.01 beta=57 prob=0.9987903920907717
ATGGGTTTCCTGATACTCTCCCGCCGAGAAGGCGAAGGCATCACCCTGTCCCTCAAGGCC
GACTACCCGGCGGAGGAACTGATTCGGCAATTGCGCGAAGGCGGCATCCGGATCCTGGTC
ACCGATATCATCGGCAACCAGGCCCGGGTCGGGATCGAGGCGCCGCGTGGCGTCCTGATC
GTTCGCGACGAGTTGAAAACGGCACCGAAAGGCTGA
>AA QryID: NC_002516_(-)_5204385_5204648 HitID: NC_017548_(-)_5244318_5244581
rank=1 bitscore=98.2 evalue=2.0E-18 perc_ident=68.97 perc_conserv=68.97 perc_gaps=0.0
query_cvg=100.0 subj_cvg=100.0 alpha=68.97 beta=25 prob=0.9984031905199666
ATGCAGCGCGTATATGAGCCGGCCGACCTGCTGGAGGCCGAGATGCTGGTGGGAATGCTC
GCCAGCGAAGGGATCGCCGCCCATGTCAGCGGCCGCCACCTGGTCGGCGCCATCGGCGAA
CTGCCGCCTGGCGGACTGCTGGCGTTGTTGGTGGCGGACGAGCAGACGGAGCGGGCGCGA
GAACTGATCCTCGCCTACAACGCCGCCGCCCCGTTGGCCGAGGAGCCGCCGGAGCCCTGC
GGCCCCGGCAGCCTGTTGTGTTGA
>AA QryID: NC_002516_(-)_1997217_1997465 HitID: NC_011770_(+)_3870638_3870865
rank=1 bitscore=116.0 evalue=3.0E-24 perc_ident=82.67 perc_conserv=82.67 perc_gaps=0.0
query_cvg=91.46 subj_cvg=100.0 alpha=44.71 beta=31 prob=0.9984012049520422
ATGGAAAAGGGAGGACCACCGATGGGTATCGAGACGGAAGAACCGAACCAGAAGCCGCCG
ACCTTCTGGCAGATGCTGCATAGCGTGATGGCTGCCGCCTTCGGCGTACAGAGCGGGCGA
AACCGCGCCCGCGACTTTACCCACGGCAAGCCCGTGCACTTCATCATGCTGGGCCTGCTC
TTCACCCTGGTGTTCGTGCTGTTGCTGGCCGGGATCGTGAAACTGGTGCTGAGCCTCAGC
GGCCTGTGA
>AA QryID: NC_002516_(-)_3115699_3115896 HitID: NC_018080_(+)_2279555_2279752
rank=1 bitscore=105.0 evalue=6.0E-21 perc_ident=100.0 perc_conserv=100.0 perc_gaps=0.0
query_cvg=100.0 subj_cvg=100.0 alpha=100.0 beta=43 prob=0.9983972660091031
ATGTCCAGAATCGAAGTCGCCGTGCTGGTGGGGACGTCCGTTCCCGAATCGCTGCGCAGC
AAGGGATTGCTGGCCTGTTGGATCCTGATGGTGGACGGCGCCATGTGGGCCGGCCCCTTC
ACCAGCCGTGACGAGGCGGAAGCCTTGAAGGGAACCTGGGAACTGAGTTGGCCGCTGGAG
GCGGCGATCTCCGCCTGA
>AA QryID: NC_002516_(+)_4352524_4352769 HitID: NC_008463_(-)_1172887_1173132
rank=1 bitscore=112.0 evalue=1.0E-22 perc_ident=85.19 perc_conserv=85.19 perc_gaps=0.0
query_cvg=100.0 subj_cvg=100.0 alpha=85.19 beta=31 prob=0.99839607862993
ATGCACTGGCTGGACTACCTGCAATGGCCCGCCATGCTGGTCACGGTGGTGGCCGCCTGG
CTGGTGGCGTCGGCGCATCGGCGAAGACGCAAGGTCGGCTTCTGGGTGTTCCTCGCCAGC
AACCTGCTGTGGATCGCCTGGGGCCTGTACGCCCAGGCCTATGCGCTGATCCTGCTGCAG
GTCTGCCTGGCGGCGATGAACCTGCGCGGCACCCATCGCAACGAAGCGCCACAGGACAGC
GCCTGA
>AA QryID: NC_002516_(+)_796960_797241 HitID: NC_007274_(+)_82424_82672
rank=1 bitscore=120.0 evalue=4.0E-25 perc_ident=82.72 perc_conserv=93.83 perc_gaps=0.0
query_cvg=87.1 subj_cvg=98.78 alpha=48.62 beta=22 prob=0.9983949184128597

GTGTTCCCACAGCAATGGAGGTACCGGCTCATGCGAGTCGAGACAATTAGTTATTTGAAA
CGTCATGCGGCTGACCTGGATTTATCCGAGCCAATGGTCGTCACGCAGAACGGTGTTCCT
GCCTATGTGGTTGAGTCATATGCTGAGCGGAAGCAGCGCGATGAAGCAATTGCGCTGGTG
AAGTTGCTTGCGATTGGCTCCCGCCAGTACGCAGAAGGCAAGCATCGCTCTGTTGATGAT
TTGAAAGCTCGCCTTTCCAGGAGGTTCGCTCAGCCAGAATAA
>AA QryID: NC_002516_(-)_788542_788808 HitID: NC_018177_(+)_1590952_1591191
rank=1 bitscore=110.0 evalue=4.0E-22 perc_ident=70.89 perc_conserv=84.81
perc_gaps=1.27 query_cvg=88.64 subj_cvg=100.0 alpha=49.91 beta=15
prob=0.9983948907099858
ATGAGCACGTCAGCCGATAGAGCAAGACTATTAATCAAGAAAATCGGCCCCAAAAAGGTC
AGCCTTCACGGGGGCGATTACGAGAGATGGAAAAGCGTCAGCAAAGGCGCGATTCGCGTG
AGCACGGAAGAAATCGACGTCCTAGTAAAAATTTTCCCTAATTACGCATTATGGATTGCA
AGCGGTTCCATCGCCCCAGAAGTCGGCCAAACTAGCCCCGACTATGACGAAGCCAATCGA
AACTTGTCCAATCAAAACGCGGGATAG
>AA QryID: NC_002516_(-)_1029541_1029732 HitID: NC_017548_(+)_4528381_4528599
rank=1 bitscore=109.0 evalue=5.0E-22 perc_ident=98.41 perc_conserv=100.0 perc_gaps=0.0
query_cvg=100.0 subj_cvg=87.5 alpha=78.12 beta=30 prob=0.9983947801834276
ATGAGTGTGACAGTGACCGAGCGTGACGATGACCACAAATCCCGCGAGCTGCGTGCGCTT
GGCCGCCGCTGCTGGGACGTGCATCAGCAGGGACTGCTGGTTGGCGTCTTCCATAACGAG
GATGACGCCCACGCCTATCGGATCAAGCTGGAGAAAGAAGAAGAACTGCGGCAGATAGGT
GAATCGGCCTGA
>AA QryID: NC_002516_(+)_863437_863664 HitID: NC_008463_(-)_4794285_4794494
rank=1 bitscore=132.0 evalue=5.0E-29 perc_ident=98.55 perc_conserv=98.55 perc_gaps=0.0
query_cvg=92.0 subj_cvg=100.0 alpha=64.55 beta=28 prob=0.9983947068239909
TTGTGTTTCGGGTCCGGAATGTCGATGCGCACACTTTTCTCCCTTCTCGTCGGTGTCGTC
CTGGCGGGTTTCTCCGGCCTCGCGGCGCAGGCCGCGCCGGCGCCCTTCTACAAATGGCAG
AGCAAGCTCGACGGTCAGGTCGCCTGCATGCAGACCTCGCCGGGAGACGGCTGGGTCAGG
CTCGACGGCCCCTACCGCGATCTCCGCTGCCGCGAACCCCTGCGCTGA
>AA QryID: NC_002516_(-)_3112422_3112667 HitID: NC_008463_(+)_2461317_2461550
rank=1 bitscore=114.0 evalue=1.0E-23 perc_ident=88.31 perc_conserv=88.31 perc_gaps=0.0
query_cvg=95.06 subj_cvg=100.0 alpha=68.95 beta=21 prob=0.9983946879532322
ATGGAGCGCACCATGTCTGTCGACCTGAAGAAAATCGCCGCTGCCTTTCCCGAGTTCCAG
TTGTCCAGCAAGAGCCGCCACGACGGCGCGCTGTTGTTGCAGTTGCAGGCCGCCGACGGC
CGCTACGTGGCTCGCACCATACCCTCGCCGCAGCTCAGCGCGCCGCCGGAGACCGAGTGG
CTGATCCGCAGCATCCGCCGGGAACTGGCCGTGCCGGGCATGGAAATGGCGATGGCGGCA
CGCTGA
>AA QryID: NC_002516_(-)_944467_944706 HitID: NC_008463_(+)_4706899_4707138
rank=1 bitscore=141.0 evalue=1.0E-31 perc_ident=98.73 perc_conserv=98.73 perc_gaps=0.0
query_cvg=100.0 subj_cvg=100.0 alpha=66.43 beta=25 prob=0.9983946753829486
TTGGCCGATAGATGTTTTTTCGGCCAACTAGACTGGCACCATCGTTCACCGTCCCTGTCA
GCCGCCATGAACTACCTGATCTCCCTCGGCATCGGCCTGCTGGTCGGCGCGCTCTACGCC
GTCCTCGACTTCCGCTCGCCGGCGCCCCCCGCCATCGCCCTGGTCGGCCTGCTTGGCATG
CAGCTCGGCGAACAGCTTTTCCCCCTTGGCCGCGACCTGCTGCAGCAGTGGCTGCACTGA
>AA QryID: NC_002516_(-)_5242571_5242849 HitID: NC_018080_(-)_5344003_5344281
rank=1 bitscore=183.0 evalue=3.0E-44 perc_ident=98.91 perc_conserv=98.91 perc_gaps=0.0
query_cvg=100.0 subj_cvg=100.0 alpha=40.84 beta=18 prob=0.9983946591396617
ATGATTCTGACCTTTCGCTGCGACGAGACTCGTCAGCTTTTTGAGACGGGTCTTTCGAGG
CGGTGGGGAGCGATCCTCACAGTCGCTACGCGTAAGCTCGCAATGCTTCATGCGGCTACG
GAGCTTCGAGACCTGCGCTCTCCACCTGGAAACCGGTTGGAGCCGTTGCAGGGAAAGCGG
GCGGGCCAACATAGCATCAGGATCAATGACCAGTGGCGTGTCTGTTTCGTCTGGACGGAT
GCGGGTCCCGAAGAAGTCGAAATAGTTGATTACCACTGA
>AA QryID: NC_002516_(-)_4215259_4215537 HitID: NC_011770_(+)_1321968_1322246
rank=1 bitscore=131.0 evalue=1.0E-28 perc_ident=86.96 perc_conserv=86.96 perc_gaps=0.0
query_cvg=100.0 subj_cvg=100.0 alpha=69.88 beta=8 prob=0.9983946560534553
ATGGCGGGCGATGCGGACTCCAACTCGTCGCCCGACCTTCTTCCCGAGGTTCGCCGGGTC
TCTCCCGGCGACACCTTGCGGCTCTGTACCTGCGGGGCTTCGGCTTCGCTTCCCGATTGT
CCCGCCGACTGCCGCAACGGCCTGACGCTCCACGCGACCCGCGAGCGCCTGTTGCTGTTG
TGCCGCTGCGGGCGCTCCGCCGACCTGCCGTATTGCGACGGCAGCCATGCGCCTTCCGCC
TCCGGGCTGAAGGCCAGGTGGCGGCGTTTCCGCGGCTGA
>AA QryID: NC_002516_(-)_3129158_3129454 HitID: NC_008463_(+)_2444516_2444812
rank=1 bitscore=160.0 evalue=2.0E-37 perc_ident=97.96 perc_conserv=97.96 perc_gaps=0.0
query_cvg=100.0 subj_cvg=100.0 alpha=70.03 beta=6 prob=0.998394655892463

TTGCGGGTTTTATTCAATCTGGGTGAATGCGTGCAGGAAATCTACTCTGAAGAACAGATG
CGCAAGGCGCTGGGCCTTGCCGAAACGCGACCGAAAAAGGTCCGGACCGAGGCCAGCCAG
CCGGTCCGCTACACCATCGTCGAACTGAGCGTGCGCAAGGGCGGGGCGGGATTGCCGCTG
CGTTTCGAACATCGCTCGCGCTCCATCAGCAAGGTCACCGCGCAACTGGAAGCGGAGAAG
GAAGCCAAGCGCCAGGGTTACCAGGTCTGGGCGCTGCTGGATATCCGTCAGATCTGA
>AA QryID: NC_002516_(+)_1246805_1247077 HitID: NC_009656_(-)_3134080_3134352
rank=1 bitscore=156.0 evalue=4.0E-36 perc_ident=96.67 perc_conserv=98.89 perc_gaps=0.0
query_cvg=100.0 subj_cvg=100.0 alpha=54.45 beta=2 prob=0.9983946558747414
ATGAGCATGGCACTTATTGATCTTGCAAGAGATTTTTTGGATGGATCGTTAAGCGGAAAG
AGTTTCACAAAAAAATTCTTTGAAATGTGGCGTAGCGAAGGTGCGTCAGGCTTGCTGAAA
AAGGATGATGATAATCTTGGCGCTTGCCTCAGGCTTATGTTTGGAATGGCAGATTGCTTC
ACGGATGGACCGAAGGATAATGATGGTGAGATAAATGAAGAGGAGCTAAAGCAGGAAGTT
AGAGGGCTTTTGAAGAAATATAAATATATCTAG
>AA QryID: NC_002516_(+)_1247493_1247726 HitID: NC_018080_(-)_4294284_4294517
rank=1 bitscore=136.0 evalue=4.0E-30 perc_ident=98.7 perc_conserv=100.0 perc_gaps=0.0
query_cvg=100.0 subj_cvg=100.0 alpha=98.7 beta=3 prob=0.9983946557457779
ATGACTGTATTGATTTCTGGGTACCTACCATCGGGAAATGATGAGTCCTTGAAATATGAA
AAAACTGTTCCTTTCGAATATATTTCGAAAGTTATGGAGGTGATGCGTTGGAAGAAGGGT
GAGAATATTGAGGGTGAATATCCTATTAAGAATGATGATGTTGTAAGGATTGAGGAGGTT
ATAGGCGAAAAGCTTCCGGTTGGATTGGAGTATTTTATTGGGGTTTACGCCTAA
>AA QryID: NC_002516_(+)_2395691_2395912 HitID: NC_011770_(-)_3476599_3476820
rank=1 bitscore=119.0 evalue=4.0E-25 perc_ident=100.0 perc_conserv=100.0 perc_gaps=0.0
query_cvg=100.0 subj_cvg=100.0 alpha=79.02 beta=16 prob=0.9983946547985433
ATGGCCAGCCATCTGTTCCTGATCAGCTACATCCTCGACGACCAGCCGATGACCTGCGAA
TTGCACAGCGAGGCGGATACCCTCAGCCGCGAAGAGGCCCGCCGCCACTTGGAGGCACTG
CATCGACCGCACGACGCCGAGGCGATCAGCGATATCCAGGTCAGCAAGGTGGAGCGTACC
CATGAGCCGGGCACCACGCCGGGACACTACCAGCAGCCCTGA
>AA QryID: NC_002516_(+)_3087813_3088037 HitID: NC_018080_(-)_2330387_2330608
rank=1 bitscore=118.0 evalue=1.0E-24 perc_ident=85.92 perc_conserv=91.55 perc_gaps=0.0
query_cvg=95.95 subj_cvg=97.26 alpha=50.78 beta=1 prob=0.9983946504321477
ATGACCATCAAGAAAGCTTCTCTTTCGAAGTCTCGGCATTTAAAAGTCGGTTATCAGCTT
TATGAAAGCCGTCATAGCGACTGGGAACGTCGTGGCAAACCACGGCAAATTCCCTACCTG
CGGTTGAGTGGCGATTGGCTGGAGGCTGCGGGCTTTGAGGTTGGCTGCAAGGTGAGCATT
CAGGTCACCAGGCGGCGCCTTGTCATGCAAGTGGTTGAGGATTGA
>AA QryID: NC_002516_(+)_1039035_1039229 HitID: NC_008463_(-)_4612305_4612499
rank=1 bitscore=107.0 evalue=1.0E-21 perc_ident=98.44 perc_conserv=98.44 perc_gaps=0.0
query_cvg=100.0 subj_cvg=100.0 alpha=72.47 beta=15 prob=0.9983946435746068
ATGCTCCCAATGTCGAACAAGGTCGTCCATTTGCATCACAAGGATGAGCAGGCAGCGCTT
GAGCGTTTGAACCGGATTACCGGGCTGCAGTTCAGCAGTTGGCCGGAGTCGCTGGTGCCG
GCGCGCAGCGTCAGCGAACCGTCGCCTGAGAAAGCGCGGGACGAACCCCTGCTCGATCAG
GTGCGCCAGGGCTGA
>AA QryID: NC_002516_(+)_788894_789142 HitID: NC_015410_(+)_2851324_2851539
rank=1 bitscore=108.0 evalue=1.0E-21 perc_ident=80.28 perc_conserv=91.55 perc_gaps=0.0
query_cvg=86.59 subj_cvg=100.0 alpha=57.69 beta=14 prob=0.9983946374830972
ATGGCTAATGCCACGAATAGTGACGGAATGAGCATGGAACTGGAAGAGCTTAGCCCCAGT
GCCCTGATAGGGCCGCAACAGGATGTGGAGTCCATCGAACGGTGGGCGGAGCGCAACGGC
ATCAGCTACGGCACCGCTCGCGCCTGGGTTTACCGGGGTGTGCTGCCGTCGGTGAAGCTG
GGGAAGCTGCGCATGGTGAATAGCGCGCTGCTGCGTACCTGGCTGTTGGAACAGGAATGG
AGTGCCTGA
>AA QryID: NC_002516_(-)_3281676_3281858 HitID: NC_011770_(+)_2313292_2313474
rank=1 bitscore=104.0 evalue=1.0E-20 perc_ident=98.33 perc_conserv=100.0 perc_gaps=0.0
query_cvg=100.0 subj_cvg=100.0 alpha=82.11 beta=5 prob=0.998394631988478
TTGCTGCGGCAGGCGCGGGACGACTGTCGCGGCGACCGCCTGTTCACTTCCTGCAACCGC
TCCAACCTGCCGATGCGGCGCCTGCTGGAGCGCAAGGGATTCCAGCCGAGCGGGGTGATC
GACAACCTCGACGAAGGTGATCCGGAGCTGGTCTTCGTGCGTTTCCTCGCGCCGTCGCGC
TGA
>AA QryID: NC_002516_(-)_2357593_2357781 HitID: NC_018080_(+)_3230148_3230336
rank=1 bitscore=103.0 evalue=3.0E-20 perc_ident=100.0 perc_conserv=100.0 perc_gaps=0.0
query_cvg=100.0 subj_cvg=100.0 alpha=100.0 beta=5 prob=0.9983946305361526
ATGCAGGCTTTCATGGTTCATTTCTCAGACGCCGGACAACCCGGCCGGACCGTGCTCACC
ACTTTCGCGCCAACCCTCAGCACCAGCGAGGCGCACGTTCGCCTGCAACTCTGCTATCCC
CTGCTGTTCCCGCAGCGTCTGAGCGCGGTGCGGGTCTACCCCCTGCTGCCGGCGGCGGCA

CGCGAGTGA
>AA QryID: NC_002516_(+)_4629605_4629811 HitID: NC_008463_(-)_898045_898251
rank=1 bitscore=103.0 evalue=4.0E-20 perc_ident=94.12 perc_conserv=98.53 perc_gaps=0.0
query_cvg=100.0 subj_cvg=100.0 alpha=72.95 beta=1 prob=0.9983946188644237
TTGGCGCATCATCTCATAAAAATAGCGAACCGCTTCCCAGTACCCACGAATATCAATGGA
TCAGCAATATCCAGATGCTTATCGCGGCATTCGAAAAAACATCGACCAATTCCACTGACA
GAATATCGGCGTCATTTGCCTAGCATGGATATTCCAAGTTCACCCTATCAACTTCCCAGA
TTGACACTCTCGCCGGCAGATCAGTAA
>AA QryID: NC_002516_(+)_5148616_5148867 HitID: NC_018080_(+)_5250026_5250277
rank=1 bitscore=107.0 evalue=3.0E-21 perc_ident=73.49 perc_conserv=73.49 perc_gaps=0.0
query_cvg=100.0 subj_cvg=100.0 alpha=53.95 beta=4 prob=0.9983946068095841
ATGACAGACCGAGAGAAAGCTGGCCAGCCACCCCGGTTTTCGTGGGGCGGGGCGCTTTGC
CGGCGGATGTTCGCCGTCCGATTCCAGGCCGCTACGCTGACGCTGAGCAGCGTACTGGCC
GCCCTTACCGTCTCCGCTACCCTCGTCCTGTCAAAGGCGTTCTGCCTGAGCGAGGCGGCG
ACCATCGCCCCGTGCGAGGTCTGGGTGGGCCTGGCACTGCTCGCGGCCATGGTGCTGCCG
CTCAAGACCTGA
>AA QryID: NC_002516_(+)_2894147_2894377 HitID: NC_017549_(+)_3899151_3899381
rank=1 bitscore=96.3 evalue=5.0E-18 perc_ident=100.0 perc_conserv=100.0 perc_gaps=0.0
query_cvg=100.0 subj_cvg=100.0 alpha=100.0 beta=6 prob=0.998394580369009
ATGGCTGAACCCCAGGACAAGTACACCCGGCGCACAGGCAGGACCTGGGCGGACGACCAG
GCGACCTACAACCGTCTGCGCGAAGAAGCCGACGCCGCTCGCCAGAAGCTGCGCGAAAGC
GGCTACAGCGGCGCCGAGTACGACCAGTTGCGTCAAGCCGCCTTCGATCTCAACCGCAAG
GCCAACCAGTACTGGGAGCAGATGCTCAGCGACCTGCGCCAGGAAGACTGA
>AA QryID: NC_002516_(+)_6110801_6111010 HitID: NC_017549_(+)_6602226_6602435
rank=1 bitscore=100.0 evalue=2.0E-19 perc_ident=85.51 perc_conserv=85.51 perc_gaps=0.0
query_cvg=100.0 subj_cvg=100.0 alpha=85.51 beta=1 prob=0.9983945633464513
ATGGATACCCTTCATCCCTTCTTTTATACCCGGCGCCCCGGCCCAGCCGCGGCGCCACGC
TCACCGGTCGAGAGGGATACCAACTTTCCCTCTCTACACTCTGAGGGACACGGCGACCTG
CGGTGTCCCTTCTTTTTTTCACCGCTCAAGACGGCCCGCAGATCTTCCCCGTTGCCGAGG
TGTTGTGTGGCTGCGCTCACCTGTGGATAA
>AA QryID: NC_002516_(+)_1997037_1997192 HitID: NC_008463_(-)_3637485_3637640
rank=1 bitscore=87.8 evalue=1.0E-15 perc_ident=98.04 perc_conserv=98.04 perc_gaps=0.0
query_cvg=100.0 subj_cvg=100.0 alpha=98.04 beta=24 prob=0.9983943904119121
ATGTCTTCGTCCCGATACCCACAAGAAACAGGGGGAGTCATGAGCGAGCCGAGCAAAACC
CAGGAAAACCGCGACAGCGTGGTCGACGCCTGCGCGGCCTTCGTCCTGATCCTGCTGGTG
GTGGCTACCGCGGTGTTCTGGGTCAGCCACCAGTGA
>AA QryID: NC_002516_(+)_1947994_1948215 HitID: NC_017549_(+)_2879993_2880214
rank=1 bitscore=93.2 evalue=3.0E-17 perc_ident=83.56 perc_conserv=83.56 perc_gaps=0.0
query_cvg=100.0 subj_cvg=100.0 alpha=83.56 beta=9 prob=0.9983943719080577
ATGTCGATGAAATGGACCGAGCAGCGCTTGCGCAAGGCTCTCAAGCAGATGGCGAACAAT
CATGAATCGGCTGCGGTCGAGGTCATGCGCGCCGTCGAGCGGGCGAACGATCCGAAGCTG
GCGCAGCGCCTGCTCGAGGTGATCGAGCAGATGCACCAGGATGCCGATGCGCTGCGCTCC
ATCGACGACGAAATCGCCAGCGGCGTGATCCGTTGCCAATGA
>AA QryID: NC_002516_(-)_4330029_4330187 HitID: NC_008463_(+)_1195465_1195623
rank=1 bitscore=87.4 evalue=2.0E-15 perc_ident=100.0 perc_conserv=100.0 perc_gaps=0.0
query_cvg=100.0 subj_cvg=100.0 alpha=100.0 beta=1 prob=0.998394308303401
ATGGACGCTTCCACGAACGGAGCGATACCGCAAAGGGAAGAATGGAGTGCAACCATCGTA
GAGCTTCAAACTGAGATATTGCAGTTGATTGCCGCATCTGGCCGGCCTATTGCCGTGCAT
TTTCTACCCACTACTGCATCGCTCAAGAAGGTTCTGTAA
>AA QryID: NC_002516_(+)_2893100_2893246 HitID: NC_017549_(+)_3898104_3898250
rank=1 bitscore=84.7 evalue=1.0E-14 perc_ident=100.0 perc_conserv=100.0 perc_gaps=0.0
query_cvg=100.0 subj_cvg=100.0 alpha=71.57 beta=10 prob=0.9983941524140378
ATGCGCTTGCGTTGTTTCTTGCGAGGTTGCCGCTGGGACGAAGGTAGCCTGGTCACGGTA
GGCCCCGACCTGATGCTGCGCCAACGTTGCCGGCGTTGCGGCGCCCATCGCTATCTCAGC
GTCGAGGCGCCGCCCGAGGAAGCCTGA
>AA QryID: NC_002516_(+)_1372752_1372916 HitID: NC_017548_(-)_4181593_4181757
rank=1 bitscore=84.7 evalue=1.0E-14 perc_ident=98.15 perc_conserv=98.15 perc_gaps=0.0
query_cvg=100.0 subj_cvg=100.0 alpha=81.34 beta=2 prob=0.9983940539144691
GTGGCCGGGCATGTGCTCGCCGGCGAACTGGGGATCAGCCTGCGCACCCTCTACCGCGAT
ATCGCCACCCTGCGACAGCAGGGCGCGCACATCGAGGGCGGCGCCGGCACCAGGCGTTGC
TGCGCGAGTGGCGTGAACGGGAAAGCATCGCCGCGCAGTGAGTGA
>AA QryID: NC_002516_(-)_227147_227311 HitID: NC_009656_(-)_295035_295199

rank=1 bitscore=87.0 evalue=3.0E-15 perc_ident=83.33 perc_conserv=87.04 perc_gaps=0.0 query_cvg=100.0 subj_cvg=100.0 alpha=58.55 beta=1 prob=0.9983937461436297
TTGCATGATTGGCAAGGAGGCTTAGCGCCTGAAGTAATTATTTTTGTTATGGAAATAACT
TCTGGAGCTATGGGTTTCGCCCCTATCGCCACAGGCCGCGTGGTTGGCGGGCTACAGCGA
TCCGACGAAAAACCCATGCCTTGCGGGCATGAAAAATATTCCTGA
>AA QryID: NC_002516_(+)_3131246_3131416 HitID: NC_011770_(-)_2472235_2472405
rank=1 bitscore=87.0 evalue=3.0E-15 perc_ident=80.36 perc_conserv=80.36 perc_gaps=0.0 query_cvg=100.0 subj_cvg=100.0 alpha=80.36 beta=7 prob=0.9983936981279575
ATGAGCAAACTGCAACATATCGGCCTGTTCCTGTTCGTTCTTCTTTCCGCCGGCCTGGCG
CTCGGCTACCGAGTCGACTGCCCGGACGGCAGCCATGTCGAGACCTACCTGCTTCGCGGC
CTGGGCGCGCGCCTCCTCGGCAACGATCTCTGCGAGCAGGCAGCGCCCTGA
>AA QryID: NC_002516_(-)_223679_223822 HitID: NC_009656_(-)_291566_291709
rank=1 bitscore=80.5 evalue=2.0E-13 perc_ident=97.87 perc_conserv=97.87 perc_gaps=0.0 query_cvg=100.0 subj_cvg=100.0 alpha=75.76 beta=1 prob=0.998393412815203
ATGACCGGCTATCAGGTCAAGCCGCTACGGCTTCGACGGGAAAATATCCTGCCAGCGCCG
CGGAATGCCGGGGTAATTAATGTTTTTAATACGCTGTATCAGCAATCCCTGATGCCTGGC
CAAAGTAGCAAAGTAAAACTTTGA
>AA QryID: NC_002516_(-)_5885170_5885325 HitID: NC_017549_(-)_6376900_6377031
rank=1 bitscore=75.1 evalue=1.0E-11 perc_ident=100.0 perc_conserv=100.0 perc_gaps=0.0 query_cvg=84.31 subj_cvg=100.0 alpha=67.5 beta=28 prob=0.998391624653196
TTGCCTGGAAGGGGGAGGCAATCGATGAAGCGCAAGCCAGACTTGCTGTGGGTTTTGGTC
ATCATCTTCGGACTTGGGGTCGTCACCACCGGTTATACCCAGAGCCTCTGGGAACGACAG
GCCAGCACGCCCGCCCAGGTGACGCAACAGCCGTAA
>AA QryID: NC_002516_(+)_273398_273553 HitID: NC_018080_(+)_266090_266218
rank=1 bitscore=75.5 evalue=7.0E-12 perc_ident=100.0 perc_conserv=100.0 perc_gaps=0.0 query_cvg=82.35 subj_cvg=100.0 alpha=91.18 beta=1 prob=0.9983905742603055
ATGGACATGAGCGGGTACCCGATTCTTGAAATTGTTATGTCGCGTGGGTTGCGCAGGTGC
CCGGAAAGAAGCGCGGAGCGCTTCCGGCAGATCGGGACGGCCTGCACGGGTGGCAGCGAT
GGTGCGCGATTGGTCGAAAAAGCGTCAATTCGATAA
>AA QryID: NC_002516_(-)_3677199_3677351 HitID: NC_011770_(+)_1925483_1925623
rank=1 bitscore=72.4 evalue=6.0E-11 perc_ident=95.65 perc_conserv=100.0 perc_gaps=0.0 query_cvg=92.0 subj_cvg=100.0 alpha=69.93 beta=2 prob=0.9983880781328388
GTGTGGCTGGGCTTGGCGGATATCGCTGCGCGATATGCGCCCTACGGAGGGGCGGCAATC
CGTTTCGCTGGGCGAGCATCCGAAGTTGAGGAGAACGCCTGGGACCTGGCGAATATCGCC
GACCGATATTTCTCCAGCAGGGGCAATCCCTGA
>AA QryID: NC_002516_(-)_4189594_4189716 HitID: NC_017549_(-)_5253873_5253995
rank=1 bitscore=71.2 evalue=8.0E-11 perc_ident=95.0 perc_conserv=95.0 perc_gaps=0.0 query_cvg=100.0 subj_cvg=100.0 alpha=72.41 beta=1 prob=0.9983879636441977
ATGTGCGGCTCTTTTGTTTTCATCATTGGCGATTTCCGCCGCCCGCTGGCGGAATCCATC
GCCAGGATGTGTCGTGGCCCGTACAGTGGTCGCGTCAACCTCGATTCGCTCGGGGAACCA
TAG
>AA QryID: NC_002516_(-)_630143_630280 HitID: NC_009656_(-)_687843_687992
rank=1 bitscore=72.4 evalue=6.0E-11 perc_ident=95.35 perc_conserv=97.67 perc_gaps=0.0 query_cvg=95.56 subj_cvg=87.76 alpha=46.05 beta=4 prob=0.9983878851821818
GTGGGCCGCAAAACGGATTGGCCGACCAACGAAAAAACCTTCCGCACCAAGCGCGACGCC
GAAGACTGGGCACGCCGCACCGAAGACGAGATGGTACGCGGCGTGTACATCCAGCACTGC
GGCTCCGAGCGCATGTGA
>AA QryID: NC_002516_(+)_53524_53778 HitID: NC_003295_(-)_3446534_3446797
rank=1 bitscore=84.7 evalue=2.0E-14 perc_ident=46.43 perc_conserv=64.29 perc_gaps=0.0 query_cvg=100.0 subj_cvg=96.55 alpha=18.75 beta=2 prob=0.9983874165005105
ATGCCGAAGTACACGTGTACCTGTGGCTACGTTATGAACCTTTCGCAAGGATGGTCAGAC
TATGAGTTGACTCTAATTCCTGAGTCAGCAATAGAGAGCTTGGGGGATAGGCTAGATAGC
GAGGATAAGCTGTCTTCTGATCAGTTATATGAAGCTCTTGATGAAAAGGCCATTACGGTG
TACCGATGCCCGAAGTGTAGAAGGTTGCACTTGGAGGAAGAGCCAAATAGATTCACCACT
TATATAGTTGAGTAA
>AA QryID: NC_002516_(+)_65535_65678 HitID: NC_017549_(+)_70194_70331
rank=1 bitscore=68.6 evalue=8.0E-10 perc_ident=100.0 perc_conserv=100.0 perc_gaps=0.0 query_cvg=95.74 subj_cvg=100.0 alpha=66.91 beta=4 prob=0.9983859428640773
TTGCAAATGAAGAAAGTACTGTTGGCTGTGTTCCTGCTCTGTGGCTTCTCTCTTACCGCC
CAGGCCGCGTGCCCGGTTTTCGGCCCGTCCGAGGACCCGTGCAGCGGGCCGGTGTTCGGT
CCGTCGACCTGCGAATGCCCTTGA
>AA QryID: NC_002516_(+)_5275967_5276098 HitID: NC_017549_(-)_968151_968282

rank=1 bitscore=65.5 evalue=5.0E-9 perc_ident=100.0 perc_conserv=100.0 perc_gaps=0.0
query_cvg=100.0 subj_cvg=100.0 alpha=72.56 beta=2 prob=0.9983815210214557
TTGCAGACGGCCCGCTACAGCGAGCTTGGATTCTTCGCATTCGTCGAACGCAAGTCAAGC
TTTATTCTGAAAATTATCAAAAAAAATTCCAAGGAAGCTGCCATACCCTTCCATAAAAAA
GCTGATCAATGA
>AA QryID: NC_002516_(+)_2096384_2096506 HitID: NC_017548_(-)_3484561_3484683
rank=1 bitscore=66.2 evalue=2.0E-9 perc_ident=95.0 perc_conserv=97.5 perc_gaps=0.0
query_cvg=100.0 subj_cvg=100.0 alpha=74.37 beta=3 prob=0.9983797749568825
TTGATTTTGCAAAACAACAATATGTTGTGGATTCTGGCGGGCGACGCCGAGCCGGCGTTG
ATCTGCGTCAGCGTCCGGCGGATTGCCCGGGTTCCATTGGAGGTATTCCCCGTGCCCGGG
TGA
>AA QryID: NC_002516_(-)_5386132_5386254 HitID: NC_008463_(-)_5656494_5656610
rank=1 bitscore=65.5 evalue=4.0E-9 perc_ident=94.74 perc_conserv=97.37 perc_gaps=0.0
query_cvg=95.0 subj_cvg=100.0 alpha=64.42 beta=4 prob=0.9983751928823636
GTGCGGGTGACCGTGGTGGAACAGACGCTGCCTCTGAGAAACCGCGCCTGCGGCGCTTTC
CAGGAAGTCAGCCTGGAACGACGCGACTGCCGAGCCCATATCGGGCTGCAATTCCGCGAT
TGA
>AA QryID: NC_002516_(-)_2901752_2901982 HitID: NC_018080_(+)_2618632_2618841
rank=1 bitscore=74.7 evalue=1.0E-11 perc_ident=63.77 perc_conserv=65.22 perc_gaps=0.0
query_cvg=90.79 subj_cvg=100.0 alpha=60.83 beta=2 prob=0.9983707819050818
ATGCTTCCACCGAGAAAAGACATGTCCGACACGCTCTCCTTCGCCTCGCGCCTCTGCAAG
TTCGTCATCCTTCTCGTCATCCTGCTGATCGGCGGCGCCAGCCTCTTCCTGTGGTGGCTG
GGGCGGCCTTACCAGGACGCCATGTCCGCCTTCGTCCCGTTGCTCATGCCGATGCTCGTG
CTGGTGCTGGCGTTCGATACGTTCCGGGCCCTCGCCCGAACGAGGCGCTAG
>AA QryID: NC_002516_(-)_1707664_1707852 HitID: NC_008463_(+)_3935669_3935857
rank=1 bitscore=63.9 evalue=3.0E-8 perc_ident=80.65 perc_conserv=80.65 perc_gaps=0.0
query_cvg=100.0 subj_cvg=100.0 alpha=80.65 beta=6 prob=0.9983512658634585
ATGCACCTACCGACGACGCGCACCCTGCCCTACCAGCCTTCCGCCAACCCGGCCGGAACG
CCCGCGCGTCTACCCCGCAAGCACAGCCGCTGGCTGGAACTGGAGCCGCTCTCCCTCCCT
CTCGAACTGGCCTTCTGGGCGCTCTGGCATTGCCGCCACGCGCGTCCGCCGGACGCTCGT
CACGCCTGA
>AA QryID: NC_002516_(-)_1948236_1948364 HitID: NC_018080_(+)_3622014_3622142
rank=1 bitscore=58.9 evalue=4.0E-7 perc_ident=97.62 perc_conserv=97.62 perc_gaps=0.0
query_cvg=100.0 subj_cvg=100.0 alpha=97.62 beta=1 prob=0.9983512042529481
ATGTTCACCGCCTTCGCCTTCCGCGGCCGCCAACCTTATACCGTTCGCCGGAACACAGCC
CAGGGCGCGACCTTGCGCCCGAAACTGGCGAACCGGCCGGATGCGCCGGTTTCAGCCGGG
AAGTCCTGA
>AA QryID: NC_002516_(+)_804381_804560 HitID: NC_009656_(-)_4917419_4917592
rank=1 bitscore=66.6 evalue=4.0E-9 perc_ident=63.16 perc_conserv=70.18 perc_gaps=0.0
query_cvg=96.61 subj_cvg=100.0 alpha=70.71 beta=8 prob=0.9983203164743983
ATGCTGATGGTCATGCGATTGTTCGGGCTGCTACTGGCGGCCTTCCTGATGAGCGGTTGC
AGCGTTCGGGTGCCGGGCGCCTACGTCGAACTGGATCCGTTCGACGATGGCTACTACGGC
TACGATCGCGGCGGCCCGCCCCATTGTCCTCCGGGCCACGCCAAGAAAGGCTGGTGCTGA
>AA QryID: NC_002516_(+)_2165028_2165204 HitID: NC_008463_(-)_3468988_3469164
rank=1 bitscore=63.2 evalue=4.0E-8 perc_ident=68.97 perc_conserv=68.97 perc_gaps=0.0
query_cvg=100.0 subj_cvg=100.0 alpha=68.97 beta=4 prob=0.998302178292285
ATGTCTCGACAACGACAATGGACAGGAGTCGCCGCCATGCGCATCGCCGTCACCCTGGTG
TTGCCCATCCTGCTGATCCTCAGCCTGCTCGGGTTCGATTTCCTCTATTCCCGCCACCTC
GGCCAGGAAAGACCTGCCGCCTGCGCCGTCGGACAGGCCTGTCGGATCGTCCCCTGA
>AA QryID: NC_002516_(+)_3108468_3108644 HitID: NC_018080_(-)_2286808_2286984
rank=1 bitscore=61.2 evalue=2.0E-7 perc_ident=67.24 perc_conserv=67.24 perc_gaps=0.0
query_cvg=100.0 subj_cvg=100.0 alpha=67.24 beta=6 prob=0.9982560450047004
ATGACTCCCGAATCCTTCGTCACATTGCTGATCGTCGCCTGGCTGGGCCTCGCCGGCGTC
ACCCTGTGGGCCTTCCTGCGCGTGCCGCGTCGGCGCGCACGCCTGCAACGCAAGGCACGG
CAAGCCGAAAACCGGCATCCCGCACGGCCGCCACAGCCTCTTCCGCAACGCAGCTGA
>AA QryID: NC_002516_(+)_3581949_3582080 HitID: NC_018080_(-)_1809019_1809150
rank=1 bitscore=52.8 evalue=3.0E-5 perc_ident=95.35 perc_conserv=95.35 perc_gaps=0.0
query_cvg=100.0 subj_cvg=100.0 alpha=55.82 beta=1 prob=0.9982519862585504
ATGCTGCCCACCATCCAGTTCGATCACCAGCATCCGCTCATGACAGACGAAATCGACGAT
GTACGGCCCGAGAACCTTCTGCCGACGAAACTTCAGCCCGAGCAATCGATGCCCGCGCAA
ACACTGCCATAG
>AA QryID: NC_002516_(+)_5456318_5456410 HitID: NC_018080_(+)_5556395_5556487

rank=1 bitscore=48.9 evalue=3.0E-4 perc_ident=100.0 perc_conserv=100.0 perc_gaps=0.0
query_cvg=100.0 subj_cvg=100.0 alpha=74.24 beta=3 prob=0.9981877376261483
TTGCTCTGGAGCCTCGACGAAGAGATCCAGGCCGCAGTGCGCACCCCGCCGTCCACCGGA
CGGATGCCCGACGACAAGCACGACGCCTCCTGA
>AA QryID: NC_002516_(+)_4327194_4327286 HitID: NC_008463_(-)_1198334_1198426
rank=1 bitscore=48.9 evalue=4.0E-4 perc_ident=100.0 perc_conserv=100.0 perc_gaps=0.0
query_cvg=100.0 subj_cvg=100.0 alpha=59.77 beta=1 prob=0.9981786616795769
GTGATCGGATCGTTTTTAATCGATTCTCCGCTTTCAAATTCGCCGGGGGCTGAAGTCGGC
CAGAAAGACATGGCTACTTCAGACCATCCCTAA
>AA QryID: NC_002516_(+)_384538_384657 HitID: NC_017549_(-)_6246575_6246694
rank=1 bitscore=43.9 evalue=0.011 perc_ident=92.31 perc_conserv=94.87 perc_gaps=0.0
query_cvg=100.0 subj_cvg=100.0 alpha=92.31 beta=2 prob=0.9976434215564912
GTGCCCGGGGCGGATAACGTCGCAGGCGTCATCCGCTCCGGGCAGCCGGCTTTCCGCTCC
AGGGCGTGCATGGCTTGCGCCGGTCGCGCCGAACTGGCGATGCAGGCTCCGTCGCCGTAG
>AA QryID: NC_002516_(+)_3147602_3147733 HitID: NC_009656_(-)_2472960_2473091
rank=1 bitscore=44.7 evalue=0.008 perc_ident=79.07 perc_conserv=88.37 perc_gaps=0.0
query_cvg=100.0 subj_cvg=100.0 alpha=84.88 beta=2 prob=0.996981992595106
TTGATACAGCAGCCCATCGCAGTTCAACGAGGAAACCCGATGAAACTCAGCATTGTCGCC
CTGGGCCTGTTGTCCATGGCAGGGGTGCTCATCTGCACCTATCTGATCCTGAAATCGCTC
AGCGCCATCTGA
>AA QryID: NC_002516_(+)_2028103_2028312 HitID: NC_017548_(-)_3551453_3551662
rank=1 bitscore=46.6 evalue=0.004 perc_ident=52.17 perc_conserv=52.17 perc_gaps=0.0
query_cvg=100.0 subj_cvg=100.0 alpha=52.17 beta=6 prob=0.9927478370215476
ATGGCTTCCGCTCCCGACGCTCTTCCCCAGGCCCTTTCCTCCGACGACGCTCCGGCCCAG
GCCGCGCCGAGCGACGCCAGCGCGTTGCTGAAGGCCGCCCTGGCGAAGAAGAACCAGGCC
CAGCTCTATCCGCAGCGGAAGATCCACGACAAGGGCCAGCGTCCCGGCATGGCGCCGCGC
GGTACCCGTCGTTCGATGGGCAAGCGCTGA
>AA QryID: NC_002516_(+)_2562140_2562283 HitID: NC_011770_(-)_3283651_3283794
rank=1 bitscore=40.0 evalue=0.35 perc_ident=61.7 perc_conserv=61.7 perc_gaps=0.0
query_cvg=100.0 subj_cvg=100.0 alpha=61.7 beta=5 prob=0.9858855104649109
ATGACCGAACCAATGCAACGCCCCGACCACGAAGCCTCGCGCTGGCCGCGCGACTTCGAT
ATCCGCCAGCGCTATGCCGAGCAGCAGGAACTGCTTGAGCCACCGCCGGCGCGGCCCCGC
GAAGAGCCTACCGCCGACGCCTGA
>AA QryID: NC_002516_(-)_1215856_1216011 HitID: NC_008463_(+)_4433921_4434076
rank=1 bitscore=37.4 evalue=2.4 perc_ident=66.67 perc_conserv=66.67 perc_gaps=0.0
query_cvg=100.0 subj_cvg=100.0 alpha=66.67 beta=2 prob=0.9820178653152509
GTGCTGCCTGGGGAGGTGAACGCCATGAGCAAGGGACTGAACGCGAAGAAGGAAACTCGT
AGGAAACCGCTGAAAACCGCCCAGGAGAAACGCCTGGCGAAACGAGCGAAAAAACACGCT
CCGGCGGCGCCGCTCGGCACCCCATCGCGGAGCTGA
>AA QryID: NC_002516_(-)_2591937_2592074 HitID: NC_017549_(-)_3590224_3590361
rank=1 bitscore=37.4 evalue=2.0 perc_ident=62.22 perc_conserv=62.22 perc_gaps=0.0
query_cvg=100.0 subj_cvg=100.0 alpha=62.22 beta=1 prob=0.9754033928963171
TTGCAGCGTGGTGGTTCCGCCAGCGGCATCGGTGTGGCGCGGCTCGCGGGTTCGGTGGTG
CGGGTGGAACGCCTGGAGGAGGGAGGGGAAACAGGCGGTAACGACATGCTCGCTGGGCAC
CTCTGGCAGTTCGGATAG
>AA QryID: NC_002516_(-)_3547362_3547658 HitID: NC_008463_(+)_2026291_2026590
rank=1 bitscore=47.4 evalue=0.003 perc_ident=35.96 perc_conserv=60.67 perc_gaps=0.0
query_cvg=90.82 subj_cvg=89.9 alpha=37.46 beta=16 prob=0.8021662375803766
ATGCTTTGGGTCAAGCGTACGTTAATGGCGGTGGGGCTGTTAGTTGTCGCCCTTTTCATG
ATTGTGGTTGCTTTGGAGAACCGGCAAAGCGTCAGCTTTGAACTCTTTGGTCTTGCCACG
CCAGATTTACCTGTGGTCCTTTATGTTGCGTTAGCATTTATTGCTGGCGGTATTATTGGT
ATGTTGATCAGCGTGCCTCTTCTGGCTCGTGCCAAAGTGCGTCTCAGATCTGCAAGATCT
GATCTGTCTCGTACTCGAAAAGAACTCGCAGTATCTCAGTCTACCGCCCTGCGGTGA

- ***Staphylococcus aureus* MRSA**

vsORFs

>AA QryID: NC_002952_(+)_1155248_1155337 HitID: NC_010079_(+)_1144638_1144736
rank=1 bitscore=99.0 evalue=2.0E-19 perc_ident=100.0 perc_conserv=100.0 perc_gaps=0.0
query_cvg=100.0 subj_cvg=90.62 alpha=41.0 beta=1 prob=0.9998780190084814

TTGAAGAAGAAAAGAATTAATAATGATATTTTGGGGCGTATGATTTATAGTAGTAGTATC
GATAAAAGAAATTTATACAATTTGAAATAA
>AA QryID: NC_002952_(+)_1161388_1161465 HitID: NC_009641_(+)_1156561_1156632
rank=1 bitscore=75.3 evalue=3.0E-12 perc_ident=95.65 perc_conserv=100.0 perc_gaps=0.0
query_cvg=92.0 subj_cvg=100.0 alpha=56.07 beta=1 prob=0.9997547318364819
GTGTTGTTGTTTTATCATAATTGCAATATAACAATATTAGTATCAAATTCAGACAAACTA
TCCAGTCATATTTTATAA
>AA QryID: NC_002952_(-)_477891_477953 HitID: NC_017331_(-)_546729_546791
rank=1 bitscore=67.2 evalue=5.0E-10 perc_ident=100.0 perc_conserv=100.0 perc_gaps=0.0
query_cvg=100.0 subj_cvg=100.0 alpha=50.39 beta=4 prob=0.9991718627291469
ATGGCTATTGTAGGTACTATCATTAAAATCATCAAAGCAATTATCGACATTTTCGCAAAA
TAA
>AA QryID: NC_002952_(-)_478234_478299 HitID: NC_010079_(-)_476891_476956
rank=1 bitscore=69.4 evalue=1.0E-10 perc_ident=100.0 perc_conserv=100.0 perc_gaps=0.0
query_cvg=100.0 subj_cvg=100.0 alpha=58.53 beta=10 prob=0.9990956616761997
ATGGGTATCATCGCTGGCATCATTAAAGTTATCAAAAGCTTAATCGAACAATTCACTGGT
AAATAA
>AA QryID: NC_002952_(-)_478137_478202 HitID: NC_016941_(-)_448534_448599
rank=1 bitscore=69.8 evalue=8.0E-11 perc_ident=100.0 perc_conserv=100.0 perc_gaps=0.0
query_cvg=100.0 subj_cvg=100.0 alpha=65.55 beta=12 prob=0.9988359854312834
ATGGGTATCATTGCAGGAATCATTAAATTCATTAAAGGATTAATTGAGAAATTCACTGGT
AAGTAA
>AA QryID: NC_002952_(-)_2597265_2597324 HitID: NC_010079_(-)_2556381_2556440
rank=1 bitscore=62.1 evalue=2.0E-8 perc_ident=94.74 perc_conserv=100.0 perc_gaps=0.0
query_cvg=100.0 subj_cvg=100.0 alpha=37.32 beta=1 prob=0.9979557729951907
ATGGCGTATAAGGATGAGGGGAAAGAGACTAAATTTGCTGTGAAAGGGTATAAAAACTGA
>AA QryID: NC_002952_(+)_848945_849013 HitID: NC_017337_(-)_1181495_1181617
rank=1 bitscore=60.4 evalue=8.0E-8 perc_ident=50.0 perc_conserv=52.5 perc_gaps=45.0
query_cvg=100.0 subj_cvg=100.0 alpha=70.45 beta=2 prob=0.9152043997811837
TTGTCTGTAGAAATTGGGAATCCAATTTCTCTTTGTTGGGGCCCACACCCCAACTTGCAT
TGCCTGTAG

sORFs

>AA QryID: NC_002952_(+)_1485992_1486285 HitID: NC_017342_(-)_1899188_1899481
rank=1 bitscore=198.0 evalue=1.0E-48 perc_ident=100.0 perc_conserv=100.0 perc_gaps=0.0
query_cvg=100.0 subj_cvg=100.0 alpha=100.0 beta=68 prob=0.999795393128471
TTGGGAGGTGAATTCCACAGAAATGATAAAGAACCCCTGATTATTTGTTTTATAGTCGTT
CTTGGGAGTGGGACAGAAATGATATTTTCGCAAAATTTATTTCGTCGTCCCACCCCAACT
TGCATTGTCTGTAGAAATTGGGAATCCAATTTCTCTTTGTTGGGGCCCCTGACTAGAATT
GAAAAAAGCTTGTTACAAGCGCATTTTCGTTCAGTCAACTACAGCCAATATAACTTCGTA
AAGCATAGAACATTGATTTATGTCCCAGCCTCACTTAAAAAATTGATTCTATGA
>AA QryID: NC_002952_(-)_2550333_2550530 HitID: NC_017342_(+)_877983_878171
rank=1 bitscore=122.0 evalue=7.0E-26 perc_ident=98.39 perc_conserv=100.0 perc_gaps=0.0
query_cvg=95.38 subj_cvg=100.0 alpha=96.12 beta=47 prob=0.9997953929761485
TTGTCTATATTGGGGAGTGGGACAGAAATGATATTTTCGAAAAAATTTATTTCGTCGTCCC
GCCCCGGCAAGGTTGACTAGAATTGAAAAAAGCTTGTTACAAGCGTATTTTCGTTCAGTC
AACTACTGCCAATATAACTTCGTAGAGCATGGAACATTGATTTATTTCCCAGGCTCTTTA
AATAACATTGACCTCTAA
>AA QryID: NC_002952_(+)_2258969_2259130 HitID: NC_017342_(-)_1162137_1162298
rank=1 bitscore=95.9 evalue=5.0E-18 perc_ident=100.0 perc_conserv=100.0 perc_gaps=0.0
query_cvg=100.0 subj_cvg=100.0 alpha=100.0 beta=53 prob=0.9997953929407764
TTGACGGGGCCCCAACATAAAGAAATTGGATTCCCAATTTCTACAGACAATGCAAGTTGG
GGGCGGGGCCCCAACACAAAAGCTGGCGGAAAGTCAGCTTACATTAATGTGCAAGTTGGG
GTGGGCCCCAACATAAAGAAATACTTTTTCTTTAGAAATTAG
>AA QryID: NC_002952_(-)_1108481_1108666 HitID: NC_017342_(+)_2262011_2262196
rank=1 bitscore=113.0 evalue=3.0E-23 perc_ident=100.0 perc_conserv=100.0 perc_gaps=0.0
query_cvg=100.0 subj_cvg=100.0 alpha=100.0 beta=46 prob=0.9997953928270451
TTGACTGAACGAAAATGCGCTTGTAACAAGCTTTTTTCAATTCTAGTCAGGGGCCCCAAC
AAAGAAGCTGACGAAAAGTCAGCTTACAATAATGTGCAAGTTGGGGATGGGCCCCAACAC
AGAGAATTTCGAAAAGAAATTCTACAGGCAATGCAAGTTGGGGTGGGACGACGAAATAAA

TTTTGA
>AA QryID: NC_002952_(-)_2459136_2459363 HitID: NC_017673_(-)_2274236_2274475
rank=1 bitscore=134.0 evalue=2.0E-29 perc_ident=96.0 perc_conserv=98.67 perc_gaps=0.0
query_cvg=100.0 subj_cvg=94.94 alpha=93.57 beta=45 prob=0.9997953926361236
TTGTTGGGGCCCCTGACTAGAGTTGAAAAAAGCTTGTTGCAAGCGCATTTTCGTTCAGTC
AACTACTGCCAATATAATATTATAGACCCTAGGACATTGATTTATGTCCCAAGCTCCCAC
ATCATTGTTACAAATGTACATTATGTAAATGAATATTGCTGTCTAAATGTGCATGTAATA
TACAATGGTGCAGATAATACACTTAAGTCTTTAAAAATGAAACGTTAG
>AA QryID: NC_002952_(-)_848893_849096 HitID: NC_017342_(+)_2539118_2539321
rank=1 bitscore=129.0 evalue=4.0E-28 perc_ident=100.0 perc_conserv=100.0 perc_gaps=0.0
query_cvg=100.0 subj_cvg=100.0 alpha=100.0 beta=37 prob=0.9997953923778524
TTGACTGAACGAAAATGCGCTTGTAACAAGCTTTTTTCAATTCTAGTCAGGGGCCCCAAC
AAAGAGAATTTCGAAAAGAAATTCTACAGGCAATGCAAGTTGGGGTGTGGGCCCCAACAA
AGAGAAATTGGATTCCCAATTTCTACAGACAATGCAAGTTGGGGTGGGACGACGATAAAG
AAATACTTTTTCTATAGAAATTAG
>AA QryID: NC_002952_(+)_309173_309382 HitID: NC_017342_(-)_241381_241590
rank=1 bitscore=125.0 evalue=9.0E-27 perc_ident=100.0 perc_conserv=100.0 perc_gaps=0.0
query_cvg=100.0 subj_cvg=100.0 alpha=100.0 beta=36 prob=0.9997953920882197
ATGGTAGGCCCTAAAGTGTTATATTGGCGGGCCCCCAACATAGAAGCTGGCGGAAATACA
GCTTACAATAATGTGCGAGTTGGCAGGCCCCCAACACAGAGAAATTGGAACACCAATTTC
AACAGGCAATGCAAGTTGGCGGGGCCCCAACATAGAGAATTTCGAAAAGAAATTCTACAG
GCAATGCAAGTTGGGGAAGGACAGAAATAA
>AA QryID: NC_002952_(-)_862188_862349 HitID: NC_017342_(+)_2525866_2526027
rank=1 bitscore=102.0 evalue=5.0E-20 perc_ident=100.0 perc_conserv=100.0 perc_gaps=0.0
query_cvg=100.0 subj_cvg=100.0 alpha=100.0 beta=32 prob=0.9997953879790339
GTGCAAGTTGGGGTGGGGCCCCAACACAGAGAATTTCGAAAAGAAATTCTACAGGCAATG
CGAGTTGGGGGGTGGGCCCCAACACAGAAGCTGACTTTCCGCCAGCTTACAATAATGTGC
AAGTTGGGGCGGGACAACGATAAAGAAATACTTTTTCTATAG
>AA QryID: NC_002952_(-)_446300_446425 HitID: NC_016912_(+)_810455_810601
rank=1 bitscore=71.6 evalue=5.0E-11 perc_ident=92.5 perc_conserv=95.0 perc_gaps=0.0
query_cvg=97.56 subj_cvg=83.33 alpha=90.24 beta=47 prob=0.999795387735996
TTGTTGGGGTCCCTGACTAGAATTGAAAAAAGCTTGTTACAAGTGCATTTTCGTTCAGTC
AACTACTGCCAATATAACTTCGTAGAGCATAGAACATTGATTTATGTCCCAGCCTGCTAT
ATATAA
>AA QryID: NC_002952_(-)_2425158_2425277 HitID: NC_017342_(+)_1001390_1001509
rank=1 bitscore=70.5 evalue=1.0E-10 perc_ident=100.0 perc_conserv=100.0 perc_gaps=0.0
query_cvg=100.0 subj_cvg=100.0 alpha=81.5 beta=53 prob=0.9997953869030467
ATGCCAAATTTAATAGAAATGATCGTTTTAAATATTTTTACAAATTGGCGGGGCCCCAAC
ATAGAAGGTGACGAAAAGTCAGCTTACAATAATGTGCAAGTTGGCGGGGCCCCAACATAG
>AA QryID: NC_002952_(-)_2259140_2259286 HitID: NC_017673_(-)_2361270_2361437
rank=1 bitscore=89.4 evalue=5.0E-16 perc_ident=91.67 perc_conserv=93.75 perc_gaps=0.0
query_cvg=100.0 subj_cvg=87.27 alpha=85.83 beta=48 prob=0.9997953865281508
TTGGGAATACAATTTCTCTTTGTTGGGGCCCCACCGGCAAGGTTGACTAGAATTGAAAAA
AGCTTGTTACAAGCGCATTTTCGTTCAGTCAACTACTGCCAATATAACTTCGTAGAGCAT
AGAACATTGATTTATGTCCCAGCCTGA
>AA QryID: NC_002952_(+)_1108470_1108583 HitID: NC_016912_(+)_1555280_1555390
rank=1 bitscore=56.6 evalue=2.0E-6 perc_ident=97.22 perc_conserv=100.0 perc_gaps=0.0
query_cvg=97.3 subj_cvg=100.0 alpha=95.91 beta=33 prob=0.9997953605847414
ATGATATTTTCTCAAAATTTATTTCGTCGTCCCACCCCAACTTGCATTGCCTGTAGAATT
TCTTTTCGAAATTCTCTGTGTTGGGGCCCATCCCCAACTTGCACATTATTGTAA
>AA QryID: NC_002952_(+)_552305_552478 HitID: NC_017342_(-)_12633_12806
rank=1 bitscore=103.0 evalue=2.0E-20 perc_ident=100.0 perc_conserv=100.0 perc_gaps=0.0
query_cvg=100.0 subj_cvg=100.0 alpha=100.0 beta=19 prob=0.9997953435702767
TTGACTGAACGAAAAAGCGCTAGTAACAAGCTTTTTTCAATTCTAGTCAGGCGCCCCAAC
ACAGAGAATTTCGAAAAGAAATTCTACAGGCAATGCGAGTTGGGGTGGGACGACGAAATA
AATTTTTTGAAAATATCATTTCTGTCCCACTCCCATTATGGCACGAATAATTAA
>AA QryID: NC_002952_(+)_2578473_2578577 HitID: NC_009487_(+)_2574517_2574621
rank=1 bitscore=39.3 evalue=0.33 perc_ident=100.0 perc_conserv=100.0 perc_gaps=0.0
query_cvg=100.0 subj_cvg=100.0 alpha=100.0 beta=27 prob=0.9997952448227276
ATGAAATTAGATTTACAGACTGCTCGTCGTAATTTAAATAGCCCTAACATTAAAACAAGA
AAGCGTGCTTTAAAGATTATTAAGCAACACAAAAGAGCGAAATAA
>AA QryID: NC_002952_(+)_2249533_2249832 HitID: NC_017342_(-)_1171435_1171734

rank=1 bitscore=191.0 evalue=2.0E-46 perc_ident=100.0 perc_conserv=100.0 perc_gaps=0.0
query_cvg=100.0 subj_cvg=100.0 alpha=88.08 beta=1 prob=0.9997951833454506
ATGATGTGGCGTTGCGGTGTTATGGGTGGCATAGCCCGTGGTATTGATGCCATGCCGTTA
CAATTCTATGATATGTCGTTGGGACGTTGCAATGTGTATTATGCCGTTGTGACGTTATTA
TTTCACACTGTTACATGTATTTGTGAATTGCTGTGGAAATGTTGCGACATATACTGCTAC
ACTGATGAATCATTGTGTCAAGATGACATTGCAATGAAGAATAGCAACTCTGTTATTAAC
CACTTTTTACATGGTGAAATCTCGTTAATATTATTTCAAATAAAAACAGCAGTAGGATGA
>AA QryID: NC_002952_(-)_2425055_2425210 HitID: NC_017342_(+)_1001539_1001727
rank=1 bitscore=76.3 evalue=4.0E-12 perc_ident=86.0 perc_conserv=90.0 perc_gaps=0.0
query_cvg=98.04 subj_cvg=80.65 alpha=84.75 beta=32 prob=0.9997951800697111
GTGACGAAAAGTCAGCTTACAATAATGTGCAAGTTGGCGGGGCCCCAACATAGAGAATTT
CAAAAAGAAATTCTACAGACAATGCAAGTTGGGGAACAACGAAATAAATTTTATGAAAAT
ATCATTTCTATCCCACTCTTAAGAATCACTACATAA
>AA QryID: NC_002952_(-)_2459344_2459496 HitID: NC_009641_(+)_1060677_1060832
rank=1 bitscore=72.4 evalue=7.0E-11 perc_ident=85.11 perc_conserv=87.23 perc_gaps=0.0
query_cvg=94.0 subj_cvg=92.16 alpha=81.2 beta=31 prob=0.9997951502127873
TTGGGAATCCAATTTCTCTTTGTTGGGGCCCCGCCAACTTGCATTGTCTGTAGAAATTGG
GAATCCAATTTCTCTTTGTTGGGACCCACACCCCAACTCGCATTGCCTGTAGAATTTCTT
TTCGAAATTCTCTTTGTTGGGGCCCCTGACTAG
>AA QryID: NC_002952_(-)_2105975_2106148 HitID: NC_018608_(-)_2016727_2016900
rank=1 bitscore=74.7 evalue=1.0E-11 perc_ident=96.49 perc_conserv=98.25 perc_gaps=0.0
query_cvg=100.0 subj_cvg=100.0 alpha=100.0 beta=14 prob=0.999794948023114
TTGAATGTTGTGGCCGTTAATTTTAAATGGTCTTTCGAATTATATATGTTGAAAGTTGAA
AATAGAGCGATGAATCGTGTACATAATAATATTTATAACTTTAATCATAACGAAAAAGGT
AGGAAGAAAACAAAAATTTATACTCAACATCGCAAATATTTCAAGAAAATGTAA
>AA QryID: NC_002952_(-)_2720942_2721109 HitID: NC_009641_(-)_2699302_2699469
rank=1 bitscore=86.3 evalue=4.0E-15 perc_ident=94.55 perc_conserv=96.36 perc_gaps=0.0
query_cvg=100.0 subj_cvg=100.0 alpha=94.55 beta=16 prob=0.999794911467055
GTGAGATACGTTATTTCAATAATAATGGGAATTGTATTAGCAATTTGGTCATTTAAACAA
CTGAGTCAAAGTCATTTAGATAGCGGATTTATGTTTTTCTTCATAGTTTATGTGTTGTAT
ATCAGTTATTTTAATAGCGATAAGCACGATAAAAATAAAAAACGATAG
>AA QryID: NC_002952_(-)_2858676_2858822 HitID: NC_017347_(-)_2871022_2871168
rank=1 bitscore=71.2 evalue=1.0E-10 perc_ident=100.0 perc_conserv=100.0 perc_gaps=0.0
query_cvg=100.0 subj_cvg=100.0 alpha=100.0 beta=12 prob=0.9997947909389737
TTGAAAAGTGAATTGAGCAACTCAGGAATAGATGTCACTGTTAAAGATGTTGAAAAGTAT
ATGAATCGATATAATGAAGTTATGAAGGGAAAAATGGCGAAAAAGCTAAAAAGTTATGTT
TGTCGTTACTACCTATTAATATCATAG
>AA QryID: NC_002952_(+)_2002762_2002950 HitID: NC_017342_(-)_1395727_1395915
rank=1 bitscore=92.0 evalue=9.0E-17 perc_ident=100.0 perc_conserv=100.0 perc_gaps=0.0
query_cvg=100.0 subj_cvg=100.0 alpha=100.0 beta=5 prob=0.9997944746777108
TTGTCATCATTTCAATATCATTCAATAATAGCTAAACCTTCTTCTTCGATTCAGTTTCAT
GAAATAATTCCCGCTGAAAGTATCATGATTTACACATTACTTCCACCATTATATAACTTA
AAAATGACTATATTTCATCAAACATTATCTAAAGGCGTCGCACCTACACCAACTCCATCC
AACAATTAA
>AA QryID: NC_002952_(+)_2255218_2255391 HitID: NC_018608_(+)_2129582_2129722
rank=1 bitscore=54.3 evalue=2.0E-5 perc_ident=97.83 perc_conserv=97.83 perc_gaps=0.0
query_cvg=80.7 subj_cvg=100.0 alpha=88.39 beta=2 prob=0.9997944415973425
GTGATTACAACGGAATCCTCCATAATTTCAGATATGATTCTATCGAGAATTGTCATGACA
GTAAAAATGTTTTTAATTTGCAATTTTTTATGTAATCGTTTTGCAATCTATGTCTATTTT
CTATTAGAAAAACTACACAAAAAGACGACCTACACAACAGTACTAGTCATGTAA
>AA QryID: NC_002952_(+)_2742195_2742308 HitID: NC_017341_(+)_2744500_2744613
rank=1 bitscore=48.5 evalue=5.0E-4 perc_ident=100.0 perc_conserv=100.0 perc_gaps=0.0
query_cvg=100.0 subj_cvg=100.0 alpha=100.0 beta=14 prob=0.9997943589326637
ATGAAAAATAGTATATCACAAAATTCTGAATTTTTAGATAAAGAAATTGGCAATTTTTCA
ATCATATTGTTACAATACACTTTTTATTTTATCTTCATTTTTAAAATCCATTAA
>AA QryID: NC_002952_(-)_2896396_2896557 HitID: NC_018608_(+)_818547_818723
rank=1 bitscore=96.3 evalue=4.0E-18 perc_ident=90.57 perc_conserv=96.23 perc_gaps=0.0
query_cvg=100.0 subj_cvg=91.38 alpha=86.66 beta=17 prob=0.999794275182617
TTGACTGAACGAAAATGCGCTTGTAACAAGCTTTTTTCAATTAAAGTCAGGGGCCCCAAC
ACAGAGAATTTCGAAAAGAAATTCTACAGGCAATGCGAGTTGGGGTGGGACGACGAAATA
AATTTTGCGAAAATATCATTTCTGTCCCACTCCCTAATTTGA
>AA QryID: NC_002952_(-)_1100299_1100490 HitID: NC_018608_(-)_1108918_1109121

rank=1 bitscore=89.0 evalue=8.0E-16 perc_ident=98.41 perc_conserv=98.41 perc_gaps=0.0
query_cvg=100.0 subj_cvg=94.03 alpha=100.0 beta=5 prob=0.9997942185335835
ATGGATAACTTTTCAACTATTTTATTTGCACTAAAACTTTGTATACAATTTTCAATACTT
AATTCACATTACCTGAATCAATCAAATCTAGTTACAAAAAAAGTACTATCCATAAACTTC
ATTGACCTGTATCTAAGTAAGTTAGAGATATATCTAACTTATAGCGTACACATCAAAATG
TTTAATAGATAG
>AA QryID: NC_002952_(+)_2783143_2783304 HitID: NC_017342_(-)_640802_640963
rank=1 bitscore=62.8 evalue=5.0E-8 perc_ident=100.0 perc_conserv=100.0 perc_gaps=0.0
query_cvg=100.0 subj_cvg=100.0 alpha=100.0 beta=9 prob=0.9997939640120137
TTGGCAATTATTGAATATTTATATAATTTTTCTCGTAATAAACAAAACTTTAATAGCGCT
AAAACAACTGTGTTAAGTTATGATTTAACGAATTTAACAAATTTTACTAGGATGGCATTT
AAGAATATTTATACGTTATTAACGAATATTTATTTATTGTAA
>AA QryID: NC_002952_(+)_2459408_2459593 HitID: NC_017342_(-)_967122_967343
rank=1 bitscore=89.4 evalue=5.0E-16 perc_ident=77.05 perc_conserv=83.61 perc_gaps=1.64
query_cvg=98.36 subj_cvg=83.56 alpha=70.72 beta=32 prob=0.9997937534311372
GTGGGTCCCAACAAAGAGAAATTGGATTCCCAATTTCTACAGACAATGCAAGTTGGCGGG
GCCCCAACAAAGAGAAATTGGATTCCCAATTTCTACAGACAATGCAAGTTGGCGGGGCCC
CAACAAAGAGAAATTGGATTCCCAATTTCTACAGACAATGCAAGTTGGGGTGGGACAACG
AAATAA
>AA QryID: NC_002952_(-)_2650144_2650299 HitID: NC_017342_(+)_777199_777354
rank=1 bitscore=67.8 evalue=1.0E-9 perc_ident=100.0 perc_conserv=100.0 perc_gaps=0.0
query_cvg=100.0 subj_cvg=100.0 alpha=100.0 beta=7 prob=0.9997937196801765
ATGTCTAAAGAACATCTACATTTTATCATATTTTCTGACTTATTAAACTTTTATATAATT
AACTATTTCTTAATTTTCCAAAATAGTGATAAATTTGTGAAATGCATCACAAACCCCTTT
ATTTATTTAGAAATTCATGTAATATTAGACTTGTAA
>AA QryID: NC_002952_(-)_2787832_2787945 HitID: NC_017341_(-)_2810055_2810168
rank=1 bitscore=58.2 evalue=7.0E-7 perc_ident=100.0 perc_conserv=100.0 perc_gaps=0.0
query_cvg=100.0 subj_cvg=100.0 alpha=100.0 beta=9 prob=0.9997936794852738
ATGGGTGGCTTTGTGAATAGTCTAATAATGAAGGATTTAAGCGATAATGATATGCGTTTT
AAATATGAATATTACAATAGAGAAAAAATACGTAGAACAAACTTAATAAAATAG
>AA QryID: NC_002952_(+)_2493206_2493394 HitID: NC_009632_(+)_2495221_2495409
rank=1 bitscore=80.1 evalue=3.0E-13 perc_ident=100.0 perc_conserv=100.0 perc_gaps=0.0
query_cvg=100.0 subj_cvg=100.0 alpha=100.0 beta=4 prob=0.9997936386212469
ATGAACTTTTCAAAACAAAAAGGAATCAACTTACACATCGTTTGTATAATAGTCTTATCT
ATAACTACTGATGCTATTGATTCCTTTTTTATGTTATATATTTTCTTTATTTCAAACTGT
ATATTAATGATGTCACTTCATTTGATACGATTCTTGATAACCTATTCAAAATTCCGCCAA
ATAACATAA
>AA QryID: NC_002952_(+)_536115_536246 HitID: NC_017347_(+)_542377_542508
rank=1 bitscore=60.5 evalue=1.0E-7 perc_ident=100.0 perc_conserv=100.0 perc_gaps=0.0
query_cvg=100.0 subj_cvg=100.0 alpha=100.0 beta=8 prob=0.9997935240770173
TTGCGTTTGAATGATGCTCGTATTTTTGAAGTAAGGAAAAAGTTGTTTTTAAAATTACAA
CGAATTAAAAACAATGCCTTTTATATGTTGAAAGAGTATTGCAGATTAAATTATAATAAT
GACGAAGTGTAA
>AA QryID: NC_002952_(-)_347415_347588 HitID: NC_017337_(-)_305014_305187
rank=1 bitscore=91.3 evalue=1.0E-16 perc_ident=100.0 perc_conserv=100.0 perc_gaps=0.0
query_cvg=100.0 subj_cvg=100.0 alpha=100.0 beta=1 prob=0.9997934719966166
ATGCACGTACGTCGTGTTGCGCTCTGTAATGTTATACATACACTTATCCTTCATTATACC
CGAACTTTTTATATTAAAAGCAAATTTATGGAAAATGCAATTAATTGTCTATTATTTTTG
TACGGTACATTTAAAATTAAGGATCAATTTAAAAACGCCTACATATACCTTTAA
>AA QryID: NC_002952_(-)_2791470_2791730 HitID: NC_018608_(-)_2673518_2673778
rank=1 bitscore=141.0 evalue=1.0E-31 perc_ident=98.84 perc_conserv=98.84 perc_gaps=0.0
query_cvg=100.0 subj_cvg=100.0 alpha=75.81 beta=9 prob=0.9997931122957144
ATGATTATAATGAGGGGTTATAACGAAAGATATTGTAGCTGGACTAATACAGCTATACAA
TGTGATTATTTTAATAAAGTAAGACAGGTGAAAAAAGACGGTAAATCGGATAGGTTGAGC
AACAACATAAACTCGAACATTATTTATCGTGTGTTTGAAAAATGTATCACGCTATATAAT
GTGTATTCGCGGTGTAAACGTGCACTTATTTTATCAGAACATTCTAAAAAGAACATATTC
AATATAAAAAAGGCTGTGTAG
>AA QryID: NC_002952_(+)_2896419_2896511 HitID: NC_017340_(+)_773775_773885
rank=1 bitscore=53.5 evalue=2.0E-5 perc_ident=96.67 perc_conserv=96.67 perc_gaps=0.0
query_cvg=100.0 subj_cvg=83.33 alpha=88.61 beta=18 prob=0.9997924909202303
ATGATATTTTCGCAAAATTTATTTCGTCGTCCCACCCCAACTCGCATTGCCTGTAGAATT
TCTTTTCGAAATTCTCTGTGTTGGGGCCCCTGA

```
>AA QryID: NC_002952_(+)_929980_930090 HitID: NC_013450_(+)_907606_907713
rank=1 bitscore=43.9 evalue=0.015 perc_ident=100.0 perc_conserv=100.0 perc_gaps=0.0
query_cvg=97.22 subj_cvg=100.0 alpha=100.0 beta=6 prob=0.9997924284774005
GTGATGACAACCAATTACTATGTTGAATCTATAAAATTAAAATTGAATTTCATTATGAAT
ATCGATATAATGAATTGTAAAAAACAAATTTTAAAACGTATTTTATATTGA
>AA QryID: NC_002952_(-)_2736938_2737084 HitID: NC_018608_(-)_2618798_2618944
rank=1 bitscore=80.9 evalue=2.0E-13 perc_ident=93.75 perc_conserv=95.83 perc_gaps=0.0
query_cvg=100.0 subj_cvg=100.0 alpha=97.92 beta=4 prob=0.9997921685147195
ATGTTAACGGTGCAGAAAAAGTGTTGTAAACATTCTAATTGGTATATTACATTCAATGAT
GCTTTGTTAGGAACAGATTACATTATGATAACAAAGACCACAAGGTACCTAATCTCTGTT
ATAGTTTGTTTTGTCGCAAAACTATAA
>AA QryID: NC_002952_(-)_2173430_2173618 HitID: NC_017342_(+)_1249818_1250006
rank=1 bitscore=103.0 evalue=3.0E-20 perc_ident=100.0 perc_conserv=100.0 perc_gaps=0.0
query_cvg=100.0 subj_cvg=100.0 alpha=68.0 beta=21 prob=0.9997918771936329
ATGCAAGTTGGCGGGGCCCCAACACAGAGAATTTCGAAAAGAAATTCTACAAACAATGTA
AGTTGGGGTGGGGCCCCAACAAAGAGAAATTGGATTCCCAATTTCAACAGACAATGCAAT
TTAAGTATGACAAATCAACGCTATAGTTTCAGTTTTACATCACCTATTCAATGGAAATAT
GCTTGTTGA
>AA QryID: NC_002952_(+)_1371966_1372094 HitID: NC_017341_(+)_1357160_1357282
rank=1 bitscore=57.4 evalue=1.0E-6 perc_ident=97.22 perc_conserv=97.22 perc_gaps=0.0
query_cvg=85.71 subj_cvg=90.0 alpha=85.42 beta=2 prob=0.9997916679520433
GTGAAGGTTATTTTTTATTGTCCAAAACGTGCTGATGACATTTTAAAAGCAAGTATGGAA
TATCAGTCGACAGACTATAAACGGAGGTATATCTCATGGAAAAAAATGAAAGTAATATTA
CTGATGTAA
>AA QryID: NC_002952_(-)_283057_283191 HitID: NC_017337_(-)_231665_231799
rank=1 bitscore=66.2 evalue=5.0E-9 perc_ident=97.73 perc_conserv=97.73 perc_gaps=0.0
query_cvg=100.0 subj_cvg=100.0 alpha=97.73 beta=5 prob=0.9997914006565752
TTGTCACGATACTTTCCGATTGTGATTACATTTGACGTTACAGTCATTTCAACGACAACC
CTTGCTACATTCGACTGCAATCCCTTTGAATTACAGTCACTGCGTTTCTATGTCATCTAC
AATCATTTGTCGTGA
>AA QryID: NC_002952_(-)_1110744_1110866 HitID: NC_018608_(-)_1119365_1119487
rank=1 bitscore=54.7 evalue=7.0E-6 perc_ident=100.0 perc_conserv=100.0 perc_gaps=0.0
query_cvg=100.0 subj_cvg=100.0 alpha=100.0 beta=5 prob=0.9997913892777199
ATGAACGACATGATGAACCTAGTTATTAACCAAATCGTTATTAAGTTACATTATAGAGAT
GATTGGAATGAATTTATCGATATATACTCCAATACGATTTTACTAGGGTTAACAATAAAT
TAA
>AA QryID: NC_002952_(-)_1652225_1652359 HitID: NC_017351_(-)_1619192_1619326
rank=1 bitscore=56.6 evalue=3.0E-6 perc_ident=97.73 perc_conserv=100.0 perc_gaps=0.0
query_cvg=100.0 subj_cvg=100.0 alpha=100.0 beta=5 prob=0.9997911902739828
ATGTTTAATGTGATACTTATTAATGATAAGTATAACGTAATTATCAAAATTTATATAGTT
ATGTCTACGTTAAAGTTAGAAAAATTAACTAGCAAAGACGAATTTTTAACAGATTTTGAT
TCAAGTATAAATTAA
>AA QryID: NC_002952_(-)_2070307_2070417 HitID: NC_010079_(-)_2032466_2032576
rank=1 bitscore=56.6 evalue=2.0E-6 perc_ident=100.0 perc_conserv=100.0 perc_gaps=0.0
query_cvg=100.0 subj_cvg=100.0 alpha=100.0 beta=4 prob=0.9997909565204375
TTGGATGGAAACAACCAAATTGATGTGTGTTTTTTTGTTCTAGTGAATAATTATTATACA
ATGAGTATCTATCCTAGAATTATCAATAGTAATGGTGATTATGCAACATGA
>AA QryID: NC_002952_(-)_2792112_2792225 HitID: NC_018608_(-)_2674160_2674273
rank=1 bitscore=60.8 evalue=1.0E-7 perc_ident=100.0 perc_conserv=100.0 perc_gaps=0.0
query_cvg=100.0 subj_cvg=100.0 alpha=100.0 beta=3 prob=0.9997909037534987
ATGAATGGCATACTTATAAAGTGTCTAAATATTGGTGGTCAATATTACTTGCAAATACGA
ATGTTAAGTAATGAAGACAATGCTGAAAAAACAATAAGTGGAAACTCTATATAG
>AA QryID: NC_002952_(+)_626241_626393 HitID: NC_017342_(-)_2732989_2733141
rank=1 bitscore=63.5 evalue=3.0E-8 perc_ident=100.0 perc_conserv=100.0 perc_gaps=0.0
query_cvg=100.0 subj_cvg=100.0 alpha=100.0 beta=2 prob=0.9997905731660783
GTGTTTATTTATAAAACAACAAATTTTTATATTTATAATGGTAGTAGTTATTCAGTCAAA
TACTACCAATATATCATTGTAGAGCTTAGGATATTGATTTATGACTCAGGCACATCAAAT
GAGAAGATTTATAAAAGAGATATACAACTCTAG
>AA QryID: NC_002952_(-)_2412135_2412230 HitID: NC_017673_(-)_2226865_2226960
rank=1 bitscore=50.4 evalue=1.0E-4 perc_ident=100.0 perc_conserv=100.0 perc_gaps=0.0
query_cvg=100.0 subj_cvg=100.0 alpha=100.0 beta=4 prob=0.9997897258389978
GTGATATTAACATGTTACAATACATTTAACACATCATTTGCAATCATTGAATTTAAATCA
```

AAGATTAGTGGAATAGATGAAGCACGTTCGAAATAA
>AA QryID: NC_002952_(+)_2691200_2691361 HitID: NC_017342_(-)_732465_732626
rank=1 bitscore=63.5 evalue=3.0E-8 perc_ident=100.0 perc_conserv=100.0 perc_gaps=0.0
query_cvg=100.0 subj_cvg=100.0 alpha=100.0 beta=1 prob=0.9997896359836693
ATGCCAATAGGTATACATTTGCGTGGAAGAAAAGTATTCAAAATCATGAATTGTTTTATT
TTGTATATCTGTATCAAAATCGATTTGATTAAAATCCGCTTTATTTTATCATCTATTCAA
ATGATTTTAGTGCGCTTATTTTACTATGGCATTATTGCCTAA
>AA QryID: NC_002952_(-)_1894365_1894478 HitID: NC_018608_(-)_1829593_1829706
rank=1 bitscore=45.4 evalue=0.004 perc_ident=100.0 perc_conserv=100.0 perc_gaps=0.0
query_cvg=100.0 subj_cvg=100.0 alpha=100.0 beta=5 prob=0.9997896126722201
ATGAAAAAAGTTTATATAAAATTAAAATATATCGTTCGTTATCATTTAGCGTTTGTTTTT
ATTTCAGGCTTTTCGCTAAATTTTTCCAAACAAAAATATGTTACTGTAAATTAA
>AA QryID: NC_002952_(-)_1422290_1422388 HitID: NC_017673_(-)_1294921_1295019
rank=1 bitscore=49.3 evalue=3.0E-4 perc_ident=100.0 perc_conserv=100.0 perc_gaps=0.0
query_cvg=100.0 subj_cvg=100.0 alpha=100.0 beta=4 prob=0.9997894742277276
ATGAATCATGCACCTTCTATAAAATTAGACCGAATTGAAACTTCAGTAAATATAGAGATA
CATCATCATTTCTTATACAATACAAGAGATTTATATTAG
>AA QryID: NC_002952_(-)_1458671_1458778 HitID: NC_010079_(-)_1425155_1425262
rank=1 bitscore=57.4 evalue=1.0E-6 perc_ident=100.0 perc_conserv=100.0 perc_gaps=0.0
query_cvg=100.0 subj_cvg=100.0 alpha=100.0 beta=2 prob=0.9997892603525294
TTGAAACATGATTTTGATGGTGAATCATTATTTAGAGTGCAAAGCTTGATTTATCGAGAC
GCTGATTTTGACATTCAGTTAGTCTACAAGCTTATCAACTTAAAATAG
>AA QryID: NC_002952_(-)_819031_819279 HitID: NC_017342_(+)_2568934_2569182
rank=1 bitscore=108.0 evalue=1.0E-21 perc_ident=100.0 perc_conserv=100.0 perc_gaps=0.0
query_cvg=100.0 subj_cvg=100.0 alpha=83.49 beta=4 prob=0.9997890719431659
TTGATTTTCCTAACTTATTGGTGTGTTTTTCATTTAGCATACATAATAGGTTACATTAAA
ATAACATTTTATACCAAAGTACACCAAAAGAATATTAGTACACGAATTAAACAACATTTT
TATAGAAACCTATTGCACTTTAACGTCAATAAGTATATTTTTATATTATCTCTAATTAAT
TGTGCGCGCTTAATAACAGAATATTCTCAATATTTTTATTTTTTTGTGATTTGTGAAATA
TTTAGTTGA
>AA QryID: NC_002952_(+)_2350488_2350613 HitID: NC_017342_(-)_1074496_1074621
rank=1 bitscore=47.0 evalue=0.002 perc_ident=100.0 perc_conserv=100.0 perc_gaps=0.0
query_cvg=100.0 subj_cvg=100.0 alpha=100.0 beta=4 prob=0.9997889114242122
ATGTATCATGGCTATATCATCTTTGAATTAATACAATTGACACCATTTAGCATGACCGTT
ATCTCTTTAATTCAGCTGATATTACCTATTGCAGTTTTATGTGACGAACTGTTGCACTTA
ATTTGA
>AA QryID: NC_002952_(-)_919123_919329 HitID: NC_017342_(+)_2452731_2452937
rank=1 bitscore=112.0 evalue=9.0E-23 perc_ident=100.0 perc_conserv=100.0 perc_gaps=0.0
query_cvg=100.0 subj_cvg=100.0 alpha=80.96 beta=5 prob=0.9997888816837908
ATGCCACAATCATGTTGTATTTATGTTGTCGCTTTATCTAGCATCAATGATTATTTTTTA
CACCAATCAAAAAATCGGACTGATATAAATAAGTACAAAGCTTATCTATCAATCCGATTT
AGTTATAAAACAAAAAAAGCCACAGTAATGTGGCTTTTTGTTATATTCAGTATCAAAATG
GTATCAATAACCGTTTTCGACAGTTAA
>AA QryID: NC_002952_(+)_1972908_1973009 HitID: NC_017337_(+)_1884513_1884614
rank=1 bitscore=50.1 evalue=2.0E-4 perc_ident=100.0 perc_conserv=100.0 perc_gaps=0.0
query_cvg=100.0 subj_cvg=100.0 alpha=100.0 beta=3 prob=0.9997885431126907
ATGCAAGGTTTTAAAGAGAAACATCAAGAACTAAAGAGTGCTCTATGCCAAATTGGACTG
ATGCGTTCAATATCGGAAGTTAGGCAACTAAACATTGCTTAA
>AA QryID: NC_002952_(+)_1366178_1366312 HitID: NC_017342_(-)_2017591_2017725
rank=1 bitscore=56.6 evalue=3.0E-6 perc_ident=100.0 perc_conserv=100.0 perc_gaps=0.0
query_cvg=100.0 subj_cvg=100.0 alpha=100.0 beta=1 prob=0.9997878325513244
ATGATTTTTAAAATTAATAGATTTGAAAAGTATACTTTATTTCATGAAAGTTTTTGGTAC
ATTTATTTGAAAATGTTTTTATTACAAAGTTACATGCCTAAATGTTTCTTAACAAAATTA
ATTCATCAAGTGTAA
>AA QryID: NC_002952_(+)_1022057_1022173 HitID: NC_018608_(+)_1034982_1035098
rank=1 bitscore=52.0 evalue=5.0E-5 perc_ident=100.0 perc_conserv=100.0 perc_gaps=0.0
query_cvg=100.0 subj_cvg=100.0 alpha=100.0 beta=2 prob=0.9997878024679929
ATGTATATGATTTTGATAAATACATGCTTTTTACGTAAATCAAGTTTGATACAGAAAGGA
CTAAATCAAACATTTATCGTTGTAATACGTTTAAATAACTTTATTAAAAAGTCATAA
>AA QryID: NC_002952_(+)_2780472_2780567 HitID: NC_007795_(+)_2701454_2701549
rank=1 bitscore=36.6 evalue=2.2 perc_ident=100.0 perc_conserv=100.0 perc_gaps=0.0
query_cvg=100.0 subj_cvg=100.0 alpha=100.0 beta=5 prob=0.9997872102197229

ATGAAAAATGTGTCAATTTACAAAACAACTCAAGAATTAAGTTTTGTAAGTTCGTTAGAT
TTTTATTTAACGAAACATTTAACTTTAATGCTTTAA
>AA QryID: NC_002952_(-)_2173344_2173448 HitID: NC_010079_(-)_2133177_2133275
rank=1 bitscore=50.8 evalue=1.0E-4 perc_ident=96.88 perc_conserv=100.0 perc_gaps=0.0
query_cvg=94.12 subj_cvg=100.0 alpha=94.03 beta=1 prob=0.9997870476584082
ATGGAAATATGCTTGTTGATATTTGAATATTTACATTCAAACAATATTCATCGCTATTTC
TATCAACTTAAAATTCGCAATAAAAAACTGCCAGCACGCGAATAG
>AA QryID: NC_002952_(+)_2201756_2201911 HitID: NC_018608_(+)_2075566_2075721
rank=1 bitscore=56.6 evalue=4.0E-6 perc_ident=96.08 perc_conserv=98.04 perc_gaps=0.0
query_cvg=100.0 subj_cvg=100.0 alpha=98.04 beta=3 prob=0.9997866876719725
ATGATTATCCATTGTTCAATCGTATCTAACTTTATATTTAACCTTTATATTGTAACAAAT
TTCAACTTAAATTTCTTATCTTTGAAACAGATTATCTCTTCAAAGTTAATTGTAAGAAAA
TTTAAAATATTTGTTGACATGCTAAAGCAGATATAG
>AA QryID: NC_002952_(-)_2073423_2073515 HitID: NC_018608_(-)_1984300_1984392
rank=1 bitscore=34.3 evalue=9.9 perc_ident=100.0 perc_conserv=100.0 perc_gaps=0.0
query_cvg=100.0 subj_cvg=100.0 alpha=100.0 beta=5 prob=0.9997864319274574
ATGACTAAAAATACAATAATTAGCCTTGAAAATGAAAAAACGCAGATCAATGATTCAGAA
AATGAATCTAGTGATCTCCGTAAAGCAAAATAA
>AA QryID: NC_002952_(+)_1366514_1366624 HitID: NC_002951_(+)_1350726_1350836
rank=1 bitscore=38.9 evalue=0.42 perc_ident=97.22 perc_conserv=100.0 perc_gaps=0.0
query_cvg=100.0 subj_cvg=100.0 alpha=97.22 beta=7 prob=0.9997862900651331
ATGAAGATAACTATTAAAGTGCGAAAAACAAATAAAAAAGAGAAAATAGAATTTTTAATA
GGGACGTTTATAATTATTTTAGTAATATTAGGGTTCAAAATTATGAAATAA
>AA QryID: NC_002952_(+)_1654007_1654138 HitID: NC_016928_(+)_1592197_1592328
rank=1 bitscore=54.3 evalue=9.0E-6 perc_ident=97.67 perc_conserv=97.67 perc_gaps=0.0
query_cvg=100.0 subj_cvg=100.0 alpha=97.67 beta=3 prob=0.999786170313621
ATGACAAGGATTGATTACGAATCAAGTATATCAATTGTTCTATCATTCGCTTTTTTTCTA
GACTATATTATGAAATTATATTTTACAATGCCCAAAACTATTTTAATAATCATTGAACAA
ATGGGTGTATAA
>AA QryID: NC_002952_(+)_2451108_2451224 HitID: NC_018608_(+)_2333472_2333588
rank=1 bitscore=42.0 evalue=0.045 perc_ident=100.0 perc_conserv=100.0 perc_gaps=0.0
query_cvg=100.0 subj_cvg=100.0 alpha=100.0 beta=3 prob=0.9997859602709354
ATGCAAATTTCTATACTTTTACAATTCTATTGTGTCAAGATATTAGCAAAAAATTACAAA
ACGTTAACAAAGCACACAAGGACGCTAATTTCTGTTATAGTCTTTCTTGTCGTTTAA
>AA QryID: NC_002952_(+)_1047434_1047535 HitID: NC_007795_(+)_942903_943004
rank=1 bitscore=50.8 evalue=1.0E-4 perc_ident=100.0 perc_conserv=100.0 perc_gaps=0.0
query_cvg=100.0 subj_cvg=100.0 alpha=100.0 beta=1 prob=0.9997858858952912
TTGAAATTTGCAGCAAATAAATTAACCTTGACTATTTTCAGAAAATTCTGTATAAATAAC
GAAAAAGAGATATGGATTAAATATGAAGAGGTGTTTTTATAG
>AA QryID: NC_002952_(-)_2581109_2581213 HitID: NC_007795_(-)_2487184_2487288
rank=1 bitscore=43.1 evalue=0.022 perc_ident=97.06 perc_conserv=100.0 perc_gaps=0.0
query_cvg=100.0 subj_cvg=100.0 alpha=97.06 beta=6 prob=0.9997858841647156
ATGTTAACTAATAGATTAACTAGTGTTAACTATGTAAAAAAGCGTTTAATTCAGAACATT
CAGCGTATTTTAAGTAAGAAAGTACTATATGGAGAAATGCAATAG
>AA QryID: NC_002952_(-)_477335_477478 HitID: NC_010079_(-)_475992_476135
rank=1 bitscore=78.2 evalue=1.0E-12 perc_ident=93.62 perc_conserv=93.62 perc_gaps=0.0
query_cvg=100.0 subj_cvg=100.0 alpha=93.62 beta=2 prob=0.9997858030659954
TTGTACTTTCAATTAACAGTTGGTACGATGGTTTTGTCATTTTTCATCAACGCAAATATA
AAAAGGACTAAGACACATACATGTCCTAGCCCTATGGATAAAATGCAAATTTCTGCTTTA
TCAAAACTATCACACTTTAGATAG
>AA QryID: NC_002952_(-)_2337472_2337639 HitID: NC_017342_(+)_1087469_1087636
rank=1 bitscore=58.5 evalue=1.0E-6 perc_ident=96.36 perc_conserv=98.18 perc_gaps=0.0
query_cvg=100.0 subj_cvg=100.0 alpha=96.36 beta=3 prob=0.9997852550890461
TTGAAAAAAGAAGCGACTTGCAAACACGAATGTTTTTCAAAAAAGTTATTGACTTTAAAT
TATAAAGTTGATAAGATAATAAAGTCGTCAAAAACGGTGATGATGTTTTTAAAGAAGAAT
TTCAAAAGTGTAAAACTTACCATTGAAATAACTATAAAAACAATATAA
>AA QryID: NC_002952_(-)_1195890_1195994 HitID: NC_010079_(-)_1184072_1184176
rank=1 bitscore=48.5 evalue=5.0E-4 perc_ident=100.0 perc_conserv=100.0 perc_gaps=0.0
query_cvg=100.0 subj_cvg=100.0 alpha=100.0 beta=1 prob=0.9997849815672124
TTGACAATTTTACGGCTTGAAAATCAATTTAATCATGGAAAATTTATAATATTCATTGTT
TTACATTTTCAAATCAATGAAAAACACAAGTGGTTTAATGTATAA
>AA QryID: NC_002952_(-)_673600_673719 HitID: NC_002951_(-)_700498_700617

```
rank=1 bitscore=49.3 evalue=3.0E-4 perc_ident=94.87 perc_conserv=97.44 perc_gaps=0.0
query_cvg=100.0 subj_cvg=100.0 alpha=97.44 beta=4 prob=0.9997840663762636
ATGCTGATCTTTTTGACTAATCCAAATGCTAACCCAGAAATACAATCACTGTGTCTAATA
AATAATTTGTTTTATAAACACTTTTTTGTTTACTTCTCATTTTTAATTAGTTATAATTAA
>AA QryID: NC_002952_(-)_2776211_2776378 HitID: NC_017342_(+)_647728_647895
rank=1 bitscore=103.0 evalue=2.0E-20 perc_ident=100.0 perc_conserv=100.0 perc_gaps=0.0
query_cvg=100.0 subj_cvg=100.0 alpha=79.55 beta=5 prob=0.9997840033552671
ATGTTAAATAATGATCATGGAATTGAAGACACAAAAATAATGGGCGACGCCTGTCCCCAT
TTGTATGGAATATTAATACTTTTTACAAAGCCTTATCTTTTAAAAAGAAGTCAAACTATG
ATTGTAATTAATAAATACGGGAATGAATTAGTAATAGATATAAAGTAA
>AA QryID: NC_002952_(-)_1945710_1945805 HitID: NC_017673_(-)_1757254_1757349
rank=1 bitscore=46.2 evalue=0.003 perc_ident=100.0 perc_conserv=100.0 perc_gaps=0.0
query_cvg=100.0 subj_cvg=100.0 alpha=100.0 beta=1 prob=0.9997839911390997
ATGAACATCTTTAAAACGAATCATAATTATGATGAAGTAATGAAGTATGACAATGGTGAA
TTCATAAAATATGGCAAATTGTTAAAAATATTTTAA
>AA QryID: NC_002952_(-)_886239_886334 HitID: NC_018608_(-)_889091_889186
rank=1 bitscore=41.2 evalue=0.089 perc_ident=100.0 perc_conserv=100.0 perc_gaps=0.0
query_cvg=100.0 subj_cvg=100.0 alpha=100.0 beta=2 prob=0.9997837633715105
TTGCAACAATACATAGAATCATTATTACAAGAATTATTTAATATCATCTTAGAATCTCAC
AAAATATCAACTTTGTTTAATTACTCAAAACGATAA
>AA QryID: NC_002952_(+)_1916941_1917036 HitID: NC_018608_(+)_1845736_1845831
rank=1 bitscore=40.0 evalue=0.16 perc_ident=100.0 perc_conserv=100.0 perc_gaps=0.0
query_cvg=100.0 subj_cvg=100.0 alpha=100.0 beta=2 prob=0.9997831986648495
ATGATGCACATCATATTTGTAACTCATAGAAAATTTTATAATTTTTATCATTATATTTCA
ACTGAAAATGAGAAACAAAATGGCACTTTTTACTAA
>AA QryID: NC_002952_(-)_2370417_2370533 HitID: NC_002951_(-)_2268888_2269004
rank=1 bitscore=53.1 evalue=2.0E-5 perc_ident=97.37 perc_conserv=100.0 perc_gaps=0.0
query_cvg=100.0 subj_cvg=100.0 alpha=97.37 beta=2 prob=0.9997831308895817
TTGTTTGTTTATGTTTATAATTTAAACGTAATCAAATTGAAAGCCTTTTCTCAAGATTTT
CATAACCTACAAATAAATATGTCATATTTTAAGAAAGGTATTTCAAAATTAAAGTAA
>AA QryID: NC_002952_(+)_275285_275392 HitID: NC_010079_(+)_282404_282511
rank=1 bitscore=52.0 evalue=5.0E-5 perc_ident=97.14 perc_conserv=97.14 perc_gaps=0.0
query_cvg=100.0 subj_cvg=100.0 alpha=97.14 beta=2 prob=0.9997819865548172
TTGTCTGTTTGTACACCGACGATTGCGCGCATTTATTTGACTAATTCAAAAAACATTGTT
GTTTTCTTAGAAAAAAGTAAACATGATAATAAAAATGTGAAAGTGTAA
>AA QryID: NC_002952_(-)_2810814_2810981 HitID: NC_017342_(+)_614031_614189
rank=1 bitscore=89.4 evalue=5.0E-16 perc_ident=100.0 perc_conserv=100.0 perc_gaps=0.0
query_cvg=94.55 subj_cvg=100.0 alpha=77.08 beta=3 prob=0.9997818666425171
ATGTATCAAATGAATAATAAAAATTTGTTTTGTATCACGTGTACACAAGTTAGTTACTAC
ATATTAACTTGTGTACGCGTTTTTTTATGGAAAGAAAGAATTCATAGTCATTCATTTGAC
TGTATAAAAAACTTTATACAACATGTTTTTGTGGGTGTTTTTGAATAA
>AA QryID: NC_002952_(-)_2628166_2628264 HitID: NC_018608_(-)_2505714_2505812
rank=1 bitscore=48.5 evalue=5.0E-4 perc_ident=96.88 perc_conserv=96.88 perc_gaps=0.0
query_cvg=100.0 subj_cvg=100.0 alpha=96.88 beta=3 prob=0.9997818215169189
ATGAAAGCAGCGGATTTTAGCGTTACTGCAAATAATTTTATATTAGTAGTGGATGTTGGT
CACACAAGAATTTCAAATATTAAAGCCCTCAGAATATGA
>AA QryID: NC_002952_(-)_1716696_1716815 HitID: NC_017333_(-)_1719732_1719851
rank=1 bitscore=53.5 evalue=2.0E-5 perc_ident=97.44 perc_conserv=97.44 perc_gaps=0.0
query_cvg=100.0 subj_cvg=100.0 alpha=97.44 beta=1 prob=0.9997811739744018
TTGAAAACAAGCACTATCGTAATGGTAGTTGCTTGTTTTTTTACGTTAAGAAAAATTGAA
AATCAAAGAGAATCTTTCGAGAAATATGAGTTGTTTAAATTACAACAAAAAATTGATTAG
>AA QryID: NC_002952_(-)_1580225_1580416 HitID: NC_017342_(+)_1806014_1806205
rank=1 bitscore=99.4 evalue=5.0E-19 perc_ident=100.0 perc_conserv=100.0 perc_gaps=0.0
query_cvg=100.0 subj_cvg=100.0 alpha=75.85 beta=7 prob=0.9997796886441153
ATGAGTGTTACAAATCTAAGAATTGCAAAAGAAGCTGATTTAGTCACAAAAAATGTCCTA
TGTAATAATTCGAGAAAGATGCACTATATACGGTCTTCTTACTATTCAAATGTAAAAGTT
GCTTATTTGCGTGGCTTTTTGTTTTATAAAAGTATAAAATTTTACTATAATATATCTTGT
AGAGAACAATGA
>AA QryID: NC_002952_(-)_1230842_1230973 HitID: NC_017673_(-)_1124771_1124902
rank=1 bitscore=44.7 evalue=0.008 perc_ident=90.7 perc_conserv=95.35 perc_gaps=0.0
query_cvg=100.0 subj_cvg=100.0 alpha=90.7 beta=9 prob=0.9997768299423784
GTGTTTATTTTTAATTTGATGCGTTCATTGAAACGAAGTATCGTGTTTTTTAATAACGTA
```

TCATCATTTAAGTTAAAAATTATCTTTGTGATTTTAAAAATGAGTGAAACTAAAATGATA
ATGTGTACTTAA
>AA QryID: NC_002952_(+)_215875_216015 HitID: NC_017342_(-)_335984_336124
rank=1 bitscore=86.7 evalue=3.0E-15 perc_ident=100.0 perc_conserv=100.0 perc_gaps=0.0
query_cvg=100.0 subj_cvg=100.0 alpha=78.02 beta=7 prob=0.9997764335464547
ATGAGTATGAAGGGGGATTTAATTGATGATTTTAAAATTCAAAATTTAAGGGGAGAGCGA
ACTATCAATGATGCTGCAAAACACAATCGCAATGAAAAAACAGGCGCGTCACCTTATGAA
GGTATACGCACCTGTTTATAG
>AA QryID: NC_002952_(+)_2424930_2425100 HitID: NC_018608_(+)_2307282_2307452
rank=1 bitscore=99.0 evalue=7.0E-19 perc_ident=100.0 perc_conserv=100.0 perc_gaps=0.0
query_cvg=100.0 subj_cvg=100.0 alpha=77.11 beta=5 prob=0.9997764138182417
TTGTTTAGATTTATATCTTTTGGACAACTTGTTGTTTATTCGACAACATTTATACCTAAA
AAAGATATATTACAACTCAATGACTTGGAGTGTGACAACAATGTTAAAGAGCCACTAAAG
ATTTATTATGTAGTGATTCTTAAGAGTGGGATAGAAATGATATTTTCATAA
>AA QryID: NC_002952_(-)_2384905_2385084 HitID: NC_017337_(-)_2317222_2317401
rank=1 bitscore=97.4 evalue=2.0E-18 perc_ident=100.0 perc_conserv=100.0 perc_gaps=0.0
query_cvg=100.0 subj_cvg=100.0 alpha=77.01 beta=5 prob=0.9997748804077384
TTGACAGAGATTGTAGAATTTCTGTCAGATGGTAAGTTAGCAAGTAGGCTAGAAATTCAA
GAAACAATGGGACTTCAAAAAAGTTATACATCTGAGTTAATTGCTGAACTAAAGAAATTA
GAGATTATCGGATCTGAAGGTAGAGGTCCGGGAACTAGATATTATTTAATTAGAGACTAA
>AA QryID: NC_002952_(+)_2701148_2701249 HitID: NC_010079_(+)_2672107_2672208
rank=1 bitscore=45.1 evalue=0.006 perc_ident=96.97 perc_conserv=96.97 perc_gaps=0.0
query_cvg=100.0 subj_cvg=100.0 alpha=96.97 beta=1 prob=0.9997736321384169
ATGATAAAAATACAAGCAATTGAACTTTATTACGGAAAATCAAACTTTTCAATGACGCTA
TTAATTTGGCTAGTCGCGAAGATGCATTTTTCTAAAATGTGA
>AA QryID: NC_002952_(+)_2302458_2302556 HitID: NC_010079_(+)_2263846_2263944
rank=1 bitscore=39.3 evalue=0.35 perc_ident=96.88 perc_conserv=96.88 perc_gaps=0.0
query_cvg=100.0 subj_cvg=100.0 alpha=96.88 beta=2 prob=0.999772069889407
ATGGCGACTTATTTGGTCTTTGATGATGCAATGTTTATTAACAAATTTTCAACTATTATT
TCTTACATTAGTCATATTTTTGACAATTTACTATTATAA
>AA QryID: NC_002952_(+)_847372_847470 HitID: NC_002951_(+)_841589_841693
rank=1 bitscore=41.6 evalue=0.069 perc_ident=96.88 perc_conserv=96.88 perc_gaps=0.0
query_cvg=100.0 subj_cvg=94.12 alpha=94.03 beta=4 prob=0.9997719339876521
GTGTTATGGGAGGAGTCTTTTTATTTTACAAGCGTTAAATACTTTAAAAAAATGTGAAGAA
GTTGTTAAACGTTGTTATGTACTTAGTTTAAAAAAATAG
>AA QryID: NC_002952_(-)_2627443_2627601 HitID: NC_007622_(-)_2475807_2475983
rank=1 bitscore=63.5 evalue=3.0E-8 perc_ident=80.85 perc_conserv=85.11 perc_gaps=0.0
query_cvg=90.38 subj_cvg=81.03 alpha=80.26 beta=5 prob=0.9997716258782687
ATGATGTTATTCAAAAATTTAAATTTTGATAATTTTTTTGTAAATATGAGGTTTTTGAAG
CGGATTTCTCACAAAGTTTTTAAAATTATTAAGCCTGAAAACGATAAAGCGGTAGGGAAC
GTTTTTCTGAAAGTTAGTGATACAATAGTTTTAAGTTGA
>AA QryID: NC_002952_(+)_1364687_1364839 HitID: NC_017349_(+)_1337921_1338091
rank=1 bitscore=72.8 evalue=5.0E-11 perc_ident=93.75 perc_conserv=95.83 perc_gaps=0.0
query_cvg=96.0 subj_cvg=85.71 alpha=85.18 beta=1 prob=0.9997715447320077
GTGATTTTGAAAAATATAGGTTATTATAATTTAGAATTGCAAATTATAAATCTTGTCATT
GGATATGGGAATTCAAACATATCACTTACAACAATTTGTGGAATGTTAAATAACTATAAA
GAGGAAAGGAGAAAAAATGATAAGAATGGTTAA
>AA QryID: NC_002952_(-)_1257631_1257735 HitID: NC_013450_(-)_1228556_1228660
rank=1 bitscore=47.4 evalue=0.001 perc_ident=94.12 perc_conserv=100.0 perc_gaps=0.0
query_cvg=100.0 subj_cvg=100.0 alpha=94.12 beta=3 prob=0.999770844208659
ATGCCTATACATAGACTATTATATTTTCCCATTTTAAATTCTAAAAAACAAGTAGAAATT
TTAGAAACAGTTATCAATTTATTGATTACTGTTTCTTATGCATGA
>AA QryID: NC_002952_(-)_2592141_2592284 HitID: NC_017673_(-)_2402233_2402376
rank=1 bitscore=89.4 evalue=4.0E-16 perc_ident=100.0 perc_conserv=100.0 perc_gaps=0.0
query_cvg=100.0 subj_cvg=100.0 alpha=66.33 beta=14 prob=0.9997688147337578
GTGAGGGGATTTGGTGTATTTAGATGTTCAATTTATTATGTAACTAATTATAGCATTGAT
GAACAAGGCGCTCAACGTAATATTACAATTAATGGATTGTTTCACAACATGGTAAAGCTA
GGATGTCTTTTTATTAAGAAATAA
>AA QryID: NC_002952_(+)_2533502_2533594 HitID: NC_010079_(+)_2494689_2494781
rank=1 bitscore=38.9 evalue=0.44 perc_ident=93.33 perc_conserv=93.33 perc_gaps=0.0
query_cvg=100.0 subj_cvg=100.0 alpha=93.33 beta=5 prob=0.9997672234012195
GTGACAAAAAAGCGAAGATATGACACAACTGAATTTGATTTAGCACATAGTATGACAGCT

AAAATAACTTTACATCAAGTGCTATACAAATAA
>AA QryID: NC_002952_(+)_1286741_1286833 HitID: NC_007795_(+)_1180529_1180621
rank=1 bitscore=35.4 evalue=4.4 perc_ident=96.67 perc_conserv=96.67 perc_gaps=0.0
query_cvg=100.0 subj_cvg=100.0 alpha=96.67 beta=2 prob=0.9997670206392983
TTGGCAGTAGTCAACGAAATGACTTTGATGATTATGTTGAAGTCATTTCTTTATTTTTGT
CTAATTTTCAATCAAATTGATATTCCGCATTAA
>AA QryID: NC_002952_(+)_886622_886777 HitID: NC_017342_(-)_2501433_2501588
rank=1 bitscore=70.9 evalue=2.0E-10 perc_ident=100.0 perc_conserv=100.0 perc_gaps=0.0
query_cvg=100.0 subj_cvg=100.0 alpha=76.69 beta=9 prob=0.9997653278727765
TTGATAGTTAAAAAATTACTTAAGGCTATAAAGCAAAATTTTTTATATGAGCAGTCGAAT
ATAACGTTTAAAATGATTCTTTTTGGATATAAACGATTAAGTAAAATGATTTTTCAGTTT
GAAATTAATCATATGAATTTCTTAAGAGAAGGGTGA
>AA QryID: NC_002952_(-)_1860275_1860379 HitID: NC_016928_(-)_1790543_1790656
rank=1 bitscore=36.6 evalue=2.2 perc_ident=90.32 perc_conserv=96.77 perc_gaps=0.0
query_cvg=91.18 subj_cvg=83.78 alpha=81.89 beta=6 prob=0.99976431784585
ATGATATTGATTCATTACATGTTACTGATGAACATACCTGCATATGTGTTTCTAGCTTGT
TTGTTACATATTGCATTTAGACTTGGACAAAAATTTATGTCCTAG
>AA QryID: NC_002952_(+)_513469_513618 HitID: NC_017342_(-)_49828_49956
rank=1 bitscore=72.8 evalue=5.0E-11 perc_ident=100.0 perc_conserv=100.0 perc_gaps=0.0
query_cvg=85.71 subj_cvg=100.0 alpha=65.76 beta=1 prob=0.9997621601251525
TTGGATTATCGAAGTAGATCTATGGTTATGGTGTGTTGGTGGCATGGTTTACCTGGTTTT
ACTGAGGGATGGGTAATCTTAAAGAAGCAAATCGTTGGTTGTGATTTGTTACTTCTAATA
GTAATGATTCGAATTGAATCATCGAAGTAG
>AA QryID: NC_002952_(-)_528150_528305 HitID: NC_007795_(-)_461457_461612
rank=1 bitscore=73.2 evalue=4.0E-11 perc_ident=100.0 perc_conserv=100.0 perc_gaps=0.0
query_cvg=100.0 subj_cvg=100.0 alpha=75.22 beta=7 prob=0.9997469482973149
ATGTTAAGGTATTTATTACTTTCAATGTATTTATTTTACATTAAAAAACGTCGATGTCAT
CATTTTTTTATCATAGTCAACATCATTTTATCAGTATCAATGCAAAATGATAACTTACCA
TTTAAAATTACAGCTTGCGTTTATAAAATCGTTTAA
>AA QryID: NC_002952_(+)_817285_817386 HitID: NC_018608_(+)_820083_820190
rank=1 bitscore=38.9 evalue=0.45 perc_ident=93.94 perc_conserv=93.94 perc_gaps=0.0
query_cvg=100.0 subj_cvg=94.29 alpha=91.26 beta=3 prob=0.9997408603743078
ATGGTAATAACTGAAATTGATGTCTATAGAGTGGGACGAGAAAATATAGTTATAGTTGTC
TATAACGAGCATATTAAGTTTTTATTTATACTGATATCTTGA
>AA QryID: NC_002952_(-)_276825_276950 HitID: NC_017341_(-)_254676_254801
rank=1 bitscore=50.8 evalue=9.0E-5 perc_ident=100.0 perc_conserv=100.0 perc_gaps=0.0
query_cvg=100.0 subj_cvg=100.0 alpha=71.64 beta=14 prob=0.9997386807703852
ATGGCAACGATGTACGGAGTAAGAAGTGACATCGCTGCTTTTATTTTTAATGTTGCATTT
GAAACATTAAGTTCATCATGCACTGTAGTGAGTCCGCAAATCGCCTCTGCTAGACAATCA
TCTTAA
>AA QryID: NC_002952_(+)_566927_567025 HitID: NC_013450_(+)_516763_516861
rank=1 bitscore=37.0 evalue=1.6 perc_ident=93.75 perc_conserv=100.0 perc_gaps=0.0
query_cvg=100.0 subj_cvg=100.0 alpha=93.75 beta=1 prob=0.9997385289509073
ATGAGTAAGCGAGAGCCGAAGGAGAGGAAAGAAGCAAGCGATTGTCACAAGTCAAGAAAG
GTTCTTAGCGACGATGGTAGCCAACTTACGTTCCGCTAG
>AA QryID: NC_002952_(-)_960608_960724 HitID: NC_002951_(-)_968265_968399
rank=1 bitscore=45.1 evalue=0.006 perc_ident=97.37 perc_conserv=97.37 perc_gaps=0.0
query_cvg=100.0 subj_cvg=86.36 alpha=90.73 beta=1 prob=0.9997377792368485
TTGTACTTAATCAATAAAATTGATATTTATGTGACGTTTCACACCTATTTTTCAACAAAC
ATTTCAAACCGGCGTCAAACACTCAAACATTTTTTATACAATTTGCATGTTTTTTAA
>AA QryID: NC_002952_(+)_1580370_1580507 HitID: NC_007795_(+)_1450171_1450308
rank=1 bitscore=49.7 evalue=4.0E-4 perc_ident=91.11 perc_conserv=93.33 perc_gaps=0.0
query_cvg=100.0 subj_cvg=100.0 alpha=91.11 beta=1 prob=0.999737189109615
GTGACTAAATCAGCTTCTTTTGCAATTCTTAGATTTGTAACACTCATTTTCTTACCATTT
GTAAAATGCTTTATGAATTTAATGAATTATTTTTGGAATTCTAATAATTTACCTTTATTA
TTTCTGTTAACTTTTTGA
>AA QryID: NC_002952_(+)_1037463_1037558 HitID: NC_002951_(+)_1036544_1036639
rank=1 bitscore=36.2 evalue=2.7 perc_ident=93.55 perc_conserv=96.77 perc_gaps=0.0
query_cvg=100.0 subj_cvg=100.0 alpha=93.55 beta=1 prob=0.9997343295425399
GTGATCTATGAAAAATGGATTTTGAAGAATATGTATCAAAAGATGCGATATAGTATTAAG
AAAATGTGCCTTTTATATTTAGCATTTTTTCAATAG
>AA QryID: NC_002952_(-)_2437537_2437725 HitID: NC_018608_(-)_2319899_2320087

rank=1 bitscore=95.1 evalue=1.0E-17 perc_ident=95.16 perc_conserv=96.77 perc_gaps=0.0
query_cvg=100.0 subj_cvg=100.0 alpha=71.85 beta=4 prob=0.9997218502395856
ATGTATCTCTGCATTAATAAGTTATATCATAATTATTTTAGTGCATACTTTATGGAAGGC
ATATCAGGGAATGCCTATCAATTAAAGAAGAGGTTTAAAAGGATTACAACAGAATGTTAT
GATTTTGTAGAAAGATATATTACAACGTTTTATAAAAACATAATATTGTTAATGGAAAAT
GAAATGTAA
>AA QryID: NC_002952_(-)_1819542_1819655 HitID: NC_018608_(-)_1754524_1754637
rank=1 bitscore=65.1 evalue=5.0E-9 perc_ident=100.0 perc_conserv=100.0 perc_gaps=0.0
query_cvg=100.0 subj_cvg=100.0 alpha=80.3 beta=2 prob=0.9997175862583094
TTGTTGCAAAATAATACTCAGCATTTTAATATAGTTTTGACGAAAAAAGTTGATTTACAA
ATATATAAACGTTGTGATTTCAATGTTTGTATAGGGAATAGACCTATGATATAA
>AA QryID: NC_002952_(+)_1065386_1065574 HitID: NC_018608_(+)_1074136_1074324
rank=1 bitscore=96.3 evalue=5.0E-18 perc_ident=100.0 perc_conserv=100.0 perc_gaps=0.0
query_cvg=100.0 subj_cvg=100.0 alpha=71.41 beta=2 prob=0.999713495732091
ATGAATGCTGATATATTATTTGAGTGCTTAAGTTTCGAAGAACAATTATCAGTTAGCATA
TTAAAAGCTTTTGATAGAATCATTTTGAGTGAGGTGTTTAAAGGAGAGGAATATAACTCG
GTAGTATTTACAGTCTCTTTAGAGTATTATTTAAAACATATAGAATATAAAAAACTGAAC
GTACTATGA
>AA QryID: NC_002952_(-)_2197012_2197140 HitID: NC_018608_(-)_2070825_2070953
rank=1 bitscore=70.5 evalue=1.0E-10 perc_ident=97.62 perc_conserv=100.0 perc_gaps=0.0
query_cvg=100.0 subj_cvg=100.0 alpha=77.47 beta=3 prob=0.999705330389274
TTGATAAAAACAATGTCAATAGAGAACATGCGTACTTATCAACAAAGAGTGTATAACTTG
TGGATAAAAGGTGTTGATAACGTCATTTATTGTGGATATCAGGTGGGAAAACAGAAAATA
AATGATTGA
>AA QryID: NC_002952_(-)_2213537_2213635 HitID: NC_013450_(+)_516763_516861
rank=1 bitscore=36.2 evalue=2.7 perc_ident=90.62 perc_conserv=96.88 perc_gaps=0.0
query_cvg=100.0 subj_cvg=100.0 alpha=90.62 beta=1 prob=0.9996864492927297
ATGAGTAAGCGAGAGCCGAAGGAGAGGAAAGAAGCAAGCGATTGTCACAAGTCAAGAAAG
GTTTTTAGCGACGATGGTAGCCAACTTACGTTCCGCTAG
>AA QryID: NC_002952_(+)_116736_116843 HitID: NC_002951_(+)_97875_97982
rank=1 bitscore=63.5 evalue=2.0E-8 perc_ident=100.0 perc_conserv=100.0 perc_gaps=0.0
query_cvg=100.0 subj_cvg=100.0 alpha=78.02 beta=2 prob=0.9996806151875118
GTGGATGGTTATTTATTGCGTTGTTCTAAAAATTTGGACATTTATATGGAAAGAACTATA
TATAATGTGAAGGTTATGGCAATTCGCTTCAATGAAGGCGACGTGTAA
>AA QryID: NC_002952_(-)_2694477_2694575 HitID: NC_002951_(-)_2601983_2602081
rank=1 bitscore=52.4 evalue=3.0E-5 perc_ident=83.87 perc_conserv=90.32 perc_gaps=0.0
query_cvg=96.88 subj_cvg=96.88 alpha=81.25 beta=3 prob=0.999671475783967 9
TTGATAAAAAAGAAGCATCTGTCTGGATCATTAGTACCAGATAGATGCTTCTTAATTATC
TTTCATAAAGTTGAAAAATTTATGGGAAATGCATGCTAA
>AA QryID: NC_002952_(+)_652695_652796 HitID: NC_017337_(+)_658409_658510
rank=1 bitscore=35.4 evalue=4.3 perc_ident=81.82 perc_conserv=84.85 perc_gaps=0.0
query_cvg=100.0 subj_cvg=100.0 alpha=84.85 beta=7 prob=0.9996513614446916
ATGTGTCAATACTTATGTTTGAAAAGTGTTGAATTAGGTGTGATTATTATGATGATTTAT
TATTTGAAAGTTGTTGCATTAACGGTGAGATTGCGTAATTGA
>AA QryID: NC_002952_(+)_869850_870008 HitID: NC_017342_(-)_2518207_2518365
rank=1 bitscore=92.4 evalue=5.0E-17 perc_ident=100.0 perc_conserv=100.0 perc_gaps=0.0
query_cvg=100.0 subj_cvg=100.0 alpha=63.94 beta=5 prob=0.9996324351081594
ATGTGTAAAACGCTGCATGATACAAACGAAGGTAAAAATTTGACTCCCTTTAGTAGTGGA
CCCGTACGTTTATCGTGCGGGTCGTTTTTATTTATATCTTATTCCCGTTATACATCAATT
CAAAGCATTAATTTTTTAAACAAATTTAAGAATAGATAG
>AA QryID: NC_002952_(+)_366034_366132 HitID: NC_017337_(+)_371314_371412
rank=1 bitscore=33.1 evalue=22.0 perc_ident=87.5 perc_conserv=93.75 perc_gaps=0.0
query_cvg=100.0 subj_cvg=100.0 alpha=87.5 beta=2 prob=0.9996064321780768
ATGGAAAGCATTTTTAAAATTAAAATTGAACTAATGAATGTCATTTGTAGGTCTGAAAAT
ATGAATATGAAAAAGAAAAATACAAGCGAAAAGATATAA
>AA QryID: NC_002952_(-)_2608897_2609001 HitID: NC_010079_(-)_2568014_2568118
rank=1 bitscore=40.4 evalue=0.14 perc_ident=100.0 perc_conserv=100.0 perc_gaps=0.0
query_cvg=100.0 subj_cvg=100.0 alpha=78.15 beta=4 prob=0.9996017661477623
GTGCCTAATGAGATTTATTTTATTATACCAGTTCTATTGAATAAATTAATTAAAGAGGTA
TATTTACCATTATTTTTGACACCTTATATGTTAATAGGTGTATAA
>AA QryID: NC_002952_(-)_2169367_2169468 HitID: NC_017673_(-)_1976314_1976415

rank=1 bitscore=47.8 evalue=0.001 perc_ident=84.85 perc_conserv=84.85 perc_gaps=0.0
query_cvg=100.0 subj_cvg=100.0 alpha=84.85 beta=1 prob=0.9995828167809223
TTGATATTGTTAGGTGCATATGAGGCATGTAGAGGTATAGTGGAAGTTAAAGAAATGAAA
ACGATGAGATATCAGTTTAAAGTTAGTCAATTCAAATTTTAA
>AA QryID: NC_002952_(-)_2079397_2079543 HitID: NC_017673_(-)_1878373_1878519
rank=1 bitscore=43.1 evalue=0.043 perc_ident=72.92 perc_conserv=72.92 perc_gaps=0.0
query_cvg=100.0 subj_cvg=100.0 alpha=72.92 beta=16 prob=0.999576110951218
TTGGTTGCTGACTTAGATGCAATCGAGGGTTTATTTATATCTATAGAAGTCATATTACTT
TTAACTTTATTCATTGTACATGTTAATGGTAAAAATATTAATTTTATTAATGCGTTAGCT
TTAATTATATTAAGGCAAACTGTATAA
>AA QryID: NC_002952_(+)_1287833_1287946 HitID: NC_002951_(+)_1285396_1285509
rank=1 bitscore=42.4 evalue=0.04 perc_ident=97.22 perc_conserv=100.0 perc_gaps=0.0
query_cvg=97.3 subj_cvg=97.3 alpha=71.46 beta=6 prob=0.9995684432086581
ATGGGTGATAAGATATTAATGCTTATCACCTTTTTTAAAAAGAAAATCAAGGCAAATTAC
AAATATTCAATTAGAGTATTGGCAATCTTGCCTATAATAATGCTAAAATTATAA
>AA QryID: NC_002952_(+)_1994869_1995060 HitID: NC_017763_(+)_1904239_1904424
rank=1 bitscore=85.1 evalue=9.0E-15 perc_ident=85.25 perc_conserv=88.52 perc_gaps=0.0
query_cvg=96.83 subj_cvg=100.0 alpha=68.28 beta=2 prob=0.9995633748853949
TTGACACTTGCTCTTTTAAGAATACTAACATTTCATTGTATTTCCGTTAAATTAACGGAG
AAGGCGGATTACCTCGATATATTTATCTCTAGAGTGTTAAAAAATTGTACATTCTTAAAC
AATCAAAAGTATACGTTATTAAATTATCATTTCCACTGGAGAATCATGGCGACTAAAAGA
TTATTAAAGTAA
>AA QryID: NC_002952_(-)_1908131_1908271 HitID: NC_016928_(-)_1840337_1840477
rank=1 bitscore=76.6 evalue=3.0E-12 perc_ident=97.83 perc_conserv=100.0 perc_gaps=0.0
query_cvg=100.0 subj_cvg=100.0 alpha=70.16 beta=2 prob=0.9995620295454619
TTGGAAATTTTAATTATTAATTTAAAATTACAGTCCGATGGTTATGATGCATTTACATAT
AATATGATTGCATTAATTTTTATAAGTAGTTGGAAGTGTATTAGTGTGGGGCGTTTCATT
TTTGTTCGCATTTATGAATAG
>AA QryID: NC_002952_(-)_1580533_1580649 HitID: NC_018608_(-)_1564044_1564160
rank=1 bitscore=62.4 evalue=4.0E-8 perc_ident=89.47 perc_conserv=89.47 perc_gaps=0.0
query_cvg=100.0 subj_cvg=100.0 alpha=65.89 beta=11 prob=0.9995547200592216
GTGCAACGCTTAATGCATATACAAATTGAATGTGCTGATAATGATTTACTCAACTTAAAA
GGTGATTTTTATTCAACGATGAATGAAAGTTGCCTTTTTACTTTTGGTAAAAGTTAA
>AA QryID: NC_002952_(+)_1068569_1068679 HitID: NC_009632_(+)_1150678_1150788
rank=1 bitscore=58.2 evalue=7.0E-7 perc_ident=100.0 perc_conserv=100.0 perc_gaps=0.0
query_cvg=100.0 subj_cvg=100.0 alpha=71.57 beta=4 prob=0.9995509982322296
GTGTCATTGTGCACACAACAAAAATTGAATCATGAATTACAAGCAAAAGTAGCGGTGATT
GTTAAAATTGATGGTAAACAATCACCGCTATTTTTGCTTGTGTATGTATAA
>AA QryID: NC_002952_(+)_703024_703116 HitID: NC_009487_(+)_736497_736589
rank=1 bitscore=48.5 evalue=5.0E-4 perc_ident=100.0 perc_conserv=100.0 perc_gaps=0.0
query_cvg=100.0 subj_cvg=100.0 alpha=70.11 beta=7 prob=0.9995368564782208
TTGTTTTGTTATTATAAACAACACAAAGGAGATAACTTCTCTATTGAAGAAGTTAAAAAC
ATTATAGCAGACAATGAAATGAAAGTAAATTAA
>AA QryID: NC_002952_(-)_2640574_2640705 HitID: NC_002951_(-)_2537025_2537156
rank=1 bitscore=77.0 evalue=1.0E-12 perc_ident=95.35 perc_conserv=100.0 perc_gaps=0.0
query_cvg=100.0 subj_cvg=100.0 alpha=70.1 beta=2 prob=0.9995326683784074
GTGAATGTAGTTAACGGACAACTGCATTCGCTTGTAGAGCCACAAGAAGCAACTTTAAAT
AAGGTTTACGGTTGCATGTTGATGCAACAACCGATTACTAAGTCATGCTTTCCACTTTGC
GGATTAGCATGA
>AA QryID: NC_002952_(+)_729232_729351 HitID: NC_010079_(+)_746982_747101
rank=1 bitscore=50.8 evalue=1.0E-4 perc_ident=92.31 perc_conserv=94.87 perc_gaps=0.0
query_cvg=100.0 subj_cvg=100.0 alpha=65.82 beta=11 prob=0.9994752097906959
TTGGTGCTTCAGCTGATTTTAATGAAAATAGCATTAAATGATTTTGAAAAAGATAAGAGT
GTGTTATTTATATTTTTGAAAAATCACTTTTATGAAGAAATGTGTGTACTATCTAATTAA
>AA QryID: NC_002952_(+)_123607_123729 HitID: NC_018608_(+)_103660_103782
rank=1 bitscore=54.7 evalue=8.0E-6 perc_ident=97.5 perc_conserv=100.0 perc_gaps=0.0
query_cvg=100.0 subj_cvg=100.0 alpha=74.5 beta=1 prob=0.9994497114974599
ATGTCTAAAAATGTCGTATATGTAATCAGAGTAGAAAGTGTTGAGGCGTTTCAGAAGTTG
TTTAGAAAAGTAAGTAAAATAAAAAATGCACTGAGCAACAAAAGATGTTGCTCGTGCATT
TAG
>AA QryID: NC_002952_(+)_1972681_1972785 HitID: NC_002976_(+)_2266163_2266258

rank=1 bitscore=45.4 evalue=0.005 perc_ident=80.65 perc_conserv=93.55 perc_gaps=0.0
query_cvg=91.18 subj_cvg=100.0 alpha=46.68 beta=21 prob=0.9994391367864808
GTGTATAAGGTGATGCTTATTTTCGTTCACATCATAGCACCAGTCATCAGTGGCTGTGCC
ATTGCGTTTTTTTCTTATTGGCTAAGTAGACGCAATACAAAATAG
>AA QryID: NC_002952_(+)_341759_341872 HitID: NC_010079_(+)_352588_352701
rank=1 bitscore=57.0 evalue=1.0E-6 perc_ident=94.59 perc_conserv=97.3 perc_gaps=0.0
query_cvg=100.0 subj_cvg=100.0 alpha=70.58 beta=2 prob=0.999291905129162
GTGCAAAACCAACCTCGTTTTACATTTGATTCAAAGAAGAAGGTAAAAAATAAGATTATT
TGCAACTTAAAAAGTCAATTAGCTTATCGGTATCCATACATCATGGATAAATAA
>AA QryID: NC_002952_(+)_556815_556907 HitID: NC_013450_(+)_508439_508531
rank=1 bitscore=41.2 evalue=0.087 perc_ident=96.67 perc_conserv=96.67 perc_gaps=0.0
query_cvg=100.0 subj_cvg=100.0 alpha=65.96 beta=7 prob=0.9991727215654009
ATGGTTAATGGTAAAGTATTAGAAACATCGAAAGAGATGTATGTTGAGCAAAAATGTCTG
ATATTTTATAAAACTTTAAAGGAAAATGTTTGA
>AA QryID: NC_002952_(-)_1166989_1167120 HitID: NC_018608_(-)_1175650_1175781
rank=1 bitscore=53.9 evalue=1.0E-5 perc_ident=90.7 perc_conserv=95.35 perc_gaps=0.0
query_cvg=100.0 subj_cvg=100.0 alpha=66.91 beta=5 prob=0.9991546500144801
TTGTTTTATCAAACCAACTATTACATATCTATCAGCATGTTTGATTATCATTTAACGTTC
ATAGATTTATTAATCCCACCATTCTCAAGTCGTAAACAATATGATGACTTATCATTCAAC
TTTATATATTAA
>AA QryID: NC_002952_(+)_22228_22320 HitID: NC_002951_(+)_22263_22355
rank=1 bitscore=34.3 evalue=9.3 perc_ident=80.0 perc_conserv=90.0 perc_gaps=0.0
query_cvg=100.0 subj_cvg=100.0 alpha=80.0 beta=1 prob=0.9990712436284594
ATGTTAAGTTTGTGTATTGGAAGTGATGAATTAGTGCTGTCAAAGACTCTGTTAAAGGGT
TTTGAGGACGTTGAAGACGATTTGTTAAAATGA
>AA QryID: NC_002952_(-)_2068115_2068303 HitID: NC_017673_(-)_815608_815802
rank=1 bitscore=67.4 evalue=2.0E-9 perc_ident=61.67 perc_conserv=68.33 perc_gaps=3.33
query_cvg=96.77 subj_cvg=90.62 alpha=58.74 beta=51 prob=0.9990254316516372
TTGGGGTGTGGGCCCCAACACAGAGAATTTCGAAAAAAGAAATTCTACAGGCAATGCAGG
TTGGGGTGTAGGCCCCAACACAGAGAAATTGGAACACCAATTTCTATAGGCAATGCAAGT
TGGCGGGGCCCCAACACAGAGAATTTCAAAAAAGAAATTCTACAGGCAATGCAAGTTGGG
GACGATTAA
>AA QryID: NC_002952_(-)_452157_452417 HitID: NC_017673_(-)_387416_387676
rank=1 bitscore=99.4 evalue=7.0E-19 perc_ident=76.74 perc_conserv=77.91 perc_gaps=0.0
query_cvg=100.0 subj_cvg=100.0 alpha=57.99 beta=3 prob=0.9988785116304371
ATGCTAACTGTTAATAATCAATTCTTATCCGTCAAGACTCTTTTTTATACAATTCACACT
TTAAACCATTTTAATTTTAAAAACTGTAAAACTTTCAGACACAAAAATGACATCATATCG
TCGCAGTACCATGTCAAAATTTTCAAAGATCATGGTGACACGCATCAACTAAAACGCGTC
ATTACGGTATTTTTAACATTAATAACATATGCGTATTTACTATACACGGCGACAATAAAT
ACAGATTGCAATTTTTTTATAA
>AA QryID: NC_002952_(+)_2540076_2540249 HitID: NC_017342_(-)_886351_886524
rank=1 bitscore=43.5 evalue=0.03 perc_ident=71.93 perc_conserv=71.93 perc_gaps=0.0
query_cvg=100.0 subj_cvg=100.0 alpha=71.93 beta=5 prob=0.9987545713554574
TTGCTTACTATCTTTCTAATTTTACTTTTATATTGTGCACGATGGATAAAAACAAGCGTT
TTCCTAGGAGTTTTCTTTAATTTCTTTTATTTTTCACTATTACCTTTTCAAAAATATTCA
ACTATAATAGGTATACTATCCATGATTTTAAAAATTTCCATGTTTTACATTTAA
>AA QryID: NC_002952_(-)_457960_458121 HitID: NC_018608_(-)_484315_484476
rank=1 bitscore=50.8 evalue=2.0E-4 perc_ident=71.7 perc_conserv=73.58 perc_gaps=0.0
query_cvg=100.0 subj_cvg=100.0 alpha=71.7 beta=1 prob=0.998587671213647
ATGCTATTTTTTTCACCATTCACACTTTCTTTCAATTTAATTATGTTTTCCACATTGTTT
CATGTCACTAGTGCGACACCGTGCGACTATAGGTATCAACTATTTTCACAGTTTTTATTG
TCGTTATTATCTTTCATCGACCCACTTCATTTCCGATCGTAA
>AA QryID: NC_002952_(-)_2626088_2626216 HitID: NC_007622_(-)_2473470_2473598
rank=1 bitscore=55.5 evalue=4.0E-6 perc_ident=88.1 perc_conserv=90.48 perc_gaps=0.0
query_cvg=100.0 subj_cvg=100.0 alpha=63.29 beta=1 prob=0.998580573299692
GTGTACTTCAATTTTCACATAAATATTAAAAAAATTTTCCAGAATGAAATGGCACTAAAT
TTTTCAAAAATGGGAATACTATATTTGATATGTTTTATTAGAGCGATTTTAATGGGTAAT
AAGCATTGA
>AA QryID: NC_002952_(-)_2530980_2531156 HitID: NC_016941_(-)_2377543_2377719
rank=1 bitscore=59.7 evalue=4.0E-7 perc_ident=84.48 perc_conserv=89.66 perc_gaps=0.0
query_cvg=100.0 subj_cvg=100.0 alpha=62.41 beta=1 prob=0.9985237956695772
ATGAAAATCGATACATTAAGACAAGCTATAATATATTATTTTGTTGCACTTGTGTTATCA

GCTTTAATGATCTATTTATTTTTAACAAACATTGAGAGAGTTATTTTGTATAGTTGTGTA
TTCACAGTCGTTTATTGGGCAATATATGCCTTTGATGTATATAGAAAGAGAAATTAA
>AA QryID: NC_002952_(+)_2100886_2100993 HitID: NC_007795_(+)_2009610_2009717
rank=1 bitscore=33.1 evalue=21.0 perc_ident=74.29 perc_conserv=74.29 perc_gaps=0.0
query_cvg=100.0 subj_cvg=100.0 alpha=74.29 beta=1 prob=0.9984824076254122
TTGGTTGACTTTCTTTTGTTTGTATTGTTTTTACACGATGCTACGGTATACGTTTCGTTA
CCACTCGTTCAAGCAATTGCTAATGTAACATTTTATTTTTCGTTCTAA
>AA QryID: NC_002952_(-)_2192176_2192268 HitID: NC_009632_(-)_2193769_2193882
rank=1 bitscore=40.4 evalue=0.15 perc_ident=76.67 perc_conserv=90.0 perc_gaps=0.0
query_cvg=100.0 subj_cvg=81.08 alpha=69.41 beta=1 prob=0.9984250005049724
GTGGCACATTTTGTTCCAAAAGATGTAATAAAACTTTACGCATTTTTGCTTTTTATAAAT
TATCAGATTATTATGGAAACAAGGGAGTGGTAA
>AA QryID: NC_002952_(-)_2373448_2373564 HitID: NC_016912_(-)_2153893_2154006
rank=1 bitscore=52.8 evalue=3.0E-5 perc_ident=86.49 perc_conserv=91.89 perc_gaps=0.0
query_cvg=97.37 subj_cvg=100.0 alpha=57.37 beta=1 prob=0.9984085677741077
TTGGTAAATTTATTCAAAAGCTTAAAGGGAAAAATCCTACCTGGAGATATCCAAAAATTA
AATGGACTATTTCCAAAAATAAAGGGCAATCATATGGTGGTTCTTATTGGAAGTTAA
>AA QryID: NC_002952_(+)_1743946_1744146 HitID: NC_018608_(+)_1679928_1680170
rank=1 bitscore=84.7 evalue=2.0E-14 perc_ident=72.73 perc_conserv=74.24 perc_gaps=0.0
query_cvg=100.0 subj_cvg=82.5 alpha=45.86 beta=1 prob=0.998139638432376
ATGTATCACACTCATAATACTTTTCAATTTGAAATACTCTGTTCGCAATTATTAGTTGAG
TTATATCAACCAATACAGTGTAAACTTAAAAATTCCAAACCGTTTAACAGTATATCAGAA
AAAATGATAATTATTTCAATTATTTTTATTAAATTTCATAATGATTCATATAATATTCGA
ATTAAAACATCACAATACTGA
>AA QryID: NC_002952_(+)_2584150_2584257 HitID: NC_016928_(+)_2443867_2443992
rank=1 bitscore=39.3 evalue=0.36 perc_ident=70.73 perc_conserv=75.61 perc_gaps=14.63
query_cvg=100.0 subj_cvg=100.0 alpha=62.85 beta=6 prob=0.9981207532620509
TTGAATCAGCCTCATAACATTAAATTATTTTATCGTACAACTGATTTTTATAAGAGCAAA
GTATCGCATCATAGTAGATACACAAATAATAAAAATGAAACAATTTAA
>AA QryID: NC_002952_(+)_2694285_2694476 HitID: NC_010079_(+)_2665243_2665434
rank=1 bitscore=60.1 evalue=3.0E-7 perc_ident=65.08 perc_conserv=65.08 perc_gaps=0.0
query_cvg=100.0 subj_cvg=100.0 alpha=65.08 beta=1 prob=0.9979606668424708
ATGAAAACGATAATCATGCTGAGCGATAAAGATTTAAATAATAGATTGACCCACGTTCCC
TCAGTCGTATATCCATATGTAATCGTTGTGTTAATGATGAATGCTATAAAGATGATTGAT
AGTCTTAGCATATCATCACTCCTTTTAAGTTATTTTAGATATACGGGGGCGCTTTTGCAA
TCACTATTTTGA
>AA QryID: NC_002952_(-)_2792155_2792268 HitID: NC_017333_(-)_2764115_2764228
rank=1 bitscore=32.3 evalue=43.0 perc_ident=67.57 perc_conserv=67.57 perc_gaps=0.0
query_cvg=100.0 subj_cvg=100.0 alpha=67.57 beta=8 prob=0.9961019911256409
ATGTATAGAAATATCAATATGAAAAATAATATGATTAAAAATAATGAATGGCATACTTAT
AAAGTGTCTAAATATTGGTGGTCAATATTACTTGCAAATACGAATGTTAAGTAA
>AA QryID: NC_002952_(-)_1938855_1938974 HitID: NC_017333_(-)_1928869_1928985
rank=1 bitscore=33.5 evalue=17.0 perc_ident=68.42 perc_conserv=68.42 perc_gaps=0.0
query_cvg=97.44 subj_cvg=100.0 alpha=69.23 beta=7 prob=0.9958393391273374
TTGATGTTGATTTCATTTGTATATAATAATATGGACAACAATAATACTATAAATAGAAAT
CTACATAGTGAATTTGCTATAATTATGTTATTGAAGATTAATGAAAAAGGGGCAGTATAA
>AA QryID: NC_002952_(+)_2866129_2866236 HitID: NC_010079_(+)_2840870_2840977
rank=1 bitscore=32.0 evalue=54.0 perc_ident=65.71 perc_conserv=65.71 perc_gaps=0.0
query_cvg=100.0 subj_cvg=100.0 alpha=65.71 beta=1 prob=0.991834636585919
GTGCCTATAAAAATGCTTAACCAAGATTTTTATATTGAAGTTGTACTTCTTGCACATATT
GTCCTTGCCTTATTATGTAAAGTTATTTTCTTTCTATCTTTTTATTAA
>AA QryID: NC_002952_(+)_2650511_2650633 HitID: NC_007795_(+)_2556559_2556681
rank=1 bitscore=31.6 evalue=73.0 perc_ident=65.0 perc_conserv=65.0 perc_gaps=0.0
query_cvg=100.0 subj_cvg=100.0 alpha=65.0 beta=3 prob=0.9868844347101147
TTGAGCTACATTATCAAATTCACCTTTAAATTTTTTGAAATGACTACCTATATTTCACAG
CTCACTTTAGATATCGAACTTGTTTCTTCAGAGTTACTTGTTATATTATCAGGTTTTATT
TGA
>AA QryID: NC_002952_(-)_2694245_2694415 HitID: NC_017673_(-)_2509135_2509305
rank=1 bitscore=39.3 evalue=0.52 perc_ident=62.5 perc_conserv=62.5 perc_gaps=0.0
query_cvg=100.0 subj_cvg=100.0 alpha=62.5 beta=16 prob=0.9726639000322597
ATGCTAAGACTATCAATCATCTTTATAGCATTCATCATTAACACAACGATTACATATGGA
TATACGACTGAGGGAACGTGGGTCAATCTATTATTTAAATCTTTATCGCTCAGCATGATT

```
ATCGTTTTCATGTTCTACTATATTCGTTTTGTTATTGAGAAAAAGCGTTAA
>AA QryID: NC_002952_(+)_2182140_2182265 HitID: NC_007622_(+)_2043598_2043795
rank=1 bitscore=51.6 evalue=7.0E-5 perc_ident=57.89 perc_conserv=57.89 perc_gaps=31.58
query_cvg=95.12 subj_cvg=87.69 alpha=65.63 beta=45 prob=0.9545479076810539
TTGTCTGTAGAAATTGGTGTTCCAATTTCTCTATGTTGGGGCCCCATCAACTACTGTCAA
TATAACATTGTAGAGCCTAGGACTTTGATTTTATGTCCTGGGCTTGTTTTTTGCGTTTCA
ACATAA
>AA QryID: NC_002952_(+)_2078802_2078942 HitID: NC_002745_(-)_1142748_1142879
rank=1 bitscore=37.4 evalue=2.3 perc_ident=65.79 perc_conserv=78.95 perc_gaps=5.26
query_cvg=80.43 subj_cvg=86.05 alpha=56.24 beta=9 prob=0.8384311262378246
TTGGTTTCGTCTAATAGGAGTGGGACAGAAATAATATTTAACAAAATTTATTTCGTTCTA
CCCCAACTTACATTGTCTGTAGAATTTCTTTTCAAAATTCTCTATGTTTGGGGCCCCACC
CCAACTTGCACATTATTGTAA
>AA QryID: NC_002952_(+)_1664182_1664361 HitID: NC_017343_(+)_1507850_1508029
rank=1 bitscore=37.4 evalue=2.2 perc_ident=59.32 perc_conserv=61.02 perc_gaps=0.0
query_cvg=100.0 subj_cvg=100.0 alpha=45.35 beta=13 prob=0.8213248733462539
TTGCTAGTTATATCGTCTCAAGTTAAAAATTTTATATCTTATGTCGTAATTATTAATACA
AAGGTTATTCATTTGGAGGCACACAAAATGCAAAATAAAGTTTTAAGAATTATCATTATC
GTTATGCTTGTATCAGTTGTATTAGCATTGTTATTAACGAGTATCATTCCAATTTTATAA
>AA QryID: NC_002952_(-)_1136839_1136967 HitID: NC_002953_(-)_859569_859811
rank=1 bitscore=52.4 evalue=4.0E-5 perc_ident=47.3 perc_conserv=47.3 perc_gaps=45.95
query_cvg=95.24 subj_cvg=92.5 alpha=63.54 beta=21 prob=0.7749474344178056
TTGACTGAACGAAAATGCGCTTGTAACAAGCTTTTTTCAATTCTAGTCAACCTTGCCGGG
GTGGGACGACGAAATAAATTTTGCGAAAATATCATTTCTGTCCCACTCCCTTTTTATATC
AATATTTAA
```
