# Supplementary material for: OCCAM: prediction of small ORFs in bacterial genomes by means of a target-decoy database approach and machine learning techniques
Source: Database (Oxford). 2020 Nov 18;2020:baaa067. doi: 10.1093/database/baaa067 (PMC7673341; doi:10.1093/database/baaa067)
Supplement: baaa067_Supp [file baaa067_supp.zip › Supporting_File_S5.pdf]

Search hit details of all small ORFs found as Missing Genes by OCCAM in the eight tested bacteria (for only 4 bacteria there were missing genes reported)

The head of each search hit indicates class (MG or GA in this case, if the GA subject is from a different family), query genome ID, query strand (positive or negative), query coordinates indicating the ORF start and end , subject (or hit) genome ID, subject strand, and subject coordinates.

It is additionally shown that the search hit was the best one found (rank=1, i.e., top-ranked), followed by scores generated by BLAST and OCCAM, including the probability generated by the ML algorithms. Also, the DNA sequence and its translation are shown. Those ORFs for which we could validate with RNA-Seq and ribosome profiling data (Table 6 in the manuscript) are marked with "***" after the probability value.

- ***Actinobacillus pleuropneumoniae*** **serovar 5b L20 uid58789**

vsORFs

>MG1 QryID: NC_009053_(+)_816047_816130 HitID: NC_013520_(-)_1407909_1407995
rank=1 bitscore=87.2 evalue=8.0E-16 perc_ident=96.15 perc_conserv=96.15 perc_gaps=0.0
query_cvg=96.3 subj_cvg=92.86 alpha=18.59 beta=1 prob=0.9997953483075565

GTGCATGTGCTGTGCTGTGCTGTGCTGTGCTGTGCTGTGCTGTGCTGTGCTGTGCTGTGC
TGTGCTGTGCTGTGCTGTCATTAG

MHVLCCAVLCCAVLCCAVLCCAVLCCH

>MG2 QryID: NC_009053_(-)_2059896_2059961 HitID: NC_017154_(+)_1236877_1236942
rank=1 bitscore=57.9 evalue=3.0E-7 perc_ident=90.0 perc_conserv=90.0 perc_gaps=0.0
query_cvg=95.24 subj_cvg=95.24 alpha=0.0 beta=6 prob=0.9977285883066762

TTGGTGGAGCTGGGGGGATTTGAACCCCCGTCCGAAATTACTCCATCTTCAGCACTACAC
GTTTAG

MVELGGFEPPSEITPSSALHV

>MG3 QryID: NC_009053_(-)_1763309_1763356 HitID: NC_011184_(-)_2858778_2858825
rank=1 bitscore=55.4 evalue=2.0E-6 perc_ident=100.0 perc_conserv=100.0 perc_gaps=0.0
query_cvg=100.0 subj_cvg=100.0 alpha=0.0 beta=1 prob=0.9953336386418956

TTGAAGAGTTTGATCATGGCTCAGATTGAACGCTGGCGGCAGGCTTAA

MKSLIMAQIERWRQA

>MG4 QryID: NC_009053_(+)_1346431_1346472 HitID: NC_008563_(-)_4833378_4833419
rank=1 bitscore=40.5 evalue=0.055 perc_ident=92.31 perc_conserv=92.31 perc_gaps=0.0
query_cvg=100.0 subj_cvg=100.0 alpha=0.0 beta=1 prob=0.7243889481693003

GTGATACGGCTAGGAACAATCTTGCCGCTTTCAGAAATGTAG

MIRLGTILPLSEM

No sORFs

- ***Escherichia coli* K12 substr MG**

vsORFs

>GA1 QryID: NC_000913_(-)_365552_365593 HitID: NC_014976_(-)_2247517_2247558 rank=1 bitscore=46.0 evalue=0.001 perc_ident=100.0 perc_conserv=100.0 perc_gaps=0.0 query_cvg=100.0 subj_cvg=100.0 alpha=32.87 beta=5 prob=0.8859503934751499 ***

ATGCTTCCGGCTCGTATGTTGTGTGGAATTGTGAGCGGATAA

MLPARMLCGIVSG

No sORFs

- ***Mycobacterium tuberculosis* H37Rv uid57777**

vsORFs

>MG1 QryID: NC_000962_(+)_3726000_3726044 HitID: NC_009142_(-)_6539108_6539152 rank=1 bitscore=47.3 evalue=5.0E-4 perc_ident=100.0 perc_conserv=100.0 perc_gaps=0.0 query_cvg=100.0 subj_cvg=100.0 alpha=49.35 beta=9 prob=0.9999404486345185

TTGCTGGCCGGCAACCCTCCAACCGCGGTGGGGTGCCCCGGGTGA

MLAGNPPTAVGCPG

No sORFs

- ***Pseudomonas aeruginosa* PAO1**

vsORFs

>GA1 QryID: NC_002516_(-)_354512_354589 HitID: NC_008782_(+)_2232344_2232421 rank=1 bitscore=80.4 evalue=9.0E-14 perc_ident=100.0 perc_conserv=100.0 perc_gaps=0.0 query_cvg=100.0 subj_cvg=100.0 alpha=52.95 beta=1 prob=0.9999726375942918 ***

ATGCCCGCAACCCCTGGAAAGACCGCATGCAACCCAGTAGGTTCGCGATTGTCCAGGACG
CGGGCCCGGCTAAGCTGA

MPATPGKTACNPVGSRLSRTRARLS

>MG1 QryID: NC_002516_(+)_352323_352376 HitID: NC_008782_(-)_2234557_2234610 rank=1 bitscore=59.2 evalue=1.0E-7 perc_ident=100.0 perc_conserv=100.0 perc_gaps=0.0 query_cvg=100.0 subj_cvg=100.0 alpha=28.92 beta=1 prob=0.9994660315968504

TTGGGTCGAGGGCGGGTTTCCCCGGTGCTTCCCGGCTACTGTTACCGGGCGTGA

MGRGRVSPVLPGYCYRA

>GA2 QryID: NC_002516_(+)_354476_354520 HitID: NC_008782_(-)_2232413_2232457
rank=1 bitscore=48.1 evalue=3.0E-4 perc_ident=100.0 perc_conserv=100.0 perc_gaps=0.0
query_cvg=100.0 subj_cvg=100.0 alpha=40.0 beta=4 prob=0.9397620101183046 ***

GTGGGAATGAACGTCGGTCGGGCCTATGCTGTTCGTTCAGCTTAG

MGMNVGRAYAVRSA

>GA3 QryID: NC_002516_(+)_3099738_3099779 HitID: NC_010682_(+)_2079443_2079484
rank=1 bitscore=45.2 evalue=0.002 perc_ident=100.0 perc_conserv=100.0 perc_gaps=0.0
query_cvg=100.0 subj_cvg=100.0 alpha=53.54 beta=106 prob=0.9274782590102473 ***

TTGACCGCGCACAAGTCGCTGACTTCGCCGATGGTGAAGTAG

MTAHKSLTSPMVK

>MG2 QryID: NC_002516_(-)_352244_352285 HitID: NC_008782_(+)_2234648_2234689
rank=1 bitscore=43.5 evalue=0.007 perc_ident=100.0 perc_conserv=100.0 perc_gaps=0.0
query_cvg=100.0 subj_cvg=100.0 alpha=24.48 beta=1 prob=0.9261306000739523

ATGGCGGGGACCAGGGCTATTTCTTGCCGATCCTTAGGATAG

MAGTRAISCRSLG

sORFs

>GA1 QryID: NC_002516_(+)_354562_354729 HitID: NC_008782_(-)_2232204_2232371
rank=1 bitscore=84.0 evalue=2.0E-14 perc_ident=98.18 perc_conserv=98.18 perc_gaps=0.0
query_cvg=100.0 subj_cvg=100.0 alpha=98.18 beta=3 prob=0.998394002678091 ***

ATGCGGTCTTTCCAGGGGTTGCGGGCATTGACCCGGATTATGTGCGCGAGCGCCGCTCTA
ACACGGGCTTCGCAGTGGAAATCGCTGTGCCAGCGATGCCTTTCCCTGATTCGGAAGGCA
CGAAGAGTGCCGGGGAAATTCCGTCCAGTGGTGTTTCCCGGGTGCTAA

MRSFQGLRALTRIMCASAALTRASQWKSLCQRCLSLIRKARRVPGKFRPVVFPGC

>MG1 QryID: NC_002516_(+)_352185_352316 HitID: NC_008782_(-)_2234617_2234748
rank=1 bitscore=62.8 evalue=3.0E-8 perc_ident=97.67 perc_conserv=97.67 perc_gaps=0.0
query_cvg=100.0 subj_cvg=100.0 alpha=97.67 beta=1 prob=0.9983719548589782 ***

TTGCGCGGGAAGCCAGTCTGCTCGGGGCGGCGGAGGTTCGCCATGGGCCGCCCGCAGTTC
TATCCTAAGGATCGGCAAGAAATAGCCCTGGTCCCCGCCATACGGGCGCTGGAGCGCTAC
CGCGAGGCCTAA

MRGKPVCSGRRRFAMGRPQFYPKDRQEIALVPAIRALERYREA

>MG2 QryID: NC_002516_(-)_352249_352380 HitID: NC_008782_(+)_2234553_2234684
rank=1 bitscore=52.8 evalue=3.0E-5 perc_ident=97.67 perc_conserv=97.67 perc_gaps=0.0
query_cvg=100.0 subj_cvg=100.0 alpha=97.67 beta=1 prob=0.9982745293677642 ***

ATGGTCACGCCCGGTAACAGTAGCCGGGAAGCACCGGGGAAACCCGCCCTCGACCCAACC
GGAGTTAGGCCTCGCGGTAGCGCTCCAGCGCCCGTATGGCGGGGACCAGGGCTATTTCTT
GCCGATCCTTAG

MVTPGNSSREAPGKPALDPTGVRPRGSAPAPVWRGPGLFLADP
