# Supplementary material for: OCCAM: prediction of small ORFs in bacterial genomes by means of a target-decoy database approach and machine learning techniques
Source: Database (Oxford). 2020 Nov 18;2020:baaa067. doi: 10.1093/database/baaa067 (PMC7673341; doi:10.1093/database/baaa067)
Supplement: baaa067_Supp [file baaa067_supp.zip › Supporting_File_S6.pdf]

Accession numbers of studies and respective experiments in SRA, involving RNA-Seq data for the organisms for which OCCAM identified MGs.

These data were used in our work to verify whether the found small ORFs are expressed by the given organism.

- *Actinobacillus pleuropneumoniae* serovar 5b L20 uid58789

  No RNA-Seq experiment could be found.

- *Pseudomonas aeruginosa* PAO1

Study	Read size	Experiments
SRP076720	51	SRX1853532<br>SRX1853533<br>SRX1853292
SRP057994	51	SRX1017131<br>SRX1017132
SRP018866	50	SRX245811<br>SRX245812<br>SRX245813
ERP117503	51	ERX3558798<br>ERX3558799<br>ERX3558800<br>ERX3558801<br>ERX3558802<br>ERX3558803<br>ERX3558804<br>ERX3558805

- *Escherichia coli* K12 substr MG

Study	Read size	Experiments
ERP016032	51	ERX1521038<br>ERX1521039<br>ERX1521040<br>ERX1521041<br>ERX1521042<br>ERX1521043<br>ERX1521044<br>ERX1521045

- *Mycobacterium tuberculosis* H37Rv uid57777

Study	Read size	Experiments
ERP002485	50	ERX237496<br>ERX237497<br>ERX237500<br>ERX237501<br>ERX237502<br>ERX237503
