# Supplementary material for: OCCAM: prediction of small ORFs in bacterial genomes by means of a target-decoy database approach and machine learning techniques
Source: Database (Oxford). 2020 Nov 18;2020:baaa067. doi: 10.1093/database/baaa067 (PMC7673341; doi:10.1093/database/baaa067)
Supplement: baaa067_Supp [file baaa067_supp.zip › Supporting_File_S7.pdf]

**Accession numbers of studies and respective experiments in SRA, involving ribosome profiling data for the organisms for which OCCAM identified MGs.**

These data were used in our work to verify whether the found small ORFs are translated by the given organism.

- *Actinobacillus pleuropneumoniae* serovar 5b L20 uid58789

  No ribosome profiling experiment could be found.

- *Pseudomonas aeruginosa* PAO1

| Study | Read size | Experiments |
|---|---|---|
| SRP102099 | 50 | SRX2652926<br>SRX2652928<br>SRX2652930<br>SRX2652932<br>SRX2652934<br>SRX2652936 |

- *Escherichia coli* K12 substr MG

| Study | Read size | Experiments |
|---|---|---|
| SRP117035 | 51 | SRX3157121<br>SRX3157122<br>SRX3157123<br>SRX3157124<br>SRX3157125<br>SRX3157126<br>SRX3157127<br>SRX3157128<br>SRX3157129<br>SRX3157130<br>SRX3157131 |

- *Mycobacterium tuberculosis* H37Rv uid57777

  No ribosome profiling experiment could be found.
