# Supplementary figures and images for: OCCAM: prediction of small ORFs in bacterial genomes by means of a target-decoy database approach and machine learning techniques
Source: Database (Oxford). 2020 Nov 18;2020:baaa067. doi: 10.1093/database/baaa067 (PMC7673341; doi:10.1093/database/baaa067)

### Start codon distribution in the datasets of the organisms used in this work

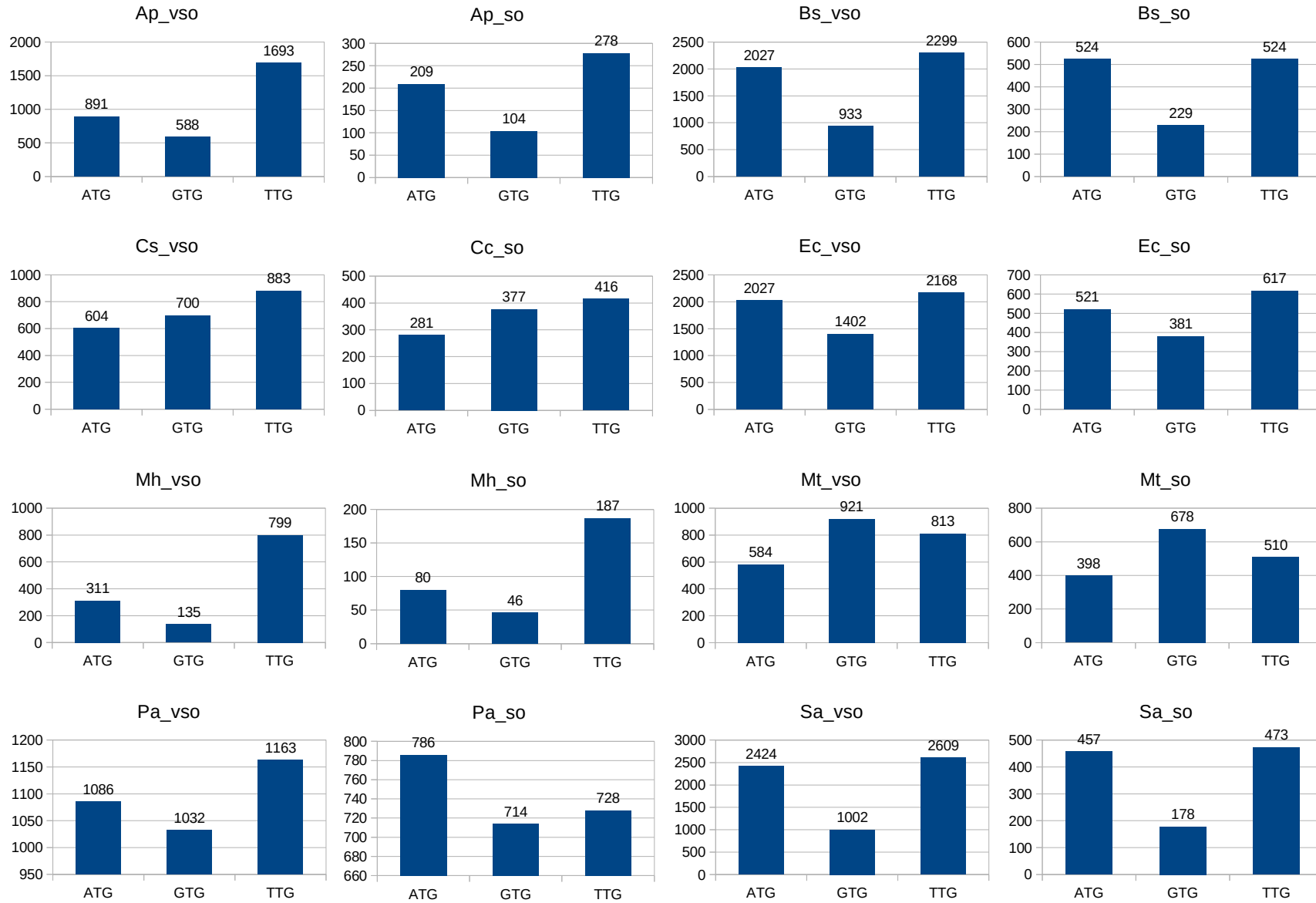

Supplement: baaa067_Supp [file baaa067_supp.zip › Supporting_File_S2.pdf]
